# Adaptive Synthesis of Functional Amphiphilic Dendrons as a Novel Approach to Artificial Supramolecular Objects

**DOI:** 10.3390/ijms23042114

**Published:** 2022-02-14

**Authors:** Antonín Edr, Dominika Wrobel, Alena Krupková, Lucie Červenková Šťastná, Petra Cuřínová, Aleš Novák, Jan Malý, Jitka Kalasová, Jan Malý, Marek Malý, Tomáš Strašák

**Affiliations:** 1 Institute of Chemical Process Fundamentals of the CAS, v.v.i., Rozvojová 135, 16502 Prague, Czech Republic; edr@icpf.cas.cz (A.E.); krupkova@icpf.cas.cz (A.K.); stastna@icpf.cas.cz (L.Č.Š.); curinova@icpf.cas.cz (P.C.); 2 Faculty of Science, J.E. Purkyně University in Ústí nad Labem, Pasteurova 15, 40096 Ústí nad Labem, Czech Republic; dominika_wrobel@o2.pl (D.W.); alesnovak.ul@gmail.com (A.N.); malyjalga@seznam.cz (J.M.); 3 Faculty of Chemical Technology, University of Chemistry and Technology Prague, Technická 5, 16828 Prague 6, Czech Republic; jan.maly.33@gmail.com (J.M.); Jitka.Kalasova@vscht.cz (J.K.)

**Keywords:** carbosilane, dendrons, amphiphiles, DLS, zeta potential, micelles, molecular dynamics, computer modeling

## Abstract

Supramolecular structures, such as micelles, liposomes, polymerosomes or dendrimerosomes, are widely studied and used as drug delivery systems. The behavior of amphiphilic building blocks strongly depends on their spatial distribution and shape of polar and nonpolar component. This report is focused on the development of new versatile synthetic protocols for amphiphilic carbosilane dendrons (amp-CS-DDNs) capable of self-assembly to regular micelles and other supramolecular objects. The presented strategy enables the fine modification of amphiphilic structure in several ways and also enables the facile connection of a desired functionality. DLS experiments demonstrated correlations between structural parameters of amp-CS-DDNs and the size of formed nanoparticles. For detailed information about the organization and spatial distribution of amp-CS-DDNs assemblies, computer simulation models were studied by using molecular dynamics in explicit water.

## 1. Introduction

The development of biotechnological protocols based on branched amphiphilic molecules is a modern trend that focuses on the fabrication of systems for various pharmaceutical and biomedical applications and material sciences [1,2]. These molecules have the capacity to self-assemble into different nature-mimicking morphological objects, from micelles to vesicles, with the ability to execute profitable functions [3,4]. The potential of such structures as antimicrobial and bio-imaging agents, supramolecular catalysts, nanoparticle stabilizers and especially drug and gene nanocarriers, was investigated and tested [5,6,7]. While lipids are fundamental building blocks of cell membranes and organelles, their limitations in the fabrication of model membranes encourage researchers to design novel synthetic amphiphiles. Their structure can be carefully tailored to meet numerous specific demands. Cationic head groups of amphiphiles provide their high affinity toward biopolyanions, such as nucleic acids, studied in the field of gene therapy [8]. Less common anionic amphiphiles were studied, e.g., as delivery systems for cationic peptides [9] and antibiotics [10]. Neutral or non-ionic surfactants benefit from their low toxicity, which renders them suitable for the development of biocompatible nanocarriers. For this purpose, carbohydrates and oligo- and short poly(ethylene glycol) chains (OEGs or PEGs) are frequently incorporated in a polar hydrophilic domain of neutral amphiphiles.

Branched and dendritic amphiphiles have attracted attention in the field of nanoscience, due to unique structural characteristics, such as their high degree of branching, resulting in a high number of end groups at the periphery—the feature that is absent in natural analogs. The branched structure of most dendrons is derived from the corresponding dendrimers [11,12]. For example, poly(amidoamine) (PAMAM)-based amphiphiles were developed as building blocks of nanomicelles for the delivery of doxorubicin or siRNA into cancer cells [13,14]. Fréchet-type aryl ether or ester dendrons provide a structural motive based on aromatic cores with varying multiplicity [15,16]. Majoral et al. synthetized various dendrons stemming from the structure of phosphorus dendrimers [17,18]. Very recent examples of such amphiphilic dendrons were designed as antiproliferative agents for the use in the field of theranostics [19]. Cationic and anionic carbosilane (CS) dendritic micelles, using silicon as a branching point, also demonstrated their ability to form complexes with therapeutic biomacromolecules, such as siRNA, and showed a high loading capacity for drugs, such as procaine, thus implying their potential as nanocarriers for therapeutics [20,21].

In most cases, methods developed for the preparation of dendrons manifest a higher level of complexity, exploiting a broader arsenal of chemical transformations than those used for the preparation of dendrimers. Synthetic protocols have to respect the presence of different functional groups in distinct parts of the molecule, typically at the periphery and in the focal point. Interesting examples of water-soluble polyglycerol dendrons with two orthogonal functional groups (azide and amine) in the focal point were reported by Zimmerman et al. [22]. The design and preparation of functionalized amphiphilic dendrons, in which the two amphipatic parts are appended with an auxiliary component (group or molecule) of additional function, are even more challenging. Examples are rather rare, but a fundamental progress in this way was made by Percec and coworkers. They have developed new synthetic pathways leading to libraries of amphiphilic dendrons or Janus dendrimers decorated with saccharides for studying interactions with lectines [23,24] or fluorescent probes enabling monitoring in given environment [25,26]. The strategy is based on sequential functionalization of selectively protected pentaerythritol or tris(hydroxymethyl)aminomethane (Trizma) as tetravalent cores by segments of hydrophobic and hydrophilic character, and by the particular functional molecule.

Our long-term interest has been focused on CS dendrimers with a well-defined structure that express multivalent presentation of functional moieties at their periphery [27,28,29,30,31] and, due to the incompatibility of their hydrophobic interior with polar periphery, can be considered as unimolecular micelles [32]. Here we turn our attention to a development of new versatile synthetic protocols for amphiphilic building blocks capable of self-assembly to regular micelles and other supramolecular objects. As previous findings pointed out that the self-assembly pattern of dendrons is controlled by structural parameters, such as size, shape, flexibility, and surface chemistry, the flexibility of the presented synthetic approach can be advantageous [33]. The versatility of our method is demonstrated on the preparation of three libraries of amphiphilic carbosilane dendrons (amp-CS-DDNs) systematically varying selected structural parameters. DLS experiments and a detailed computational study were used to follow the influence of structure changes on the self-assembly behavior of the dendrons.

## 2. Results and Discussion

### 2.1. Synthesis and Characterization of Carbosilane Amphiphilic Dendrons

Presented synthetic methodology allows for a high-yielding preparation of a novel type of amphiphilic dendrons with uniform structure bearing either ionic (cationic or anionic) or non-ionic groups. The tuning of the hydrophobic domain is possible by varying the length and number of aliphatic chains. In addition, the synthetic pathway leading to amp-CS-DDNs with an added functionality is also investigated. In three consecutive parts of the synthetic section, we describe representative libraries of amphiphilic compounds. Each part demonstrates a specific direction in structure modulation.

**Scheme 1:**
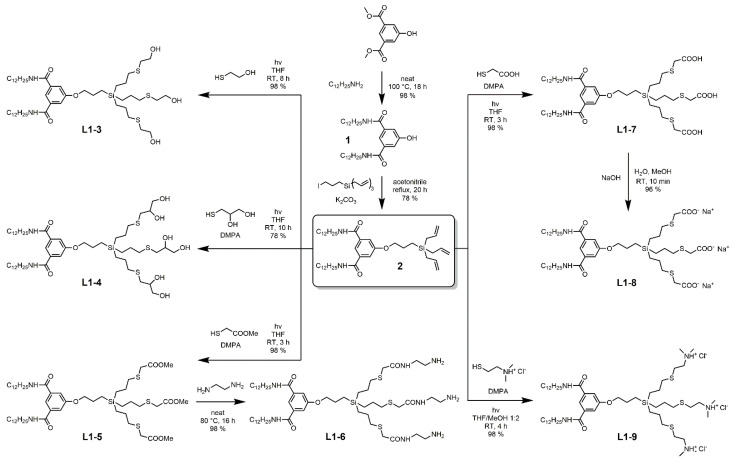
Synthesis of amp-CS-DDNs containing two dodecyl chains and one polar wedge (Library 1).

The first pathway is directed to produce amphiphilic compounds of Library 1 (**L1**–**X**) with the same hydrophobic domain and variable hydrophilic units (Scheme 1). The synthesis starts from dimethyl 5-hydroxyisophthalate, a trivalent bifunctional AB_2_ aromatic core. First, aminolysis of both methylester groups by 1-dodecylamine provides hydrophobic segment **1**, carrying two alkyl chains and a reactive hydroxyl. Second, a three-armed carbosilane building block is attached as a scaffold for the polar part of the molecule. Obtained triallylic compound **2** serves as a key component for the preparation of a library of amphiphilic dendrons in the next one or two steps. The attachment of target end groups is achieved by an efficient and regioselective photocatalytic thiol-ene coupling (TEC), a radical addition of polar thiols to double bonds, and eventual subsequent modification [34]. Examples of non-ionic amphiphiles **L1**–**3** and **L1**–**4** bearing multiple hydroxyls are readily accessible by using the corresponding mercaptoalcohols. Primary amines can be introduced by a reaction of polyester **L1**–**5** with ethylenediamine, such as in the case of **L1**–**6**. Ionic amphiphilic compounds, both anionic **L1**–**8** (obtained after the neutralization of **L1**–**7**) and cationic **L1**–**9,** were prepared in high yield. In our hands, TEC proved to be a regioselective reaction forming no side-products and giving (almost) quantitative yields, usually under mild conditions. In most cases, the excess reagent is readily separable by using extraction or evaporation. Only in the case of compound-**L1**–**9**-bearing dimethylammonium groups, the separation requires organic solvent nanofiltration (OSN) [35,36,37]—an effective method which we previously exploited for the purification of CS-dendrimers [27,28,38,39]. The key feature of synthetic amphiphiles, essential for their investigation in biological environments, is their water-solubility. In this respect, ionic derivatives (**L1**–**8** and **L1**–**9**) prove to be promising candidates. All other substrates were insoluble or poorly soluble in water. Cationic nanoparticles show high affinity toward biopolyanions, such as cell membranes, nucleic acids, and intracellular organelles. Thus, we selected ammonium head groups as hydrophilic moieties for Library 2.

The second part of our synthetic effort was focused on structural modifications balancing the hydrophilic and hydrophobic domain of the amphiphiles through the number and length of alkyl chains and the number of polar dendritic wedges. Thus, Library 2 is defined by identical ammonium branched units, combined at a variable ratio with the hydrocarbon chains, and connected by the bivalent or trivalent aromatic core. Dendron **L2**–**12**, structurally related to **L1**–**9** with prolonged eighteen-carbon hydrophobic chains, was prepared in a same way via the corresponding intermediates **10** and **11**. Dendrons **L2**–**15a** and **L2**–**15b** with one-chain nonpolar domain were prepared analogically starting from bivalent methyl 4-hydroxybenzoate. Interestingly, its aminolysis by 1-dodecylamine yields a salt soluble in chloroform and confirmed by NMR and ESI–TOF-MS. This salt had to be converted to free phenol **13a**, and this additional step and subsequent purification resulted in a significant material loss. Another drop in yield was observed in the last step, and it was due to OSN purification. As the target dendron **L2**–**15a** is the smallest and least branched one from the whole series, it is not fully retained by the 1kDa nanofiltration membrane, thus leading to decreased substrate recovery. This is, however, not the case of derivative **L2**–**15b** with longer hydrocarbon chain. Dendrons **L2**–**19a** and **L2**–**19b** with two carbosilane wedges were prepared by an altered synthetic sequence starting from methyl 3,5-dihydroxybenzoate. In this case, direct aminolysis leads to an inseparable mixture of products. Therefore, the reaction order was reversed and the dendritic wedges were introduced first, followed by a basic hydrolysis of ester group of the resulting dendritic module **16** to obtain the key module **17** in excellent yield. Compound **17** was subjected to amidic coupling with 1-dodecylamine and 1-octadecylamine, respectively, and the obtained dendrons **L2**–**18a** and **L2**–**18b** were finally functionalized by TEC. To achieve a sufficient rate of TEC reaction, a proper ratio of THF and MeOH must have been found for each substrate (see Supplementary Materials [40,41,42,43,44,45,46,47,48,49,50,51,52,53,54,55,56]).

**Scheme 2:**
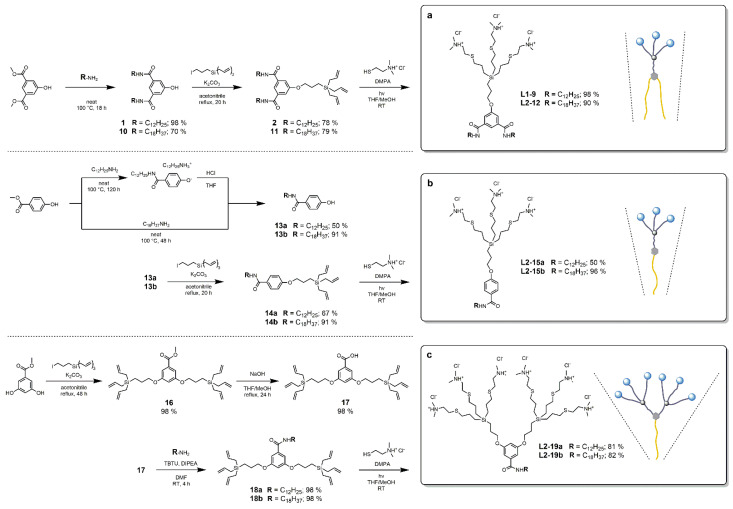
Synthesis of amp-CS-DDNs of Library 2 containing ammonium-terminated dendritic wedges and alkyl chains in different ratio: one dendritic wedge and two alkyl chains (**a**), one dendritic wedge and one alkyl chain (**b**), and two dendritic wedges and one alkyl chain (**c**).

It was previously found that the supramolecular behavior of amphiphilic compounds depends on their spatial distribution and shape of polar and nonpolar component [3]. Thus, the prepared series follows a simple trend from the most cylindrical to the most conical structure, as illustrated in Scheme 2.

**Scheme 3:**
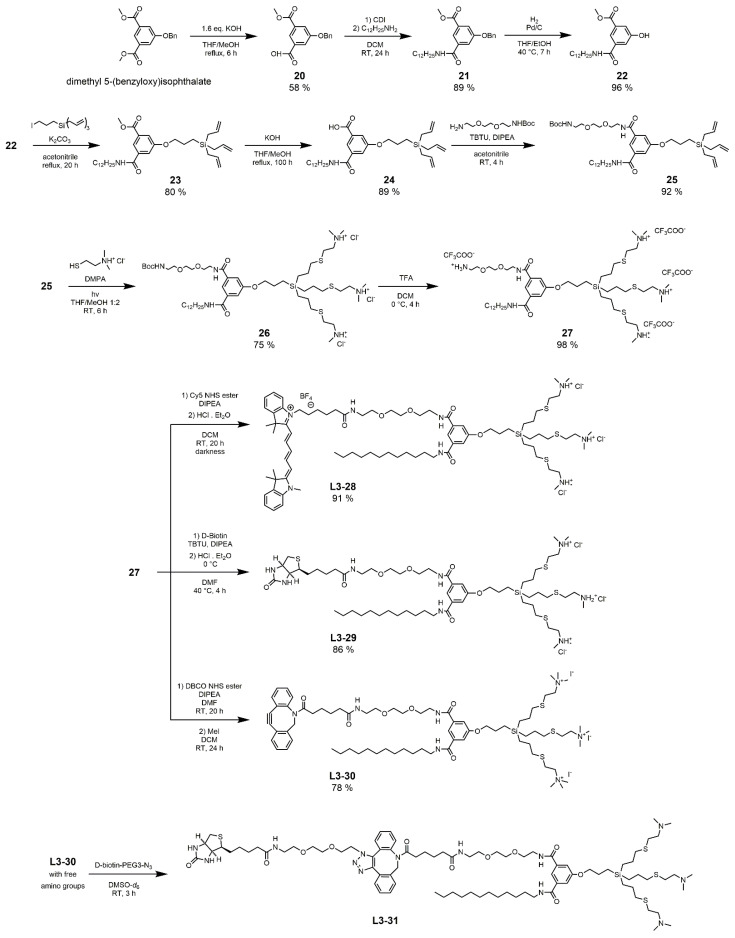
Synthesis of functionalized amp-CS-DDNs (Library 3).

To better address the needs of diverse applications, our effort was finally directed to the construction of amphiphiles with added functionality. We targeted a derivative possessing a free reactive group and, thus, the ability to subsequently introduce the functional moieties, as outlined in Scheme 3.

Our synthetic endeavor started with an optimization of reaction conditions of basic ester hydrolysis to prepare a partially hydrolyzed carboxylic acid monoester **20**. This compound was previously described without sufficient synthetic details [57]. The highest yield was obtained by refluxing the reaction mixture for 6 h, using 1.6 equivalents of hydroxide. Compound **20** is a universal building block bearing three orthogonal functional groups and a wide range of possible modifications. In this case, 1-dodecylamine was attached to obtain ester amide **21**. The hydroxyl group was deprotected, yielding phenol **22**, which was reacted with the branched triallyl unit to yield dendron **23**. The hydrolysis of the second ester group afforded carboxylic acid **24b**, to which a partially protected oligoethyleneglycol diamine was connected by amidic coupling, yielding dendron **25.** Ammonium groups were attached to the periphery of **25** by TEC, leading to amphiphilic structure **26**, but other polar groups can be principally introduced as well. The terminal amino group of the OEG linker was deprotected to give key substrate **27**, ready for conjugation of diverse functional molecules through an amide coupling. As applicable examples, we selected a blue fluorescent probe Cyanine-5 and biotin, which can form a strong noncovalent bond with avidine proteins, and the functional amphiphiles **L3**–**28** and **L3**–**29** were obtained in high yields. A versatile derivative, **L3**–**30,** equipped with a strained aza-dibenzocyclooctyne (DBCO), was also prepared. To avoid the acidic conditions during HCl protonation that are potentially incompatible with the highly reactive triple bond in the dendron structure, the cationic nature of head groups was achieved, in this case, by the quaternization with methyl iodide. A biotin-modified compound, **L3**–**31**, was prepared as an example of **L3**–**30** utilization by a simple non-catalyzed reaction. The formation of the product was confirmed by ESI–TOF-MS.

### 2.2. Dynamic Light Scattering and Zeta Potential

As the size of nanoparticles and their surface charge were previously identified as important factors influencing both the cellular uptake [58] and cytotoxicity effects [29,31,59,60,61,62,63,64], the behavior of prepared amphiphilic dendrons in water was investigated in terms of hydrodynamic size and Zeta potential. These parameters are crucial in the case of nanoparticles intended for biomedical applications. Generally, the hydrodynamic size obtained for all molecules implies that carbosilane amphiphilic dendrons create larger structures in water (Table 1). Diverse composition of amphiphilic molecules was reflected in their non-uniform behavior. Cationic molecules with two alkyl chains (**L1**–**9** and **L1**–**12**) formed assemblies whose size decreased with the increasing concentration; the values obtained for the highest concentration applied were 8 ± 1 nm, 7 ± 1 nm, and 3 ± 1 nm, respectively. On the contrary, the behavior of cationic amphiphiles containing only one alkyl chain per molecule (**L2**–**15a** and **L2**–**15b**) was concentration independent. The average hydrodynamic diameter across the investigated concentration range was 75 ± 14 nm for **L2**–**15a** and 23 ± 3 nm for **L2**–**15b**. A similar behavior in aqueous solution was observed for the anionic two-chain derivative **L1**–**8** with an average size 6 ± 1 nm. Amphiphilic dendrons with two hydrophilic branches and one hydrophobic chain formed nanoparticles of concentration-independent size (**L2**–**19b**—average size 6 ± 1 nm), at least after exceeding certain concentration threshold (**L2**–**19a** reached the highest value of 63 ± 11 nm).

For the majority of amphiphilic dendrons, the hydrodynamic size of the observed structures at the highest concentration was relatively small (3–8 nm). Only in the case of three amphiphilic dendrons (**L2**–**15a**, **L2**–**15b**, and **L2**–**19a**) were larger (28–70 nm) structures observed. The results suggest the self-assembly of investigated amphiphilic dendrons; the formation of micelles, which, in some cases, may aggregate into larger structures, can be anticipated. The self-assembling nature of amphiphilic structures is commonly recognized [65], and several amphiphilic dendrons capable of self-assembly were already described. For example, amphiphilic fluorescent phosphorus dendron–based micelles formed in aqueous solutions possessed diameters of approximately 30–40 nm [19]. Similar sizes (~50 nm) were observed in solutions of thermosensitive poly(benzyl ether) dendrons [66], as well as for amphiphilic-polyphenylene-dendronized proteins [67].

As expected, on account of previous investigations [19,68,69,70], the Zeta potential of self-assembled structures was dependent on the nature of functional end groups of the hydrophilic dendritic domains, forming the nanoparticle surface. Thus, the values obtained for dendrons bearing cationic ammonium groups were highly cationic (39–72 mV), whereas the amphiphilic dendron **L1**–**8** containing anionic carboxyl groups gave rise to negatively charged micelles (−27.2 ± 3.9 mV).

### 2.3. Computational Study

The behavior of cationic dendrons **L1**–**9**, **L2**–**12**, **L2**–**15a**, **L2**–**15b**, **L2**–**19a**, and **L2**–**19b** in water was also studied by means of molecular simulations. The simulations showed that all of these dendrons assembled in water into micellar clusters. The final simulated systems typically contain about 10–30 micellar clusters. The “in silico” approach, which allowed us to study the realistic models of molecular systems at atomic resolution, also provided detailed information concerning the structure of these clusters. It can be deduced how various structural parameters of a particular dendron (the number of aliphatic chains and their length, the number of ionic end groups) affect the structure of clusters. Due to high computational time requirements, the time and dimensional simulation parameters do not reflect the conditions of an experiment (the simulations cover only 400 ns time interval, simulated systems consist of a 300 molecules of dendrons, and dendron concentrations are set to much higher values (approximately 100 mM) than those used in DLS experiments, due to the size limits of the simulation box (see Supplementary Figure S1)). Thus, the simulated molecular cluster sizes cannot be directly compared to the data obtained from DLS. Nevertheless, a good agreement can be found for some dendrons (**L1**–**9**, **L2**–**12**, and **L2**–**19b**), especially at the highest concentration used (100 μM). In other cases, the outputs of molecular simulations (i.e., micellar clusters) should be understood as building blocks for the formation of larger aggregates.

**Figure 1:**
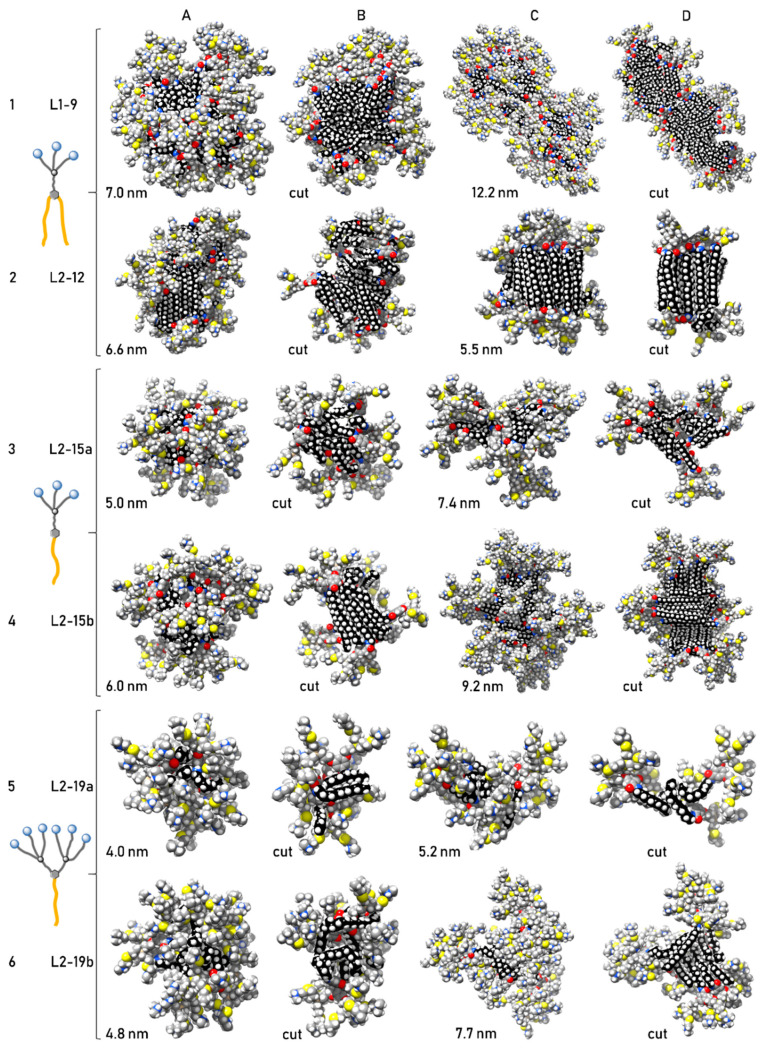
Typical micellar structures composed of ammonium-terminated dendrons **L1**–**9**, **L2**–**12**, **L2**–**15a**, **L2**–**15b**, **L2**–**19a** and **L2**–**19b** (lines 1–6 in that order), obtained from molecular dynamics simulations. For each dendron, two representative micellar structures are shown (columns (**A**,**C**)). Each dendron cluster is appended by its cut (on the right from cluster figure; columns (**B**,**D**)), realized by removing a surface layer of dendrons from the cluster. Aliphatic chain carbons are highlighted in black. Colors: C—gray/black; O—red; H—white; Si—beige; N—blue; S—yellow.

Figure 1 shows typical micellar clusters consisting of individual dendrons (two representative structures for each dendron, including the indication of their dimensions). To get a better view of the internal framework of the particles, cuts of each cluster are included, created by removing a surface layer of dendrons from the cluster. Figure 1 depicts micellar clusters obtained by simulations of cationic ammonium-terminated dendrons in water, arranged in rows according to the dendron type.

**Figure 2:**
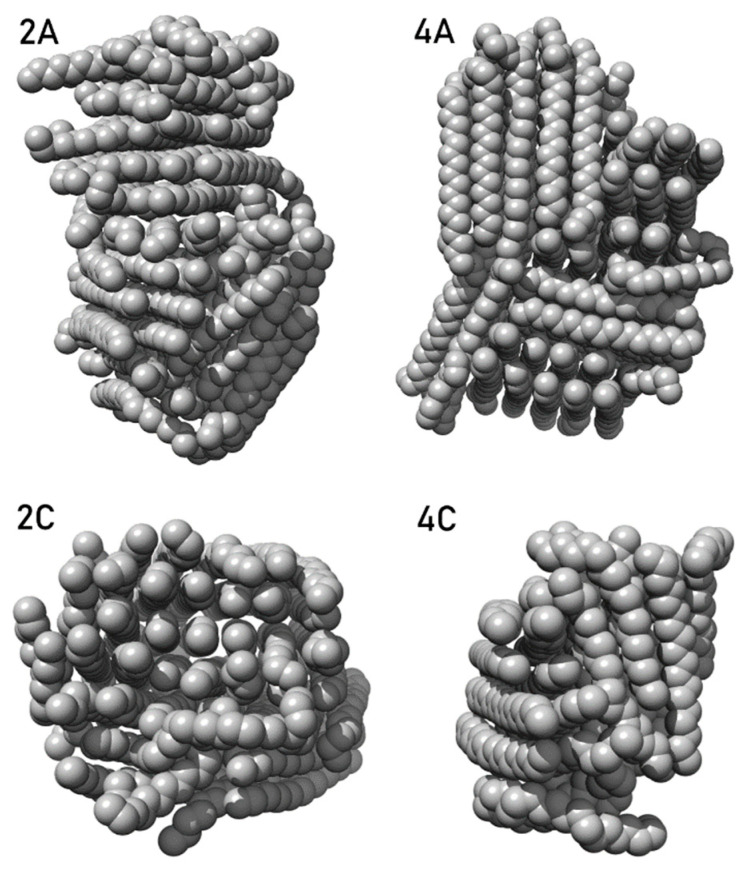
Hydrophobic cores of typical dendron clusters of **L2**–**12** (**left**) and **L2**–**15b** (**right**) depicted in Figure 1 (Lines 2 and 4). The labeling refers to structures in Figure 1. Only carbon atoms of aliphatic chains are shown for better clarity.

Dendrons **L1**–**9** and **L2**–**12** both have one hydrophilic branch and two hydrophobic tails. The size of their clusters obtained from simulations agrees quite well with the experimental values for the highest concentration, so it can be assumed that the simulations provide here a realistic description of micellar clusters studied experimentally at 100 μM. In the case of dendron **L1**–**9**, mostly spherical micellar structures were formed, together with some elongated clusters. Two typical representatives of **L1**–**9** clusters with 7.0 nm and 12.2 nm diameters are shown in Line 1. Dendron **L2**–**12** also created spherical (size approximately 6.6 nm) and smaller “barrel-shaped” clusters (larger size 5.5 nm) (Line 2). As these two dendrons differ only in the length of the aliphatic chains, we can well observe the effect of this parameter on the cluster structure. In the case of dendron **L2**–**12**, we can observe a very good parallel arrangement of aliphatic tails. Due to their considerable length (18 carbons), their mutual interactions are dominant for the arrangement of individual dendrons in the cluster. The internal structure of larger **L2**–**12** clusters (Figure 1 and Figure 2A,B) is composed of several differently oriented aliphatic domains. The hydrophobic core of smaller **L2**–**12** clusters is typically composed of only one domain, and, thus, such clusters have the character of an “amphiphilic bilayer” (Figure 1 and Figure 2C,D; both hydrophobic cores are also shown in detail in Figure 2). Therefore, hydrophobic aliphatic chains can occupy a relatively large part of the **L2**–**12** cluster surface, which implies less even distribution of the electric charge on the micellar surface compared to **L1**–**9** clusters and may cause the observed significant differences in zeta potential values of **L1**–**9** and **L2**–**12** samples.

A very similar effect of the length of aliphatic chains on the internal structure of micellar clusters can be observed also in the case of dendrons **L2**–**15a** and **L2**–**15b** having one hydrophilic branch and one hydrophobic tail (Figure 1, Lines 3 and 4). However, in comparison with dendron **L2**–**12**, the aliphatic domains in **L2**–**15b** clusters sample the space of all spatial orientations more evenly, and, thus, the hydrophilic head groups are also more evenly distributed on the surface. In addition, even smaller clusters of **L2**–**15b** consist of several differently oriented domains, in contrast to size-comparable “barrel-shaped” **L2**–**12** clusters. The simulated **L2**–**15a** clusters are predominantly spherical, but there are also some non-spherical “protruding” shapes (Line 3). In the case of the **L2**–**15b** dendron, only spherical micellar structures were created by simulation (Line 4). In the larger ones (size approximately 9.2 nm), the hydrophilic dendron heads are arranged in bundles, corresponding to individual aliphatic domains. This creates deeper hydrophobic “pockets” on the surface of such clusters, in which some smaller molecules of a hydrophobic nature (e.g., some lipophilic drugs) could bind well. In contrast to **L1**–**9** and **L2**–**12** clusters, the simulated **L2**–**15a** and **L2**–**15b** clusters are significantly smaller than experimentally observed particles, and, as such, they represent only building blocks from which resulting aggregates are formed. Regarding the charged dendron head groups, we can assume elongated shapes of these larger structures, created by “concatenation” of the smaller primary micellar structures.

For dendrons **L2**–**19a** and **L2**–**19b**, bearing two hydrophilic branches and only one hydrophobic tail, the simulation created a mix of spherical and irregular micellar clusters with sizes of about 4–5 nm (**L2**–**19a**, Line 5) and 5–8 nm (**L2**–**19b**, Line 6) with an even distribution of cationic groups on the surface of the clusters. The effect of aliphatic chain length in smaller clusters is no longer substantial, and the arrangement of dendrons in such clusters is determined mainly by the steric requirements of bulky hydrophilic wedges. However, in the case of dendron **L2**–**19b** with a longer aliphatic tail, larger clusters (quite similar to larger **L2**–**15b** clusters but less symmetrical) were also formed, again showing a fairly clear domain arrangement of aliphatic chains. While, in the case of **L2**–**19b**, the size of simulated micellar structures is comparable with experimental values, simulation of dendron **L2**–**19a** rather provided information about the building blocks of larger final aggregates, as apparent from DLS data.

## 3. Materials and Methods

### 3.1. General Remarks

All commercially available chemicals were used without further purification. Chemicals were purchased from the following distributors: dimethyl 5-hydroxybenzene-1,3-dicarboxylate (TCI Chemicals), methyl 4-hydroxybenzoate, 2-mercaptoethanol, 2-dimethylaminoethanthiol hydrochloride, 2,2′-(Ethylenedioxy)bis(ethylamine), 1,1′-Carbonyldiimidazole (Merck), methyl 3,5-dihydroxybenzoate, dodecan-1-amine, octadecan-1-amine, 2,2-Dimethoxy-2-phenylacetophenone, D-biotine (Fluorochem), 3-mercaptopropan-1,2-diol, methyl 2-mercaptoacetate (Acros Organics), Palladium on active carbon (Apollo Scientific), TBTU tetrafluoroborate (Carbosynth), cyanine-5 NHS ester, DBCO NHS ester, and Biotin-PEG3-azide (Lumiprobe). All other chemicals were from laboratory stock. Ethan-1,2-diamine was p0ur0ified by distillation at atmospheric pressure under argon. “Distilled THF” was purified by distillation from sodium under argon but was not kept dry. Experiments using UV light were carried out in thin-wall vials and irradiated by using a 400 W mercury lamp through PYREX filter, if not stated otherwise. Experiments under inert atmosphere were performed by using standard septum technique. TLC was carried out with Sigma-Aldrich TLC Silica gel 60 F254, and spots were detected by UV lamp 254 nm or visualized with KMnO_4_ solution (H_2_O/K_2_CO_3_/NaOH). Column chromatography was performed with silica gel 60 (70–230 mesh, Material Harvest). Nanofiltration was carried out by using solvent-resistant stirred cell (Millipore) equipped with 1 kDa MWCO regenerated cellulose ultrafiltration discs Ultracel^®^ (Millipore, Merck KGaA, Darmstadt, Germany) and PTFE encapsulated O-rings (ERIKS), with nitrogen as a driving gas. Separated mixture was quantitatively transferred into the cell, diluted with eluent to a total volume of 20–60 mL and passed through the membrane under pressure until approximately 1/20 of the initial volume. The cycle was repeated until sufficient purity was reached. Removal of solvents was performed on a rotary evaporator at maximum 50 °C, if not stated otherwise.

NMR spectra were measured on Bruker Avance 400 (^1^H at 400.1 MHz; ^13^C at 100.6 MHz; ^19^F at 376.4 MHz, ^29^Si {^1^H} (inept technique) at 79.5 MHz) at 25 °C. ^1^H and ^13^C NMR signals of prepared compounds were assigned to corresponding atoms utilizing HSQC, COSY, and HMBS 2D NMR correlation spectra. ^1^H and ^13^C chemical shifts (δ/ppm) are given relative to solvent signals (δ_H_/δ_C_: DMSO-*d*_6_ 2.50/39.52, CDCl_3_ 7.26/77.16, MeOH-*d*_4_ 3.31/49.00, THF-*d*_8_ 3.58/137.86); ^29^Si spectra were referenced to external standard hexamethyldisilane (δ/ppm; −19.87 ppm). The ^19^F NMR spectra were referenced to external standard hexafluorobenzene (δ/ppm; −163.86). CDCl_3_ and DMSO-*d*_6_ were dried over molecular sieves. THF-*d*_8_ ad MeOH-*d*_4_ were used as received. HRMS spectra were measured on a MicroTOF III spectrometer (Bruker) in the range of m/z 80–3000 Da. For ionization, APCI in positive mode or ESI in positive or negative mode was used with nitrogen as a nebulizer and dry gas. For calibration of accurate masses, an ESI–APCI Low Concentration Tuning Mix (Agilent) was used.

### 3.2. Dynamic Light Scattering (DLS)

The size of nanoparticles was measured by using the dynamic light scattering (DLS) method in a Zetasizer Nano-ZS instrument (ZEN3600, Malvern Instruments, Malvern, UK) [71,72]. The refraction factor was assumed to be 1.33, while the detection angle was 173° and the 633 nm wavelength of a He-Ne laser was used. Samples in water were placed in the glass cell (PCS8501, Malvern) and measured at 37 °C. The analysis of data was performed by using Malvern software.

The particle-charge measurements were conducted with a Zetasizer Nano-ZS, using an electrophoretic light scattering (ELS) measurement technique. The electrophoretic mobility of particles was measured in an applied electric field, using Malvern capillary plastic cells (DTS1061) with a gold-plated copper electrode. Samples were prepared and measured at 37 °C in water. The Zeta potential values were calculated by Malvern software from the Helmholtz–Smoluchowski equation [48]. The size of amphiphilic dendrons was measured in the concentration range from 0.1 to 100 µM, whereas Zeta potential value was measured only for 100 µM. All measurements were performed in triplicate to ensure consistency.

### 3.3. Computational Study

Three-dimensional computer models of dendron structures were created by using dendrimer builder, as implemented in the Materials Studio software package from BIOVIA, San Diego, CA, USA (formerly Accelrys). Generalized amber force field (GAFF) [48] was used for parameterization of all dendrons. The RESP technique [44] was used to calculate partial charges of dendrons. The pmemd.cuda module [55] from Amber18 package [45] was used for molecular dynamics simulations (explicit water, NPT, P = 1 bar, T = 295 K, length of simulation 400 ns). Please see the Supplementary Materials and also other relevant citations [45,56] for further simulation details.

## 4. Conclusions

The presented versatile synthetic protocol allows for straightforward access to libraries of amphiphilic dendrons with finely tunable properties of both the hydrophilic and hydrophobic domains by the choice of the aromatic core, the number and type of polar dendritic wedges and the number and length of aliphatic chains. The incorporation of a linker with a reactive end group enables the introduction of additional functionality of choice to the dendrons. DLS experiments at different concentrations confirmed that fine structure changes significantly affected the self-assembly behavior of dendrons in water. The balance between the hydrophilic and hydrophobic part influenced not only the size of the formed nanoparticles, but also its concentration dependence. Across different dendron types, all possible modes were observed. In the studied concentration range, both the increase and decrease of the particle size with concentration, along with concentration-independent behavior, were found. A detailed computational study at the molecular level showed how the structure of individual dendrons determines the structure of micellar clusters. The size of the simulated clusters, in some cases, agrees well with the values obtained from DLS for high-concentration samples.

## Figures and Tables

**Table 1:** Changes in hydrodynamic diameter of amphiphilic dendrons solution and Zeta potential values.

Amphiphilic Structure	Size (nm)							Zeta Potential ^1^ (mV)
	0.1 (µM)	0.5 (µM)	1 (µM)	5 (µM)	10 (µM)	50 (µM)	100 (µM)	
**L1**–**9**	69 ± 13	55 ± 5	52 ± 4	47 ± 18	28 ± 2	8 ± 1	8 ± 1	39.3 ± 3.0
**L2**–**12**	124 ± 2	76 ± 34	25 ± 19	13 ± 2	36 ± 2	24 ± 18	7 ± 1	71.5 ± 3.5
**L2**–**15a**	78 ± 27	75 ± 5	104 ± 1	68 ± 8	73 ± 4	60 ± 7	70 ± 1	62.7 ± 7.0
**L2**–**15b**	20 ± 4	20 ± 8	20 ± 6	25 ± 4	23 ± 3	25 ± 4	28 ± 5	68.5 ± 0.5
**L2**–**19a**	17 ± 4	18 ± 6	31 ± 47	36 ± 29	67 ± 37	79 ± 9	63 ± 11	71.7 ± 1.6
**L2**–**19b**	6 ± 7	3 ± 1	7 ± 3	7 ± 4	6 ± 2	7 ± 2	7 ± 1	52.8 ± 8.6
**L1**–**8**	6 ± 2	7 ± 1	5 ± 3	7 ± 1	7 ± 4	7 ± 2	6 ± 2	−27.2 ± 3.9

^1^ Zeta potential was measured at 100 mM concentration.

## Data Availability

All data presented here are available on request to the corresponding author.

## Supplementary Materials

The following supporting information can be downloaded at https://www.mdpi.com/article/10.3390/ijms23042114/s1.
