# Supplementary material for: Adaptive Synthesis of Functional Amphiphilic Dendrons as a Novel Approach to Artificial Supramolecular Objects
Source: Int J Mol Sci. 2022 Feb 14;23(4):2114. doi: 10.3390/ijms23042114 (PMC8877797; doi:10.3390/ijms23042114)
Supplement: Supplementary file 1 [file ijms-23-02114-s001.zip › ijms-1576897-supplementary.pdf]

International Journal of *Molecular Sciences*

MDPI

Supplementary Materials

Adaptive Synthesis of Functional Amphiphilic Dendrons as a Novel Approach to Artificial Supramolecular Objects

Antonín Edr[1,2], Dominika Wrobel[2], Alena Krupková[1,2], Lucie Červenková Šťastná[1,2], Petra Cuřínová[1,2], Aleš Novák[2], Jan Malý[3], Jitka Kalasová[3], Jan Malý[2], Marek Malý[2,*], Tomáš Strašák[1,2,*]

[1] Institute of Chemical Process Fundamentals of the CAS, v.v.i., Rozvojová 135, 165 02 Prague, Czech Republic; edr@icpf.cas.cz (A.E.); krupkova@icpf.cas.cz (A.K.); stastna@icpf.cas.cz (L.C.S.); curinova@icpf.cas.cz (P.C.)

[2] Faculty of Science, J.E. Purkyně University in Ústí nad Labem, Pasteurova 15, 400 96 Ústí nad Labem, Czech Republic; dominika_wrobel@o2.pl (D.W.); alesnovak.ul@gmail.com (A.N.); malyjalga@seznam.cz (J.M.)

[3] Faculty of Chemical Technology, University of Chemistry and Technology Prague, Technická 5, 16828 Prague 6, Czech Republic; jan.maly.33@gmail.com (J.M.); Jitka.Kalasova@vscht.cz (J.K.)

[*] Correspondence: strasak@icpf.cas.cz (T.S.); marek.maly@ujep.cz (M.M.)

Table of Contents:

1. Synthesis and characterization of compounds **1 - L3-31** 2
2. Computer modeling 17
3. NMR spectra of the prepared compounds 20
4. Mass spectrum of compound **L3-31** 65
References 65

1. Synthesis and characterization of compounds

Didodecyl 5-hydroxybenzene-1,3-dicarbamide 1: Dimethyl 5-hydroxybenzene-1,3-dicarboxylate (2.0 g, 9.5 mmol) and dodecane-1-amine (10.6 g, 57 mmol) were mixed under argon atmosphere and stirred at 100 °C for 18 h. After cooling to RT, the mixture was three times filtered through short columns of silicagel (eluent THF) - during the first filtration, the mixture was dispersed is Et_2O, applied on the column and firstly washed by Et_2O (400 mL) before applying THF. After the last filtration, solvent was removed obtaining product **1** (4.9 g, 98 %, beige amorphous substance). ^{1}H NMR (400 MHz, dmso-d_6, H-H COSY): δ 0.79 – 0.89 (m, 6H, *Me*), 1.18 – 1.31 (m, 36H, CH_2), 1.49 (p, *J* = 7.0 Hz, 4H, NCH_2CH_2), 3.21 (td, *J* = 7.0, 5.6 Hz, 4H, NCH_2), 7.31 (d, *J* = 1.5 Hz, 2H, CH_{Ph}), 7.69 (t, *J* = 1.5 Hz, 1H, CH_{Ph}), 8.40 (t, *J* = 5.6 Hz, 2H, N*H*), 9.86 (s, 1H, O*H*). ^{13}C {^{1}H} NMR (101 MHz, dmso-d_6, HSQC): δ 14.0 (*Me*), 22.1, 26.5, 28.7, 28.8, 29.00 (5 × CH_2), 29.01 (2CH_2), 29.03, 29.1, 31.3 (3 × CH_2), overlapped with DMSO from HSQC 38.9 (NCH_2), 116.5 (2CH_{Ph}), 116.6 (CH_{Ph}), 136.3 (C_q), 157.2 (OC_q), 165.8 (CO). HRMS (APCI+): Calcd. for $[C_{32}H_{57}N_2O_3]^+$ 517.4363, found $[M+H]^+$ 517.4359.

Compound 2: Didodecyl 5-hydroxybenzene-1,3-dicarbamide (**1**, 5.00 g, 9.67 mmol) and calcinated K_2CO_3 (1.47 g, 10.6 mmol) were dissolved in dry acetonitrile (150 mL) and stirred at reflux for 30 min. Afterwards, triallyl(3-iodopropyl)silane (2.94 g, 9.19 mmol) was added under argon atmosphere and the reaction mixture stirred at reflux for 24 h. Then, solvent was removed on rotary evaporator, EtOAc (300 mL) was added and the mixture was washed (150 mL H_2O, 3 × 100 mL 10% sol. K_2CO_3, again 100 mL H_2O and finally 150 mL brine) and dried by anhydrous $MgSO_4$. Solvent was removed and the residue filtered through a short column of silicagel (eluents: 1. 400 mL petrolether/EtOAc 20:1, 2. 400 mL petrolether/EtOAc 4:1). The desired product **2** was obtained by solvents removal from the second fraction (5.11 g, 78 %, orange viscous liquid). ^{1}H NMR (400 MHz, chloroform-*d*, H-H COSY): δ 0.69 – 0.73 (m, 2H, $SiCH_2CH_2$), 0.86 – 0.89 (m, 6H, *Me*), 1.23 – 1.42 (m, 36H, CH_2), 1.60 (p, *J* = 7.2 Hz, 4H, NCH_2CH_2), 1.63 (dt, *J* = 8.1, 1.2 Hz, 6H,$SiCH_2CH$), 1.78 – 1.89 (m, 2H, $SiCH_2CH_2$), 3.43 (td, *J* = 7.2, 5.7 Hz, 4H, NCH_2), 3.98 (t, *J* = 6.6 Hz, 2H, OCH_2), 4.97 – 4.83 (m, 6H, $CHCH_2$), 5.79 (ddt, *J* = 16.9, 10.1, 8.1 Hz, 3H, CH_2CH), 6.25 (t, *J* = 5.7 Hz, 2H, N*H*), 7.14 (d, *J* = 1.5 Hz, 2H, CH_{Ph}), 7.67 (t, *J* = 1.5 Hz, 1H, CH_{Ph}). ^{13}C {^{1}H} NMR (101 MHz, chloroform-*d*, HSQC, HMBC): δ 7.5 ($SiCH_2$), 14.1 (*Me*), 19.5 ($SiCH_2CH$), 22.7 (CH_2), 23.3 (OCH_2CH_2), 27.0, 29.3, 29.4, 29.55 (4 × CH_2), 29.61 (2CH_2), 29.63, 29.7, 31.9 (3 × CH_2), 40.3 (NCH_2), 71.0 (OCH_2), 113.9 ($CHCH_2$), 115.8 (2CH_{Ph}), 116.9 (CH_{Ph}), 134.1 ($CHCH_2$), 136.5 (C_q), 159.4 (OC_q), 166.5 (CO). ^{29}Si {^{1}H} NMR (79 MHz, chloroform-*d*): δ –0.13. HRMS (APCI+): Calcd. for $[C_{44}H_{77}N_2O_3Si]^+$ 709.5697, found $[M+H]^+$ 709.5696.

Dendron L1-3. Triallylic dendritic compound **2** (100 mg, 0.141 mmol) and 2-mercaptoethanol (0.16 g, 0.85 mmol, 0.06 mL) were dissolved in distilled THF (4 mL) in 10mL vial and deoxygenated. The reaction mixture was stirred under argon atmosphere and irradiated for 8 h. Afterwards, solvent was removed on rotary evaporator and the excess of 2-mercaptoethanol was removed by heating to 90 °C under reduced pressure (500 Pa) obtaining dendron **L1-3** (132 mg, 98 %, colourless viscous liquid). ^{1}H NMR (400 MHz, dmso-d_6, H-H COSY): δ 0.61 – 0.67 (m, 8H, $SiCH_2$), 0.83 (t, *J* = 6.6 Hz, 6H, *Me*), 1.23 – 1.28 (m, 36H, CH_2), 1.48 – 1.56 (m, 10H, $CH_2CH_2CH_2S$, CH_2CH_2NH), 1.71 – 1.75 (m, 2H, OCH_2CH_2), 2.53 (t, *J* = 7.2 Hz, 12H, CH_2S), 3.24 (td, *J* = 7.2, 5.6 Hz, 4H, $NHCH_2$), 3.50 (td, *J* = 7.0, 5.6 Hz, 6H, $OHCH_2$), 4.01 (t, *J* = 6.4 Hz, 2H, OCH_2), 4.74 (t, *J* = 5.6 Hz, 3H, O*H*), 7.47 (d, *J* = 1.5 Hz, 2H, CH_{Ph}), 7.86 (t, *J* = 1.5 Hz, 1H, CH_{Ph}), 8.47 (t, *J* = 5.6 Hz, 2H, N*H*). ^{13}C {^{1}H} NMR (101 MHz, dmso-d_6, HSQC, HMBC): δ 7.9 (CH_2Si), 11.2 (CH_2Si), 14.0 (*Me*), 22.1 (CH_2), 23.3 (OCH_2CH_2), 24.0 ($CH_2CH_2CH_2S$), 26.5, 28.7, 28.8, 28.9 (4 × CH_2), 29.00 (2CH_2), 29.02, 29.1, 31.3 (3 × CH_2), 33.9 (SCH_2CH_2OH), 35.2 ($CH_2CH_2CH_2S$), overlapped with DMSO from HSQC 39.2 (CH_2NH), 61.0 (CH_2OH), 70.5 (OCH_2), 115.4 (2CH_{Ph}), 118.5 (CH_{Ph}), 136.2 (C_q), 158.4 (C_qO), 165.4 (CO). ^{29}Si {^{1}H} NMR (79 MHz, dmso-d_6): δ 3.97. HRMS (ESI+): Calcd. for $[C_{50}H_{94}N_2O_6S_3SiNa]^+$ 965.5935, found $[M+Na]^+$ 965.5942.

Dendron L1-4. Triallylic dendritic compound **2** (300 mg, 0.423 mmol), 3-mercaptopropane-1,2-diol (0.34 g, 3.2 mmol, 0.27 mL) and DMPA (33 mg, 0.13 mmol) were dissolved in distilled THF (8 mL) in 10mL vial and deoxygenated. The reaction mixture was stirred under inert argon atmosphere and irradiated for 10 h. Afterwards, solvent was removed on rotary evaporator and the acquired mixture separated by column chromatography (eluent DCM/MeOH 92:8) obtaining dendron **L1-4** (343 mg, 78 %, R_f = 0.35, yellowish viscous liquid). ^{1}H NMR (400 MHz, dmso-d_6, H-H COSY): δ 0.61 – 0.66 (m, 8H, SiCH_2), 0.83 (t, J = 6.6 Hz, 6H, *Me*), 1.25 – 1.28 (m, 36H, CH_2), 1.48 – 1.54 (m, 10H, CH_2C$H_2$$CH_2S, CH_2$$CH_2$NH), 1.69 – 1.77 (m, 2H, OCH_2CH_2), 2.42 (dd, J = 13.3, 6.7 Hz, 3H, SCH*H*CH), 2.52 (t, J = 7.1 Hz, 6H, CH_2CH_2S), 2.59 (dd, J = 13.3, 5.3 Hz, 3H, SCH*H*CH), 3.24 (td, J = 7.3, 5.7 Hz, 4H, NHCH_2), 3.34 (d, J = 5.3 Hz, 6H, OHCH_2), 3.54 (qd, J = 6.7, 5.3 Hz, 3H, C*H*CH_2), 4.00 (t, J = 6.5 Hz, 2H, OCH_2), 7.48 (d, J = 1.5 Hz, 2H, CH_{Ph}), 7.89 (t, J = 1.5 Hz, 1H, CH_{Ph}), 8.51 (t, J = 5.7 Hz, 2H, N*H*). ^{13}C {^{1}H} NMR (101 MHz, dmso-d_6, HSQC, HMBC): δ 7.9 (CH_2Si), 11.3 (CH_2Si), 14.0 (*Me*), 22.1 (CH_2), 23.3 (O$CH_2$$CH_2$), 23.9 ($CH_2$$CH_2$S), 26.5, 28.7, 28.8, 28.99 (4 × CH_2), 29.01 (2CH_2), 29.03, 29.1, 31.3 (3 × CH_2), 35.2 (SCH_2CH), 35.9 ($CH_2$$CH_2$S), overlapped with DMSO from HSQC 39.3 (CH_2NH), 64.5 (CH_2OH), 71.5 (HOCH), 115.4 (2CH_{Ph}), 118.5 (CH_{Ph}), 136.2 (C_q), 158.5 (C_qO), 165.4 (CO). ^{29}Si {^{1}H} NMR (79 MHz, dmso-d_6): δ 3.96. HRMS (ESI+): Calcd. for $[C_{53}H_{100}N_2O_9S_3SiNa]^+$ 1055.6252, found $[M+Na]^+$ 1055.6239.

Dendron L1-5. Triallylic dendritic compound **2** (500 mg, 0.705 mmol), methyl 2-mercaptoacetate (0.67 g, 6.4 mmol, 0.57 mL) and DMPA (18 mg, 0.071 mmol) were dissolved in distilled THF (7 mL) in 10mL vial and deoxygenated. The reaction mixture was stirred under argon atmosphere and irradiated for 3 h. Afterwards, solvent was removed on rotary evaporator, the acquired mixture dissolved in Et_2O (200 mL) and washed (2 × 50 mL 1% sol. NaOH, 2 × 50 mL H_2O and 50 mL brine). Organic layer was dried by anhydrous $MgSO_4$ and solvent removed obtaining dendron **L1-5** (709 mg, 98 %, yellowish opaque viscous liquid). ^{1}H NMR (400 MHz, dmso-d_6, H-H COSY): δ 0.61 – 0.66 (m, 8H, SiCH_2), 0.83 (t, J = 6.6 Hz, 6H, *Me*), 1.22 – 1.28 (m, 36H, CH_2), 1.49 – 1.57 (m, 10H, C$H_2$$CH_2S, CH_2$$CH_2$NH), 1.71 – 1.73 (m, 2H, OCH_2CH_2), 2.58 (t, J = 7.2 Hz, 6H, CH_2S), 3.24 (td, J = 7.2, 5.6 Hz, 4H, NHCH_2), 3.30 (s, 6H, CH_2COOMe), 3.62 (s, 9H, CH_2COO*Me*), 4.01 (t, J = 6.5 Hz, 2H, OCH_2), 7.48 (d, J = 1.4 Hz, 2H, CH_{Ph}), 7.87 (t, J = 1.4 Hz, 1H, CH_{Ph}), 8.47 (t, J = 5.6 Hz, 2H, N*H*). ^{13}C {^{1}H} NMR (101 MHz, dmso-d_6, HSQC, HMBC): δ 7.8 (CH_2Si), 11.2 (CH_2Si), 13.9 (*Me*), 22.1 (CH_2), 23.2 (O$CH_2$$CH_2$), 23.3 ($CH_2$$CH_2$S), 26.5, 28.7, 28.8, 29.00, 29.01 (5 × CH_2), 29.03 (2CH_2), 29.01, 31.3 (2 × CH_2), 32.6 (CH_2COOMe), 35.6 ($CH_2$$CH_2$S), overlapped with DMSO from HSQC 39.2 (CH_2NH), 51.9 (COO*Me*), 70.5 (OCH_2), 115.4 (2CH_{Ph}), 118.4 (CH_{Ph}), 136.2 (C_q), 158.4 (C_qO), 165.4 (CONH), 170.7 (COO). ^{29}Si {^{1}H} NMR (79 MHz, dmso-d_6): δ 4.06. HRMS (ESI+): Calcd. for $[C_{53}H_{94}N_2O_9S_3SiNa]^+$ 1049.5783, found $[M+Na]^+$ 1049.5785.

Dendron L1-6. Distilled ethan-1,2-diamine (1,8 g, 30 mmol, 2.0 mL) was added to dendron **L1-5** (100 mg, 0.097 mmol) and the reaction mixture was deoxygenized. The reaction mixture was stirred under argon atmosphere at 80 °C for 16 h. Afterwards, the excess of the diamine was evaporated at 60 °C under reduced pressure (200 Pa) under argon atmosphere obtaining dendron **L1-6** (107 mg, 98 %, yellow viscous substance). ^{1}H NMR (400 MHz, dmso-d_6, H-H COSY): δ 0.59 – 0.67 (m, 8H, SiCH_2), 0.84 (t, J = 6.8 Hz, 6H, *Me*), 1.23 – 1.28 (m, 36H, CH_2), 1.49 – 1.56 (m, 10H, C$H_2$$CH_2S, CH_2CH_2$$CH_2$NH), 1.72 (p, J = 6.5 Hz, 2H, OCH_2CH_2), 2.56 (t, J = 7.2 Hz, 6H, CH_2CH_2S), 2.59 (t, J = 6.4 Hz, 4H, NH_2CH_2), 3.07 (s, 6H, SCH_2CO), 3.07 (td, J = 6.4, 5.6 Hz, 6H, $NH_2$$CH_2CH_2$), 3.24 (td, J = 6.8, 5.6 Hz, 4H, NHCH_2), 4.00 (t, J = 6.5 Hz, 2H, OCH_2), 7.48 (d, J = 1.5 Hz, 2H, CH_{Ph}), 7.87 (t, J = 1.5 Hz, 1H, CH_{Ph}), 8.00 (t, J = 5.6 Hz, 3H, N*H*), 8.52 (t, J = 5.6 Hz, 2H, N*H*). ^{13}C {^{1}H} NMR (101 MHz, dmso-d_6, HSQC, HMBC): δ 7.8 (CH_2Si), 11.3 (CH_2Si), 14.0 (*Me*), 22.1 (CH_2), 23.3 (O$CH_2$$CH_2$), 23.5 ($CH_2$$CH_2$S), 26.5, 28.7, 28.8, 28.98, 29.00 (5 × CH_2), 29.02 (2CH_2), 29.1, 31.3 (2 × CH_2), 34.6 (SCH_2CO), 35.6 ($CH_2$$CH_2$S), overlapped with DMSO from HSQC 39.3 (CH_2NH), 40.9 ($NH_2$$CH_2$), 41.7 ($NH_2$$CH_2$$CH_2$), 70.6 (O$CH_2$), 115.4 (2$CH_{Ph}$), 118.4 ($CH_{Ph}$), 136.2 ($C_q$), 158.4 ($C_q$O), 165.4 (2CO), 169.2 (3CO). ^{29}Si {^{1}H} NMR (79 MHz, dmso-d_6): δ 4.00. HRMS (ESI+): Calcd. for $[C_{56}H_{107}N_8O_6S_3Si]^+$ 1111.7239, found $[M+H]^+$ 1111.7238.

Dendron L1-7. Triallylic dendritic compound **2** (500 mg, 0.705 mmol), 2-mercaptoacetic acid (0.58 g, 6.4 mmol, 0.44 mL) and DMPA (18 mg, 0.071 mmol) were dissolved in distilled THF (7 mL) in 10mL vial and deoxygenated. The reaction mixture was stirred under argon atmosphere and irradiated for 3 h. Afterwards, solvent was removed on rotary evaporator, the acquired mixture dissolved in DCM (200 mL) and washed (3 × 100 mL H_2O). Organic layer was dried by anhydrous $MgSO_4$ and solvent removed and obtained pure dendron **L1-7** lyophilised (683 mg, 98 %, yellowish viscous substance). ^{1}H NMR (400 MHz, dmso-d_6, H-H COSY): δ 0.61 – 0.65 (m, 8H, SiCH_2), 0.84 (t, *J* = 6.5 Hz, 6H, *Me*), 1.22 – 1.28 (m, 36H, CH_2), 1.49 – 1.58 (m, 10H, CH_2CH$_2$S, CH_2CH$_2$NH), 1.70 – 1.76 (m, 2H, OCH$_2$CH_2), 2.59 (t, *J* = 7.2 Hz, 6H, CH$_2$CH_2S), 3.19 (s, 6H, CH_2COOH), 3.24 (td, *J* = 7.2, 5.6 Hz, 4H, NHCH_2), 4.00 (t, *J* = 6.5 Hz, 2H, OCH_2), 7.48 (s, 2H, CH_{Ph}), 7.87 (s, 1H, CH_{Ph}), 8.47 (t, *J* = 5.6 Hz, 2H, N*H*). ^{13}C {^{1}H} NMR (101 MHz, dmso-d_6, HSQC, HMBC): δ 7.8 (CH$_2$Si), 11.2 (CH$_2$Si), 14.0 (*Me*), 22.1 (CH$_2$), 23.27 (CH$_2$CH$_2$S), 23.30 (OCH$_2$CH$_2$), 26.5, 28.7, 28.8, 29.00, 29.01 (5 × CH$_2$), 29.03 (2CH$_2$), 29.1, 31.3 (2 × CH$_2$), 33.2 (CH$_2$COOH), 35.5 (CH$_2$CH$_2$S), overlapped with DMSO from HSQC 39.3 (CH$_2$NH), 70.5 (OCH$_2$), 115.4 (2CH$_{Ph}$), 118.5 (CH$_{Ph}$), 136.2 (C$_q$), 158.4 (C$_q$O), 165.4 (CONH), 171.7 (COOH). ^{29}Si {^{1}H} NMR (79 MHz, dmso-d_6): δ 4.08. HRMS (ESI+): Calcd. for $[C_{50}H_{88}N_2O_9S_3SiNa]^+$ 1007.5313, found $[M+Na]^+$ 1007.5310.

Dendron L1-8. Dendron **L1-7** (660 mg, 0.67 mmol) was dispersed in H_2O (10 mL) and NaOH (134 mg, 3.35 mmol) in H_2O (15 mL) was slowly added. To the reaction mixture, MeOH (1 mL) was added and the reaction mixture was stirred at RT for 10 min. The mixture was then separated by nanofiltration in a mixture of methanol and water 1:20 obtaining dendron **L1-8** (682 mg, 98 %, yellowish foam). ^{1}H NMR (400 MHz, MeOH-d_4, H-H COSY): δ 0.69 – 0.77 (m, 8H, SiCH_2), 0.88 (t, *J* = 6.5 Hz, 6H, *Me*), 1.28 – 1.41 (m, 36H, CH_2), 1.59 – 1.67 (m, 10H, CH_2CH$_2$S, CH_2CH$_2$NH), 1.80 – 1.87 (m, 2H, OCH$_2$CH_2), 2.60 (t, *J* = 7.2 Hz, 6H, CH$_2$CH_2S), 3.13 (s, 6H, CH_2COONa), 3.37 (t, *J* = 7.2 Hz, 4H, NHCH_2), 4.06 (t, *J* = 6.3 Hz, 2H, OCH_2), 7.52 (d, *J* = 1.6 Hz, 2H, CH_{Ph}), 7.83 (t, *J* = 1.6 Hz, 1H, CH_{Ph}). ^{13}C {^{1}H} NMR (101 MHz, MeOH-d_4, HSQC, HMBC): δ 9.2 (CH$_2$Si), 12.9 (CH$_2$Si), 14.5 (*Me*), 23.7 (CH$_2$), 24.8 (OCH$_2$CH$_2$), 25.0 (CH$_2$CH$_2$S), 28.1, 30.4, 30.46, 30.47, 30.69. 30.72, 30.76, 30.77, 33.1 (9 × CH$_2$), 37.3 (CH$_2$CH$_2$S), 38.1 (CH$_2$COONa), 41.2 (CH$_2$NH), 72.1 (OCH$_2$), 117.1 (2CH$_{Ph}$), 119.5 (CH$_{Ph}$), 137.2 (C$_q$), 160.7 (C$_q$O), 169.3 (CONH), 178.3 (COONa). ^{29}Si {^{1}H} NMR (79 MHz, dmso-d_6): δ 3.79. HRMS (ESI-): Calcd. for $[C_{50}H_{87}N_2O_9S_3Si]^-$ 983.5348, found $[M-3Na+2H]^-$ 983.5350.

Dendron L1-9. Triallylic dendritic compound **2** (500 mg, 0.705 mmol), 2-(dimethylamino)ethanthiol hydrochloride (379 mg, 2.54 mmol) and DMPA (18 mg, 0.071 mmol) were dissolved in a mixture of distilled THF (2 mL) and MeOH (4 mL) in 10mL vial and deoxygenated. The reaction mixture was stirred under argon atmosphere and irradiated for 4 h. Afterwards, solvent was removed on rotary evaporator and the acquired mixture separated by nanofiltration in a mixture of DCM and methanol 1:1 obtaining dendron **L1-9** (799 mg, 98 %, white foam). ^{1}H NMR (400 MHz, dmso-d_6, H-H COSY): δ 0.64 – 0.68 (m, 8H, SiCH_2), 0.83 (t, *J* = 6.6 Hz, 6H, *Me*), 1.21 – 1.28 (m, 36H, CH_2), 1.48 – 1.59 (m, 10H, CH$_2$CH_2CH$_2$S, CH_2CH$_2$NH), 1.69 – 1.77 (m, 2H, OCH$_2$CH_2), 2.58 (t, *J* = 7.1 Hz, 6H, CH$_2$CH$_2$CH_2S), 2.74 (s, 18H, NH*Me*$_2$), 2.84 – 2.88 (m, 6H, SCH_2CH$_2$NH), 3.19 – 3.24 (m, 10H, NHCH_2, CH_2NHMe$_2$), 3.33 (br s, 3H, N*H*Me$_2$), 4.03 (t, *J* = 6.5 Hz, 2H, OCH_2), 7.51 (s, 2H, CH_{Ph}), 8.06 (s, 1H, CH_{Ph}), 8.71 (t, *J* = 5.7 Hz, 2H, N*H*). ^{13}C {^{1}H} NMR (101 MHz, dmso-d_6, HSQC, HMBC): δ 7.8 (CH$_2$Si), 11.1 (CH$_2$Si), 13.9 (*Me*), 22.1 (CH$_2$), 23.3 (OCH$_2$CH$_2$), 23.7 (CH$_2$CH$_2$CH$_2$S), 24.5 (SCH$_2$CH$_2$NH), 26.5, 28.7, 28.8, 29.01 (4 × CH$_2$), 29.02 (3CH$_2$), 29.1, 31.3 (2 × CH$_2$), 34.5 (CH$_2$CH$_2$CH$_2$S), overlapped with DMSO from HSQC 39.0 (CH$_2$NH), 41.8 (NH*Me*$_2$), 55.8 (CH$_2$NHMe$_2$), 70.5 (OCH$_2$), 115.6 (2CH$_{Ph}$), 118.4 (CH$_{Ph}$), 136.0 (C$_q$), 158.5 (C$_q$O), 165.3 (CO). ^{29}Si {^{1}H} NMR (79 MHz, dmso-d_6): δ 4.06. HRMS (ESI+): Calcd. for $[C_{56}H_{110}N_5O_3S_3Si]^+$ 1024.7534, found $[M-3Cl-2H]^+$ 1024.7528.

Compound 10. Dimethyl 5-hydroxybenzene-1,3-dicarboxylate (1.0 g, 4.8 mmol) and octadecane-1-amine (7.7 g, 29 mmol) were mixed under argon atmosphere and stirred at 100 °C for 18 h. After cooling to RT, the mixture was three times filtered through short column of silicagel (eluent THF). During the first filtration, the mixture was dispersed is

Et_2O, applied on the column and firstly washed by Et_2O (200 mL) before applying THF. Before the second filtration, concentrated HCl (2.0 mL, 16 mmol) was slowly added to a solution of crude product in THF (50 mL). After the last filtration, solvent was removed obtaining product **10** (2.3 g, 70 %, white powder). ^1H NMR (400 MHz, THF-d_8, H-H COSY): δ 0.87 – 0.91 (m, 6H, *Me*), 1.29 – 1.36 (m, 60H, CH_2), 1.57 (p, *J* = 7.3 Hz, 4H, NCH_2CH_2), 3.33 (td, *J* = 7.3, 5.7 Hz, 4H, NCH_2), 7.34 (d, *J* = 1.5 Hz, 2H, CH_{Ph}), 7.66 (t, *J* = 5.7 Hz, 2H, N*H*), 7.69 (t, *J* = 1.5 Hz, 1H, CH_{Ph}), 8.66 (s, 1H, O*H*). ^{13}C {^1H} NMR (101 MHz, THF-d_8, HSQC, HMBC): δ 14.6 (*Me*), 23.7, 28.2, 30.5, 30.6, 30.78 (5 × CH_2), 30.82, 30.83 (2 × 4CH_2), 30.9, 31.0, 33.0 (3 × CH_2), 40.7 (NCH_2), 117.4 (CH_{Ph}), 117.5 (2CH_{Ph}), 138.0 (C_q), 158.7 (OC_q), 166.8 (*C*O). HRMS (APCI+): Calcd. for $[C_{44}H_{81}N_2O_3]^+$ 685.6242, found $[M+H]^+$ 685.6242.

Compound 11. Dioctadecyl 5-hydroxybenzene-1,3-dicarbamide (**10**, 1.30 g, 1.90 mmol) and calcinated K_2CO_3 (0.40 g, 2.9 mmol) were dissolved in dry DMF (30 mL) and stirred at 60 °C for 30 min. Then, triallyl(3-iodopropyl)silane (0.61 g, 1.9 mmol) was added under argon atmosphere and the reaction mixture stirred at 90 °C for 18 h. Afterwards, solvent was removed on rotary evaporator and the mixture filtered through a short column of silicagel (eluents: 1. 200 mL pentane/Et_2O 10:1, 2. 250 mL Et_2O/DCM 10:1). The desired product **11** was obtained by solvents removal from the second fraction (1.32 g, 79 %, brownish viscous liquid). ^1H NMR (400 MHz, THF-d_8, H-H COSY): δ 0.75 – 0.80 (m, 2H, SiCH_2CH$_2$), 0.87 – 0.91 (m, 6H, *Me*), 1.29 – 1.39 (m, 60 H, CH_2), 1.57 (p, *J* = 7.2 Hz, 4H, NCH_2CH_2), 1.66 (dt, *J* = 8.2, 1.3 Hz, 6H, SiCH_2CH), 1.83 – 1.89 (m, 2H, $SiCH_2CH_2$), 3.35 (td, *J* = 7.2, 5.6 Hz, 4H, NCH_2), 3.99 (t, *J* = 6.5 Hz, 2H, OCH_2), 4.83 – 4.93 (m, 6H, CHCH_2), 5.84 (ddt, *J* = 16.5, 10.1, 8.1 Hz, 3H, CH_2C*H*), 7.48 (d, *J* = 1.5 Hz, 2H, CH_{Ph}), 7.72 (t, *J* = 5.6 Hz, 2H, N*H*), 7.82 (t, *J* = 1.5 Hz, 1H, CH_{Ph}). ^{13}C {^1H} NMR (101 MHz, THF-d_8, HSQC, HMBC): δ 8.6 ($SiCH_2$), 14.6 (*Me*), 20.4 ($SiCH_2CH$), 23.7 (CH_2), 24.5 (OCH_2CH_2), 28.2, 30.5, 30.6, 30.77 (4 × CH_2), 30.83 (9CH_2), 31.0, 33.0 (2 CH_2), 40.8 (NCH_2), 71.6 (OCH_2), 114.2 ($CHCH_2$), 116.5 (2CH_{Ph}), 118.7 (CH_{Ph}), 135.4 ($CHCH_2$), 138.0 (C_q), 160.2 (OC_q), 166.6 (*C*O). ^{29}Si {^1H} NMR (79 MHz, THF-d_8): δ –0.29. HRMS (APCI+): Calcd. for $[C_{56}H_{101}N_2O_3Si]^+$ 877.7575, found $[M+H]^+$ 877.7504.

Dendron L2-12. Triallylic dendritic compound **11** (300 mg, 0.342 mmol), 2-(dimethylamino)ethanthiol hydrochloride (581 mg, 4.10 mmol) and DMPA (9 mg, 0.034 mmol) were dissolved in a mixture of distilled THF (5 mL) and MeOH (2.5 mL) in 10mL vial and deoxygenated. The reaction mixture was stirred under argon atmosphere and irradiated for 6 h. Afterwards, solvent was removed on rotary evaporator and the acquired mixture separated by nanofiltration in a mixture of DCM and methanol 10:1 obtaining dendron **L2-12** (400 mg, 90 %, white amorphous substance). ^1H NMR (400 MHz, dmso-d_6, H-H COSY): δ 0.64 – 0.68 (m, 8H, SiCH_2), 0.83 – 0.86 (m, 6H, *Me*), 1.22 – 1.28 (m, 60H, CH_2), 1.40 – 1.58 (m, 10H, $CH_2CH_2CH_2S$, CH_2CH_2NH), 1.68 – 1.75 (m, 2H, OCH_2CH_2), 2.58 (t, *J* = 7.1 Hz, 6H, $CH_2CH_2CH_2S$), 2.74 (s, 18H, NH*Me*$_2$), 2.81 – 2.85 (m, 6H, SCH_2CH_2NH), 3.19 – 3.26 (m, 10H, NHCH_2, CH_2NHMe_2), 4.02 (t, *J* = 6.5 Hz, 2H, OCH_2), 7.48 (d, *J* = 1.4 Hz, 2H, CH_{Ph}), 7.92 (t, *J* = 1.4 Hz, 1H, CH_{Ph}), 8.56 (t, *J* = 5.6 Hz, 2H, N*H*), 10.3 (br s, 3H, N^+H). ^{13}C {^1H} NMR (101 MHz, dmso-d_6, HSQC, HMBC): δ 7.5 (CH_2Si), 10.9 ($CHCH_2Si$), 13.8 (*Me*), 21.9 (CH_2), 23.1 (OCH_2CH_2), 23.5 ($CH_2CH_2CH_2S$), 24.5 (SCH_2CH_2NH), 26.3, 28.5, 28.6 (3 × CH_2), 28.77, 28.81 (2 × 2CH_2), 28.8 (5CH_2), 28.83 (2CH_2), 31.1 (CH_2), 34.3 ($CH_2CH_2CH_2S$), overlapped with DMSO from HSQC 39.2 (CH_2NH), 41.8 (NH*Me*$_2$), 55.6 (CH_2NHMe_2), 70.4 (OCH_2), 115.4 (2CH_{Ph}), 118.1 (CH_{Ph}), 136.0 (C_q), 158.3 (C_qO), 165.2(CO). ^{29}Si {^1H} NMR (79 MHz, dmso-d_6): δ 4.07. HRMS (ESI+): Calcd. for $[C_{68}H_{134}N_5O_3S_3Si]^+$ 1192.9412, found $[M-3Cl-2H]^+$ 1192.9380; calcd. for $[C_{68}H_{135}N_5O_3S_3Si]^{2+}$ 596.9742, found $[M-3Cl-1H]^{2+}$ 596.9771.

Compound 13a. Methyl 4-hydroxybenzoate (1.00 g, 6.57 mmol) and dodecane-1-amine (3.65 g, 19.7 mmol) were mixed under argon atmosphere and stirred at 100 °C for 100 h. After cooling to RT, the mixture was two times filtered through short columns of silicagel (eluents: 1. 150 mL Et_2O, 2. 200 mL THF) each time keeping the second fraction. After the second filtration, THF was removed obtaining intermedial salt. This salt (1.61 g, 3.28 mmol) was dissolved in THF (80 mL), treated with conc. HCl (0.40 mL, 4.7 mmol) and filtered through a short column of silicagel (eluent THF). Solvent was removed obtaining product **13a** (1.00 g, 50 %, beige amorphous substance). ^1H NMR (400 MHz, dmso-d_6,

H-H COSY): δ 0.83 – 0.87 (m, 3H, *Me*), 1.23 – 1.29 (m, 18H, C*H*$_2$), 1.48 (p, *J* = 7.0 Hz, 2H, NCH$_2$C*H*$_2$), 3.19 (td, *J* = 7.0, 5.6 Hz, 2H, NC*H*$_2$), 6.77, 7.69 (2 × d, *J* = 8.7 Hz, 2 × 2H, C*H*$_{Ph}$), 8.14 (t, *J* = 5.6 Hz, 1H, N*H*), 9.89 (s, 1H, O*H*). ^{13}C {^{1}H} NMR (101 MHz, dmso-*d*$_6$, HSQC, HMBC): δ 14.0 (*Me*), 22.1, 26.5, 28.7, 28.8, 29.00, 29.01, 29.04, 29.2, 30.4, 31.3 (10 × *C*H$_2$), overlapped with DMSO from HSQC 38.8 (NCH$_2$), 114.7 (2*C*H$_{Ph}$), 125.5 (*C*$_q$), 129.0 (2*C*H$_{Ph}$), 159.9 (O*C*$_q$), 165.7 (*C*O). HRMS (APCI+): Calcd. for [$C_{19}H_{32}NO_2$]$^+$ 306.2427, found [M+H]$^+$ 306.2433.

Compound 14a. N-dodecyl-4-hydroxybenzamide (**13a**, 500 mg, 1.64 mmol) and calcinated K_2CO_3 (249 mg, 1,80 mmol) were dissolved in dry acetonitrile (20 mL) under argon atmosphere and stirred at reflux for 20 min. Afterwards, triallyl(3-iodopropyl)silane (2.94 g, 9.19 mmol) was added and the reaction mixture stirred at reflux for 24 h. Then, solvent was removed on rotary evaporator, EtOAc (100 mL) was added and the mixture was washed (50 mL H_2O, 3 × 50 mL 10% sol. K_2CO_3, again 50 mL H_2O and finally 50 mL brine) and dried by anhydrous $MgSO_4$. Solvent was removed and the residue filtered through a short column of silicagel (eluent petrolether/EtOAc 5:1). Solvents were removed obtaining product **14a** (517 m g, 67 %, brown viscous liquid). ^{1}H NMR (400 MHz, dmso-*d*$_6$, H-H COSY): δ 0.64 – 0.68 (m, 2H, SiC*H*$_2$CH$_2$), 0.82 – 0.86 (m, 3H, *Me*), 1.22 – 1.29 (m, 18H, C*H*$_2$), 1.48 (p, *J* = 7.0 Hz, 2H, NCH$_2$C*H*$_2$), 1.59 (dt, *J* = 8.1, 1.2 Hz, 6H,SiC*H*$_2$CH), 1.72 – 1.79 (m, 2H, SiCH$_2$C*H*$_2$), 3.21 (td, *J* = 7.0, 5.6 Hz, 2H, NC*H*$_2$), 3.94 (t, *J* = 6.6 Hz, 2H, OC*H*$_2$), 4.82 – 4.90 (m, 6H, SiCH$_2$CHC*H*$_2$), 5.79 (ddt, *J* = 17.0, 10.1, 8.1 Hz, 3H, SiCH$_2$C*H*), 6.93, 7.80 (2 × d, *J* = 8.9 Hz, 2 × 2H, C*H*$_{Ph}$), 8.24 (t, *J* = 5.6 Hz, 1H, N*H*). ^{13}C {^{1}H} NMR (101 MHz, dmso-*d*$_6$, HSQC, HMBC): δ 7.1 (Si*C*H$_2$), 13.9 (*Me*), 19.2 (SiCH$_2$CH), 22.1 (CH$_2$), 22.7 (OCH$_2$CH$_2$), 26.5, 28.7, 28.8, 29.01, 29.04, 29.1, 29.2, 30.4, 31.3 (9 × *C*H$_2$), 39.1 (NCH$_2$), 70.0 (OCH$_2$), 113.6 (SiCH$_2$CHCH$_2$), 113.7 (2CH$_{Ph}$), 126.8 (*C*$_q$), 128.9 (2CH$_{Ph}$), 134.3 (CHCH$_2$), 160.7 (OC$_q$), 165.5 (*C*O). ^{29}Si {^{1}H} NMR (79 MHz, dmso-*d*$_6$): δ –0.03. HRMS (APCI+): Calcd. for [$C_{31}H_{52}NO_2Si$]$^+$ 498.3760, found [M+H]$^+$ 498.3752.

Dendron L2-15a. Triallylic dendritic compound **14a** (475 mg, 0.954 mmol), 2-(dimethylamino)ethanthiol hydrochloride (486 mg, 3.43 mmol) and DMPA (24 mg, 0.095 mmol) were dissolved in a mixture of distilled THF (2.5 mL) and MeOH (5 mL) in 10mL vial and deoxygenated. The reaction mixture was stirred under argon atmosphere and irradiated for 6 h. Afterwards, solvent was removed on rotary evaporator and the residue separated by nanofiltration in a mixture of DCM and methanol 2:1 obtaining dendron **L2-15a** (463 mg, 53 %, white foam). ^{1}H NMR (400 MHz, dmso-*d*$_6$, H-H COSY): δ 0.63 – 0.68 (m, 8H, SiC*H*$_2$), 0.83 – 0.86 (m, 3H, *Me*), 1.23 – 1.29 (m, 18H, C*H*$_2$), 1.49 – 1.58 (m, 8H, CH$_2$C*H*$_2$CH$_2$S, C*H*$_2$CH$_2$NH), 1.70 – 1.76 (m, 2H, OCH$_2$C*H*$_2$), 2.58 (t, *J* = 7.1 Hz, 6H, CH$_2$CH$_2$C*H*$_2$S), 2.73 (s, 18H, NH*Me*$_2$), 2.83 – 2.91 (m, 6H, SC*H*$_2$CH$_2$NH), 3.18 – 3.22 (m, 8H, NHC*H*$_2$, C*H*$_2$NHMe$_2$), 3.33 (br s, 3H, N*H*Me$_2$), 3.99 (t, *J* = 6.6 Hz, 2H, OC*H*$_2$), 6.95, 7.81 (2 × d, *J* = 8.9 Hz, 2 × 2H, C*H*$_{Ph}$), 8.31 (t, *J* = 5.6 Hz, 1H, N*H*). ^{13}C {^{1}H} NMR (101 MHz, dmso-*d*$_6$, HSQC, HMBC): δ 7.6 (CH$_2$Si), 11.1 (CH$_2$Si), 14.0 (*Me*), 22.1 (CH$_2$), 23.2 (OCH$_2$CH$_2$), 23.7 (CH$_2$CH$_2$CH$_2$S), 24.6 (SCH$_2$CH$_2$NH), 26.5, 28.7, 28.8 (3 × CH$_2$), 28.99 (2CH$_2$), 29.01, 29.04, 29.2, 31.3 (4 × CH$_2$), 34.5 (CH$_2$CH$_2$CH$_2$S), overlapped with DMSO from HSQC 39.1 (CH$_2$NH), 41.8 (NH*Me*$_2$), 55.8 (CH$_2$NHMe$_2$), 70.2 (OCH$_2$), 113.8 (2CH$_{Ph}$), 126.8 (C$_q$), 129.0 (2CH$_{Ph}$), 160.7 (C$_q$O), 165.5(CO). ^{29}Si {^{1}H} NMR (79 MHz, dmso-*d*$_6$): δ 4.09. HRMS (ESI+): Calcd. for [$C_{43}H_{85}N_4O_2S_3Si$]$^+$ 813.5598, found [M-3Cl-2H]$^+$ 813.5594.

Compound 13b. Methyl 4-hydroxybenzoate (2.0 g, 13 mmol) and octadecan-1-amine (11 g, 39 mmol) were stirred at 100 °C for 48 h under inert argon atmosphere. Afterwards, crude product was separated by column chromatography (eluent petrolether/EtOAc 2:1) to afford desired product **13b** (R_f = 0.40, 4.7 g, 91 %, white amorphous substance). ^{1}H NMR (400 MHz, dmso-*d*$_6$, H-H COSY): δ 0.83 – 0.86 (m, 3H, *Me*), 1.23 – 1.26 (m, 30H, C*H*$_2$), 1.47 (p, *J* = 6.9 Hz, 2H, NCH$_2$C*H*$_2$), 3.19 (td, *J* = 6.9, 5.6 Hz, 2H, NC*H*$_2$), 6.77, 7.69 (2 × d, *J* = 8.7 Hz, 2 × 2H, C*H*$_{Ph}$), 8.14 (t, *J* = 5.6 Hz, 1H, N*H*), 9.89 (s, 1H, O*H*). ^{13}C {^{1}H} NMR (101 MHz, dmso-*d*$_6$, HSQC, HMBC): δ 14.0 (*Me*), 22.1, 26.5, 28.7, 28.8 (4 × CH$_2$), 29.00, 29.02 (2 × 4CH$_2$), 29.04 (3CH$_2$), 29.3 (CH$_2$), 31.3 (NCH$_2$), 114.7 (2CH$_{Ph}$), 125.5 (C$_q$), 129.0 (2CH$_{Ph}$), 159.9 (OC$_q$), 165.7 (CO). HRMS (APCI+): Calcd. for [$C_{25}H_{44}NO_2$]$^+$ 390.3366, found [M+H]$^+$ 390.3368.

Compound 14b. N-octadecyl-4-hydroxybenzamide (**13b**, 500 mg, 1.29 mmol) and calcinated K_2CO_3 (195 mg, 1.41 mmol) were dissolved in dry acetonitrile (40 mL) under inert argon atmosphere. The mixture was stirred at reflux for 1 h. Triallyl(3-iodopropyl)silane (390 mg, 1.22 mmol) was added and the reaction mixture was stirred at reflux for 20 h. Afterwards, solvent was removed on rotary evaporator, ethyl acetate (150 mL) added and the mixture washed (3 × 75 mL 10% K_2CO_3 sol., 1 × 75 mL H_2O and 1 × 150 mL brine). Organic layer was dried by anhydrous $MgSO_4$ and solvent removed obtaining product **14b** (647 mg, 91 %, white amorphous solid). ^{1}H NMR (400 MHz, dmso-d_6, H-H COSY): δ 0.64 – 0.68 (m, 2H, $SiCH_2CH_2$), 0.83 – 0.86 (m, 3H, *Me*), 1.22 – 1.26 (m, 30 H, CH_2), 1.48 (p, *J* = 6.7 Hz, 2H, NCH_2CH_2), 1.60 (dt, *J* = 8.1, 1.2 Hz, 6H, $SiCH_2CH$), 1.72 – 1.79 (m, 2H, $SiCH_2CH_2$), 3.21 (td, *J* = 6.7, 5.6 Hz, 2H, NCH_2), 3.95 (t, *J* = 6.6 Hz, 2H, OCH_2), 4.83 – 4.91 (m, 6H, $CHCH_2$), 5.80 (ddt, *J* = 16.9, 10.1, 8.1 Hz, 3H, CH_2CH), 6.94, 7.78 (2 × d, *J* = 8.8 Hz, 2 × 2H, CH_{Ph}), 8.25 (t, *J* = 5.6 Hz, 1H, N*H*). ^{13}C {^{1}H} NMR (101 MHz, dmso-d_6, HSQC, HMBC): δ 7.1 ($SiCH_2$), 14.0 (*Me*), 19.2 ($SiCH_2CH$), 22.1 (CH_2), 22.7 (OCH_2CH_2), 26.5, 28.7, 28.76, 28.77, 28.97 (5 × CH_2), 29.03 (4CH_2), 29.04, 29.1 (2 × 2CH_2), 29.2, 31.3 (2 × CH_2), overlapped with DMSO from HSQC 39.1 (NCH_2), 70.0 (OCH_2), 113.7 ($SiCH_2CHCH_2$), 113.8 (2CH_{Ph}), 126.8 (C_q), 128.9 (2CH_{Ph}), 134.4 ($CHCH_2$), 160.7 (OC_q), 165.5 (*C*O). ^{29}Si {^{1}H} NMR (79 MHz, dmso-d_6): δ –0.40. HRMS (APCI+): Calcd. for $[C_{37}H_{64}NO_2Si]^+$ 582.4701, found $[M+H]^+$ 582.4678.

Dendron L2-15b. Triallylic dendritic compound **14b** (200 mg, 0.344 mmol), 2-(dimethylamino)ethanthiol hydrochloride (175 mg, 1.24 mmol) and DMPA (9 mg, 0.03 mmol) were dissolved in distilled THF (1 mL) and methanol (1 mL) in 5mL vial and deoxygenated. The reaction mixture was stirred under inert argon atmosphere and irradiated for 7 h. Afterwards, solvent was removed on rotary evaporator and product was purified by nanofiltration in a mixture of DCM and methanol 2:1 obtaining dendron **L2-15b** (334 mg, 96%, yellowish foam). ^{1}H NMR (400 MHz, dmso-d_6, H-H COSY): δ 0.63 – 0.67 (m, 8H, $SiCH_2$), 0.83 – 0.86 (m, 3H, *Me*), 1.22 – 1.28 (m, 30H, CH_2), 1.47 – 1.58 (m, 8H, $CH_2CH_2CH_2S$, CH_2CH_2NH), 1.68 – 1.75 (m, 2H, OCH_2CH_2), 2.58 (t, *J* = 7.1 Hz, 6H, $CH_2CH_2CH_2S$), 2.73 (s, 18H, $NHMe_2$), 2.84 – 2.88 (m, 6H, SCH_2CH_2NH), 3.18 – 3.22 (m, 8H, $NHCH_2$, CH_2NHMe_2), 3.99 (t, *J* = 6.6 Hz, 2H, OCH_2), 6.95, 7.81 (2 × d, *J* = 8.8 Hz, 2 × 2H, CH_{Ph}), 8.32 (t, *J* = 5.6 Hz, 1H, N*H*). ^{13}C {^{1}H} NMR (101 MHz, dmso-d_6, HSQC, HMBC): δ 7.6 (CH_2Si), 11.1 (CH_2Si), 14.0 (*Me*), 22.1 (CH_2), 23.2 (OCH_2CH_2), 23.7 ($CH_2CH_2CH_2S$), 24.6 (SCH_2CH_2NH), 26.5, 28.7, 28.8 (3 × CH_2), 28.99 (2CH_2), 29.02, 29.03 (2 × 4CH_2), 29.2, 31.3 (2 × CH_2), 34.5 ($CH_2CH_2CH_2S$), overlapped with DMSO from HSQC 39.1 (CH_2NH), 41.8 ($NHMe_2$), 55.8 (CH_2NHMe_2), 70.2 (OCH_2), 113.8 (2CH_{Ph}), 126.8 (C_q), 129.0 (2CH_{Ph}), 160.7 (C_qO), 165.5(CO). ^{29}Si {^{1}H} NMR (79 MHz, dmso-d_6): δ 4.09. HRMS (ESI+): Calcd. for $[C_{49}H_{97}N_4O_2S_3Si]^+$ 897.6543, found $[M-3Cl-2H]^+$ 897.6539.

Compound 16. Methyl 3,5-dihydroxybenzoate (1.00 g, 5.95 mmol), triallyl(3-iodopropyl)silane (3.62 g, 11.3 mmol) and calcinated K_2CO_3 (0.82 g, 6.0 mmol) were dissolved in dry acetonitrile (60 mL) under inert argon atmosphere and stirred at reflux for 72 h. Afterwards, solvent was removed on rotary evaporator and the mixture filtered through a short column of silicagel (pentane/Et_2O 10:1). Solvents were removed obtaining product **16** (3.27 g, 98 %, colourless viscous liquid). ^{1}H NMR (400 MHz, dmso-d_6, H-H COSY): δ 0.64 – 0.68 (m, 4H, $SiCH_2CH_2$), 1.60 (dt, *J* = 8.1, 1.2 Hz, 12H, $SiCH_2CH$), 1.71 – 1.79 (m, 4H, $SiCH_2CH_2$), 3.83 (s, 3H, *Me*), 3.94 (t, *J* = 6.6 Hz, 4H, OCH_2), 4.83 – 4.90 (m, 12H, $CHCH_2$), 5.80 (ddt, *J* = 16.9, 10.1, 8.1 Hz, 6H, CH_2CH), 6.71 (t, *J* = 2.3 Hz, 1H, CH_{Ph}), 7.02 (d, *J* = 2.3 Hz, 2H, CH_{Ph}). ^{13}C {^{1}H} NMR (101 MHz, dmso-d_6, HSQC, HMBC): δ 7.0 ($SiCH_2$), 19.2 ($SiCH_2CH$), 22.7 (OCH_2CH_2), 52.2 (*Me*), 70.2 (OCH_2), 106.0 (CH_{Ph}), 107.2 (2CH_{Ph}), 113.7 ($CHCH_2$), 131.5 (C_q), 134.4 ($CHCH_2$), 159.7 (OC_q), 165.9 (*C*O). ^{29}Si {^{1}H} NMR (79 MHz, dmso-d_6): δ 0.03. HRMS (APCI+): Calcd. for $[C_{32}H_{49}O_4Si_2]^+$ 553.3163, found $[M+H]^+$ 553.3157.

Compound 17. Dendritic methyl benzoate **16** (649 mg, 1.17 mmol) and NaOH (141 mg, 3.52 mmol) were dissolved in THF (8 mL) and MeOH (8 mL) under inert argon atmosphere and stirred at reflux for 24 h. Afterwards, EtOAc (200 mL) was added, the mixture washed (2 × 100 mL 1M HCl, 2 × 100 mL H_2O and 200 mL brine) and the organic layer dried by anhydrous $MgSO_4$. Solvents were removed obtaining product **17** (620 mg, 98 %, white amorphous substance).

^{1}H NMR (400 MHz, chloroform-*d*, H-H COSY): δ 0.70 – 0.74 (m, 4H, SiC*H*$_2$CH$_2$), 1.64 (dt, *J* = 8.1, 1.2 Hz, 12H, SiC*H*$_2$CH), 1.79 – 1.89 (m, 4H, SiCH$_2$C*H*$_2$), 3.94 (t, *J* = 6.7 Hz, 4H, OC*H*$_2$), 4.87 – 4.94 (m, 12H, CHC*H*$_2$), 5.80 (ddt, *J* = 16.5, 10.1, 8.1 Hz, 6H, CH$_2$C*H*), 6.67 (t, *J* = 2.4 Hz, 1H, C*H*$_{Ph}$), 7.22 (d, *J* = 2.4 Hz, 2H, C*H*$_{Ph}$). ^{13}C {^{1}H} NMR (101 MHz, chloroform-*d*, HSQC, HMBC): δ 7.6 (SiCH$_2$), 19.7 (SiCH$_2$CH), 23.5 (OCH$_2$CH$_2$), 70.9 (OCH$_2$), 107.6 (*C*H$_{Ph}$), 108.3 (2*C*H$_{Ph}$), 114.0 (CHCH$_2$), 131.1 (*C*$_q$), 134.3 (CHCH$_2$), 160.2 (OC$_q$), 171.5 (*C*O). ^{29}Si {^{1}H} NMR (79 MHz, chloroform-*d*): δ –0.07. HRMS (APCI+): Calcd. for [$C_{31}H_{47}O_4Si_2$]$^+$ 539.3007, found [M+H]$^+$ 539.3005.

Compound 18a. Dendritic benzoic acid **17** (300 mg, 557 μmol) and dodecylamine (124 mg, 668 μmol) were dissolved in dry DMF (10 mL) under inert argon atmosphere and stirred at RT for 5 min. TBTU (188 mg, 585 μmol) was added in dry DMF (5 mL) and the solution stirred at RT for 20 min. Then, DIPEA (0.11 mL, 0.59 mmol) was added and the reaction mixture was stirred at RT for another 4 h. Afterwards, DCM (75 mL) was added, the mixture washed (3 × 50 mL 1M HCl, 2 × 50 mL 1% sol. NaOH and 2 × 50 mL H_2O) and the organic layer dried by anhydrous $MgSO_4$. Solvent was removed obtaining product **18a** (391 mg, 98 %, yellowish viscous liquid). ^{1}H NMR (400 MHz, dmso-*d$_6$*, H-H COSY): δ 0.64 – 0.68 (m, 4H, SiC*H*$_2$CH$_2$), 0.84 (t, *J* = 7.2 Hz, 3H, *Me*), 1.23 – 1.27 (m, 18H, C*H*$_2$), 1.48 (p, *J* = 6.7 Hz, 2H, NCH$_2$C*H*$_2$), 1.60 (dt, *J* = 8.1, 1.2 Hz, 12H, SiC*H*$_2$CH), 1.70 – 1.78 (m, 4H, SiCH$_2$C*H*$_2$), 3.21 (td, *J* = 6.7, 5.7 Hz, 2H, NC*H*$_2$), 3.92 (t, *J* = 6.6 Hz, 4H, OC*H*$_2$), 4.83 – 4.90 (m, 12H, CHC*H*$_2$), 5.80 (ddt, *J* = 16.9, 10.1, 8.1 Hz, 6H, CH$_2$C*H*), 6.55 (t, *J* = 2.2 Hz, 1H, C*H*$_{Ph}$), 6.95 (d, *J* = 2.2 Hz, 2H, C*H*$_{Ph}$), 8.35 (t, *J* = 5.7 Hz, 1H, N*H*). ^{13}C {^{1}H} NMR (101 MHz, dmso-*d$_6$*, HSQC, HMBC): δ 7.2 (SiCH$_2$), 13.9 (*Me*), 19.2 (SiCH$_2$CH), 22.1 (*C*H$_2$), 22.7 (OCH$_2$CH$_2$), 26.4, 28.71, 28.74 (3 × *C*H$_2$), 29.96 (2CH$_2$), 29.00, 29.01, 29.04, 31.3 (4 × *C*H$_2$), overlapped with DMSO from HSQC 39.1 (NCH$_2$), 70.1 (OCH$_2$), 103.6 (*C*H$_{Ph}$), 105.6 (2CH$_{Ph}$), 113.7 (CHCH$_2$), 134.4 (CHCH$_2$), 136.7 (*C*$_q$), 159.5 (OC$_q$), 165.5 (*C*O). ^{29}Si {^{1}H} NMR (79 MHz, dmso-*d$_6$*): δ 0.04. HRMS (APCI+): Calcd. for [$C_{43}H_{72}NO_3Si_2$]$^+$ 706.5045, found [M+H]$^+$ 706.5043.

Dendron L2-19a. Hexaallylic dendritic compound **18a** (400 mg, 0.566 mmol), 2-(dimethylamino)ethanthiol hydrochloride (722 mg, 5.10 mmol) and DMPA (15 mg, 0.057 mmol) were dissolved in a mixture of distilled THF (2.5 mL) and MeOH (5 mL) in 10mL vial and deoxygenated. The reaction mixture was stirred under argon atmosphere and irradiated for 3 h. Afterwards, solvent was removed on rotary evaporator, DCM (10 mL) added and the white crystals filtered off using filter paper (properly washed with DCM). The filtrate was then purified by nanofiltration in a mixture of DCM and methanol 2:1 obtaining dendron **L2-19a** (717 mg, 81 %, white foam). ^{1}H NMR (400 MHz, dmso-*d$_6$*, H-H COSY): δ 0.63 – 0.68 (m, 16H, SiC*H*$_2$), 0.85 (t, *J* = 6.8 Hz, 3H, *Me*), 1.20 – 1.27 (m, 18H, C*H*$_2$), 1.51 – 1.59 (m, 14H, CH$_2$C*H*$_2$CH$_2$S, C*H*$_2$CH$_2$NH), 1.67 – 1.74 (m, 4H, OCH$_2$C*H*$_2$), 2.59 (t, *J* = 7.1 Hz, 12H, CH$_2$CH$_2$C*H*$_2$S), 2.74 (s, 36H, NH*Me*$_2$), 2.83 – 2.87 (m, 12H, SC*H*$_2$CH$_2$NH), 3.19 – 3.23 (m, 14H, NHC*H*$_2$, C*H*$_2$NHMe$_2$), 3.95 (t, *J* = 6.6 Hz, 4H, OC*H*$_2$), 6.57 (t, *J* = 2.2 Hz, 1H, C*H*$_{Ph}$), 6.99 (d, *J* = 2.2 Hz, 2H, C*H*$_{Ph}$), 8.46 (t, *J* = 5.7 Hz, 1H, N*H*). ^{13}C {^{1}H} NMR (101 MHz, dmso-*d$_6$*, HSQC, HMBC): δ 7.7 (CH$_2$Si), 11.1 (CH$_2$Si), 14.0 (*Me*), 22.1 (CH$_2$), 23.3 (OCH$_2$CH$_2$), 23.7 (CH$_2$CH$_2$CH$_2$S), 24.6 (SCH$_2$CH$_2$NH), 26.5, 28.7, 28.8 (3 × CH$_2$), 28.99 (2CH$_2$), 29.02 (CH$_2$), 29.04 (2CH$_2$), 31.3 (CH$_2$), 34.5 (CH$_2$CH$_2$CH$_2$S), overlapped with DMSO from HSQC 39.3 (CH$_2$NH), 41.9 (NH*Me*$_2$), 55.8 (CH$_2$NHMe$_2$), 70.4 (OCH$_2$), 103.6 (CH$_{Ph}$), 105.6 (2CH$_{Ph}$), 136.7 (*C*$_q$), 159.6 (*C*$_q$O), 165.5(CO). ^{29}Si {^{1}H} NMR (79 MHz, dmso-*d$_6$*): δ 4.07. HRMS (ESI+): Calcd. for [$C_{67}H_{138}N_7O_3S_6Si_2$]$^+$ 1336.8718, found [M-6Cl-5H]$^+$ 1336.8711; calcd. for [$C_{67}H_{139}N_7O_3S_6Si_2$]$^{2+}$ 668.9395, found [M-6Cl-4H]$^{2+}$ 668.9392; calcd. for [$C_{67}H_{140}N_7O_3S_6Si_2$]$^{3+}$ 446.2954, found [M-6Cl-3H]$^{3+}$ 446.2950.

Compound 18b. Dendritic benzoic acid **17** (200 mg, 371 μmol) and octadecylamine (109 mg, 445 μmol) were dissolved in dry DMF (8 mL) under inert argon atmosphere and stirred at 50 °C for 5 min. TBTU (125 mg, 390 μmol) was added in dry DMF (8 mL) and the solution stirred at 50 °C for 15 min. Then, DIPEA (0.07 mL, 0.4 mmol) was added and the reaction mixture was stirred at 50 °C for another 4 h. Afterwards, Et_2O (70 mL) was added, the mixture washed (3 × 30 mL 1M HCl, 2 × 30 mL 1% sol. NaOH, 2 × 30 mL H_2O and 50 mL brine) and the organic layer dried by anhydrous $MgSO_4$. Solvent was removed obtaining product **18b** (286 mg, 98 %, yellow-brown viscous substance). ^{1}H NMR

(400 MHz, chloroform-*d*, H-H COSY): δ 0.68 – 0.72 (m, 4H, SiC*H*$_2$CH$_2$), 0.86 – 0.89 (m, 3H, *Me*), 1.25 – 1.33 (m, 30H, C*H*$_2$), 1.57 (p, *J* = 7.3 Hz, 2H, NCH$_2$C*H*$_2$), 1.63 (dt, *J* = 8.1, 1.2 Hz, 12H, SiC*H*$_2$CH), 1.80 – 1.83 (m, 4H, SiCH$_2$C*H*$_2$), 3.42 (td, *J* = 7.3, 5.7 Hz, 2H, NC*H*$_2$), 3.91 (t, *J* = 6.7 Hz, 4H, OC*H*$_2$), 4.86 – 4.93 (m, 12H, CHC*H*$_2$), 5.80 (ddt, *J* = 16.3, 10.1, 8.1 Hz, 6H, CH$_2$C*H*), 6.03 (t, *J* = 5.7 Hz, 1H, N*H*), 6.53 (t, *J* = 2.2 Hz, 1H, C*H*$_{Ph}$), 6.84 (d, *J* = 2.2 Hz, 2H, C*H*$_{Ph}$). ^{13}C {^{1}H} NMR (101 MHz, chloroform-*d*, HSQC, HMBC): δ 7.7 (SiCH$_2$), 14.3 (*Me*), 19.7 (SiCH$_2$CH), 22.8 (*C*H$_2$), 23.5 (OCH$_2$CH$_2$), 27.2, 29.48, 29.51, 29.7 (4 × *C*H$_2$), 29.80 (4*C*H$_2$), 29.82 (*C*H$_2$), 29.9 (5*C*H$_2$), 32.1 (*C*H$_2$), 40.3 (NCH$_2$), 70.9 (OCH$_2$), 104.2 (*C*H$_{Ph}$), 105.4 (2*C*H$_{Ph}$), 114.0 (SiCH$_2$CH*C*H$_2$), 134.2 (*C*HCH$_2$), 137.2 (*C*$_q$), 160.4 (O*C*$_q$), 167.5 (*C*O). ^{29}Si {^{1}H} NMR (79 MHz, dmso-*d*$_6$): δ –0.08. HRMS (APCI+): Calcd. for $[C_{49}H_{84}NO_3Si_2]^+$ 790.5984, found $[M+H]^+$ 790.5988.

Dendron L2-19b. Hexaallylic dendritic compound **18b** (257 mg, 0.325 mmol), 2-(dimethylamino)ethanthiol hydrochloride (415 mg, 2.93 mmol) and DMPA (8 mg, 0.033 mmol) were dissolved in a mixture of distilled THF (4 mL) and MeOH (4 mL) in 10mL vial and deoxygenated. The reaction mixture was stirred under argon atmosphere and irradiated for 6 h which heated the reaction mixture to approximately 60 °C. Afterwards, solvent was removed on rotary evaporator and the residue was separated by nanofiltration in a mixture of DCM and methanol 1:10 obtaining dendron **L2-19b** (439 mg, 82 %, yellowish foam). ^{1}H NMR (400 MHz, dmso-*d*$_6$, H-H COSY): δ 0.63 – 0.68 (m, 16H, SiC*H*$_2$), 0.83 – 0.87 (m, 3H, *Me*), 1.18 – 1.27 (m, 30H, C*H*$_2$), 1.48 – 1.59 (m, 14H, CH$_2$C*H*$_2$CH$_2$S, C*H*$_2$CH$_2$NH), 1.67 – 1.74 (m, 4H, OCH$_2$C*H*$_2$), 2.59 (t, *J* = 7.0 Hz, 12H, CH$_2$CH$_2$C*H*$_2$S), 2.74 (s, 36H, NH*Me*$_2$), 2.83 – 2.87 (m, 12H, SC*H*$_2$CH$_2$NH), 3.18 – 3.23 (m, 14H, NHC*H*$_2$, C*H*$_2$NHMe$_2$), 3.95 (t, *J* = 6.6 Hz, 4H, OC*H*$_2$), 6.57 (t, *J* = 2.2 Hz, 1H, C*H*$_{Ph}$), 6.99 (d, *J* = 2.2 Hz, 2H, C*H*$_{Ph}$), 8.47 (t, *J* = 5.8 Hz, 1H, N*H*). ^{13}C {^{1}H} NMR (101 MHz, dmso-*d*$_6$, HSQC, HMBC): δ 7.8 (*C*H$_2$Si), 11.2 (*C*H$_2$Si), 14.0 (*Me*), 22.1 (*C*H$_2$), 23.2 (OCH$_2$CH$_2$), 23.7 (CH$_2$CH$_2$CH$_2$S), 24.6 (SCH$_2$CH$_2$NH), 26.5, 28.7, 28.8 (3 × *C*H$_2$), 28.99 (2CH$_2$), 29.01, 29.03, 29.04 (3 × 3CH$_2$), 31.3 (CH$_2$), 34.5 (CH$_2$CH$_2$CH$_2$S), overlapped with DMSO from HSQC 39.3 (*C*H$_2$NH), 41.9 (NH*Me*$_2$), 55.8 (*C*H$_2$NHMe$_2$), 70.4 (OCH$_2$), 103.6 (*C*H$_{Ph}$), 105.6 (2*C*H$_{Ph}$), 136.7 (*C*$_q$), 159.6 (*C*$_q$O), 165.5(*C*O). ^{29}Si {^{1}H} NMR (79 MHz, dmso-*d*$_6$): δ 4.07. HRMS (ESI+): Calcd. for $[C_{73}H_{150}N_7O_3S_6Si_2]^+$ 1420.9657, found $[M\text{-}6Cl\text{-}5H]^+$ 1420.9645; calcd. for $[C_{73}H_{151}N_7O_3S_6Si_2]^{2+}$ 729.9749, found $[M\text{-}6Cl\text{-}4H]^{2+}$ 729.9748.

Dimethyl 5-(benzyloxy)isophthalate. Protection of hydroxyl group of dimethyl 5-hydroxyisophthalate was performed according to a published procedure [40].

Compound 20. 5-(benzyloxy)isophthalate (13.0 g, 43.4 mmol) and KOH (3.89 g, 69.4 mmol, 1.60 eq) were dissolved in a mixture of THF (156 mL) and MeOH (156 mL) and stirred at reflux for 6 h. Afterwards, solvents were removed on rotary evaporator, H_2O (250 mL) was added to the residue and the mixture was cooled to 0 °C and acidified to pH ≈ 3. DCM (200 mL) was added and the two-phase system was firstly properly mixed and then filtered using celite and then filter paper. Organic phase was separated, washed (2 × 200 mL H_2O) and dried by anhydrous $MgSO_4$. Solvent was removed obtaining unsymmetrical product **20** (7.22 g, 58 %, white amorphous substance). Spectroscopic data were in agreement with literature [41].

Compound 21. Unsymmetrical benzoic acid **20** (1.00 g, 3.49 mmol) and CDI (567 mg, 3.49 mmol) were dissolved in DCM (25 mL) and stirred at RT for 20 min. Then, dodecane-1-amine (1.10 g, 5.93 mmol) was added and the reaction mixture was stirred at RT for 24 h. Afterwards, solvent was removed on rotary evaporator, the residue dissolved in EtOAc (60 mL) and the solution washed (40 mL H_2O, 2 × 40 mL 1M sol. HCl, again 40 mL H_2O, 2 × 40 mL 10% sol. K_2CO_3, again 40 mL H_2O and finally 40 mL brine) and dried by anhydrous $MgSO_4$. Solvent was removed obtaining product **21** (1.41 g, 89 %, white amorphous substance). ^{1}H NMR (400 MHz, Chloroform-*d*, H-H COSY): δ 0.86 – 0.89 (m, 3H, *Me*), 1.26 – 1.38 (m, 18H, C*H*$_2$), 1.61 (p, *J* = 7.0 Hz, 2H, NCH$_2$C*H*$_2$), 3.45 (td, *J* = 7.0, 5.8 Hz, 2H, NC*H*$_2$), 3.93 (s, 3H, *Me*O), 5.14 (s, 2H, C*H*$_2$ Bn), 6.19 (t, *J* = 5.8 Hz, 1H, N*H*), 7.34 – 7.45 (m, 5H, C*H*$_{Bn}$), 7.69 (dd, *J* = 2.6, 1.6 Hz, 1H, C*H*$_{Ph}$), 7.76 (dd, *J* = 2.6, 1.3 Hz, 1H, C*H*$_{Ph}$), 7.91 (dd, *J* = 1.6, 1.3 Hz, 1H, C*H*$_{Ph}$). ^{13}C {^{1}H} NMR (101 MHz, Chloroform-*d*, HSQC, HMBC): δ 14.3 (*Me*), 22.8, 27.1, 29.46, 29.49, 29.68, 29.73, 29.76 (7 × *C*H$_2$), 29.79 (2*C*H$_2$), 32.1 (*C*H$_2$), 40.4 (NCH$_2$), 52.6

(*Me*O), 70.6 (*C*H_2 Bn), 118.6, 118.8, 119.7 (3 × *C*H_{Ph}), 127.7 (2*C*H_{Bn}), 128.4 (*C*H_{Bn}), 128.8 (2*C*H_{Bn}), 131.8 (*C*$_q$), 136.2 (*C*$_{ipso}$ Bn), 136.7, 159.2 (2 × *C*$_q$), 166.3, 166.4 (2 × *C*O). HRMS (APCI+): Calcd. for $[C_{28}H_{40}NO_4]^+$ 454.2951, found $[M+H]^+$ 454.2955.

Compound 22. To unsymmetrical methyl benzoate **21** (1.39 g, 3.06 mmol) and 10% palladium on active carbon (261 mg, 0.245 mmol) pure EtOH (30 mL) and distilled THF (30 mL) were added and the mixture was stirred and bubbled with H_2 at 40 °C for 7 h. Afterwards, palladium was filtered off using celite and solvents removed obtaining product **22** (1.07 g, 96 %, yellowish amorphous substance). ^{1}H NMR (400 MHz, Chloroform-*d*): δ 0.82 – 0.94 (m, 3H, *Me*), 1.25 – 1.39 (m, 18H, C*H*$_2$), 1.63 (p, *J* = 7.3 Hz, 2H, NCH$_2$C*H*$_2$), 3.46 (td, *J* = 7.3, 5.8 Hz, 2H, NC*H*$_2$), 3.92 (s, 3H, *Me*O), 6.46 (t, *J* = 5.8 Hz, 1H, N*H*), 7.69 (dd, *J* = 2.6, 1.4 Hz, 1H, C*H*$_{Ph}$), 7.78 (t, *J* = 1.4 Hz, 1H, C*H*$_{Ph}$), 7.97 (dd, *J* = 2.6, 1.4 Hz, 1H, C*H*$_{Ph}$), 8.37 (s, 1H, O*H*). ^{13}C {^{1}H} NMR (101 MHz, Chloroform-*d*): δ 14.3 (*Me*), 22.8, 27.1, 29.45, 29.49, 29.6, 29.68, 29.73, 29.77, 29.79, 32.1 (10 × CH$_2$ dodecyl), 40.6 (NCH$_2$), 52.6 (*Me*O), 118.2, 120.21, 12.23 (3 × CH$_{Ph}$), 131.8, 135.7, 157.8 (3 × C$_q$), 166.7, 167.2 (2 × CO). HRMS (APCI+): Calcd. for $[C_{21}H_{34}NO_4]^+$ 364.2482, found $[M+H]^+$ 364.2480.

Compound 23. Unsymmetrical methyl benzoate **22** (1.07 g, 2.94 mmol) and K_2CO_3 (447 mg, 3.23 mmol) were dissolved in dry acetonitrile (80 mL) and stirred at reflux for 1 h. Then, triallyl(3-iodopropyl)silan (896 mg, 2.80 mmol) was added and the reaction mixture was stirred at reflux for another 20 h. Afterwards, solvent was removed on rotary evaporator, EtOAc (150 mL) was added and the mixture was washed (3 × 100 mL 10% sol. K_2CO_3, 100 mL 10% sol. Na_2SO_3, 100 mL H_2O and finally 150 mL brine) and dried by anhydrous $MgSO_4$. Solvent was removed and the residue separated by column chromatography (eluent petrolether/EtOAc 12:1) obtaining product **23** (1.32 g, 80 %, R_f = 0.50, colourless viscous liquid). ^{1}H NMR (400 MHz, Chloroform-*d*, H-H COSY): δ 0.66 – 0.75 (m, 2H, SiC*H*$_2$), 0.82 – 0.91 (m, 3H, *Me*), 1.22 – 1.41 (m, 18H, C*H*$_2$), 1.62 (dt, *J* = 8.1, 1.2 Hz, 6H, SiC*H*$_2$), 1.61 – 1.64 (m, 2H, NCH$_2$C*H*$_2$), 1.77 – 1.89 (m, 2H, SiCH$_2$C*H*$_2$), 3.44 (td, *J* = 7.3, 5.6 Hz, 2H, NC*H*$_2$), 3.92 (s, 3H, COO*Me*), 3.97 (t, *J* = 6.6 Hz, 2H, OC*H*$_2$), 4.86 – 4.92 (m, 6H, CHC*H*$_2$), 5.79 (ddt, *J* = 17.0, 10.1, 8.1 Hz, 3H, CH$_2$C*H*), 6.24 (t, *J* = 5.6 Hz, 1H, N*H*), 7.58 (dd, *J* = 2.6, 1.5 Hz, 1H, C*H*$_{Ph}$), 7.64 (dd, *J* = 2.6, 1.3 Hz, 1H, C*H*$_{Ph}$), 7.88 (t, *J* = 1.3 Hz, 1H, C*H*$_{Ph}$). ^{13}C {^{1}H} NMR (101 MHz, Chloroform-*d*, HSQC, HMBC): δ 7.6 (SiCH$_2$), 14.2 (*Me*), 19.6 (CHCH$_2$Si), 22.8 (*C*H$_2$), 23.4 (OCH$_2$CH$_2$), 27.1, 29.45, 29.47, 29.66, 29.72, 29.75, 29.76, 29.77, 32.0 (9 × *C*H$_2$), 40.4 (NCH$_2$), 52.5 (COO*Me*), 71.1 (OCH$_2$), 114.0 (CHCH$_2$), 118.4 (2CH$_{Ph}$), 119.2 (*C*H$_{Ph}$), 131.7 (C$_q$), 134.2 (CHCH$_2$), 136.6 (C$_q$), 159.5 (OC$_q$), 166.4, 166.5 (2 × *C*O). ^{29}Si {^{1}H} NMR (79 MHz, Chloroform-*d*): δ –0.14. HRMS (APCI+): Calcd. for $[C_{33}H_{54}NO_4Si]^+$ 556.3816, found $[M+H]^+$ 556.3816.

Compound 24. Unsymmetrical dendritic ester **23** (500 mg, 0.899 mmol) and KOH (151 mg, 2.70 mmol) were dissolved in a mixture of distilled THF (10 mL) and MeOH (10 mL) and stirred at reflux for 100 h. Afterwards, solvents were removed on rotary evaporator, H_2O (60 mL) was added and the mixture was acidified to pH ≈ 2. The mixture was then extracted (3 × 25 mL EtOAc) and the combined organic layers washed (50 mL H_2O and 50 mL brine) and dried by anhydrous $MgSO_4$. Solvent was removed obtaining product **24** (436 mg, 89 %, white amorphous substance). ^{1}H NMR (400 MHz, Chloroform-*d*, H-H COSY): δ 0.69 – 0.77 (m, 2H, SiC*H*$_2$), 0.84 – 0.90 (m, 3H, *Me*), 1.23 – 1.42 (m, 18H, C*H*$_2$), 1.63 (dt, *J* = 8.1, 1.3 Hz, 6H, SiC*H*$_2$), 1.62 – 1.65 (m, 2H, NCH$_2$C*H*$_2$), 1.78 – 1.89 (m, 2H, SiCH$_2$C*H*$_2$), 3.47 (td, *J* = 7.2, 5.7 Hz, 2H, NC*H*$_2$), 4.00 (t, *J* = 6.6 Hz, 2H, OC*H*$_2$), 4.87 – 4.93 (m, 6H, CHC*H*$_2$), 5.80 (ddt, *J* = 16.5, 10.1, 8.1 Hz, 3H, CH$_2$C*H*), 6.31 (t, *J* = 5.7 Hz, 1H, N*H*), 7.64 (dd, *J* = 2.6, 1.5 Hz, 1H, C*H*$_{Ph}$), 7.72 (dd, *J* = 2.6, 1.4 Hz, 1H, C*H*$_{Ph}$), 7.96 (t, *J* = 1.4 Hz, 1H, C*H*$_{Ph}$). ^{13}C {^{1}H} NMR (101 MHz, Chloroform-*d*, HSQC, HMBC): δ 7.7 (SiCH$_2$), 14.3 (*Me*), 19.7 (CHCH$_2$Si), 22.8 (CH$_2$), 23.4 (OCH$_2$CH$_2$), 27.2, 29.47, 29.47, 29.69 (4 × CH$_2$), 29.74 (2CH$_2$), 29.77, 29.79, 32.1 (3 × CH$_2$), 40.5 (NCH$_2$), 71.2 (OCH$_2$), 114.1 (CHCH$_2$), 118.9, 119.2, 119.8 (3 × *C*H$_{Ph}$), 130.9 (*C*$_q$), 134.2 (CHCH$_2$), 136.7 (*C*$_q$), 159.6 (O*C*$_q$), 166.5, 170.5 (2 × *C*O). ^{29}Si {^{1}H} NMR (79 MHz, Chloroform-*d*): δ -0.12. HRMS (APCI+): Calcd. for $[C_{32}H_{52}NO_4Si]^+$ 542.3660, found $[M+H]^+$ 542.3662.

Compound 25. Unsymmetrical dendritic benzoic acid **24** (165 mg, 359 μmol) and mono Boc-protected 2,2′-(ethylenedioxy)bis(ethylamine) (107 mg, 431 μmol) were dissolved in dry acetonitrile (40 mL) under inert argon

atmosphere and stirred at 60 °C for 10 min. After cooling back to RT, TBTU (121 mg, 377 μmol) was added in dry acetonitrile (10 mL) and the solution stirred at RT for 10 min. Then, DIPEA (0.07 mL, 0.4 mmol) was added and the reaction mixture was stirred at RT for another 3 h. Afterwards, solvent was removed on rotary evaporator, DCM (70 mL) was added, the mixture washed (4 × 30 mL sol. of HCl at pH ≈ 3-4 and 2 × 30 mL H_2O) and the organic layer dried by anhydrous $MgSO_4$. Solvent was removed obtaining product **25** (254 mg, 92 %, brown viscous liquid). ^{1}H NMR (400 MHz, dmso-*d*$_6$, H-H COSY): δ 0.67 – 0.71 (m, 2H, SiC*H*$_2$), 0.82 – 0.86 (m, 3H, *Me*), 1.19 – 1.29 (m, 18H, C*H*$_2$), 1.36 (s, 9H, *Me* Boc), 1.51 (p, *J* = 7.0 Hz, 2H, NCH$_2$C*H*$_2$), 1.61 (dt, *J* = 8.2, 1.2 Hz, 6H, SiC*H*$_2$), 1.74 – 1.82 (m, 2H, OCH$_2$C*H*$_2$), 3.05 (td, *J* = 6.1, 5.7 Hz, 2H, C*H*$_2$NHBoc), 3.24 (td, *J* = 6.7, 5.6 Hz, 2H, NHC*H*$_2$), 3.35 – 3.38 (m, 2H, BocNHCH$_2$C*H*$_2$), 3.42 (td, *J* = 6.7, 5.6 Hz, 2H, NHC*H*$_2$CH$_2$O), 3.48 – 3.53 (m, 6H, C*H*$_2$O), 4.00 (t, *J* = 6.5 Hz, 2H, OC*H*$_2$), 4.83 – 4.91 (m, 6H, CHC*H*$_2$), 5.80 (ddt, *J* = 17.0, 10.2, 8.2 Hz, 3H, C*H*CH$_2$), 6.73 (t, *J* = 5.7 Hz, 1H, N*H*Boc), 7.48, 7.49, 7.89 (3 × t, *J* = 1.5 Hz, 3 × 1H, CH$_{Ph}$), 8.49, 8.58 (2 × t, *J* = 5.6 Hz, 2 × 1H, N*H*). ^{13}C {^{1}H} NMR (101 MHz, dmso-*d*$_6$, HSQC, HMBC): δ 7.2 (SiCH$_2$), 13.9 (*Me*), 19.2 (SiCH$_2$CH), 22.1 (CH$_2$), 22.8 (OCH$_2$CH$_2$CH$_2$), 26.5 (CH$_2$), 28.2 (*Me* Boc), 28.7, 28.8, 28.99, 29.01 (4× CH$_2$), 29.02 (2CH$_2$), 29.1, 31.3 (2 × CH$_2$), overlapped with DMSO from HSQC 39.27 (CONHCH$_2$), from HSQC 39.28 (CONHCH$_2$CH$_2$O), from HSQC 39.7 (CH$_2$NHBoc), 68.9 (OCH$_2$CH$_2$NH), 69.2 (CH$_2$CH$_2$NHBoc), 69.5, 69.6 (OCH$_2$CH$_2$O), 70.3 (OCH$_2$CH$_2$CH$_2$), 77.6 (*C$_q$* Boc), 113.7 (CHCH$_2$), 115.4, 115.7, 118.5 (3 × CH$_{Ph}$), 134.4 (CHCH$_2$), 135.9, 136.2 (2 × C$_q$), 155.6 (CO Boc), 158.4 (C$_q$O), 165.4, 165.6 (2 × CO). ^{29}Si {^{1}H} NMR (79 MHz, dmso-*d*$_6$): δ 0.02. HRMS (APCI+): Calcd. for $[C_{38}H_{66}N_3O_5Si]^+$ 672.4766, found $[M\text{-}Boc+2H]^+$ 672.4892.

Dendron 26. Triallylic dendritic unsymmetrical diamide **25** (225 mg, 0.291 mmol), 2-(dimethylamino)ethanthiol hydrochloride (413 mg, 2.91 mmol) and DMPA (15 mg, 0.058 mmol) were dissolved in a mixture of distilled THF (2.5 mL) and MeOH (5 mL) in 10mL vial and deoxygenated. The reaction mixture was stirred under argon atmosphere and irradiated for 15 h. Afterwards, solvent was removed on rotary evaporator, DCM (5 mL) added and the white crystals filtered off using filter paper (properly washed with DCM). The filtrate was then purified by nanofiltration in a mixture of DCM and methanol 10:1 obtaining dendron **26** (223 mg, 64 %, yellowish viscous substance). ^{1}H NMR (400 MHz, dmso-*d*$_6$, H-H COSY): δ 0.64 – 0.68 (m, 8H, SiC*H*$_2$), 0.82 – 0.86 (m, 3H, *Me*), 1.17 – 1.29 (m, 18H, C*H*$_2$), 1.36 (s, 9H, *Me* Boc), 1.51 – 1.56 (m, 10H, 3CH$_2$C*H*$_2$CH$_2$S, C*H*$_2$CH$_2$NH), 1.70 – 1.79 (m, 2H, OCH$_2$C*H*$_2$), 2.59 (t, *J* = 7.1 Hz, 6H, CH$_2$CH$_2$C*H*$_2$S), 2.74 (s, 18H, NH*Me*$_2$), 2.83 – 2.87 (m, 6H, SC*H*$_2$CH$_2$NH), 3.04 (q, *J* = 6.0 Hz, 2H, C*H*$_2$NHBoc), 3.19 – 3.25 (m, 8H, 3NHMe$_2$C*H*$_2$, C*H*$_2$NH), 3.34 – 3.44 (m, 4H, NHC*H*$_2$, OC*H*$_2$CH$_2$NHBoc), 3.48 – 3.54 (m, 6H, C*H*$_2$O), 4.04 (t, *J* = 6.5 Hz, 2H, OC*H*$_2$), 6.75 (t, *J* = 6.0 Hz, 1H, N*H*), 7.51 (t, *J* = 1.5 Hz, 2H, C*H*$_{Ph}$), 7.98 (d, *J* = 1.5 Hz, 1H, C*H*$_{Ph}$), 8.63 (t, *J* = 5.7 Hz, 1H, N*H*), 8.72 (t, *J* = 5.6 Hz, 1H, N*H*). ^{13}C {^{1}H} NMR (101 MHz, dmso-*d*$_6$, HSQC, HMBC): δ 7.8 (CH$_2$Si), 11.1 (CH$_2$Si), 14.0 (*Me*), 22.1 (CH$_2$), 23.3 (OCH$_2$CH$_2$), 23.7 (CH$_2$CH$_2$CH$_2$S), 24.6 (SCH$_2$CH$_2$NH), 26.5 (CH$_2$), 28.3 (*Me* Boc), 28.7, 28.8, 29.00 (3 × CH$_2$), 29.03 (3CH$_2$), 29.1, 31.3 (2 × CH$_2$), 34.5 (CH$_2$CH$_2$CH$_2$S), overlapped with DMSO from HSQC 39.1 (OCH$_2$CH$_2$NH), from HSQC 39.2 (CH$_2$NH), from HSQC 39.6 (CH$_2$NHBoc), 41.9 (NH*Me*$_2$), 55.8 (CH$_2$NHMe$_2$), 68.8 (OCH$_2$CH$_2$NH), 69.2 (OCH$_2$CH$_2$NHBoc), 69.5, 69.6 (OCH$_2$CH$_2$O), 70.6 (OCH$_2$), 77.6 (C$_q$ Boc), 115.6, 115.8, 118.4 (3 × CH$_{Ph}$), 135.8, 136.1 (2 × C$_q$), 155.6 (CO Boc), 158.5 (C$_q$O), 165.3, 165.6 (2 × CO). ^{29}Si {^{1}H} NMR (79 MHz, dmso-*d*$_6$): δ 4.09. HRMS (ESI+): Calcd. for $[C_{55}H_{107}N_6O_7S_3Si]^+$ 1087.7127, found $[M\text{-}3Cl\text{-}2H]^+$ 1087.7137; calcd. for $[C_{55}H_{108}N_6O_7S_3Si]^{2+}$ 544.3599, found $[M\text{-}3Cl\text{-}1H]^{2+}$ 544.3601.

Dendron 27. Unsymmetrical dendron **26** with protected terminal aminogroup (36 mg, 30 μmol) was dissolved in DCM (2.3 mL) and cooled to 0 °C. TFA (0.23 mL) was slowly added and the reaction mixture was stirred at 0 °C for 4 h. Afterwards, solvent and the excess of TFA were removed on water-pump obtaining dendron **27** (43 mg, 98 %, yellow-brown viscous substance). ^{1}H NMR (400 MHz, dmso-*d*$_6$, H-H COSY): δ 0.63 – 0.67 (m, 8H, SiC*H*$_2$), 0.82 – 0.86 (m, 3H, *Me*), 1.17 – 1.29 (m, 18H, C*H*$_2$), 1.43 – 1.55 (m, 8H, 3CH$_2$C*H*$_2$CH$_2$S, C*H*$_2$CH$_2$NH), 1.70 – 1.79 (m, 2H, OCH$_2$C*H*$_2$), 2.57 (t, *J* = 7.1 Hz, 6H, CH$_2$CH$_2$C*H*$_2$S), 2.76 – 2.80 (m, 6H, SC*H*$_2$CH$_2$NH), 2.78 (s, 18H, N$^+$H*Me*$_2$), 2.97 (q, *J* = 5.5 Hz, 2H,

C*H*$_2$N$^+$H$_3$), 3.22 – 3.27 (m, 8H, 3NHMe$_2$C*H*$_2$, C*H*$_2$NH dodecyl), 3.43 (td, *J* = 6.2, 5.6 Hz, 2H, C*H*$_2$NH), 3.54 (t, *J* = 6.2 Hz, 2H, OC*H*$_2$CH$_2$NH), 3.57 – 3.60 (m, 6H, OC*H*$_2$), 4.01 (t, *J* = 6.5 Hz, 2H, OC*H*$_2$), 7.49 (d, *J* = 1.5 Hz, 2H, C*H*$_{Ph}$), 7.90 (br s, 4H, C*H*$_{Ph}$, N$^+$*H*$_3$), 8.54, 8.64 (2 × t, *J* = 5.6 Hz, 2 × 1H, N*H*), 10.00 (br s, 3H, N$^+$*H*Me$_2$). ^{13}C {^{1}H} NMR (101 MHz, dmso-*d*$_6$, HSQC, HMBC): δ 7.7 (CH$_2$Si), 11.2 (CH$_2$Si), 14.0 (*Me*), 22.1 (CH$_2$), 23.3 (OCH$_2$CH$_2$), 23.7 (CH$_2$CH$_2$CH$_2$S), 24.9 (SCH$_2$CH$_2$NH), 26.5, 28.7, 28.8, 29.03, 29.05 (5 × CH$_2$), 29.06 (2CH$_2$), 29.1, 31.3 (2 × CH$_2$), 34.6 (CH$_2$CH$_2$CH$_2$S), 38.6 (CH$_2$N$^+$H$_3$), overlapped with DMSO from HSQC 39.2 (OCH$_2$CH$_2$NH), from HSQC 39.4 (CH$_2$NH), 42.1 (NH*Me*$_2$), 55.9 (CH$_2$NHMe$_2$), 66.7 (OCH$_2$CH$_2$N$^+$H$_3$), 68.9 (CONHCH$_2$CH$_2$O), 69.5, 69.7 (OCH$_2$CH$_2$O), 70.6 (OCH$_2$), 115.6, 115.8 (2 × CH$_{Ph}$), 116.1 (q, $^1J_{CF}$ = 293.2 Hz, CF$_3$), 118.5 (CH$_{Ph}$), 135.9, 136.3 (2 × C$_q$), 158.5 (C$_q$O), 158.6 (q, $^2J_{CF}$ = 35.1 Hz, CF$_3$COO), 165.4, 165.8 (2 × CO). ^{29}Si {^{1}H} NMR (79 MHz, dmso-*d*$_6$): δ 4.00. ^{19}F NMR (377 MHz, dmso-*d*$_6$): δ –75.85. HRMS (ESI+): Calcd. for [$C_{50}H_{99}N_6O_5S_3Si$]$^+$ 987.6603, found [M-4(CF$_3$COOH)+H]$^+$ 987.6609; calcd. for [$C_{50}H_{100}N_6O_5S_3Si$]$^{2+}$ 494.3337, found [M-4(CF$_3$COOH)+2H]$^{2+}$ 494.3368.

Dendron L3-28. Unsymmetrical dendron **27** with terminal aminogroup (29.5 mg, 20.4 μmol) and cyanine 5 NHS ester (15.0 mg, 22.5 μmol) were dissolved in dry DCM (2.5 mL) under inert argon atmosphere, DIPEA (29 μl, 163 μmol) was added and the mixture stirred at RT in darkness for 24 h. Afterwards, 1 M HCl.Et$_2$O (0.20 mL, 0.20 mmol) was slowly added at vigorous stirring. The reaction mixture was separated by nanofiltration in methanol obtaining dendron **L3-28** (28.6 mg, 91 %, intensively blue amorphous substance). ^{1}H NMR (400 MHz, dmso-*d*$_6$, H-H COSY): δ 0.64 – 0.68 (m, 8H, SiC*H*$_2$), 0.84 (t, *J* = 6.6 Hz, 3H, *Me*), 1.16 – 1.35 (m, 20H, C*H*$_2$), 1.51 – 1.59 (m, 10H, SiCH$_2$C*H*$_2$, 2C*H*$_2$), 1.66 – 1.77 (m, 4H, C$_q$OCH$_2$C*H*$_2$, C*H*$_2$), 1.67 (s, 12H, C*Me*$_2$), 2.06 (t, *J* = 7.2 Hz, 2H, C*H*$_2$), 2.58 (t, *J* = 7.1 Hz, 6H, SC*H*$_2$), 2.73 (s, 18H, N*Me*$_2$), 2.83 – 2.87 (m, 6H, SC*H*$_2$), 3.11 – 3.26 (m, 10H, 2C*H*$_2$NH, C*H*$_2$NMe$_2$), 3.35 – 3.42 (m, 4H, C*H*$_2$NH, C*H*$_2$O), 3.47 – 3.56 (m, 6H, C*H*$_2$O), 3.59 (s, 3H, N*Me*), 4.02 (t, *J* = 6.6 Hz, 2H, C$_q$OC*H*$_2$), 4.07 (t, *J* = 7.5 Hz, 2H, C*H*$_2$), 6.25, 6.30 (2 × d, *J* = 13.3 Hz, 2 × 1H, C*H*), 6.56 (t, *J* = 13.3 Hz, 1H, C*H*), 7.21 – 7.26 (m, 2H, C*H*$_{Ph}$), 7.35 – 7.41 (m, 4H, C*H*$_{Ph}$), 7.52 (br s, 2H, C*H*$_{Ph}$), 7.40 (d, *J* = 7.4 Hz, 2H, C*H*$_{Ph}$), 7.88 (t, *J* = 5.2 Hz, 1H, N*H*), 8.03 (br s, 1H, C*H*$_{Ph}$), 8.34 (t, *J* = 13.3 Hz, 2H, C*H*), 8.67, 8.77 (2 × t, *J* = 5.5 Hz, 2 × 1H, N*H*). ^{13}C {^{1}H} NMR (101 MHz, dmso-*d*$_6$, HSQC, HMBC): δ 7.7 (SiCH$_2$), 11.1 (CH$_2$Si), 14.0 (*Me*), 22.1 (CH$_2$), 23.3 (SiCH$_2$CH$_2$), 23.8 (SCH$_2$CH$_2$CH$_2$Si), 24.7 (CH$_2$S), 24.9, 25.7, 26.5, 26.7 (4 × CH$_2$), 27.0, 27.2 (2 × C*Me*$_2$), 28.7, 28.8, 28.99 (3 × CH$_2$), 29.03 (3CH$_2$), 29.04 (CH$_2$), 31.1 (N*Me*), 31.3 (CH$_2$), 34.5 (CH$_2$S), 35.0 (CH$_2$), 38.4 (CH$_2$NH), overlapped with DMSO from HSQC 39.2, 39.1 (2 × CH$_2$NH), 41.9 (N*Me*$_2$), 43.3 (CH$_2$), 48.8, 48.9 (2 × CMe$_2$), 55.8 (CH$_2$NMe$_2$), 68.8, 69.1, 69.6, 69.8 (4 × OCH$_2$), 70.6 (OCH$_2$CH$_2$CH$_2$), 103.1, 103.3 (2 × CH), 111.0 (2CH$_{Ph}$), 115.6, 115.8, 118.4 (3 × CH$_{Ph}$), 122.3, 122.4, 124.6, 124.7 (4 × CH$_{Ph}$), 125.4 (CH), 128.3, 128.4 (2 × CH$_{Ph}$), 135.7, 136.1, 141.0, 141.1, 142.0, 142.8 (6 × C$_q$), 154.0, 154.1 (2 × CH), 158.5 (C$_q$O), 165.3, 165.6, 172.0 (3 × CO), 172.5, 173.2 (2 × C$_q$). ^{29}Si {^{1}H} NMR (79 MHz, dmso-*d*$_6$): δ 4.06. HRMS (ESI+): Calcd. for [$C_{82}H_{135}N_8O_6S_3Si$]$^+$ 100% 1452.9459, found [M-BF$_4$-3HCl]$^+$ 1452.9463; calcd. for [$C_{82}H_{136}N_8O_6S_3Si$]$^{2+}$ 726.9766, found [M-BF$_4$-3HCl+H]$^{2+}$ 726.9762.

Dendron L3-29. Unsymmetrical dendron **27** with terminal aminogroup (98 mg, 0.068 mmol) and D-biotin (20 mg, 0.081 mmol) were dissolved in dry DMF (2 mL) under inert argon atmosphere and stirred at RT for 10 min. TBTU (24 mg, 0.075 mmol) was added in dry DMF (2 mL) and the mixture stirred at 40 °C for 15 min. DIPEA (0.10 mL, 0.54 mmol) was added and the mixture stirred at 40 °C for 4 h. The reaction mixture was separated by nanofiltration in a mixture of DCM and methanol 2:1 obtaining dendron with free tertiary amino groups (74 mg, 90 %, brownish viscous substance). ^{1}H NMR (400 MHz, dmso-*d*$_6$, H-H COSY): δ 0.62 – 0.66 (m, 8H, SiC*H*$_2$), 0.84 (t, *J* = 6.7 Hz, 3H, *Me*), 1.19 – 1.27 (m, 20H, C*H*$_2$), 1.45 – 1.54 (m, 12H, SiCH$_2$C*H*$_2$, 3C*H*$_2$), 1.71 – 1.75 (m, 2H, C$_q$OCH$_2$C*H*$_2$), 2.05 (t, *J* = 7.4 Hz, 2H, C*H*$_2$), 2.12 (s, 12H, N*Me*$_2$), 2.39 (dd, *J* = 8.9, 5.9 Hz, 6H, C*H*$_2$NMe$_2$), 2.52 – 2.59 (m, 13H, SC*H*$_2$), 2.81 (dd, *J* = 12.4, 5.1 Hz, 1H, SCH*H*), 3.08 (ddd, *J* = 8.5, 6.1, 4.3 Hz, 1H, SC*H*), 3.17 (q, *J* = 5.7 Hz, 2H, C*H*$_2$NH), 3.24 (td, *J* = 7.5, 5.6 Hz, 2H, C*H*$_2$NH), 3.39 (t, *J* = 5.7 Hz, 2H, OC*H*$_2$CH$_2$NH), 3.42 (q, *J* = 5.6 Hz, 2H, C*H*$_2$NH), 3.52 – 3.56 (m, 6H, C*H*$_2$O), 4.01 (t, *J* = 6.5 Hz, 2H, C$_q$OC*H*$_2$), 4.12 (ddd, *J* = 7.5, 4.3, 1.8 Hz, 1H, SCHC*H*), 4.29 (dd, *J* = 7.5, 5.1 Hz, 1H, SCH$_2$C*H*), 6.35, 6.41 (2 × s, 2 × 1H,

CO(N*H*)$_2$), 7.49 (d, *J* = 1.5 Hz, 2H, C*H*$_{Ph}$), 7.81 (t, *J* = 5.7 Hz, 1H, N*H*), 7.89 (t, *J* = 1.5 Hz, 1H, C*H*$_{Ph}$), 8.48, 8.58 (2 × t, *J* = 5.6 Hz, 2 × 1H, N*H*). ^{13}C {^{1}H} NMR (101 MHz, dmso-*d*$_6$, HSQC, HMBC): δ 7.8 (SiCH$_2$), 11.2 (CH$_2$Si), 14.0 (*Me*), 22.1 (CH$_2$), 23.3 (SiCH$_2$CH$_2$), 23.9(SCH$_2$CH$_2$CH$_2$Si), 25.3, 26.5, 23.0, 28.2, 28.7, 28.8 (6 × CH$_2$), 28.96 (2CH$_2$), 28.99, 29.01 (2 × CH$_2$), 29.02 (CH$_2$CH$_2$NMe$_2$), 29.1, 31.3, 35.1 (3 × CH$_2$), 35.2 (CH$_2$S), 38.4 (NHCH$_2$), overlapped with DMSO 39.8 (SCH$_2$CH), 39.3 (2CH$_2$NH), 44.9 (N*Me*$_2$), 55.4 (SCH), 59.2 (CHCH$_2$S), 59.2 (CH$_2$NMe$_2$), 61.0 (CHCHS), 68.9, 69.2 (2 × OCH$_2$), 69.6 (2 CH$_2$O), 70.5 (OCH$_2$CH$_2$CH$_2$), 115.4, 115.7, 118.5 (3 × CH$_{Ph}$), 135.8, 136.2 (2 × C$_q$), 158.4 (C$_q$O), 162.7 (CO(NH)$_2$), 165.4, 165.6, 172.1 (3 × CO). ^{29}Si {^{1}H} NMR (79 MHz, dmso-*d*$_6$): δ 4.02. HRMS (ESI+): Calcd. for [$C_{60}H_{113}N_8O_7S_4Si$]$^+$ 1213.7379, found [M+H]$^+$ 1213.7379; calcd. for [$C_{60}H_{114}N_8O_7S_4Si$]$^{2+}$ 607.3726, found [M+2H]$^{2+}$ 607.3725. Dendron with free amino groups (69 mg, 0.057 mmol) was dissolved in dry DCM (10 mL) and cooled to 0 °C. 1 M solution of HCl · Et$_2$O (0.07 mL, 0.07 mmol) was added dropwise and the reaction mixture stirred at 0 °C for 5 min. Afterwards, solvent and the excess of HCl · Et$_2$O were removed on water-pump, DCM (5 mL) was added and removed again and the dendron **L3-29** dried on rotary evaporator (72 mg, 95 %, brownish viscous substance). ^{1}H NMR (400 MHz, dmso-*d*$_6$, H-H COSY): δ 0.64 – 0.68 (m, 8H, SiC*H*$_2$), 0.85 (t, *J* = 6.6 Hz, 3H, C*H*$_3$), 1.18 – 1.29 (m, 20H, C*H*$_2$), 1.48 – 1.57 (m, 6H, SiCH$_2$C*H*$_2$, 6H, C*H*$_2$), 1.71 – 1.77 (m, 2H, C$_q$OCH$_2$C*H*$_2$), 2.05 (t, *J* = 7.3 Hz, 2H, C*H*$_2$), 2.56 – 2.59 (m, 7H, SC*H*$_2$), 2.63 (s, 18H, N*Me*$_2$), 2.77 – 2.83 (m, 7H, SC*H*$_2$), 3.08 – 3.09 (m, 7H, SC*H*, C*H*$_2$NMe$_2$), 3.17 (q, *J* = 5.5 Hz, 2H, C*H*$_2$NH), 3.24 (td, *J* = 7.5, 5.7 Hz, 2H, C*H*$_2$NH), 3.39 (t, *J* = 7.5 Hz, 2H, OC*H*$_2$CH$_2$NH), 3.42 (q, *J* = 5.7 Hz, 2H, C*H*$_2$NH), 3.51 – 3.56 (m, 6H, C*H*$_2$O), 4.03 (t, *J* = 6.6 Hz, 2H, C$_q$OC*H*$_2$), 4.10 – 4.13 (m, 1H, SCHC*H*), 4.28 – 4.31 (m, 1H, SCH$_2$C*H*), 6.36, 6.41 (2 × s, 2 × 1H, CO(N*H*)$_2$), 7.51 (br s, 2H, C*H*$_{Ph}$), 7.85 (t, *J* = 5.5 Hz, 1H, N*H*), 7.98 (br s, 1H, C*H*$_{Ph}$), 8.60, 8.70 (2 × t, *J* = 5.7 Hz, 2 × 1H, N*H*). ^{13}C {^{1}H} NMR (101 MHz, dmso-*d*$_6$, HSQC, HMBC): δ 7.7 (SiCH$_2$), 11.2 (CH$_2$Si), 14.0 (*Me*), 22.1 (CH$_2$), 23.3 (SiCH$_2$CH$_2$), 23.7(SCH$_2$CH$_2$CH$_2$Si), 25.3 (CH$_2$), 25.4 (CH$_2$S), 26.4, 26.5, 28.0, 28.7, 28.8, 28.99 (6 × CH$_2$), 29.02 (3CH$_2$), 29.02 (CH$_2$CH$_2$NMe$_2$), 29.1, 31.3 (2 × CH$_2$), 34.6 (CH$_2$S), 35.1 (CH$_2$), 38.4 (CH$_2$NH), overlapped with DMSO from HSQC 39.15, 39.22 (2 × CH$_2$NH), 39.8 (SCH$_2$CH), 42.4 (N*Me*$_2$), 55.4 (SCH), 56.4 (CH$_2$NMe$_2$), 59.2 (CHCH$_2$S), 61.0 (CHCHS), 68.8, 69.2, 69.5, 69.6 (4 × OCH$_2$), 70.5 (OCH$_2$CH$_2$CH$_2$), 115.5, 115.8, 118.4 (3 × CH$_{Ph}$), 135.8, 136.1 (2 × C$_q$), 158.5 (C$_q$O), 162.7 (CO(NH)$_2$), 165.3, 165.6, 172.1 (3 × CO). ^{29}Si {^{1}H} NMR (79 MHz, dmso-*d*$_6$): δ 4.06. HRMS (ESI+): Calcd. for [$C_{60}H_{113}N_8O_7S_4Si$]$^+$ 1213.7379, found [M-3HCl+H]$^+$ 1213.7380; calcd. for [$C_{60}H_{114}N_8O_7S_4Si$]$^{2+}$ 607.3726, found [M-3HCl+2H]$^{2+}$ 607.3771.

Dendron L3-30. Unsymmetrical dendron **27** with terminal aminogroup (78 mg, 0.054 mmol) was dissolved in dry DMF (2 mL) under inert argon atmosphere. DIPEA (0.075 mL, 0.43 mmol) was slowly added and the mixture stirred at RT for 5 min. Finally, DBCO NHS ester (26 mg, 0.065 mmol) was added in dry DMF (1 mL) and the reaction mixture stirred at RT for 20 h. Afterwards, solvent was reduced to approximately 0.5 mL, DCM (10 mL) added and the obtained suspension filtered through cotton wool. The filtrate was separated by nanofiltration in a mixture of DMC and methanol 1:1 obtaining dendron with free tertiary amino groups (58 mg, 82 %, brown foam). ^{1}H NMR (400 MHz, dmso-*d*$_6$, H-H COSY, HSQC): δ 0.63 – 0.67 (m, 8H, SiC*H*$_2$), 0.83 (t, *J* = 6.9 Hz, 3H, *Me*), 1.16–1.29 (m, 24H, C*H*$_2$), 1.49 – 1.55 (m, 6H, C*H*$_2$CH$_2$S), 1.69 – 1.77 (m, 3H, COC*H*H, OCH$_2$C*H*$_2$CH$_2$), 1.83 (t, *J* = 6.9 Hz, 2H, COC*H*$_2$), 2.15 (dt, *J* = 13.5, 6.5 Hz, 1H, COCH*H*), 2.57 (t, *J* = 7.1 Hz, 6H, SC*H*$_2$), 2.71 (s, 18H, N*Me*$_2$), 2.75 (t, *J* = 8.2 Hz, 6H, C*H*$_2$CH$_2$NMe$_2$), 3.09 (td, *J* = 6.0, 5.6 Hz, 2H, C*H*$_2$NH), 3.14 (t, *J* = 8.2 Hz, 6H, C*H*$_2$NMe$_2$), 3.24 (td, *J* = 6.6, 5.6 Hz, 2H, C*H*$_2$NH), 3.32 (t, *J* = 6.0 Hz, 2H, OC*H*$_2$), 3.42 (td, *J* = 6.0, 5.6 Hz, 2H, C*H*$_2$NH), 3.46 – 3.53 (m, 6H, OC*H*$_2$), 3.59 (d, *J* = 14.0 Hz, 1H, C$_q$NCH*H*), 4.01 (t, *J* = 6.4 Hz, 2H, C$_q$OC*H*$_2$), 5.03 (d, *J* = 14.0 Hz, 1H, C$_q$NCH*H*), 7.28 – 7.62 (m, 10H, C*H*$_{Ph}$), 7.68 (t, *J* = 5.6 Hz, 1H, N*H*), 7.90 (s, 1H, C*H*$_{Ph}$), 8.51, 8.60 (2 × t, *J* = 5.6 Hz, 2 × 1H, 2 × N*H*). ^{13}C {^{1}H} NMR (101 MHz, dmso-*d*$_6$, HSQC, HMBC): δ 7.7 (OCH$_2$CH$_2$CH$_2$), 11.2 (CH$_2$Si), 14.0 (*Me*), 22.1 (CH$_2$), 23.3 (OCH$_2$CH$_2$CH$_2$), 23.7 (SCH$_2$CH$_2$CH$_2$), 24.5, 24.6 (2 × CH$_2$), 25.3 (SCH$_2$CH$_2$NMe$_2$), 26.5, 28.7, 28.8, 28.99, 29.01, (5 × CH$_2$), 29.02 (2CH$_2$), 29.1, 31.3 (2 × CH$_2$), 34.0 (COCH$_2$), 34.9 (COCH$_2$), 35.6 (CH$_2$CH$_2$CH$_2$S), 38.4 (CH$_2$NH), overlapped with DMSO from HSQC

39.2, 39.3 (2 × CH_2NH), 42.4 (NMe_2), 54.8 (C_qNCH_2), 56.2 (CH_2NMe_2), 68.8, 69.1, 69.51, 69.53 (4 × OCH_2), 70.5 (C_qOCH_2), 108.2, 114.3 (C≡C), 115.5 (2CH_{Ph}), 118.3 (CH_{Ph}), 121.4, 122.5 (2 × C_q), 125.1, 126.8, 127.7, 128.0, 128.1, 128.9,129.4, 132.4 (8 × CH_{Ph}), 135.9, 136.3, 148.4, 151.8 (4 × C_q), 158.4, (C_qO), 165.4, 165.7, 171.7, 171.9 (4 × CONH). ^{29}Si NMR (79 MHz, dmso-d_6) δ 3.99. HRMS (ESI+): Calcd. for $[C_{71}H_{116}N_7O_7S_3Si]^+$ 1302.7862, found $[M+H]^+$ 1302.7865; calcd. for $[C_{71}H_{117}N_7O_7S_3Si]^{2+}$ 651.8967, found $[M+2H]^{2+}$ 651.8983.

Dendron with free amino groups (28 mg, 0.021 mmol) was dissolved in DCM (1 mL) and methyl iodide (8.0 µl, 0.13 mmol) was slowly added. The reaction mixture was stirred at RT for 24 h. Afterwards, solvent and the excess of MeI were carefully removed and disposed on rotary evaporator obtaining dendron **L3-30** (27 mg, 95 %, brown viscous substance). ^{1}H NMR (400 MHz, dmso-d_6, H-H COSY, HSQC): δ 0.63 – 0.69 (m, 8H, $SiCH_2$), 0.83 (t, J = 6.9 Hz, 3H, *Me*), 1.17 – 1.27 (m, 22H, CH_2), 1.49 – 1.57 (m, 8H, CH_2CH_2S, CH_2), 1.70 – 1.77 (m, 3H, COCHH, $OCH_2CH_2CH_2$), 1.83 (t, J = 6.9 Hz, 2H, $COCH_2$), 2.15 (dt, J = 13.9, 6.7 Hz, 1H, COCHH), 2.58 (t, J = 7.1 Hz, 6H, SCH_2), 2.78 (s, 27H, N^+Me_3), 2.73 – 2.83 (m, 6H, $CH_2CH_2N^+$), 3.09 – 3.12 (m, 2H, CH_2NH), 3.22 – 3.26 (m, 10H, CH_2NH, CH_2N^+), overlapped with water 3.31 – 3.53 (m, 10H, OCH_2, CH_2NH), 3.59 (d, J = 14.0 Hz, 1H, C_qNCHH), 4.01 (t, J = 6.4 Hz, 2H, C_qOCH_2), 5.03 (d, J = 14.0 Hz, 1H, C_qNCHH), 7.28 – 7.62 (m, 10H, CH_{Ph}), 7.68 (t, J = 5.7 Hz, 1H, NH), 7.90 (s, 1H, CH_{Ph}), 8.52, 8.60 (2 × t, J = 5.6 Hz, 2 × 1H, 2 × NH). ^{13}C {^{1}H} NMR (101 MHz, dmso-d_6, HSQC, HMBC): δ 7.7 ($OCH_2CH_2CH_2$), 11.2 (CH_2Si), 14.0 (*Me*), 22.1 (CH_2), 23.3 ($OCH_2CH_2CH_2$), 23.6 ($SCH_2CH_2CH_2$), 24.5, 24.6 (2 × CH_2), 24.9 ($SCH_2CH_2N^+$), 26.5, 28.7, 28.8, 28.99, 29.01, 29.02, 29.03, 29.05, 31.3 (9 × CH_2), 33.9 ($COCH_2$), 34.9 ($COCH_2$), 35.6 ($CH_2CH_2CH_2S$), 38.3 (CH_2NH), overlapped with DMSO from HSQC 39.2, 39.3 (2 × CH_2NH), 42.2 (N^+Me_3), 54.8 (C_qNCH_2), 55.9 (CH_2Me_3), 68.8, 69.1, 69.51, 69.52 (4 × OCH_2), 70.5 (C_qOCH_2), 108.2, 114.3 (C≡C), 115.5, 115.7, 118.3 (3 × CH_{Ph}), 121.4, 122.5 (2 × C_q), 125.2, 126.8, 127.6, 128.0, 128.1, 128.9, 129.4, 132.3 (8 × CH_{Ph}), 135.9, 136.3, 148.4, 151.8 (4 × C_q) 158.5 (C_qO), 165.4, 165.7, 171.6, 171.9 (4×CONH). ^{29}Si NMR (79 MHz, dmso-d_6) δ 3.99. HRMS (ESI+): Calcd. for $[C_{74}H_{124}IN_7O_7S_3Si]^{2+}$ 736.8764, found $[M-2I]^{2+}$ 736.8640.

Dendron L3-31. Analog of dendron **L3-30** with free amino groups (25 mg, 19 µmol) and D-biotin-PEG3-azide (8.1 mg, 20 µmol) were dissolved in dry DMSO-d_6 under inert argon atmosphere and stirred at RT for 3 h. Afterwards, NMR and HRMS spectra of the crude reaction mixture were measured and confirmed presence of dendron **L3-31**. See **Figure S91**. Dendron **L3-31** was not isolated.

Triallyl(3-chloropropyl)silane. Triallyl(3-chloropropyl)silane was prepared in analogy to a previously published procedure. Magnesium (23.0 g, 947 mmol) and a small amount of iodine were put to dry three-neck flask under inert argon atmosphere with dropping funnel and condenser connected. Iodine was heated by heat gun until iodine vapours appeared. Dry Et_2O (250 mL) was poured to the dry dropping funnel and a small amount (10 mL) was added to magnesium in the flask. Allyl bromide (41.0 mL, 473 mmol) was added to the dropping funnel and a small amount of the solution (10 mL) was also added to magnesium in the flask. The reaction mixture was stirred and heated by heat gun until initiation occurred. The solution of allyl bromide was then added dropwise at such a rate that the reaction mixture only mildly refluxed. After addition of all of the solution, the reaction mixture was stirred and heated to reflux for 90 min. After cooling to RT, titration was performed.

Titration: Next day, menthol (318 mg, 2.03 mmol) and phenanthroline (4 mg) were dissolved in dry THF (6 mL) in dry Schlenk flask under inert argon atmosphere and were titrated by Grignard agent solution (1,92 mL) until the colourless solution turned yellow. Concentration of the Grignard agent solution was determined as 1,06 M.

Trichloro(3-chloropropyl)silane (17.0 g, 80.2 mmol) was dissolved in dry Et_2O (170 mL) in dry three-neck flask with dropping funnel and condenser connected and cooled to 0 °C. 1,06 M Grignard agent solution (250 mL, 265 mmol) was poured to the dry dropping funnel and added dropwise to the cooled and stirred solution of trichloro(3-chloropropyl)silane. After addition of all of the Grignard agent solution, the reaction mixture was stirred at RT for 3 h. The reaction mixture was then poured into 400 mL of ice and the organic phase was separated. The

aqueous phase was extracted (2 × 150 mL Et_2O) and combined organic layers washed (2 × 150 mL H_2O, 2 × 150 mL brine) and dried by anhydrous $MgSO_4$. Solvent was removed on rotary evaporator and the product filtered through filter paper (washed with Et_2O). Solvent was again removed obtaining triallyl(3-chloropropyl)silane (17.9 g, 97 %, yellowish liquid). Spectroscopic data were in agreement with literature [42].

Triallyl(3-iodopropyl)silane. Triallyl(3-chloropropyl)silane (6.13 g, 26.8 mmol) and NaI (16.0 g, 107 mmol) were mixed in dry butanone (40 mL) under inert argon atmosphere and stirred and heated to 50 °C for 60 h. Afterwards, solvent was removed, DCM (40 mL) was added and the mixture filtered through filter paper (washed with DCM). DCM was removed on rotary evaporator obtaining triallyl(3-iodopropyl)silane (8.05 g, 94 %, orange oily liquid). ^1H NMR (400 MHz, $CDCl_3$): δ 0.68 – 0.72 (m, 2H, SiCH_2), 1.60 (dt, *J* = 8.1, 1.2 Hz, 6H, SiCH_2), 1.81 – 1.89 (m, 2H, SiCH_2CH_2), 3.17 (t, *J* = 7.1 Hz, 2H, CH_2I), 4.87 – 4.93 (m, 6H, CH_2CH), 5.78 (ddt, *J* = 16.9, 10.1, 8.1 Hz, 3H, CH_2CH). ^{13}C {^1H} NMR (101 MHz, $CDCl_3$): δ 11.0 (CH_2I), 13.7 (CH_2CH_2Si), 19.6 (SiCH_2CH), 28.5 (CH_2CH_2I), 114.1 (CH_2CH), 134.1 (CH_2CH). ^{29}Si {^1H} NMR (79 MHz, dmso-d_6): δ –1.08.

Monoprotected 2,2′-(ethylenedioxy)bis(ethylamine). Mono protection of 2,2′-(ethylenedioxy)bis(ethylamine) was performed according to published procedure. 2,2′-(ethylenedioxy)bis(ethylamine) (5.0 g, 34 mmol) was dissolved in DCM (340 mL) and cooled to 0 °C. Di-*tert*-butyl dicarbonate (1.1 g, 5.1 mmol) was added in DCM (20 mL) during 4 h using linear dispenser. The reaction mixture was stirred for another 2 h at 0 °C and then at RT for 18 h. Afterwards, the mixture was washed (3 × 300 mL H_2O) and briefly dried by anhydrous Na_2SO_4. Solvent was removed obtaining desired product (1.07 g, 84 %, colourless viscous liquid). Spectroscopic data were in agreement with literature [43].

2. Computer modeling

Computational details

3D computer models of dendrons were created using dendrimer builder, as implemented in the Materials Studio software package from BIOVIA (formerly Accelrys). The RESP technique [44] was used for calculation of dendrimers and doxorubicin atoms partial charges. For this charge parametrization the R.E.D. Server Development [45] was used. The necessary QM calculations (QM structure minimizations, molecular electrostatic potential (MEP) calculations) were done using GAMESS [46,47]. The default, HF/6-31G*, level of theory was used for all charge-related QM calculations and the MEP potential was fitted on Connolly molecular surface. GAFF force field (Generalized Amber Force Field) [48] was used for parameterization of all dendrons. Missing force constants and energy barriers for "Si containing" force field terms were fitted by minimizing the differences between QM and force field based relative energies of 100 configurations (obtained from simulation (using initial parameters) at 310 K) + 100 configurations (obtained from simulation at 450 K) of properly chosen molecular fragment (see figure S2) (i.e. ff parameters that most accurately ensures the following requirement were used: $E^i_{force\text{-}field} = E^i_{quantum} + K$ for all configurations *i* - where $E^i_{force\text{-}field}$ and $E^i_{quantum}$ are force field and QM based energy of molecular configuration *i* and K is constant) – see figure S2 and table S1. Equilibrium values of "Si-containing" bonds and angles were obtained using QM optimization of the given molecular fragment. Average energy error (QM vs MM energy), when fitted "Si-containing" parameters were used, 0.9959 kcal / mol and 1.3766 kcal / mol on conformations (of the molecular fragment – see Figure. S2) generated at 310 K and 450 K, respectively. QM energies were calculated at MP2/HF/6-31G** level of theory using GAMESS and fitting was accomplished using *paramfit* routine from AMBER software [49]. Slightly adjusted van der Waals parameters for Si atoms from MM3 force field were used in this study [50] (see table S1). Dendron molecular systems (300 dendrons in each system, randomly placed into sphere with diameter ca 170 Å, and minimal intermolecular distance requirement 4 Å.), prepared using *Packmol* [51], were solvated in explicit water (TIP3P model) [52] – see figure **SM1**. Diameter of truncated octahedron simulation box was ca 240 Å. Proper number of Cl- ions was added to ensure the neutrality of the simulation box. First, the systems were minimized (5000 steps with 2 kcal/(mol Å2) restraint + 50000 without restraint), heated (200 ps NVT) to 295 K and equilibrated using 400 ns long

molecular dynamics simulations (NPT, T = 295 K, P = 0.1 MPa). The first 0.5 ns with restrained solute. Hydrogens were constrained with the SHAKE algorithm to allow 2 fs time step [53] and Langevin thermostat with collision frequency 2 ps^{-1} was used for all MD runs [54]. The pressure relaxation time for weak-coupling barostat was 2 ps. Particle mesh Ewald method (PME) was used to treat long range electrostatic interactions under periodic conditions with a direct space cutoff of 10 Angstroms. The same cutoff was used for van der Waals interactions. The *pmemd.cuda* module from Amber18 package was used for all simulation steps [55]. UCSF Chimera software was used for all visualizations [56].

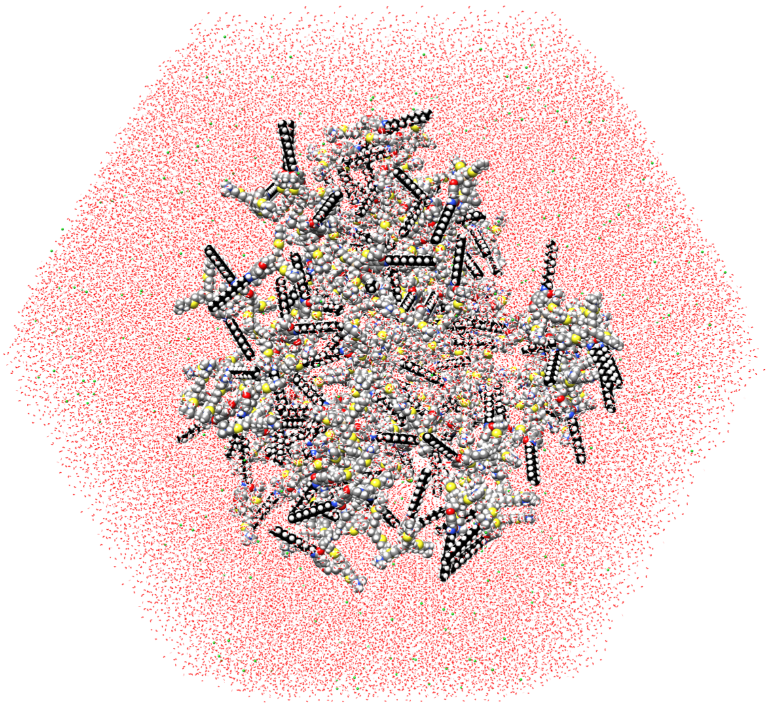

Figure S1: Example of the initial configuration of solvated 300 dendrons system. This is specifically a cross-section of the solvated dendron system AE 1.38. Dendrons were randomly placed into sphere with diameter 170 Å, with minimal intermolecular distance requirement 4 Å. Diameter of truncated octahedron simulation box is ca 240 Å. Carbons belonging to aliphatic chains are highlighted in black. Colors: C – grey/black; O – red; H – white; Si – beige; N – blue; S – yellow, Cl- anions - green.

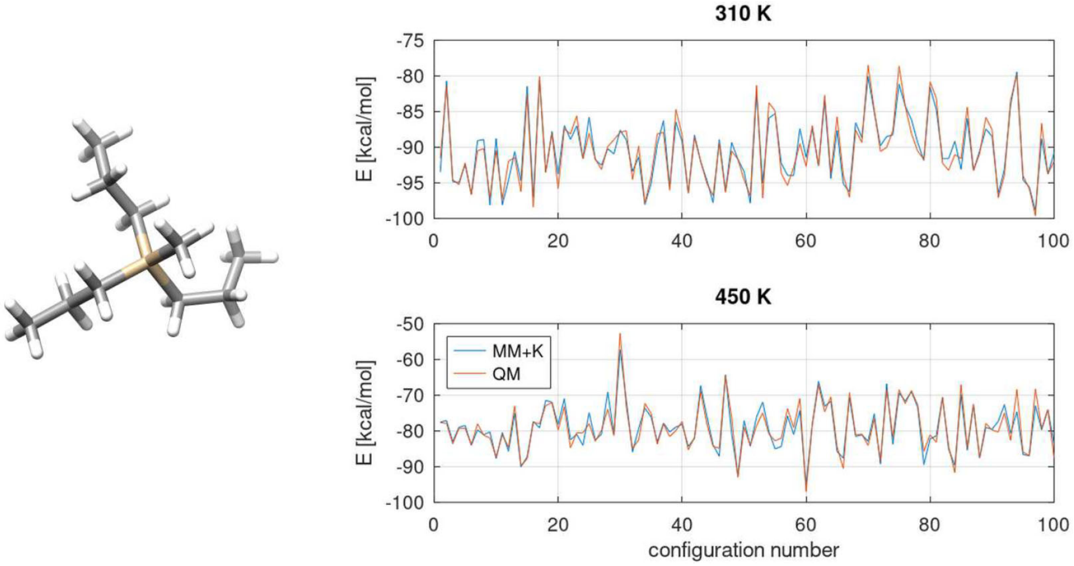

Figure S2: Molecular fragment which was used for Si-ff parameters fitting (left), Colors: C – grey; H – white; Si – beige. Reduced energies "MM + K" (force field energy), "QM" (Quantum mechanics energy) of the given molecular fragment configurations after fitting (right).

Table S1: "Si containing" force field parameters. Used GAFF and added (Si) atom types: c3 - Sp3 carbon, hc - H bonded to aliphatic carbon, Si - Sp3 silicon.

				**energy contribution formula**
**Bond type**	k_l [kcal mol^{-1} Å^{-2}]	l_0 [Å]		$k_l(l-l_0)^2$
c3-Si	211.6344	1.8985		
**Bond angle type**	k_θ [kcal mol^{-1} rad^{-2}]	θ_0 [degrees][1)]		$k_\theta(\theta-\theta_0)^2$
hc-c3-Si	35.0116	109.4977		
c3-c3-Si	53.6369	114.9343		
c3-Si-c3	48.9606	109.4623		
**Dihedral angle type**	$\frac{V_i}{2}$ [kcal mol^{-1}]	φ_0[degrees]	n_i	$\sum_i \frac{V_i}{2}(1+\cos(n_i\varphi-\varphi_0))$
hc-c3-c3-Si	0.0338	0.0000	3.0000	*i=1*
c3-Si-c3-hc	0.0639	0.0000	3.0000	*i=1*
c3-c3-c3-Si	0.1042	180.0000	2.0000	*i=1*
c3-c3-c3-Si	0.1450	0.0000	3.0000	*i=2*
c3-c3-Si-c3	0.0917	0.0000	3.0000	*i=1*
**Lennard-Jones potential parameters for "Si" atom**				$\varepsilon\left(\left(\frac{R_{min}}{r}\right)^{12}-2\left(\frac{R_{min}}{r}\right)^{6}\right)$
R_{min} [Å]	ε [kcal mol^{-1}]			
4.6239	0.1557			
**All parameters written in Amber FRCMOD file format**				

MASS
Si 28.08600

BOND
c3-Si 211.6344 1.8985

ANGL
hc-c3-Si 35.0116 109.4977
c3-c3-Si 53.6369 114.9343

c3-Si-c3	48.9606	109.4623	
DIHE			
hc-c3-c3-Si	0.0338	0.0000	3.0000
c3-Si-c3-hc	0.0639	0.0000	3.0000
c3-c3-c3-Si	0.1042	180.0000	-2.0000 [2]
c3-c3-c3-Si	0.1450	0.0000	3.0000
c3-c3-Si-c3	0.0917	0.0000	3.0000
NONBON			
Si	2.31195 [3]	0.1557	

1) For the energy calculation the degrees are converted to radians.
2) Negative value of the periodicity is here just indication, that there are more terms for this dihedral. The last term is recognized by its positive periodicity value.
3) In FRCMOD file $R_{min}/2$ is used.

3. NMR Spectra of the Compounds

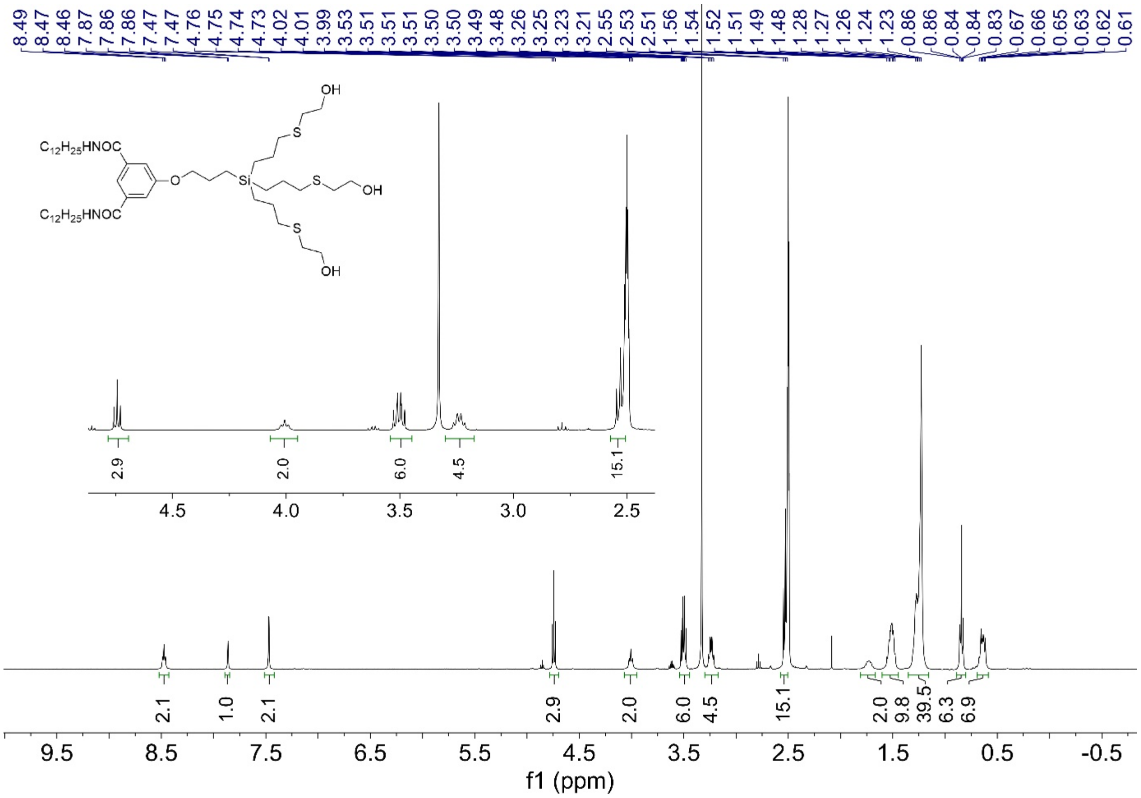

Figure S3. ^{1}H NMR (400 MHz, DMSO-d_6) of **L1-3**

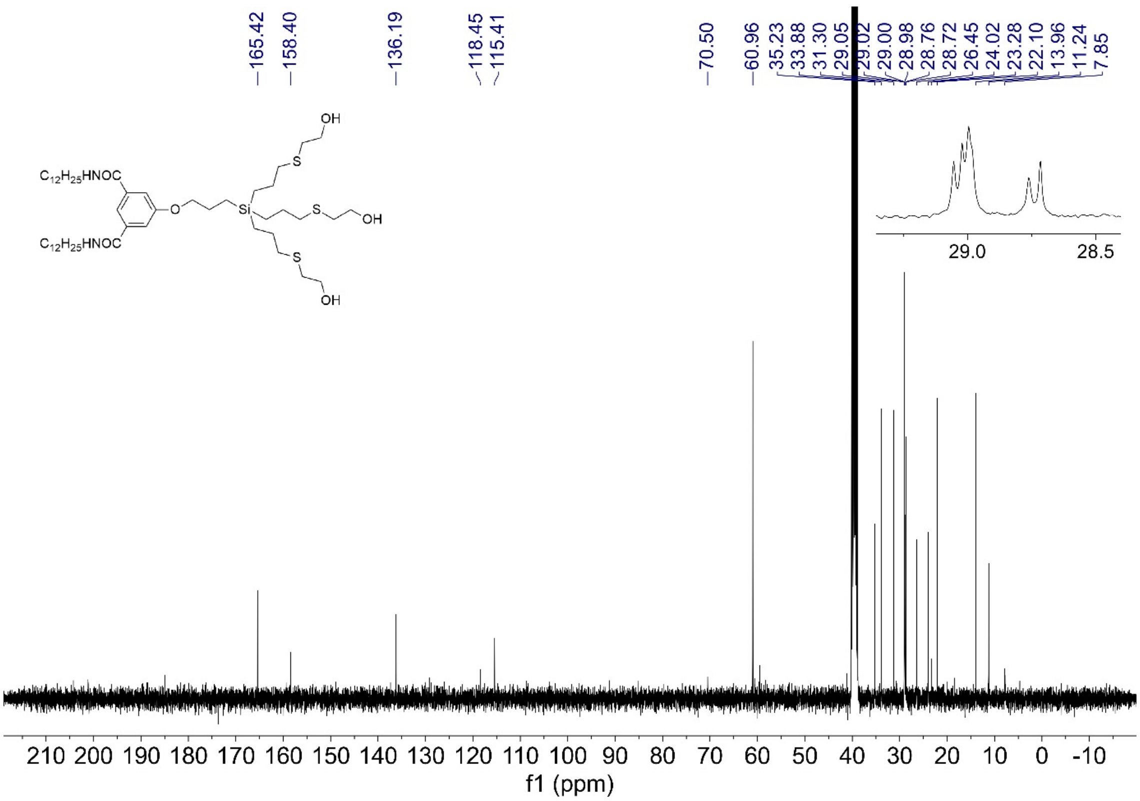

Figure S4. ^{13}C NMR (400 MHz, DMSO-d_6) of **L1-3**

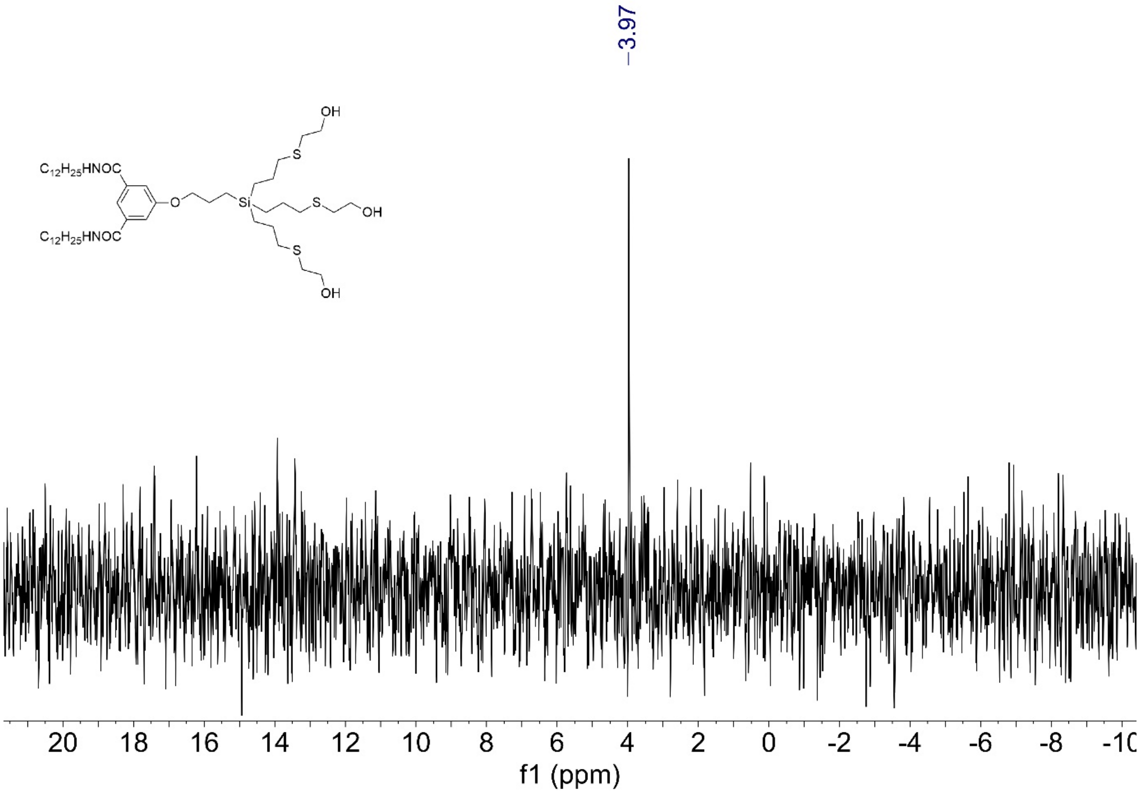

Figure S5. ^{29}Si NMR (400 MHz, DMSO-d_6) of **L1-3**

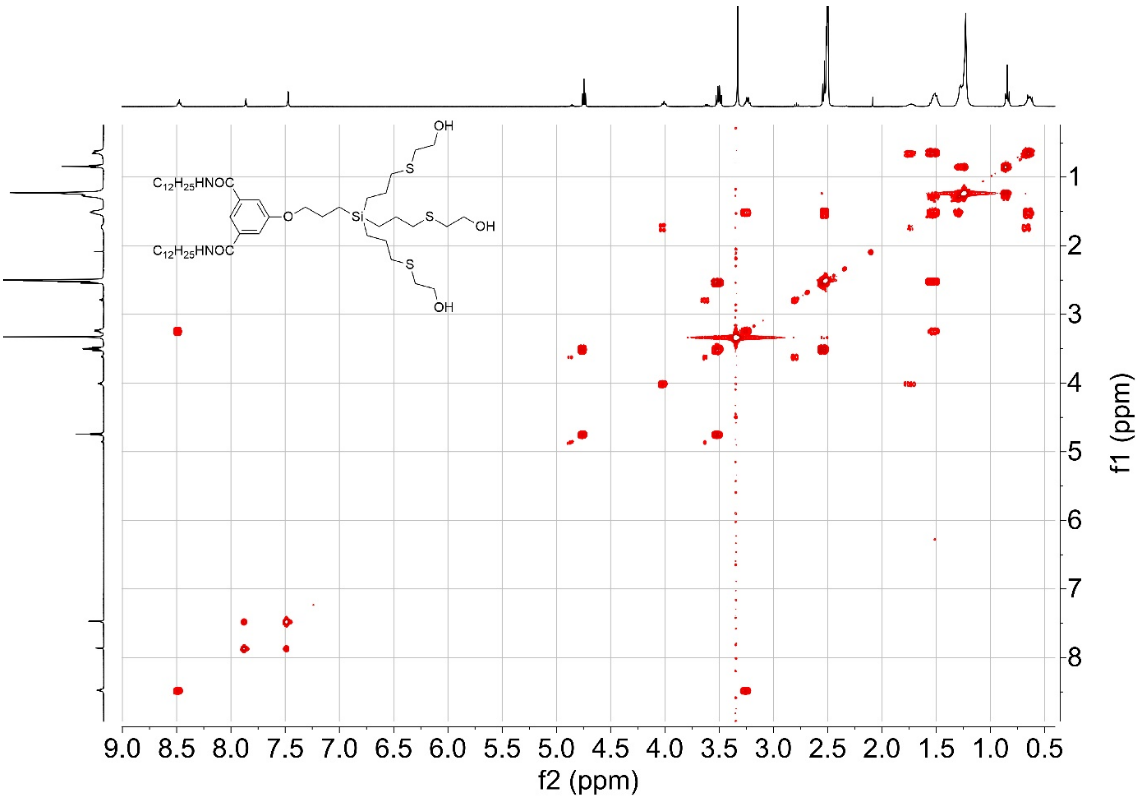

Figure S6. COSY NMR (400 MHz, DMSO-d_6) of **L1-3**

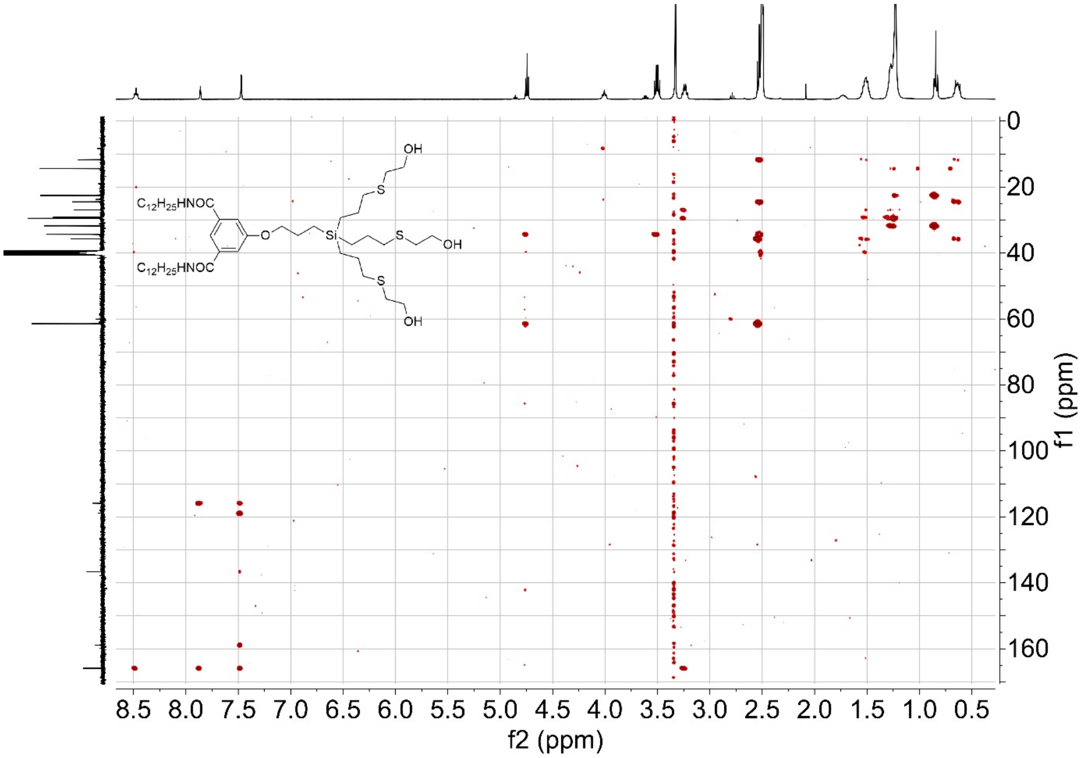

Figure S7. HMBC NMR (400 MHz, DMSO-d_6) of **L1-3**

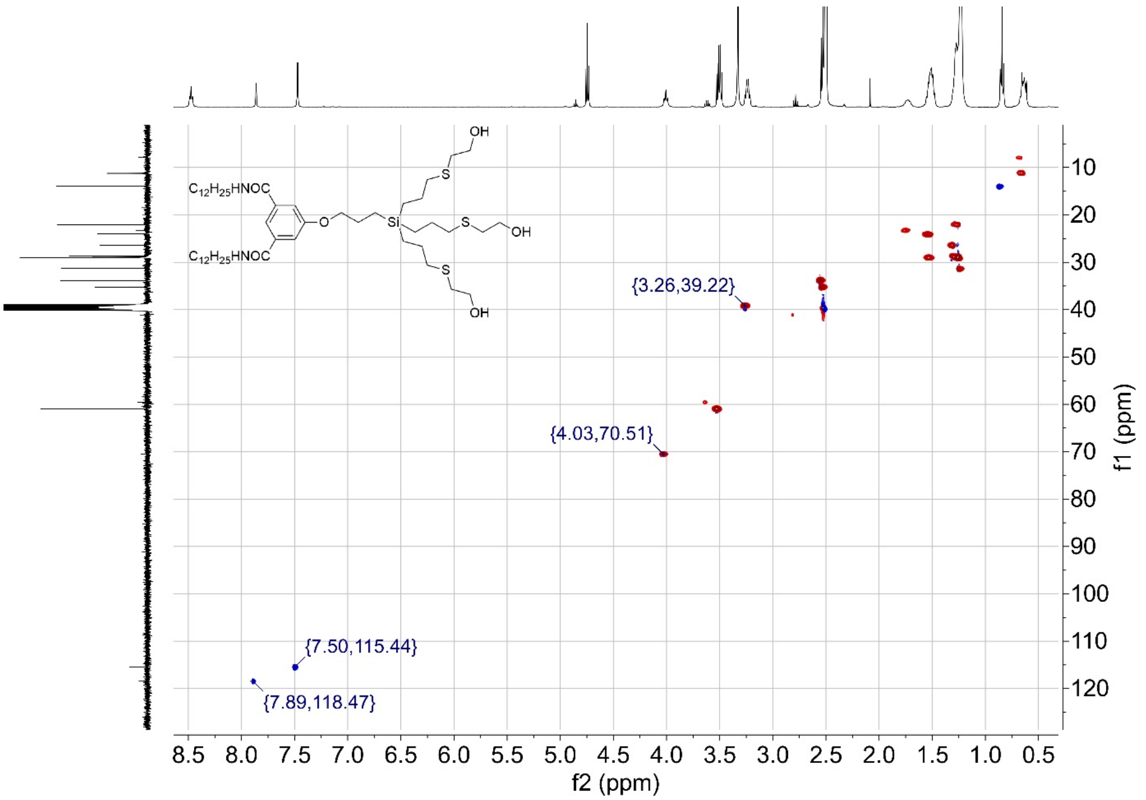

Figure S8. HSQC NMR (400 MHz, DMSO-d_6) of **L1-3**

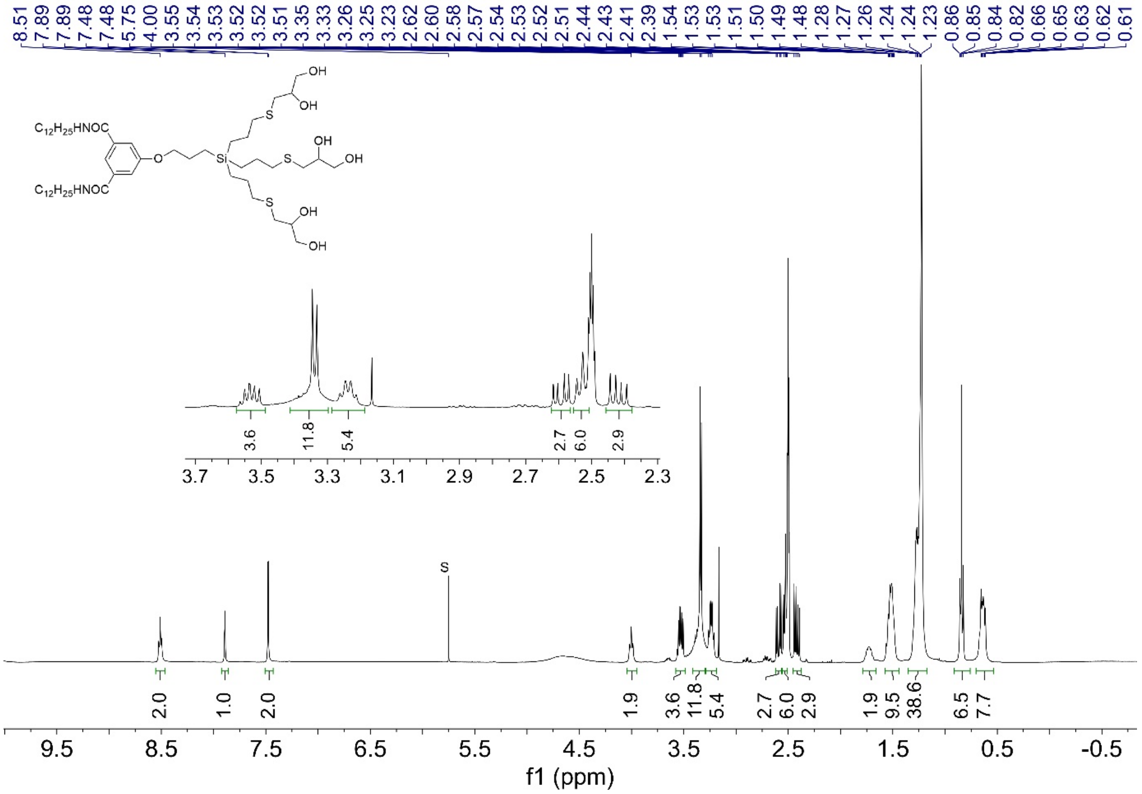

Figure S9. ^{1}H NMR (400 MHz, DMSO-d_6) of **L1-4**

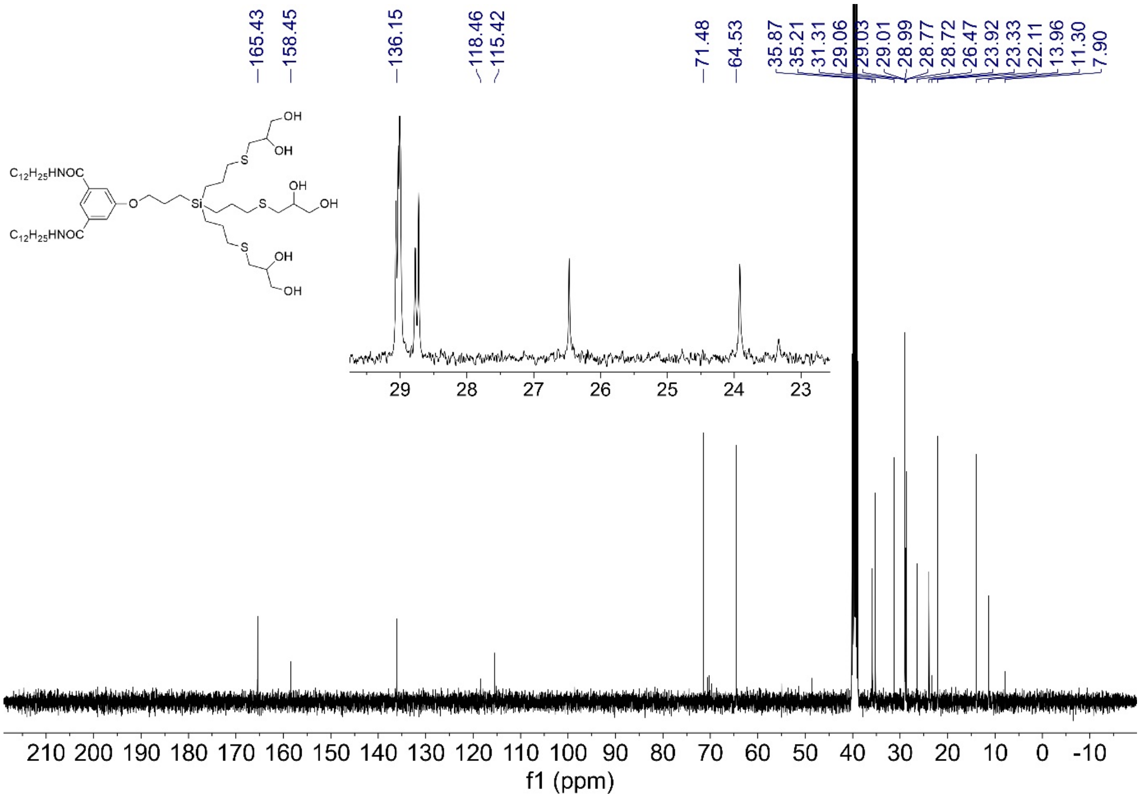

Figure S10. ^{13}C NMR (400 MHz, DMSO-*d*$_6$) of **L1-4**

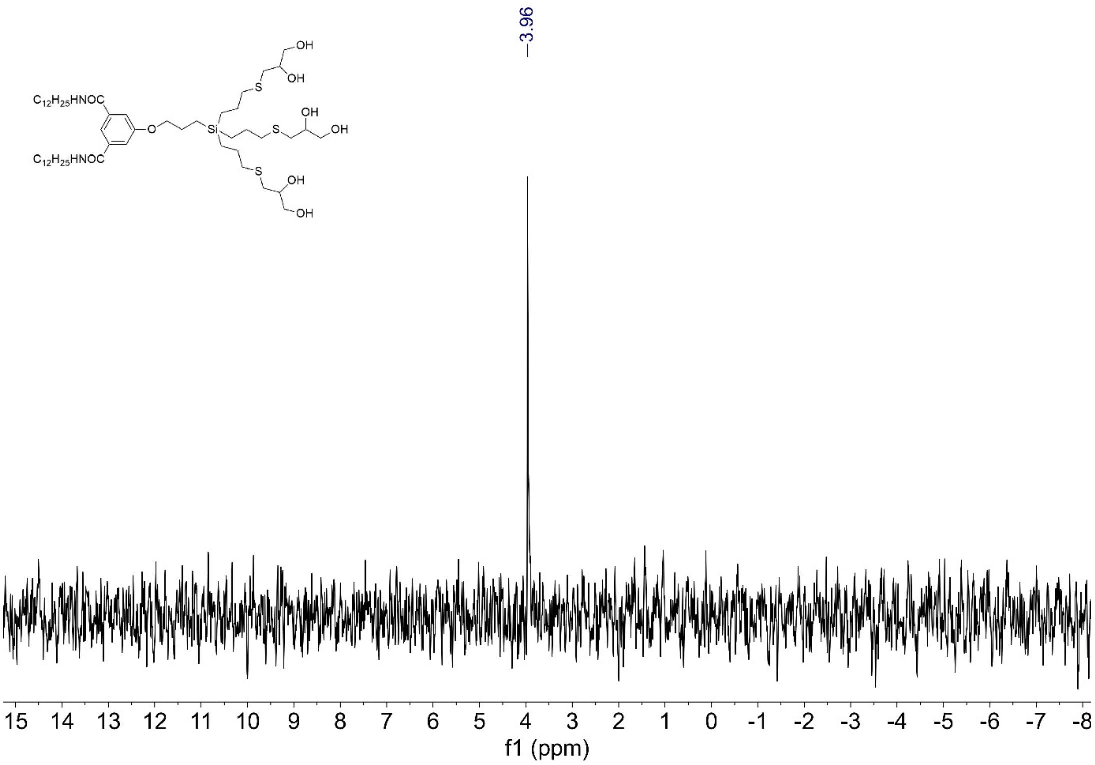

Figure S11. ^{29}Si NMR (400 MHz, DMSO-*d*$_6$) of **L1-4**

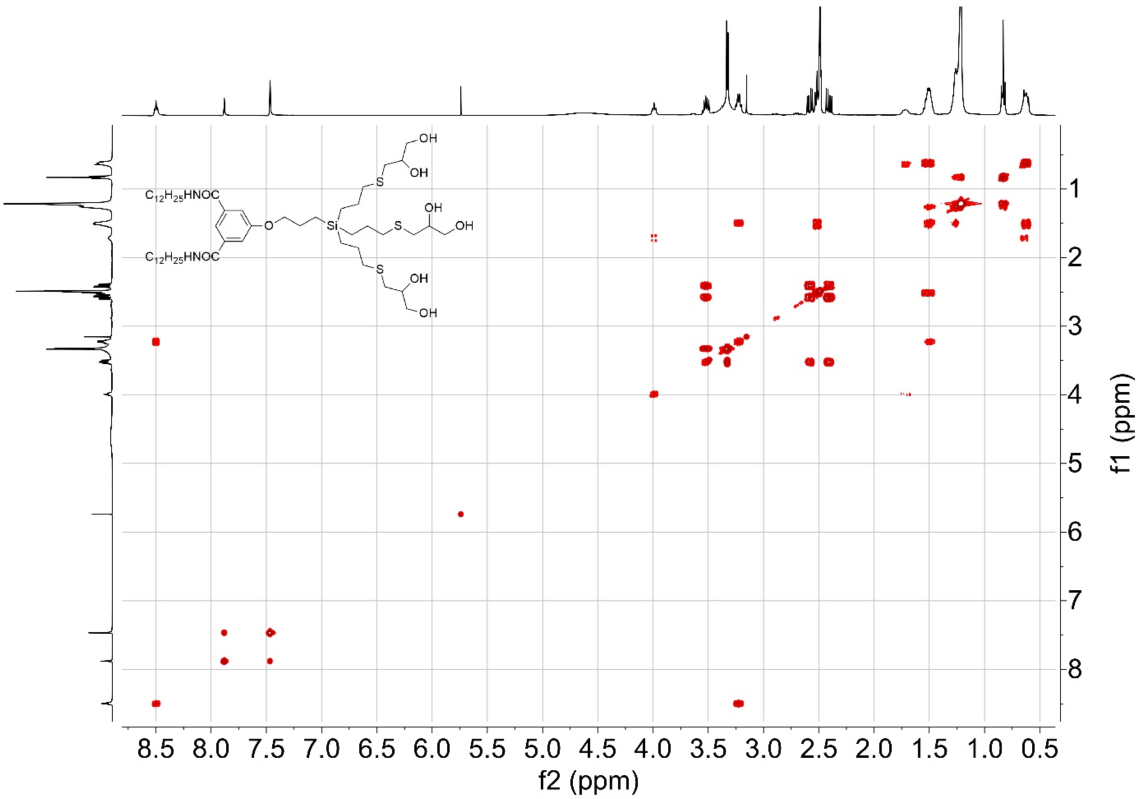

Figure S12. COSY NMR (400 MHz, DMSO-d_6) of **L1-4**

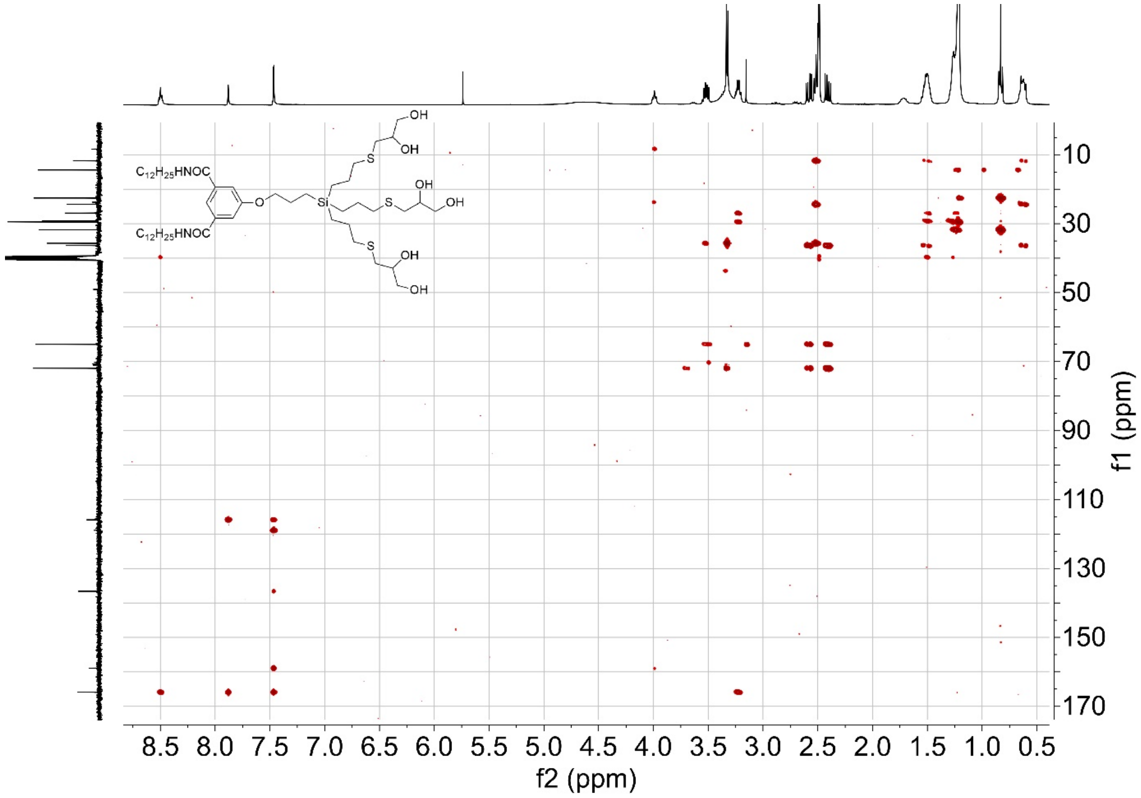

Figure S13. HMBC NMR (400 MHz, DMSO-d_6) of **L1-4**

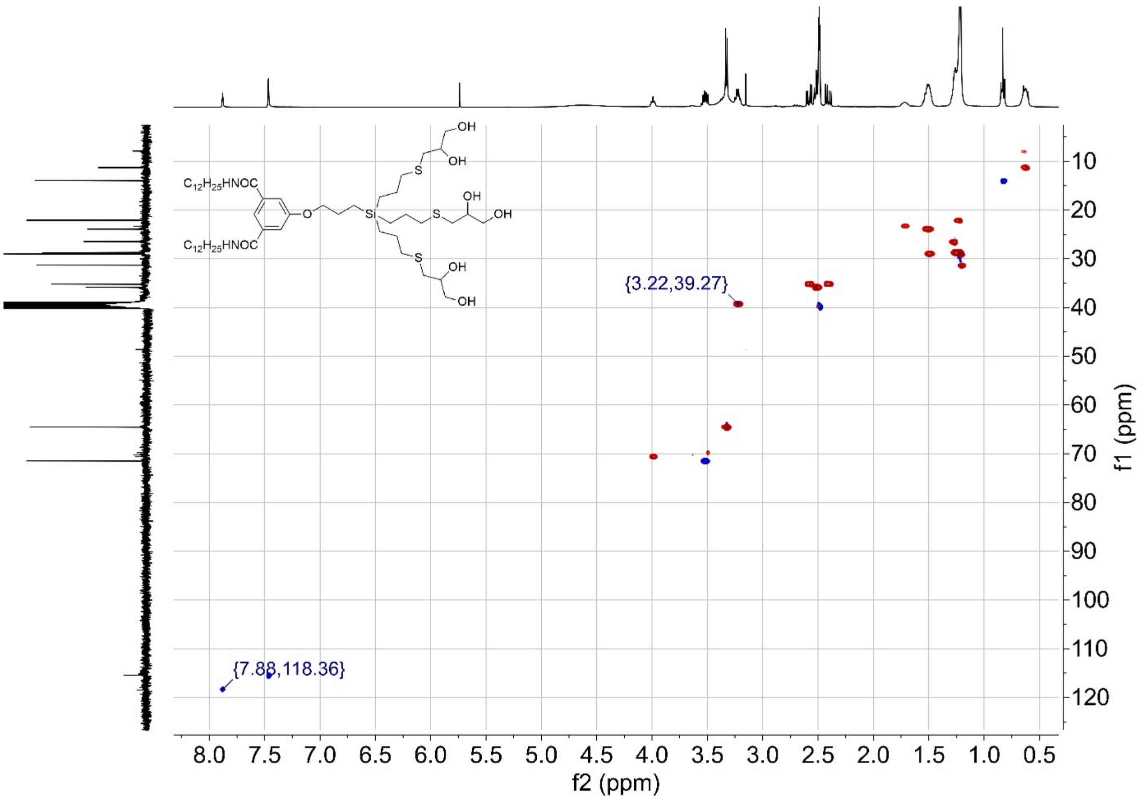

Figure S14. HSQC NMR (400 MHz, DMSO-d_6) of **L1-4**

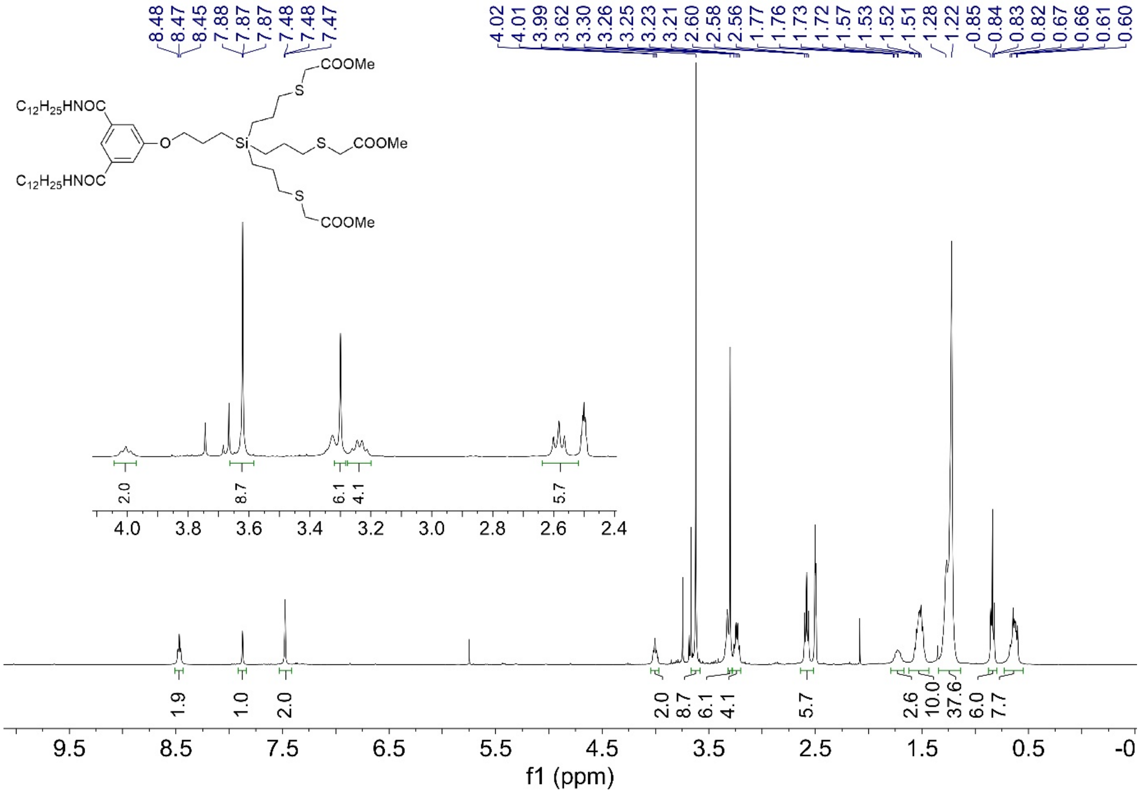

Figure S15. ^{1}H NMR (400 MHz, DMSO-d_6) of **L1-5**

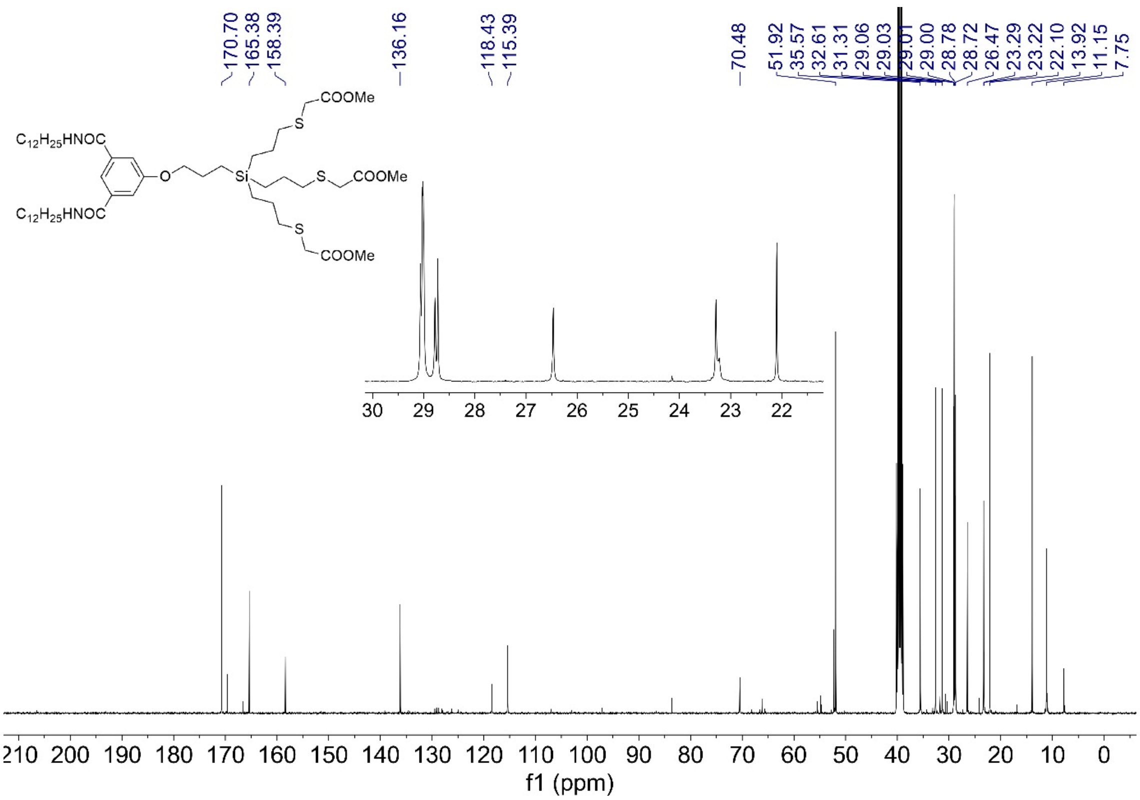

Figure S16. ^{13}C NMR (400 MHz, DMSO-d_6) of **L1-5**

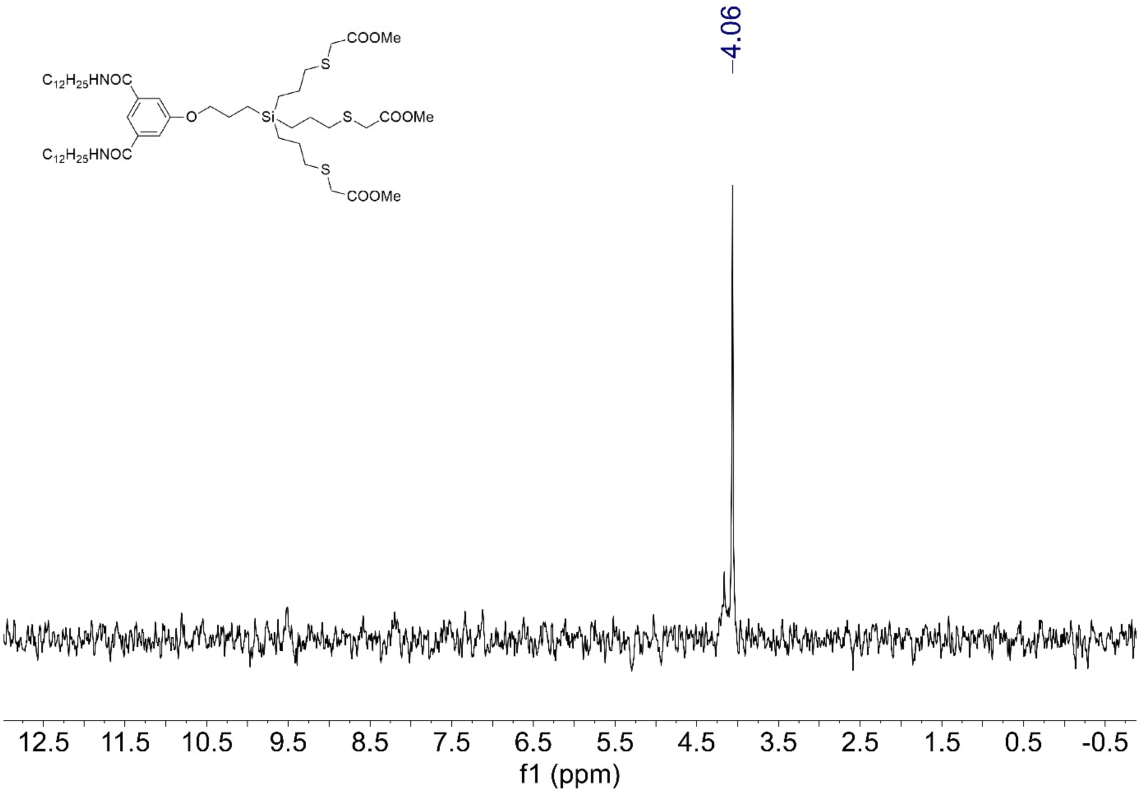

Figure S17. ^{29}Si NMR (400 MHz, DMSO-d_6) of **L1-5**

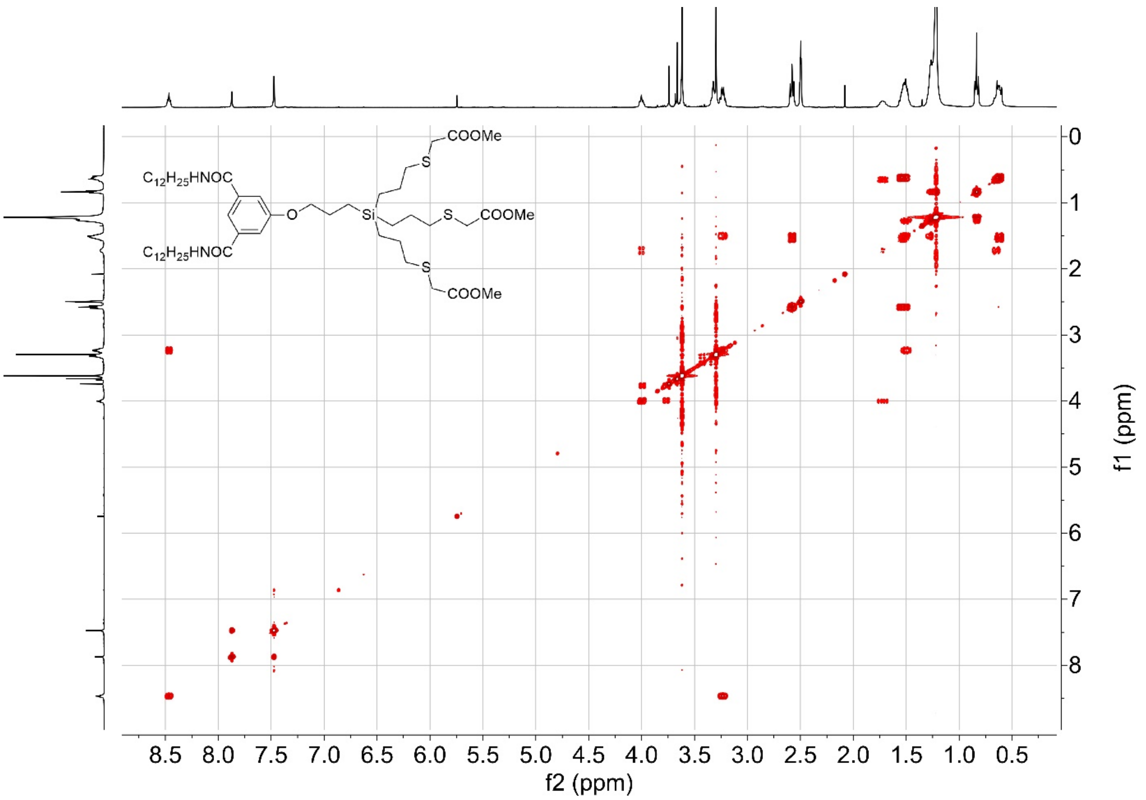

Figure S18. COSY NMR (400 MHz, DMSO-d_6) of **L1-5**

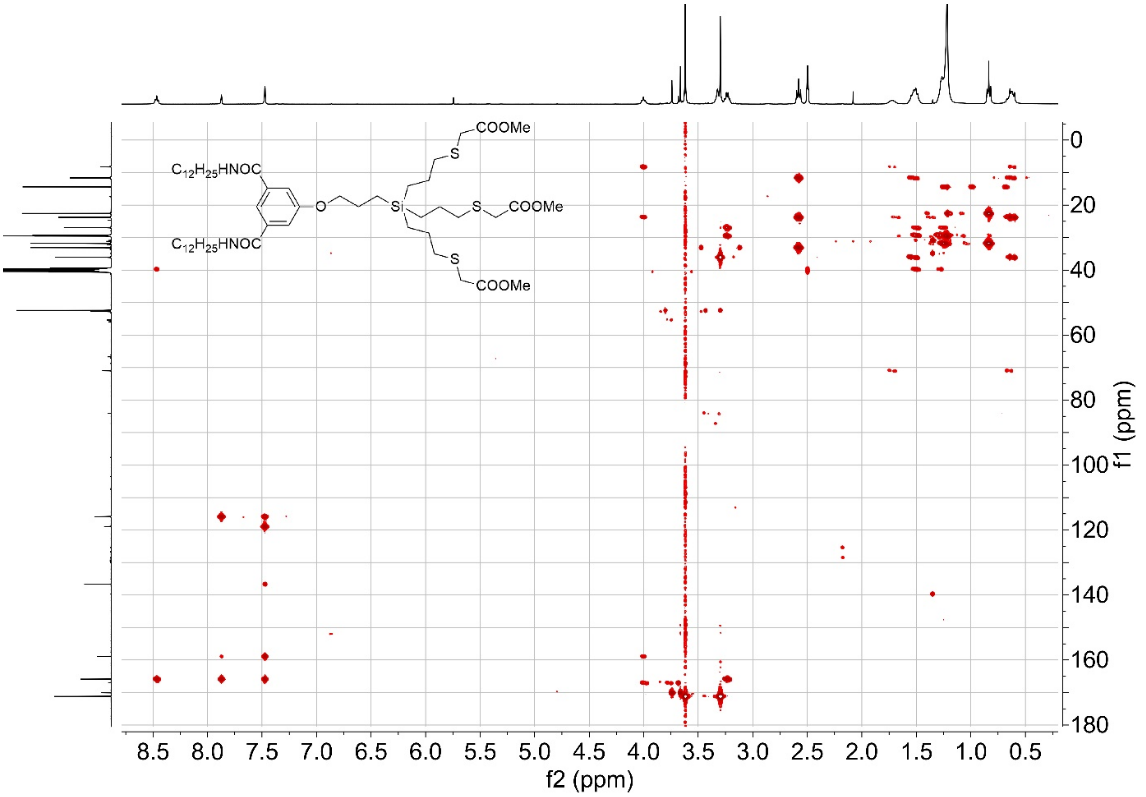

Figure S19. HMBC NMR (400 MHz, DMSO-d_6) of **L1-5**

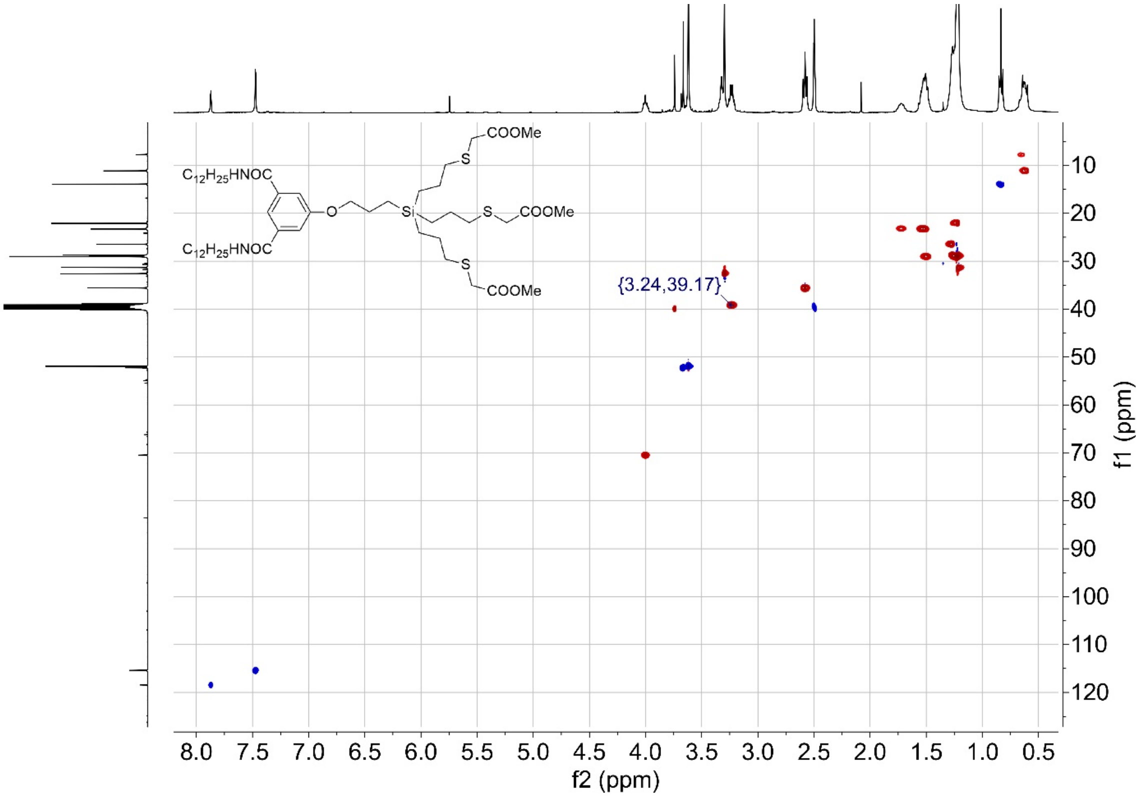

Figure S20. HSQC NMR (400 MHz, DMSO-d_6) of **L1-5**

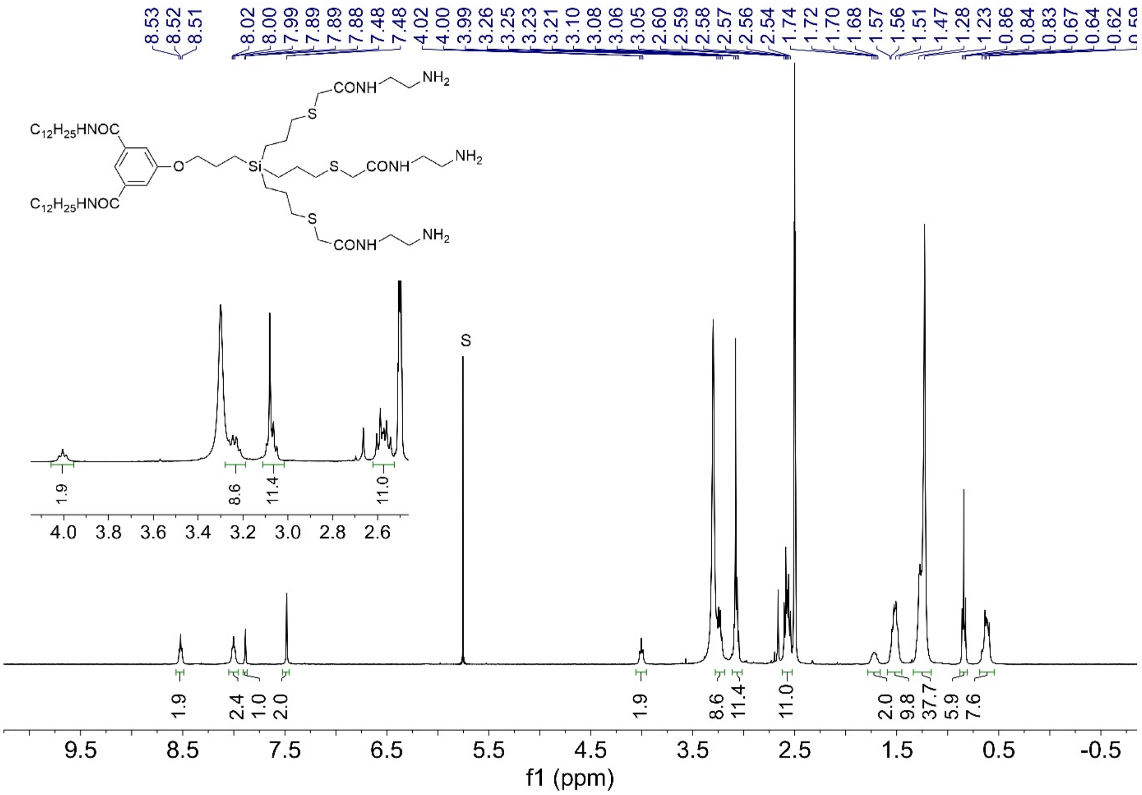

Figure S21. ^{1}H NMR (400 MHz, DMSO-d_6) of **L1-6**

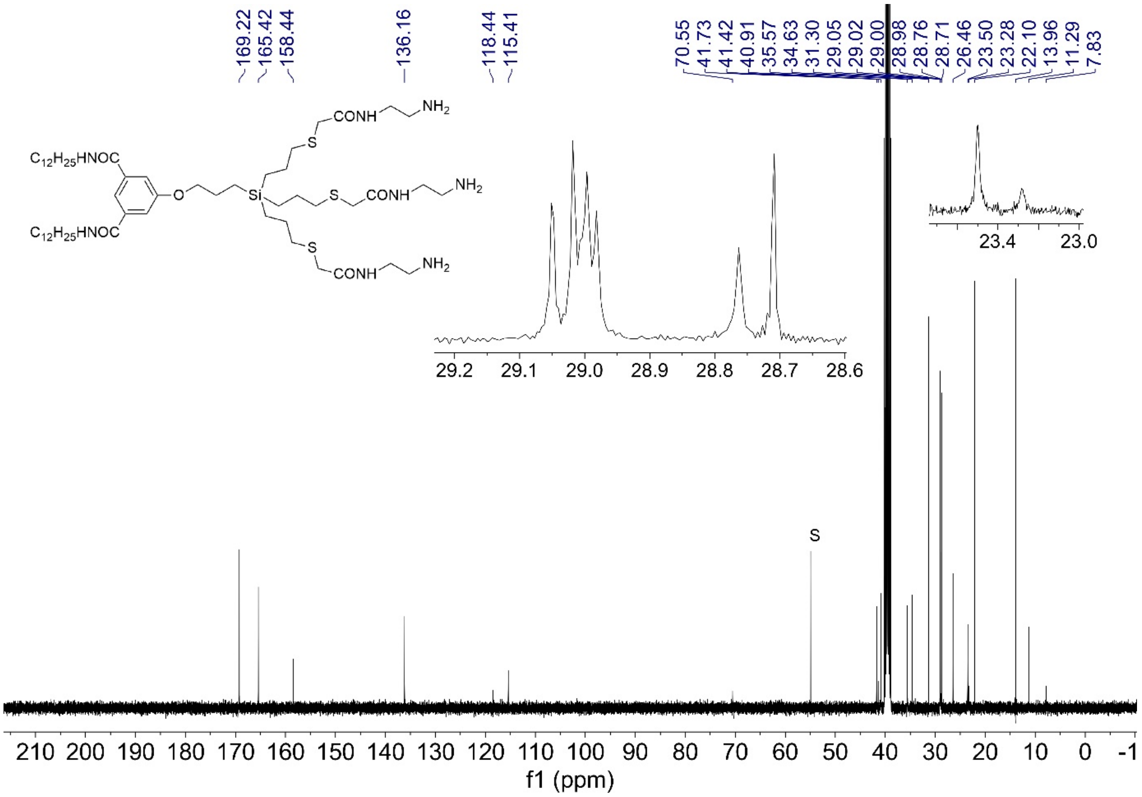

Figure S22. ^{13}C NMR (400 MHz, DMSO-d_6) of **L1-6**

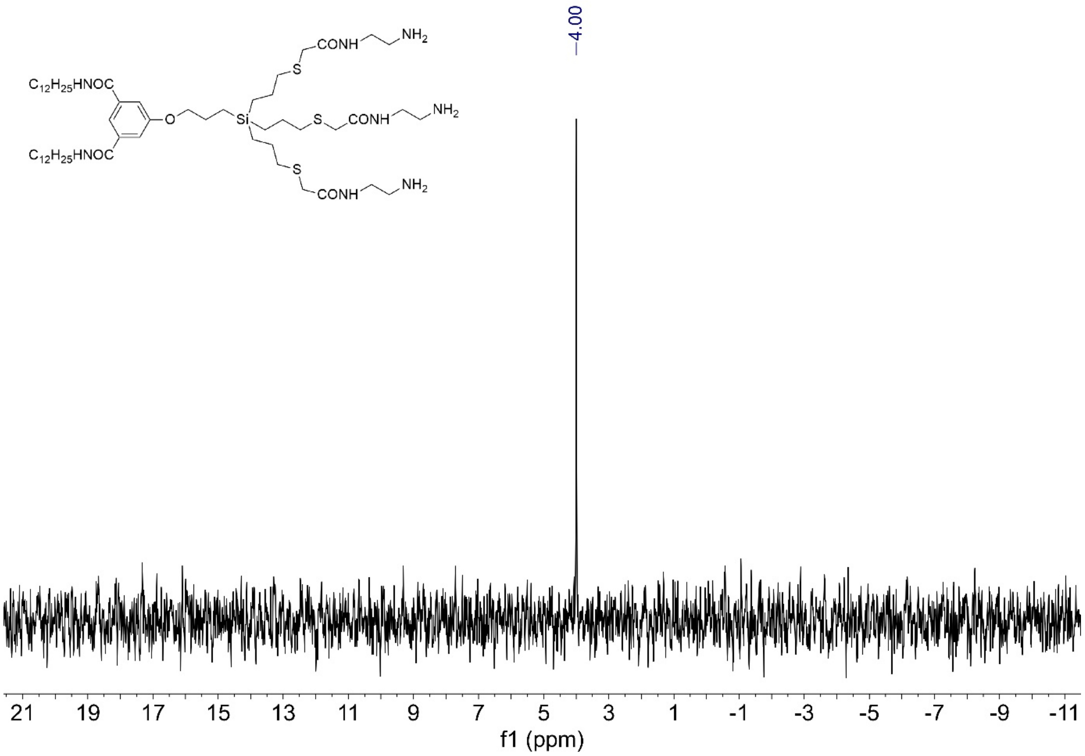

Figure S23. ^{29}Si NMR (400 MHz, DMSO-d_6) of **L1-6**

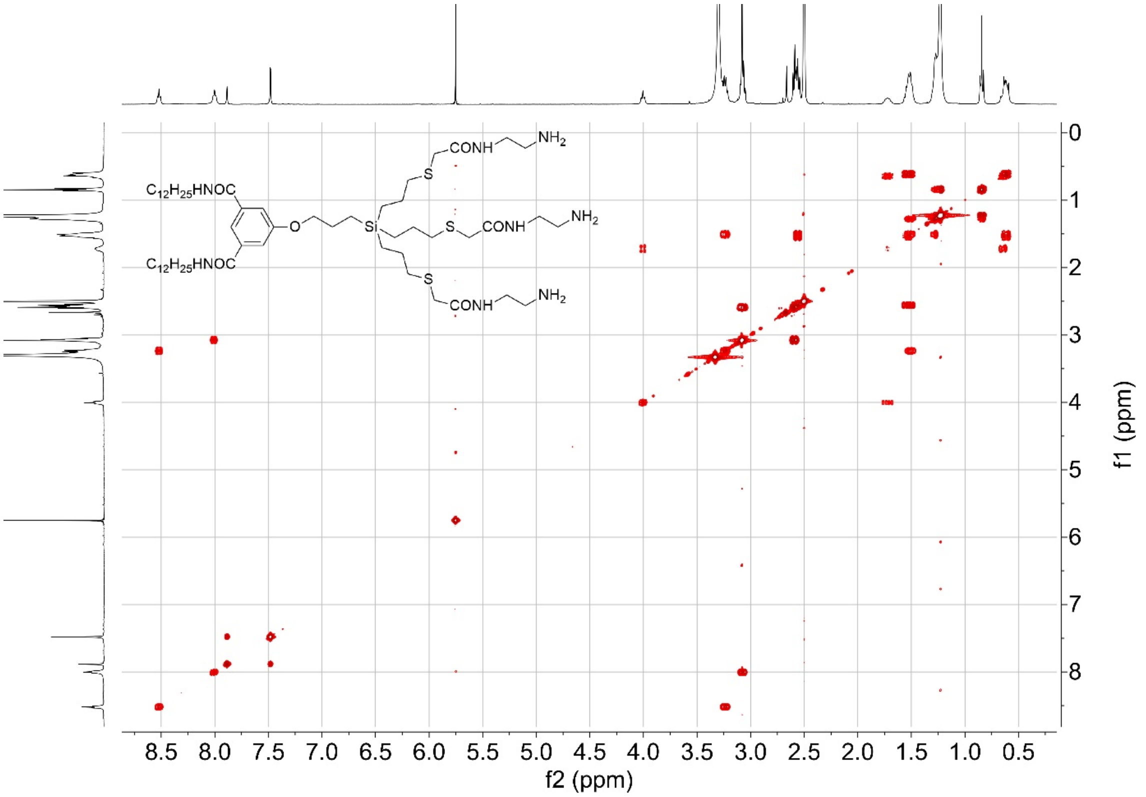

Figure S24. COSY NMR (400 MHz, DMSO-d_6) of **L1-6**

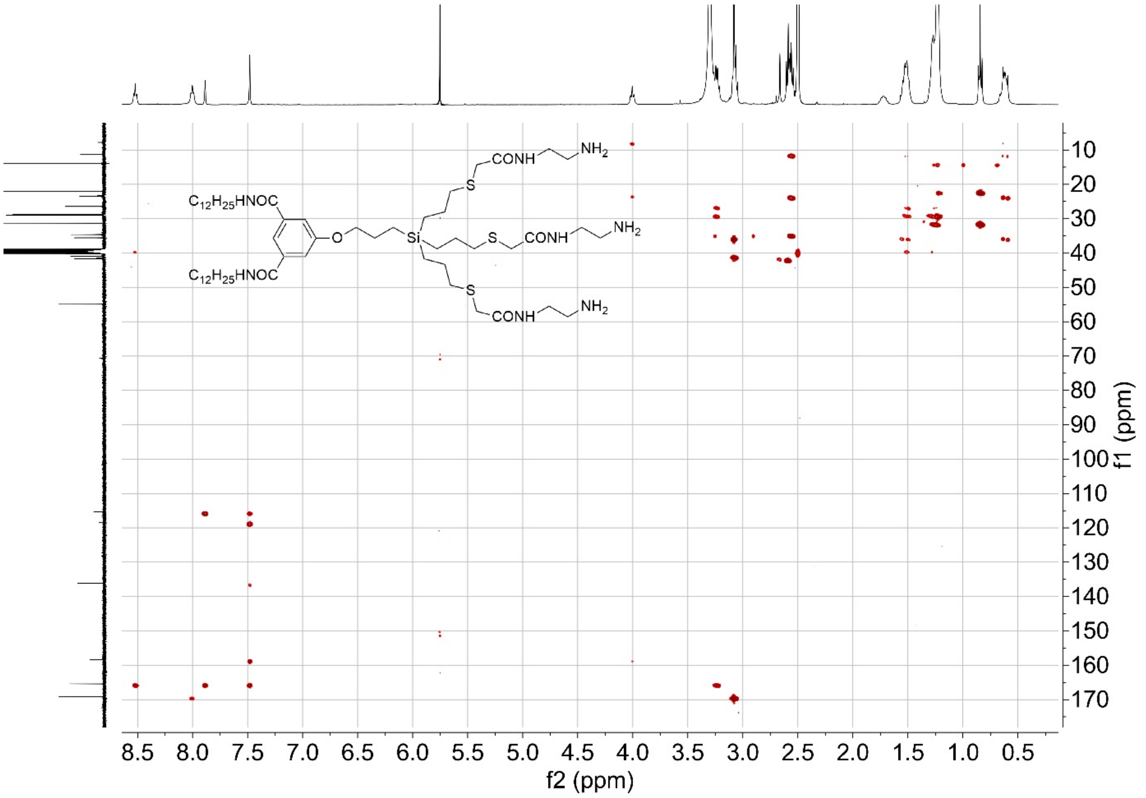

Figure S25. HMBC NMR (400 MHz, DMSO-d_6) of **L1-6**

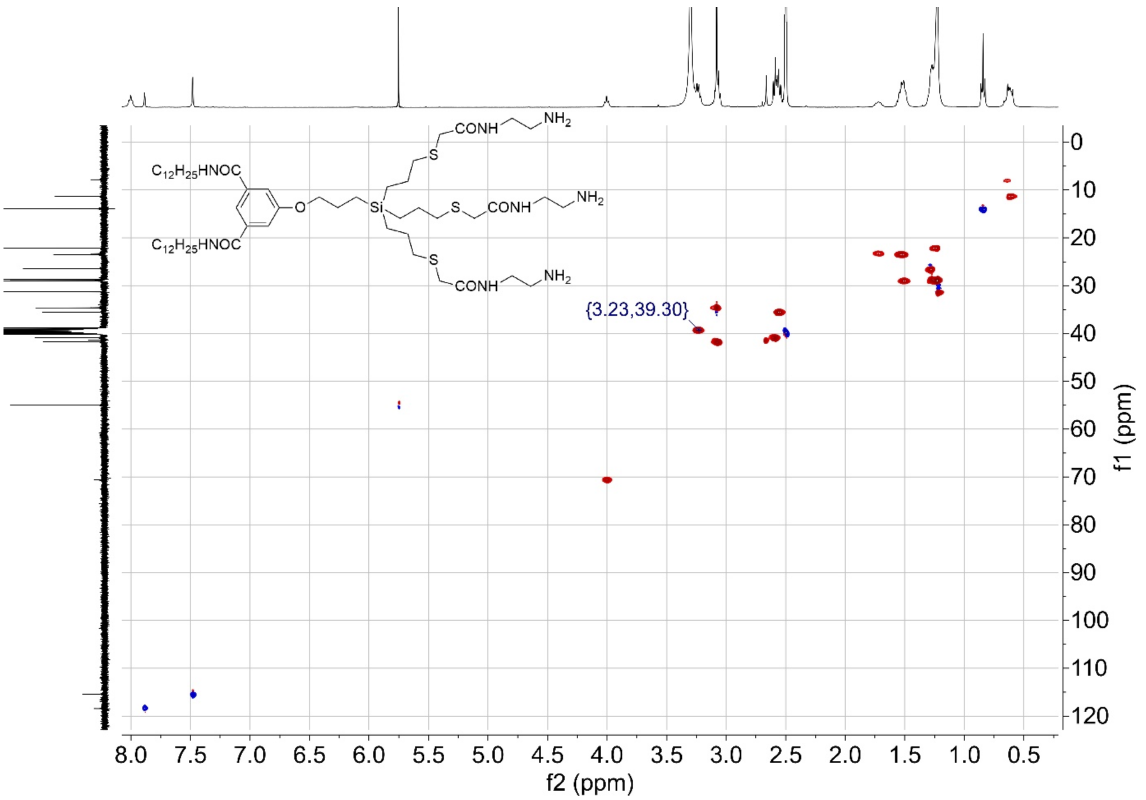

Figure S26. HSQC NMR (400 MHz, DMSO-d_6) of **L1-6**

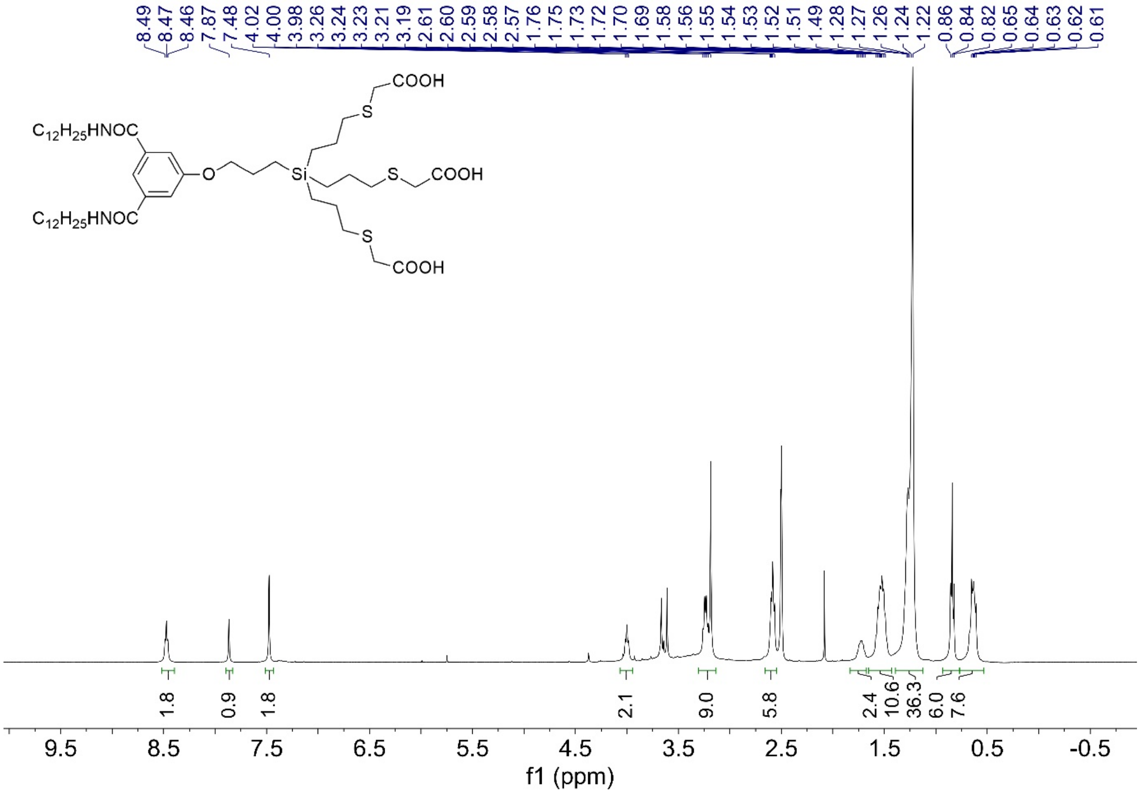

Figure S27. ^{1}H NMR (400 MHz, DMSO-d_6) of **L1-7**

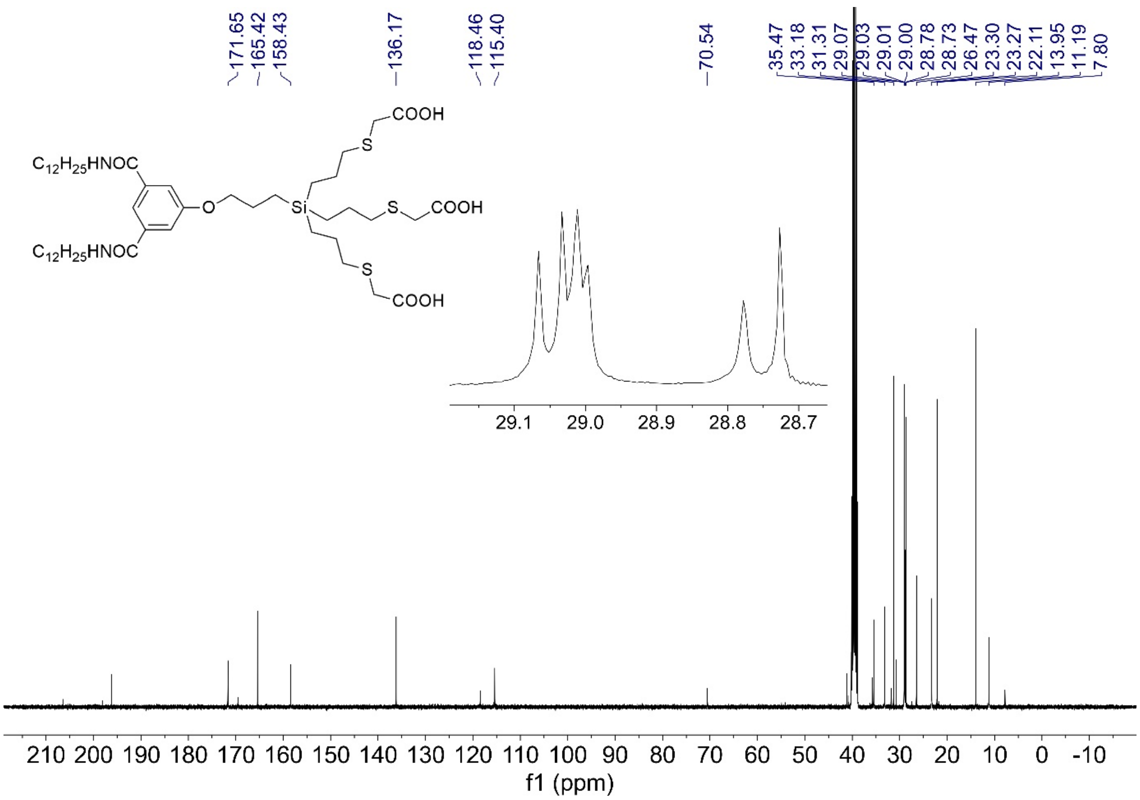

Figure S28. ^{13}C NMR (400 MHz, DMSO-d_6) of **L1-7**

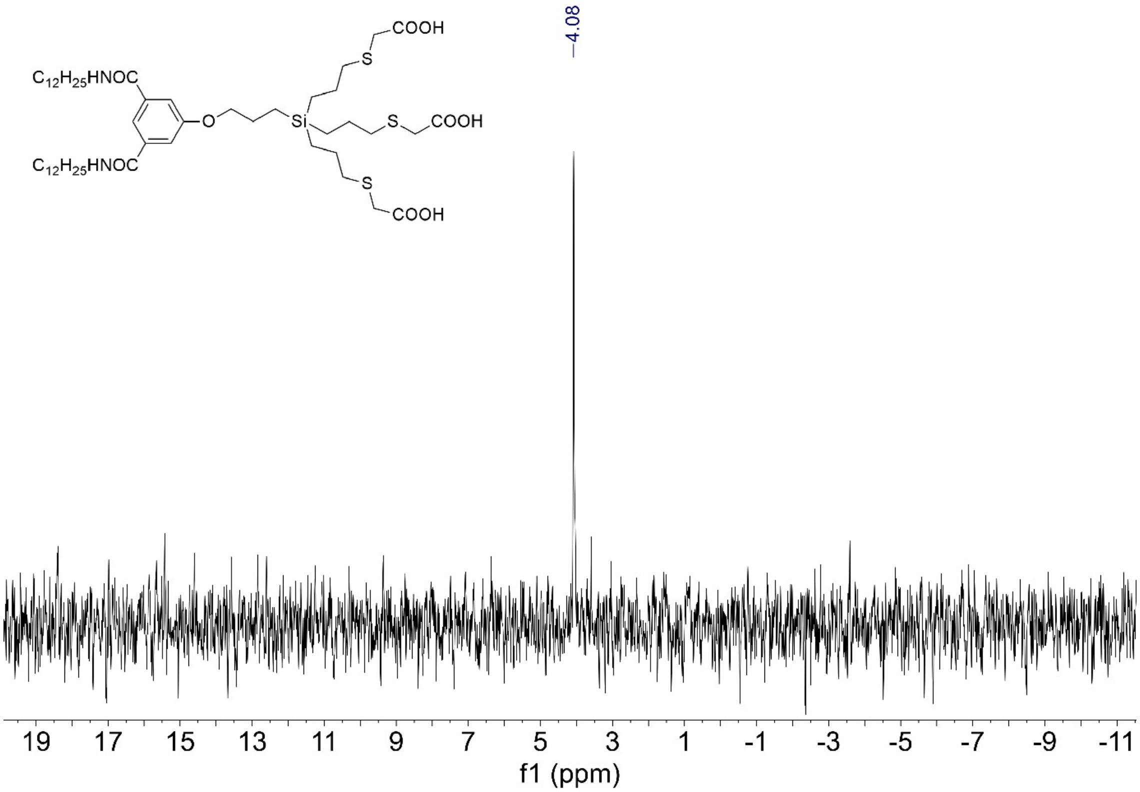

Figure S29. ^{29}Si NMR (400 MHz, DMSO-d_6) of **L1-7**

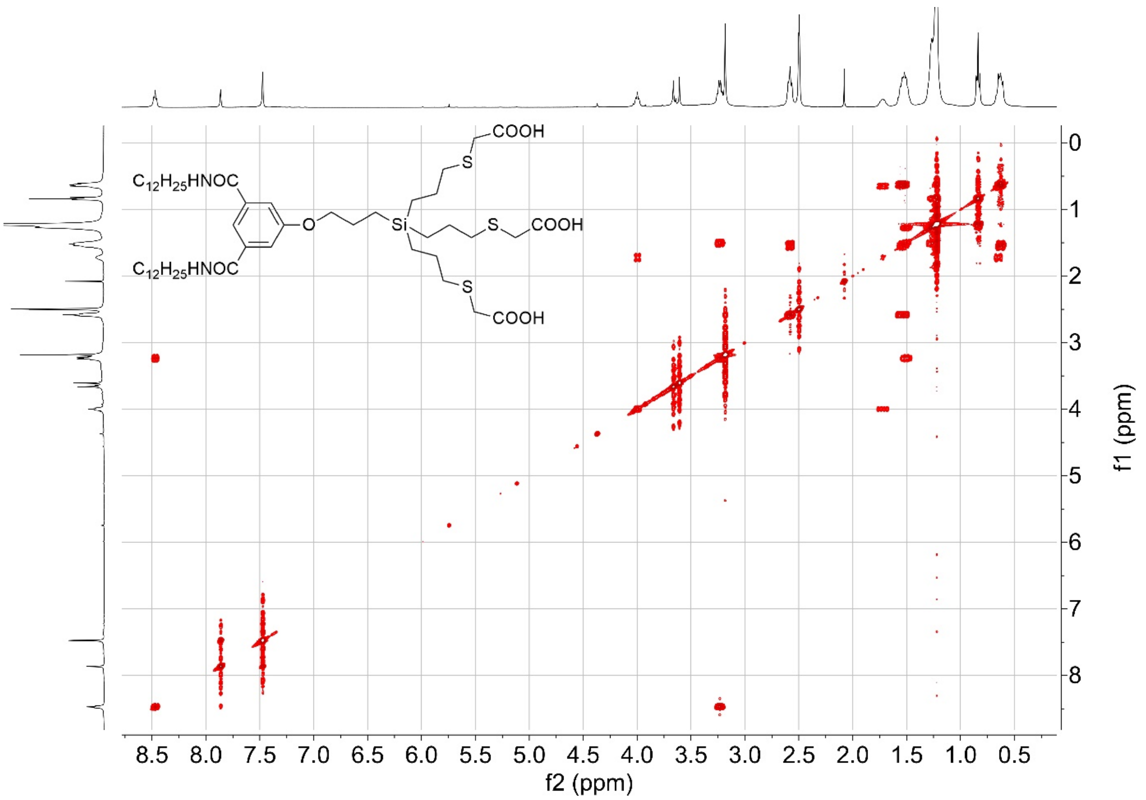

Figure S30. COSY NMR (400 MHz, DMSO-*d*6) of **L1-7**

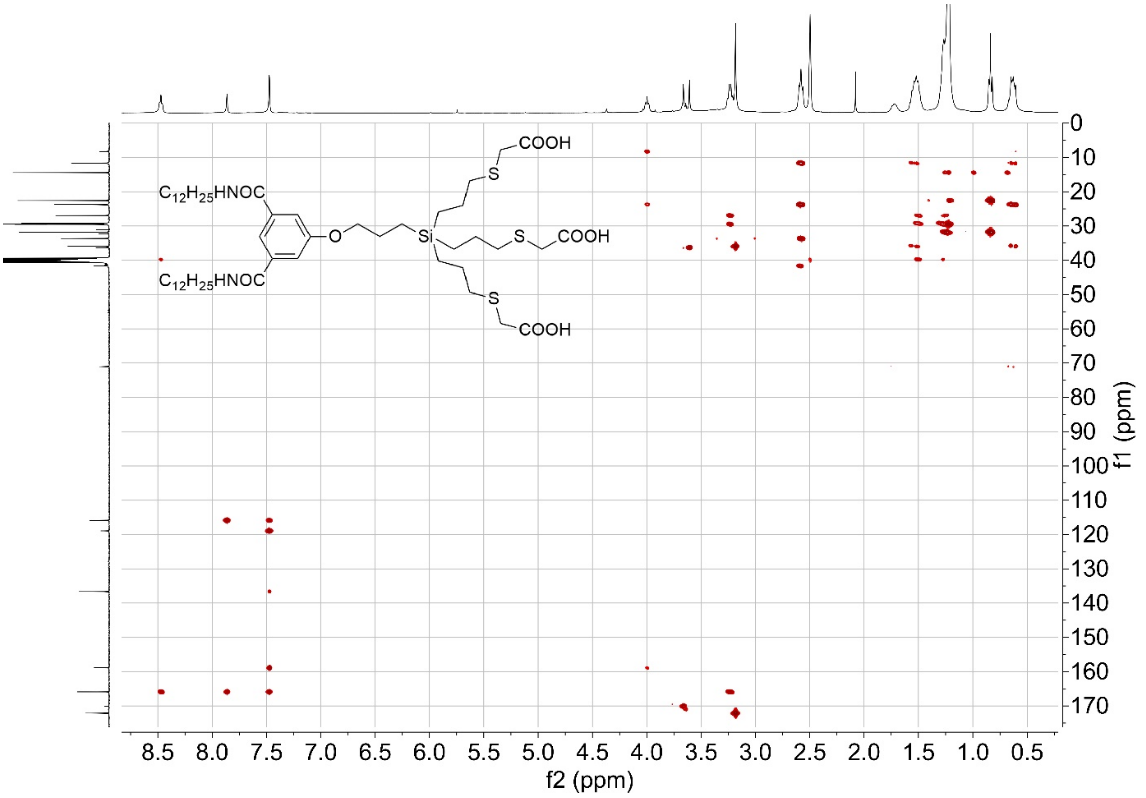

Figure S31. HMBC NMR (400 MHz, DMSO-*d*6) of **L1-7**

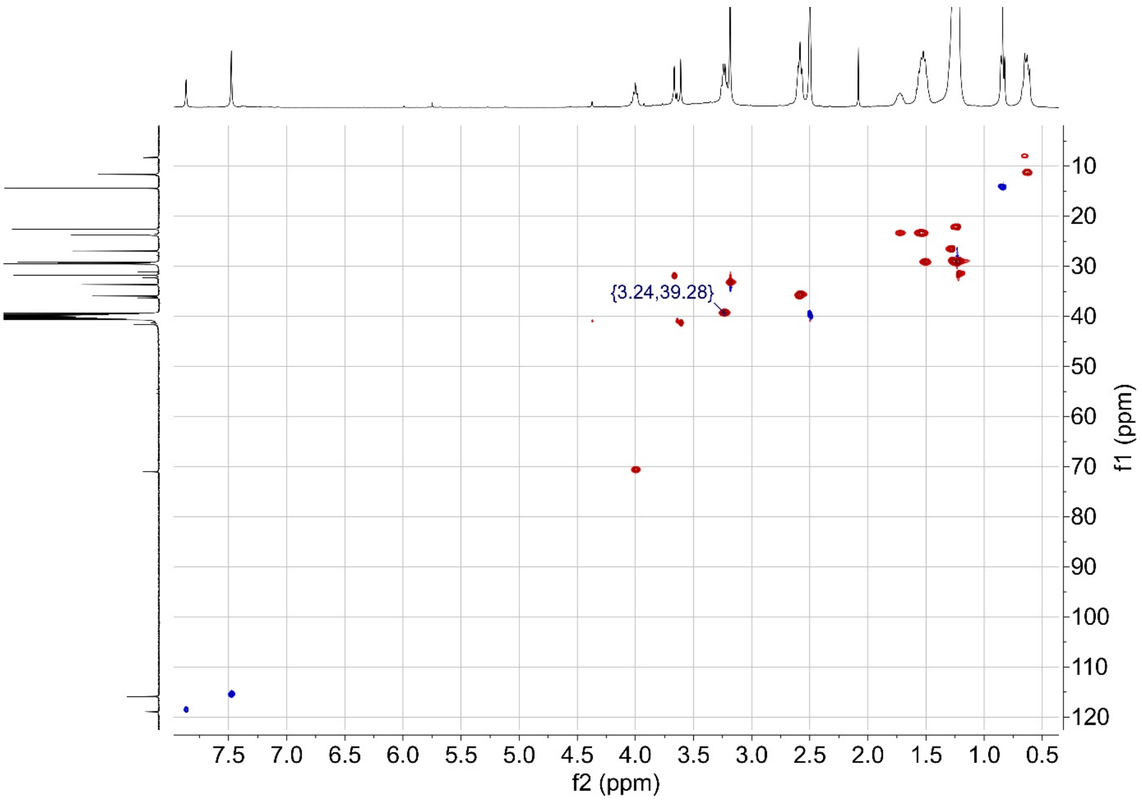

Figure S32. HSQC NMR (400 MHz, DMSO-d_6) of **L1-7**

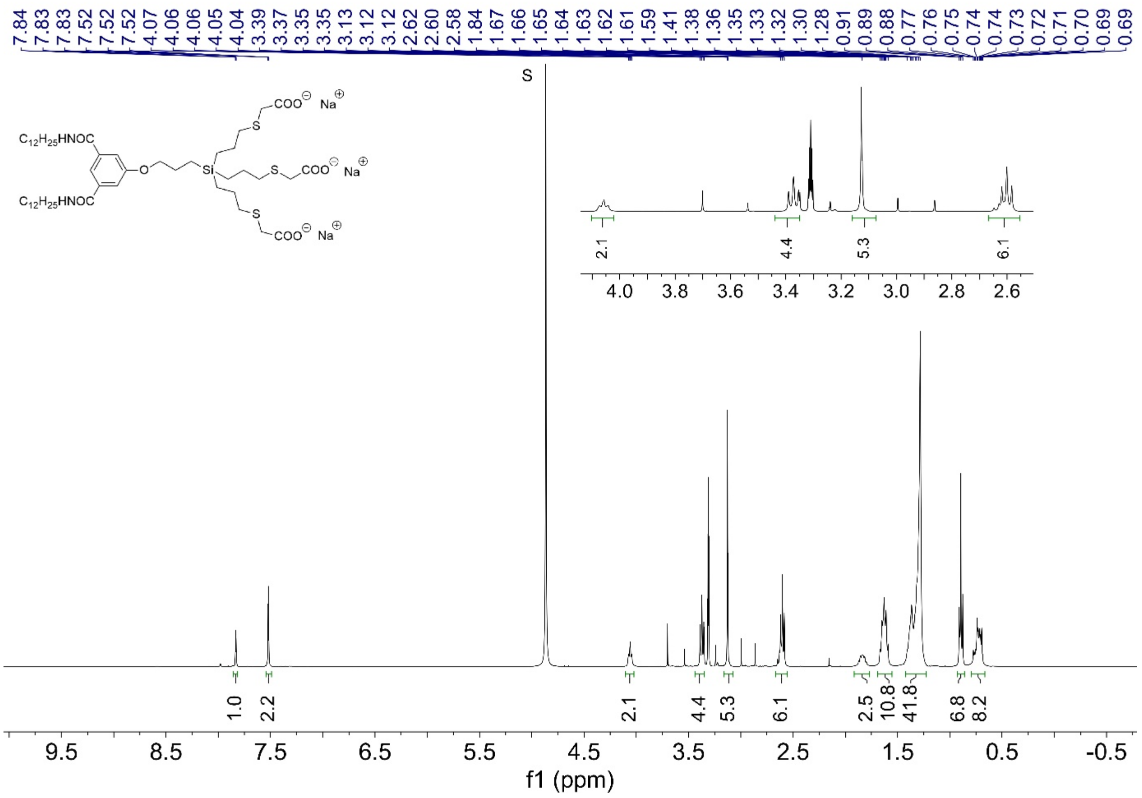

Figure S33. ^1H NMR (400 MHz, MeOH-d_4) of **L1-8**

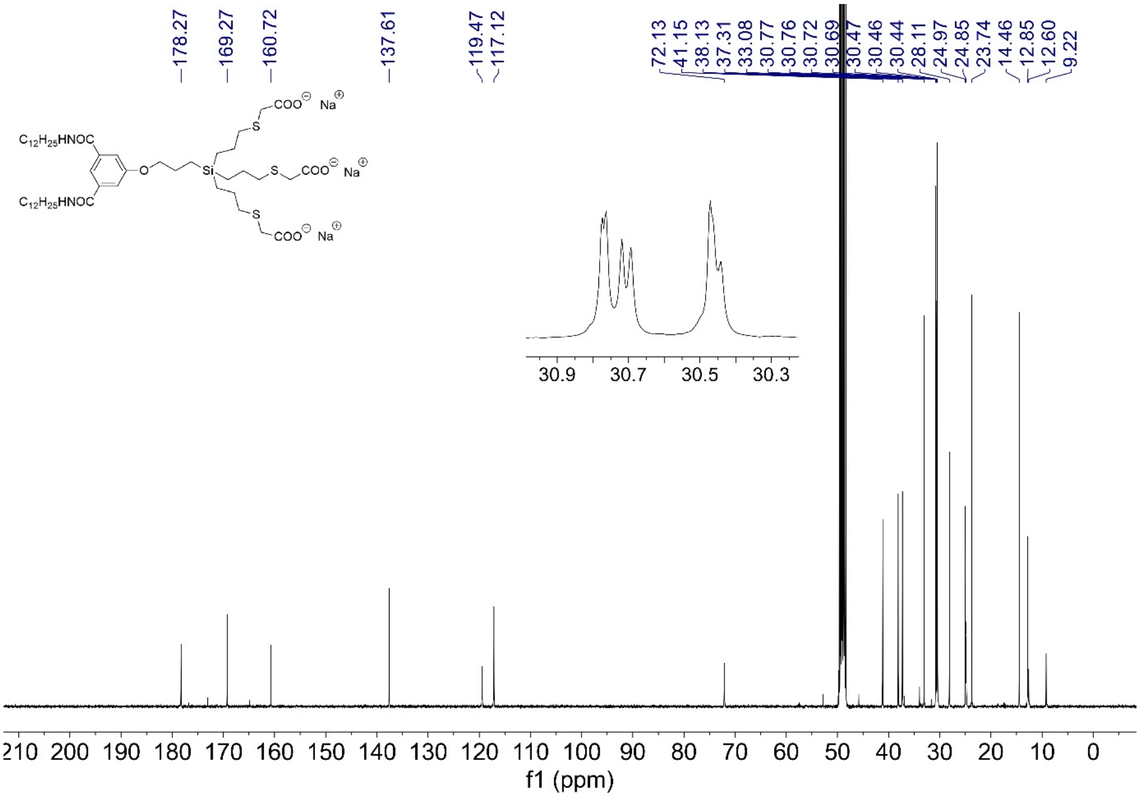

Figure S34. ^{13}C NMR (400 MHz, MeOH-d_4) of **L1-8**

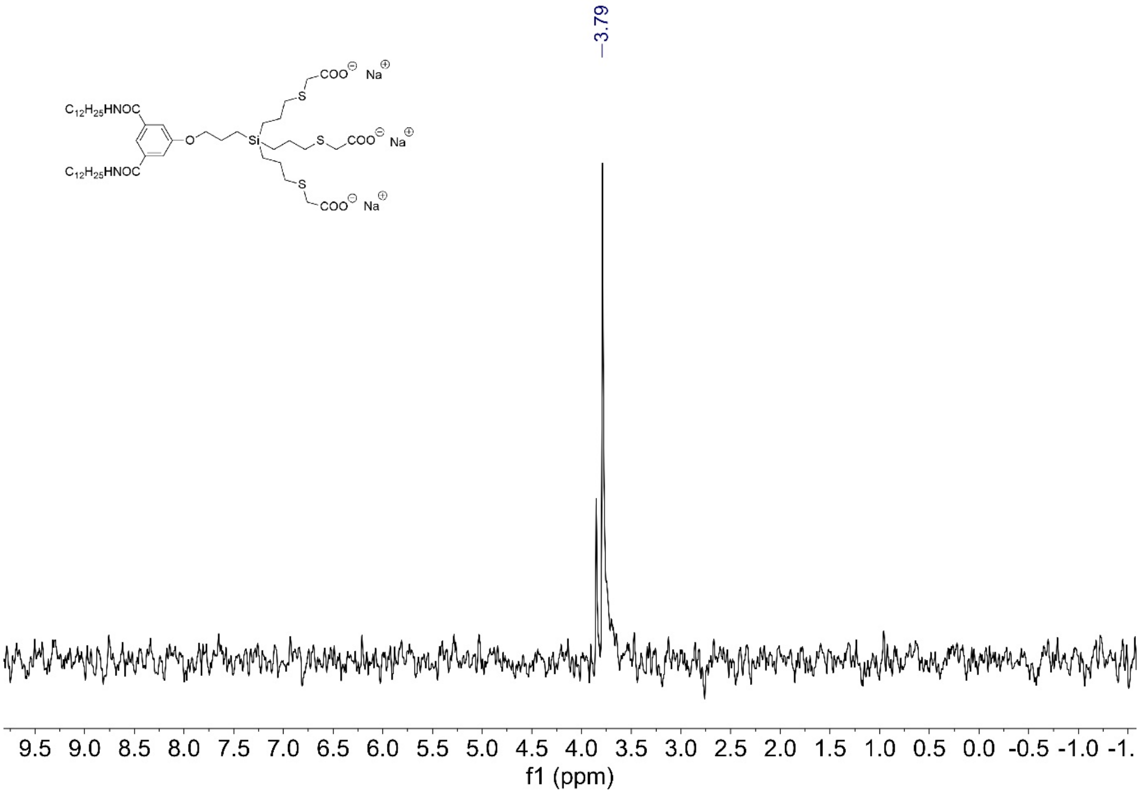

Figure S35. ^{29}Si NMR (400 MHz, MeOH-d_4) of **L1-8**

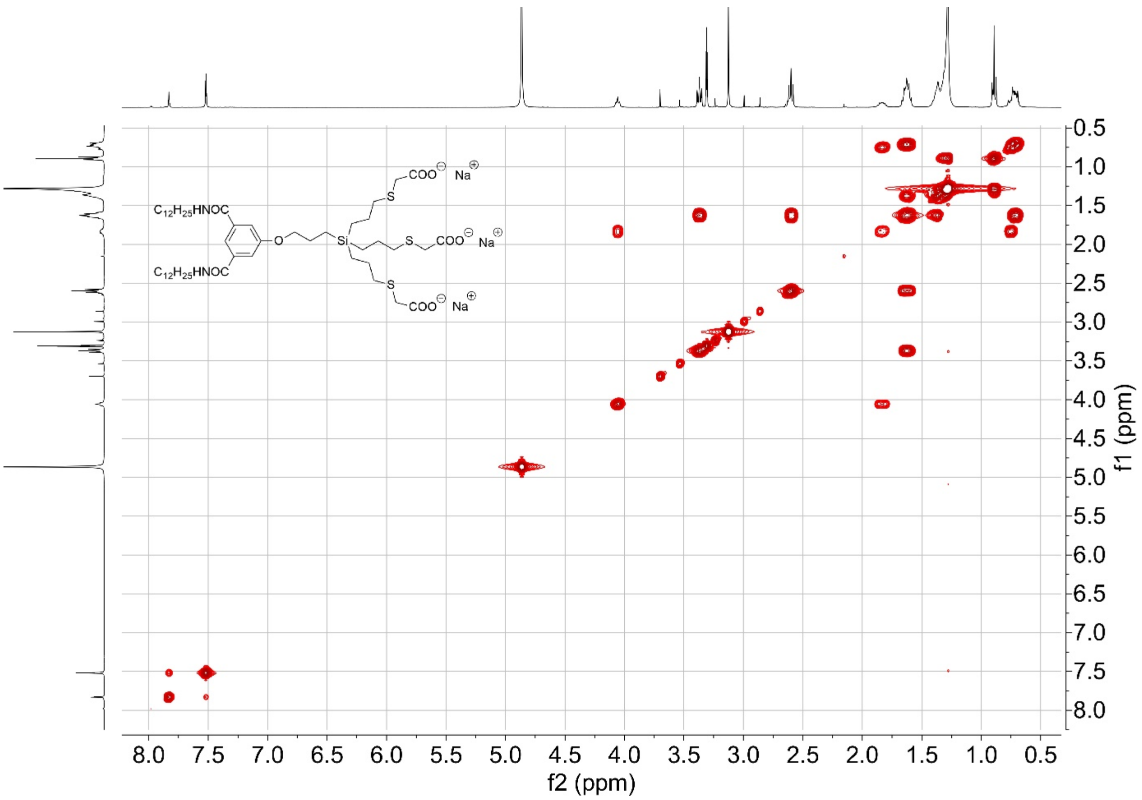

Figure S36. COSY NMR (400 MHz, MeOH-*d*4) of **L1-8**

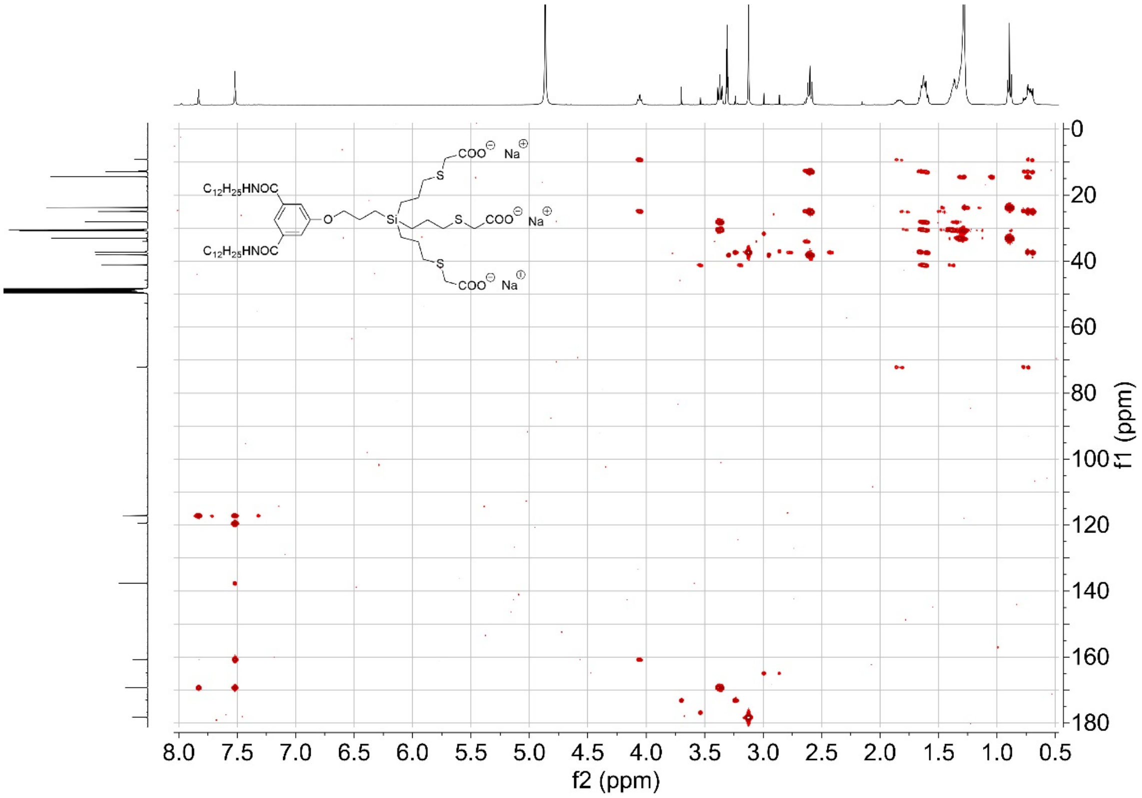

Figure S37. HMBC NMR (400 MHz, MeOH-*d*4) of **L1-8**

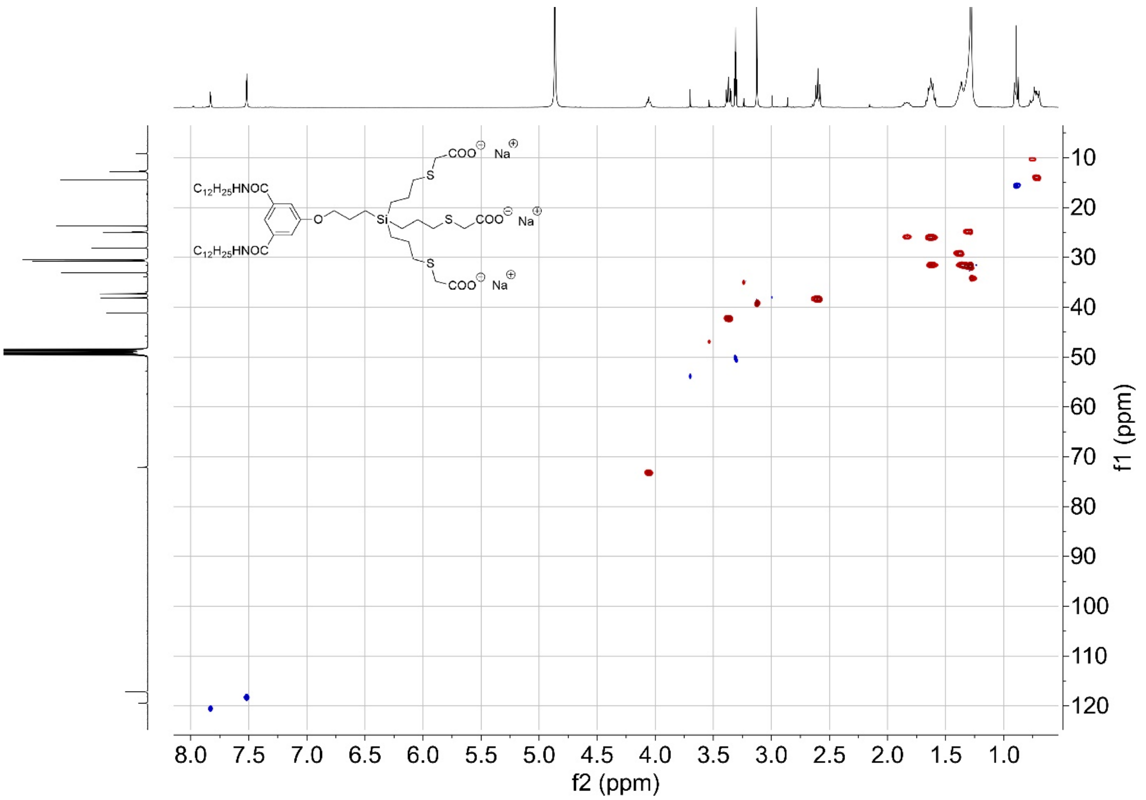

Figure S38. HSQC NMR (400 MHz, MeOH-d_4) of **L1-8**

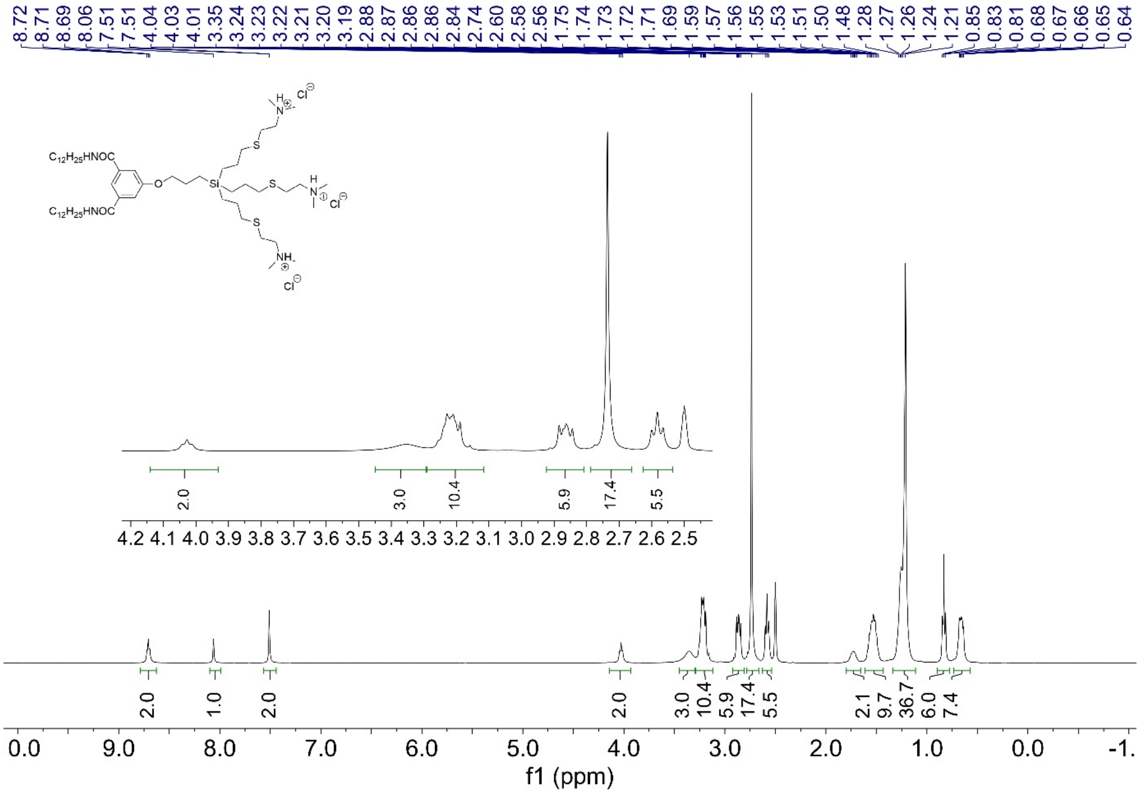

Figure S39. ^{1}H NMR (400 MHz, DMSO-d_6) of **L1-9**

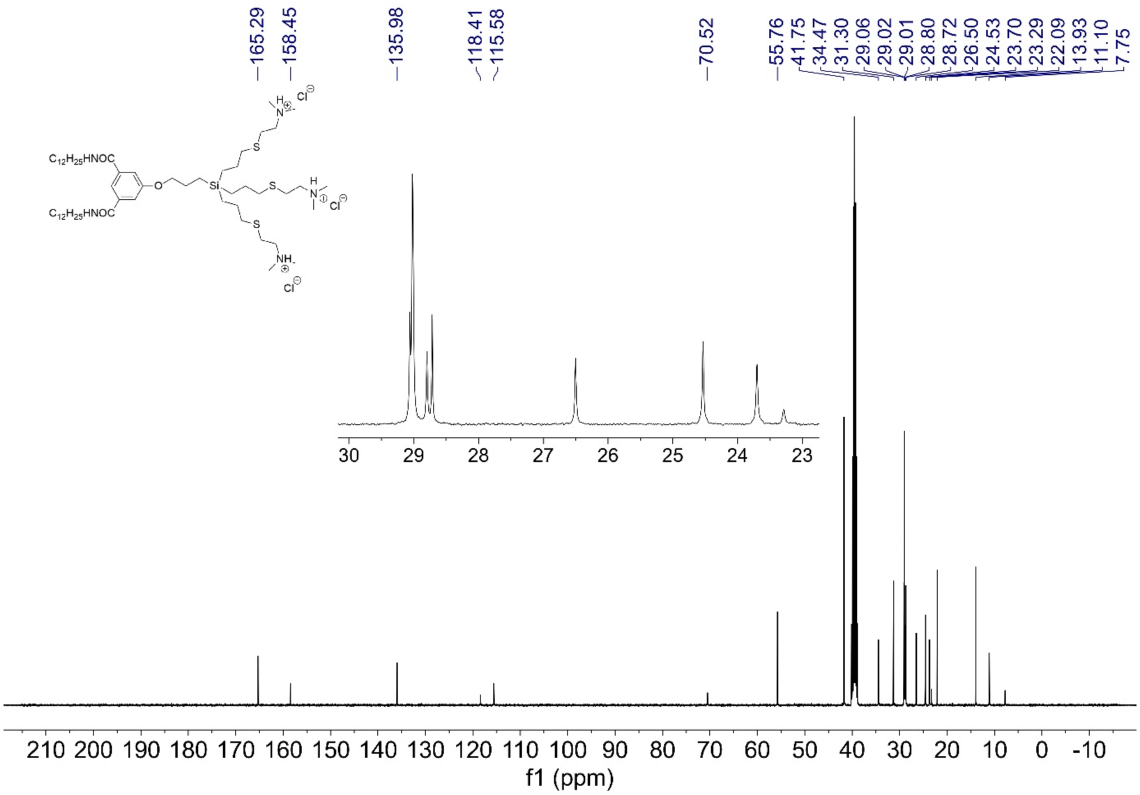

Figure S40. ^{13}C NMR (400 MHz, DMSO-d_6) of **L1-9**

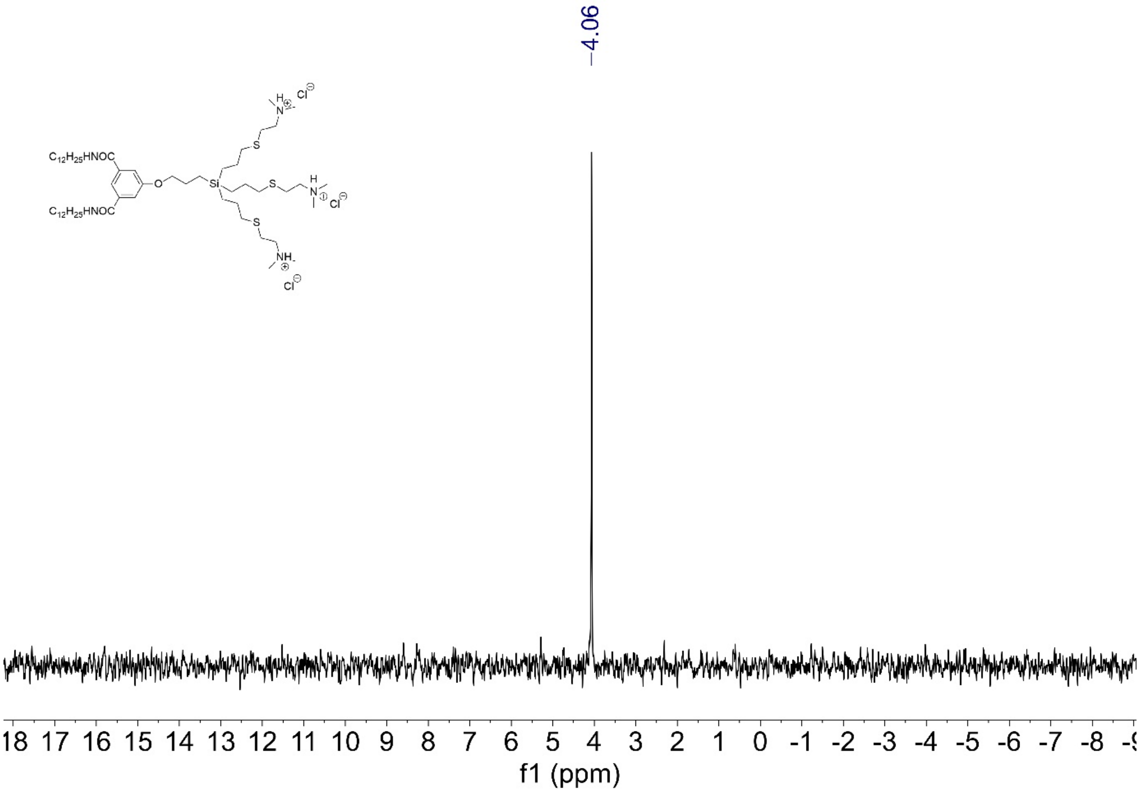

Figure S41. ^{29}Si NMR (400 MHz, DMSO-d_6) of **L1-9**

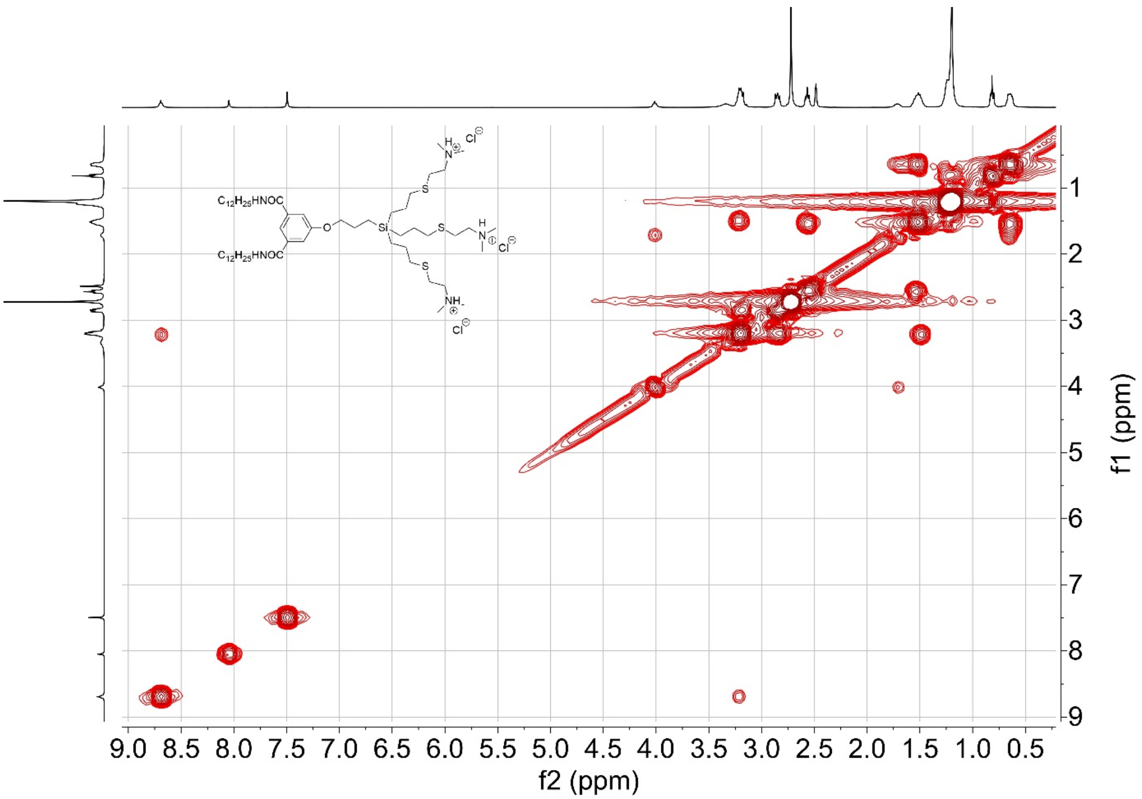

Figure S42. COSY NMR (400 MHz, DMSO-d_6) of **L1-9**

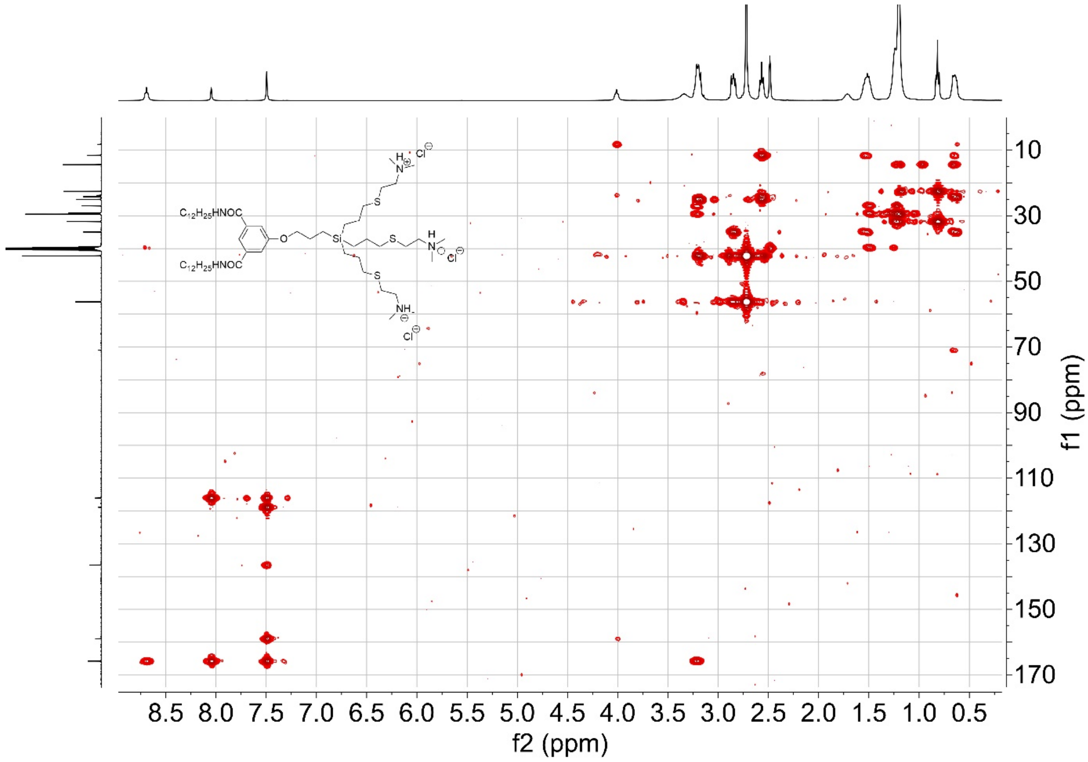

Figure S43. HMBC NMR (400 MHz, DMSO-d_6) of **L1-9**

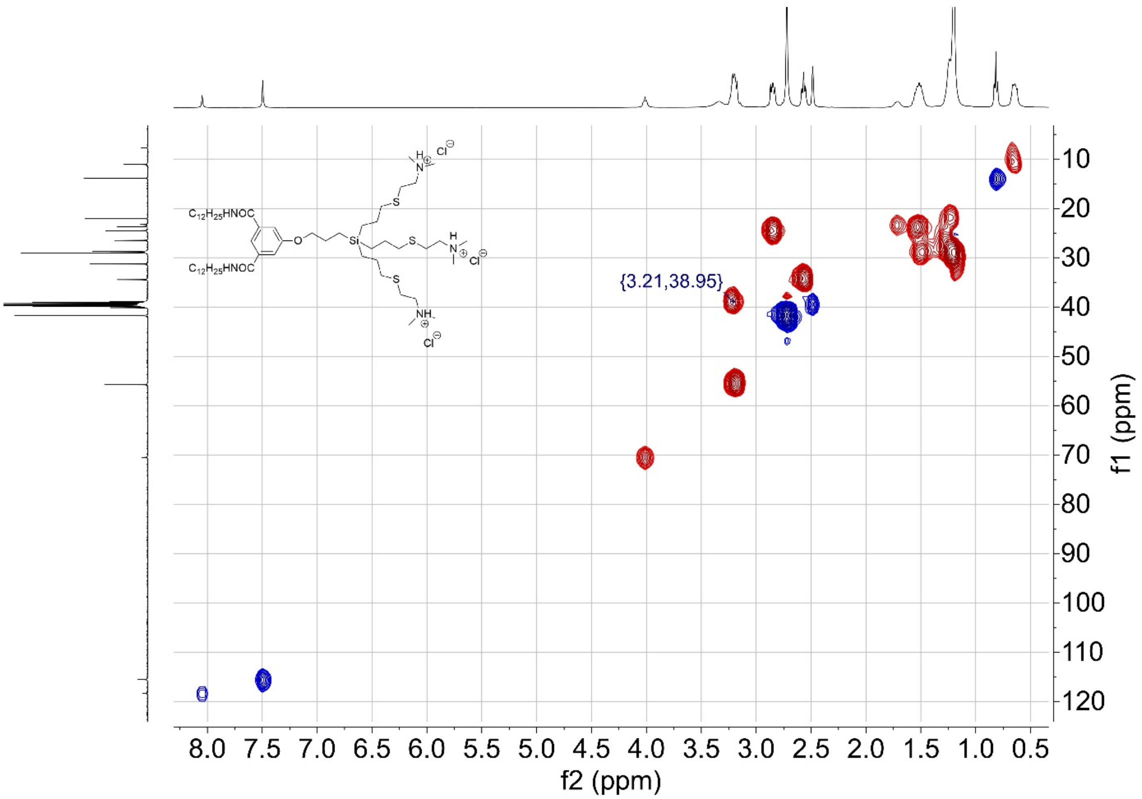

Figure S44. HSQC NMR (400 MHz, DMSO-d_6) of **L1-9**

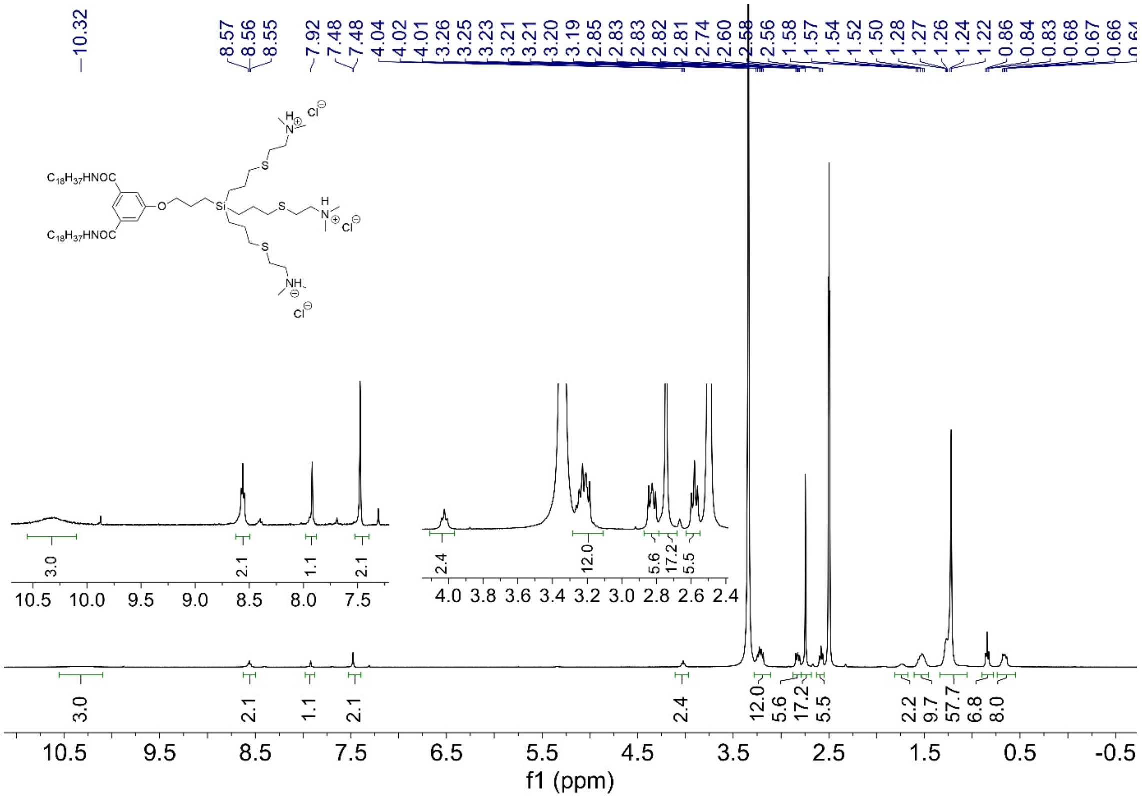

Figure S45. ^{1}H NMR (400 MHz, DMSO-d_6) of **L2-12**

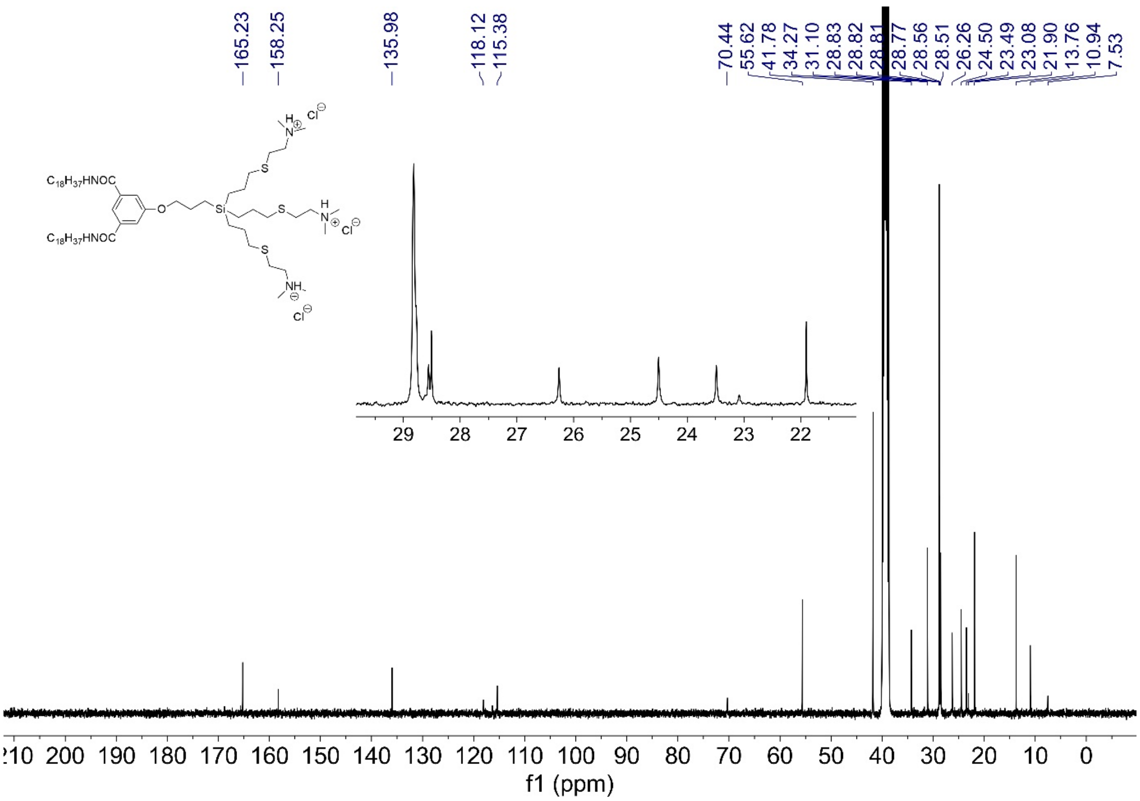

Figure S46. ^{13}C NMR (400 MHz, DMSO-d_6) of **L2-12**

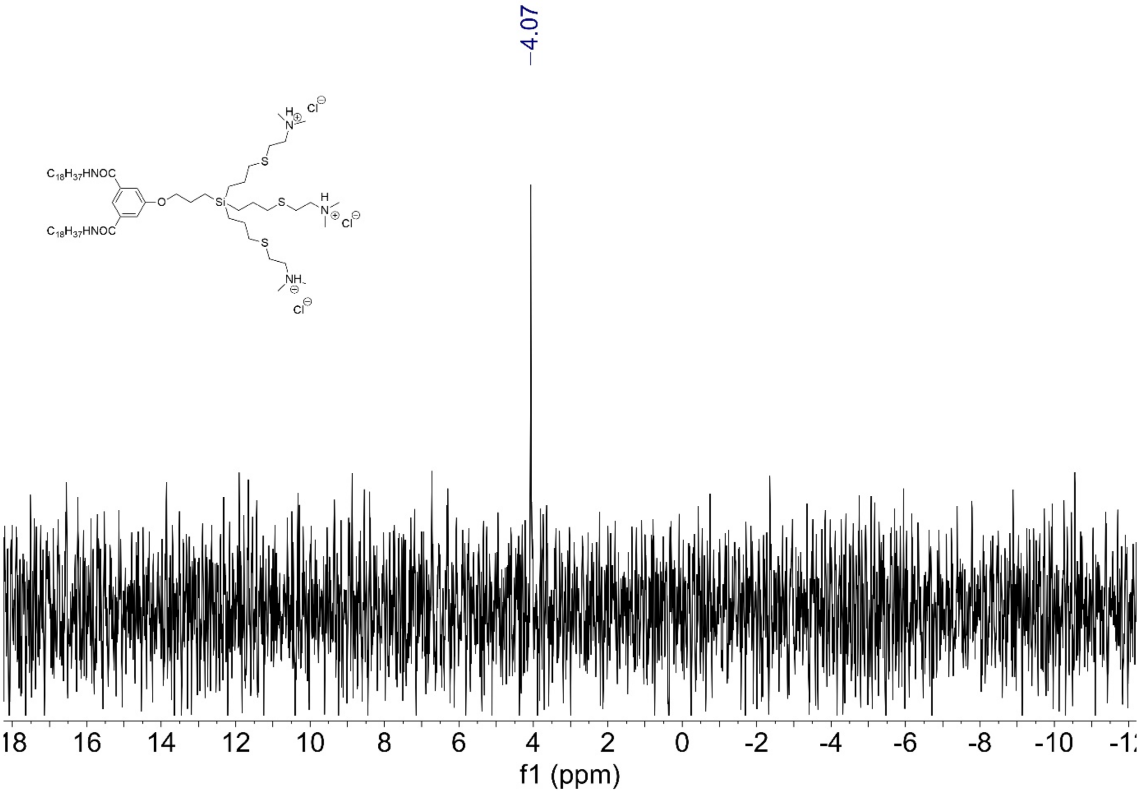

Figure S47. ^{29}Si NMR (400 MHz, DMSO-d_6) of **L2-12**

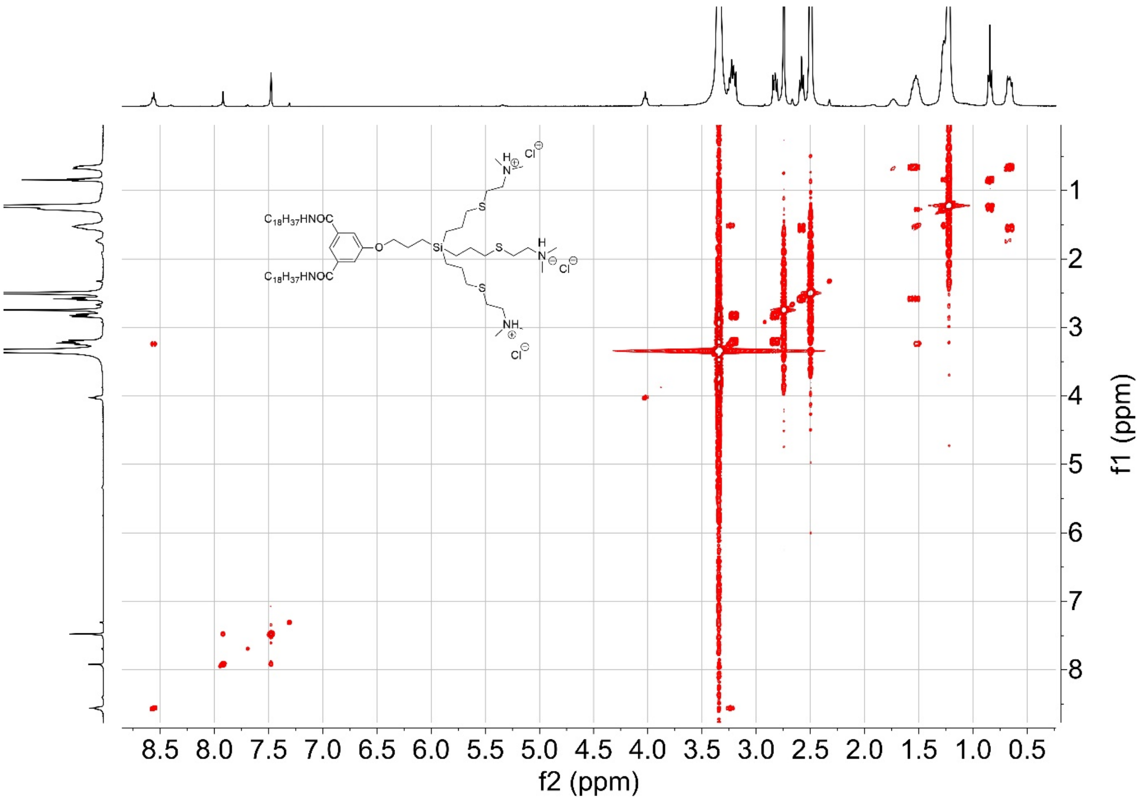

Figure S48. COSY (400 MHz, DMSO-d_6) of **L2-12**

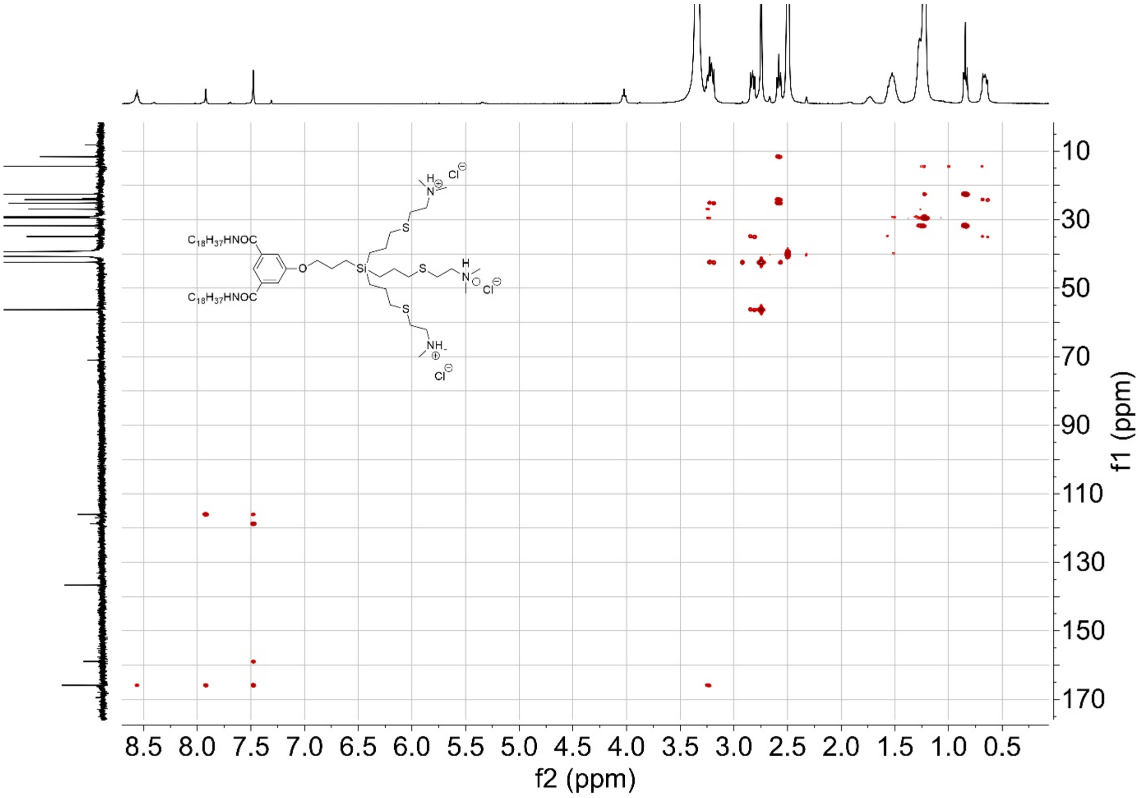

Figure S49. HMBC (400 MHz, DMSO-d_6) of **L2-12**

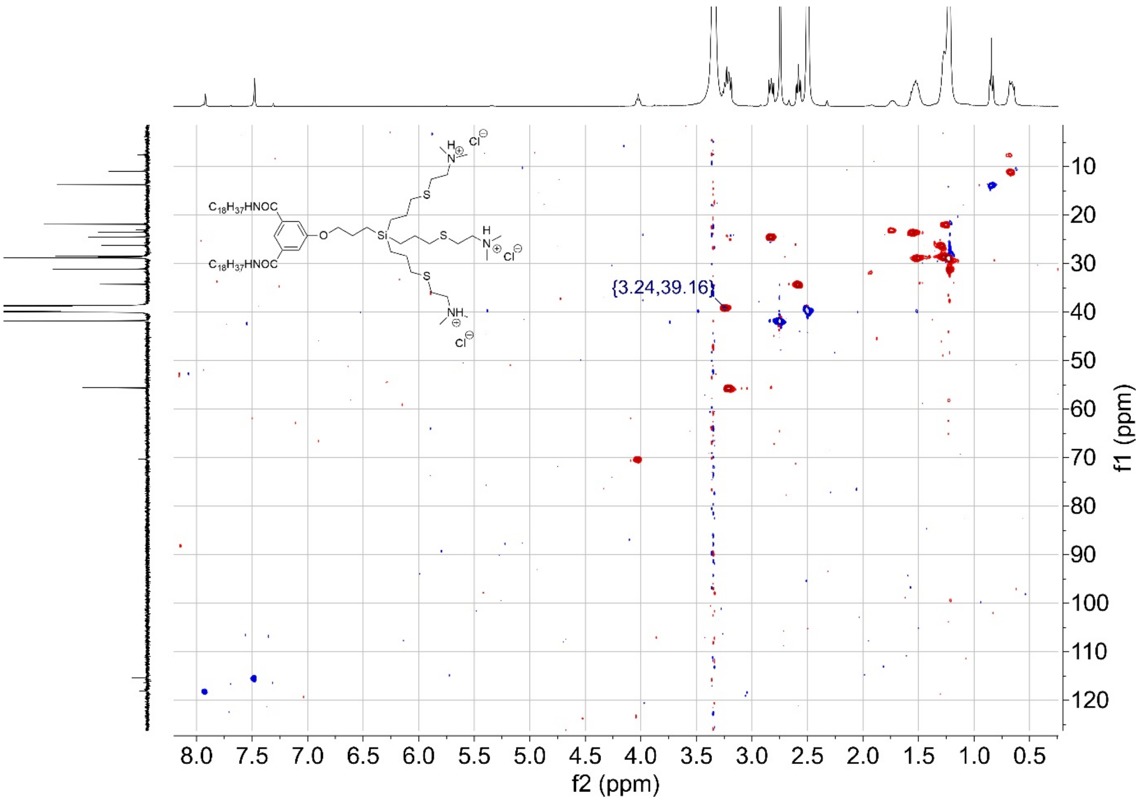

Figure S50. HSQC (400 MHz, DMSO-d_6) of **L2-12**

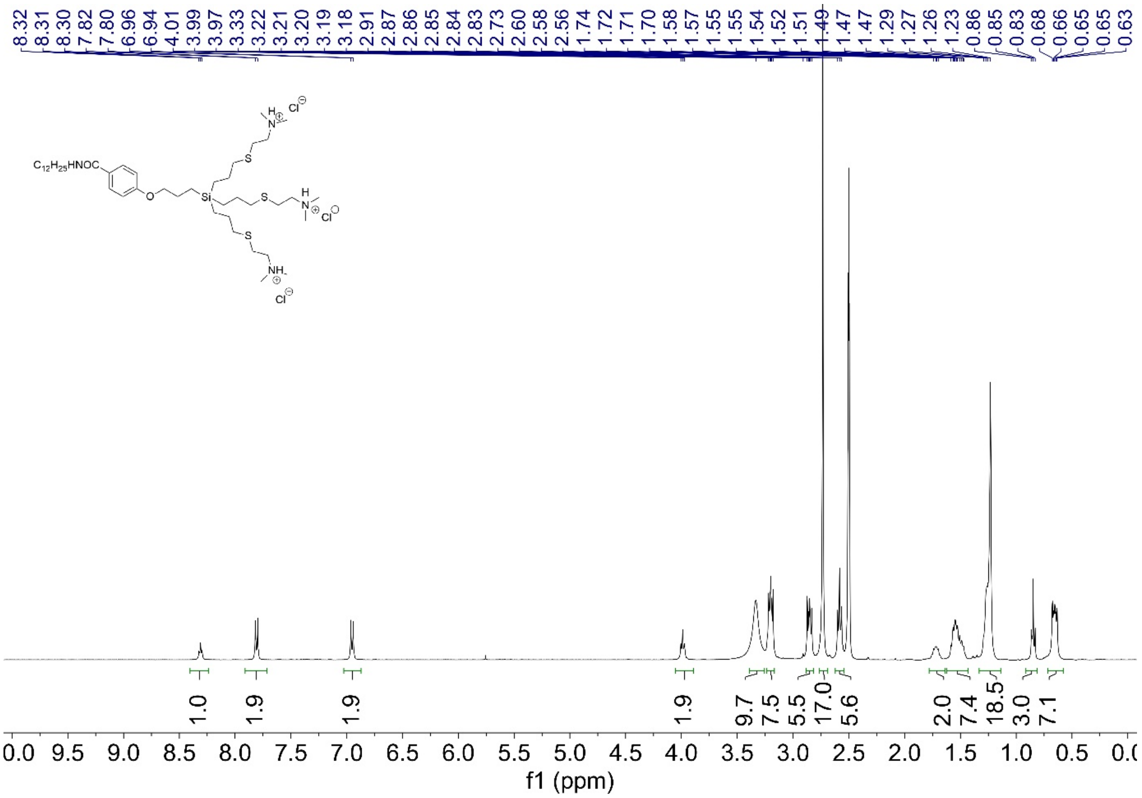

Figure S51. ^{1}H (400 MHz, DMSO-d_6) of **L2-15a**

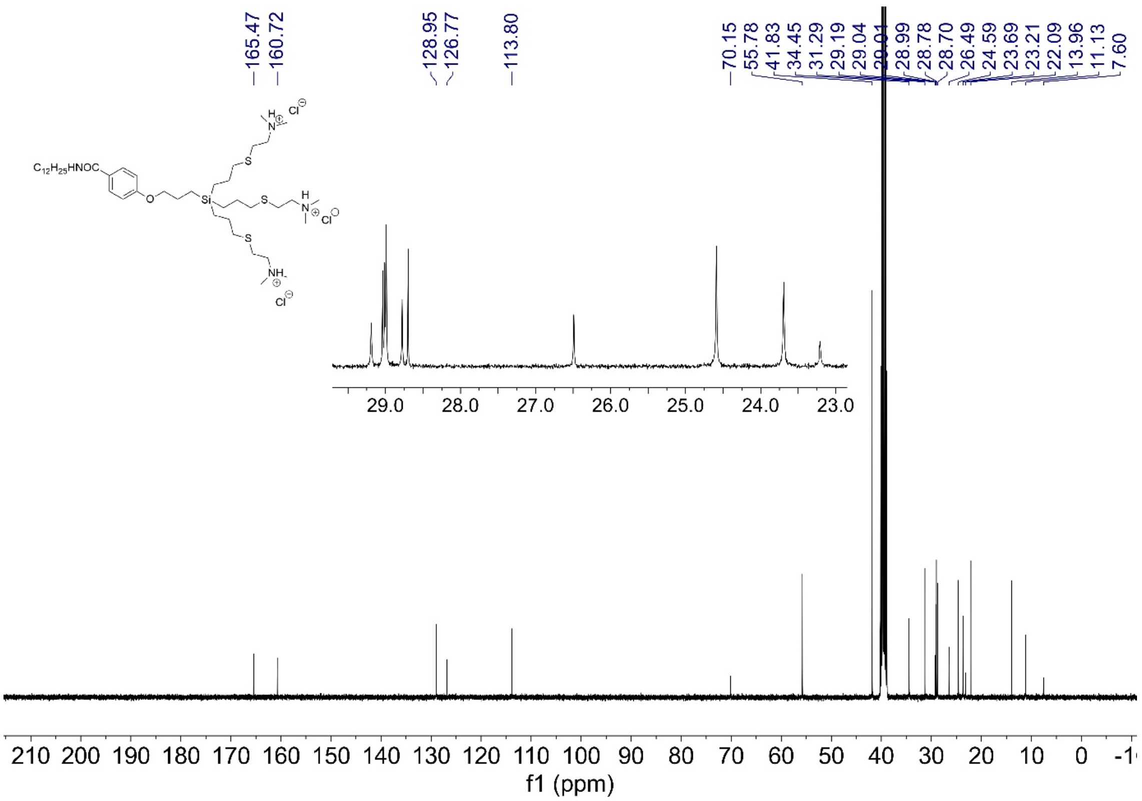

Figure S52. ^{13}C (400 MHz, DMSO-d_6) of **L2-15a**

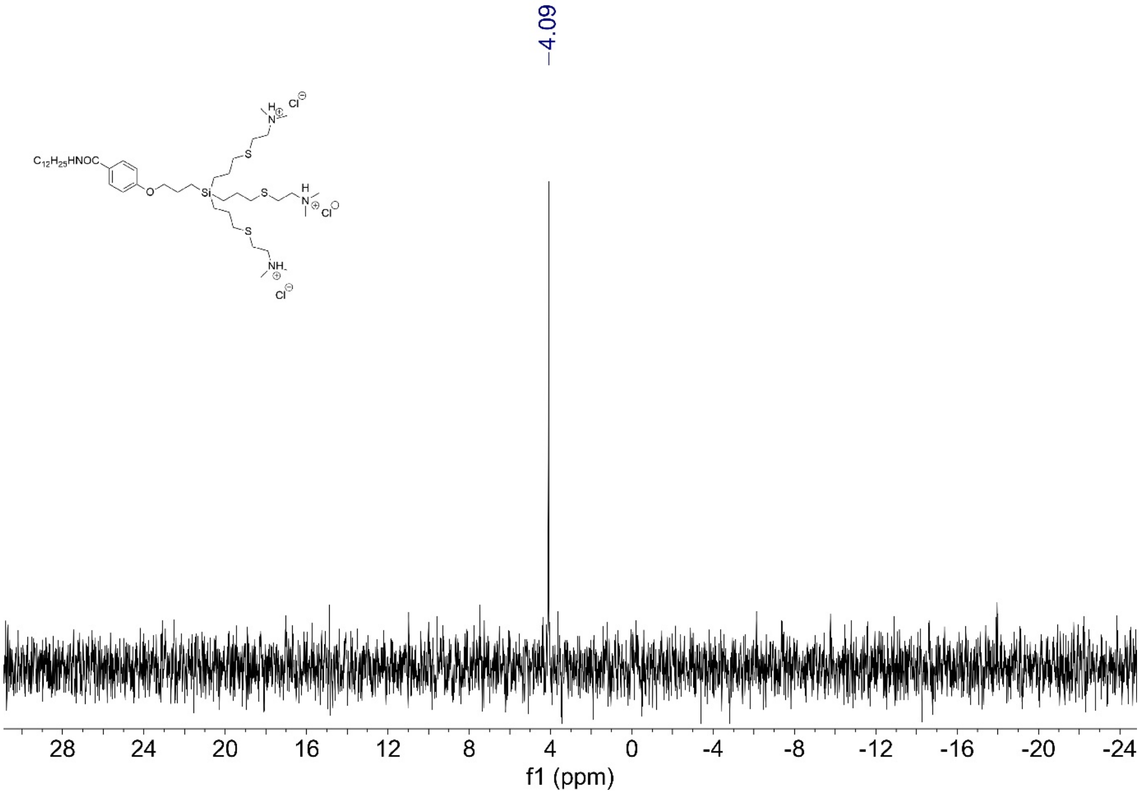

Figure S53. ^{29}Si (400 MHz, DMSO-d_6) of **L2-15a**

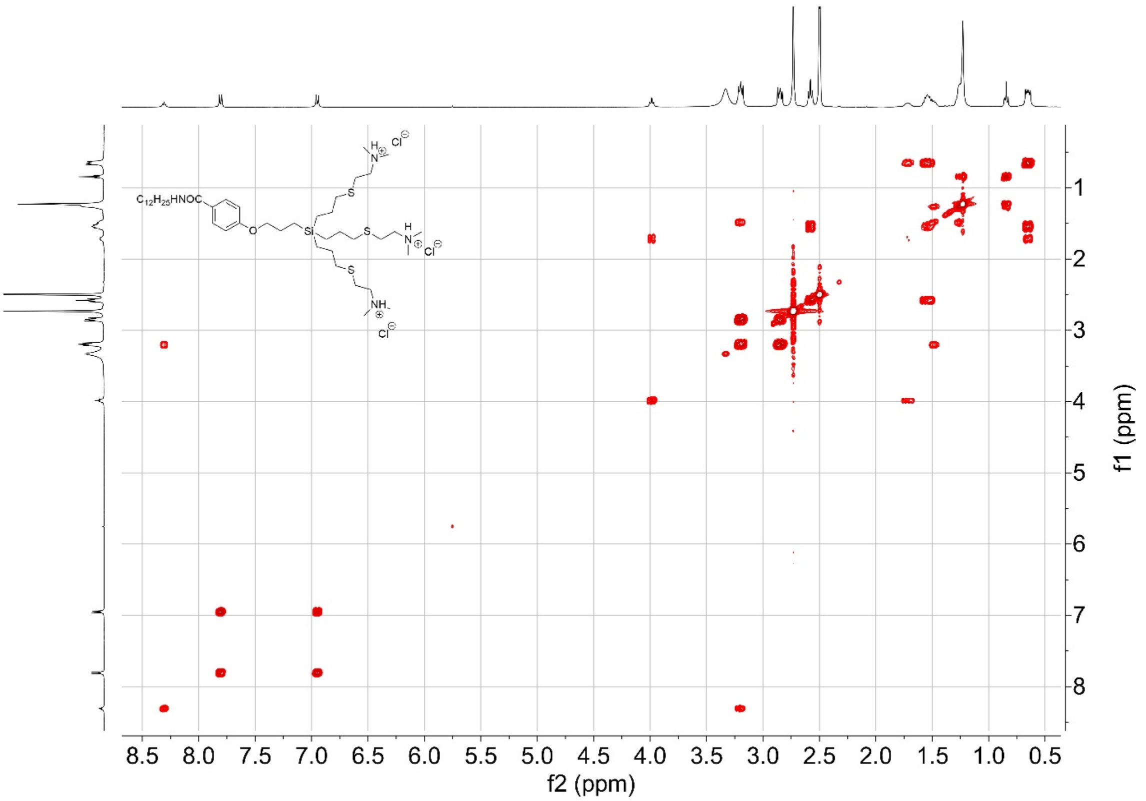

Figure S54. COSY (400 MHz, DMSO-d_6) of **L2-15a**

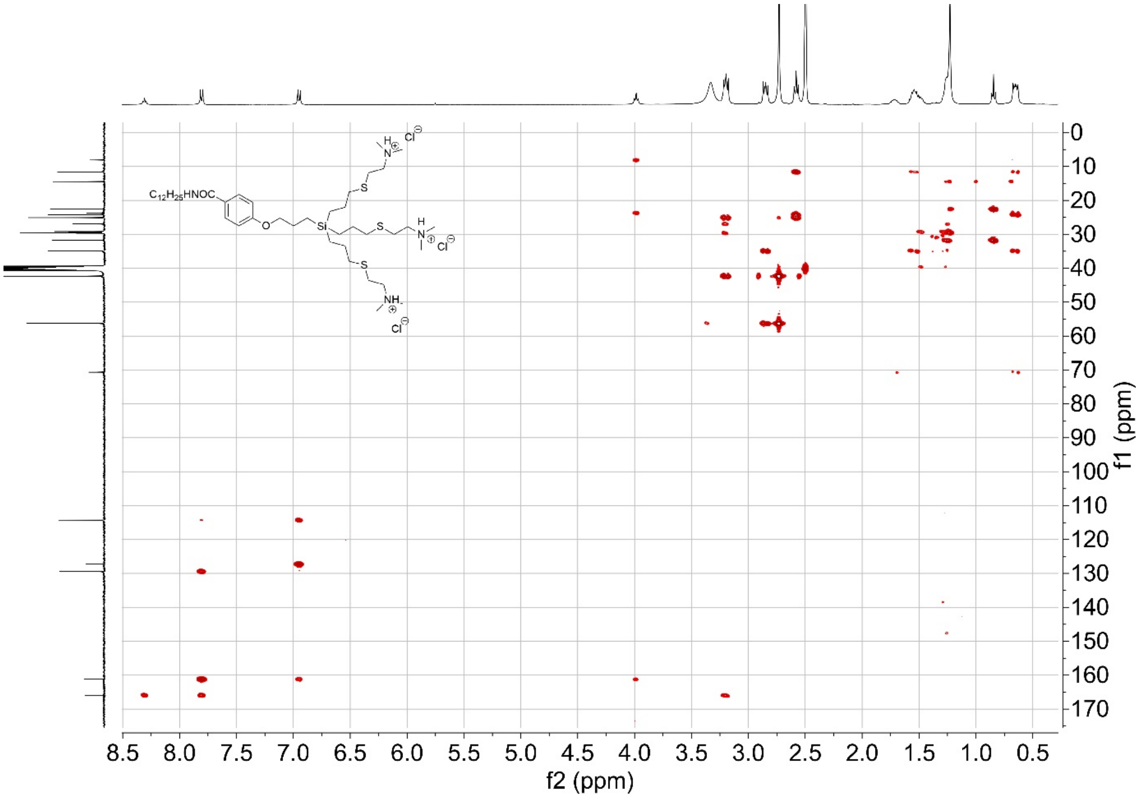

Figure S55. HMBC (400 MHz, DMSO-d_6) of **L2-15a**

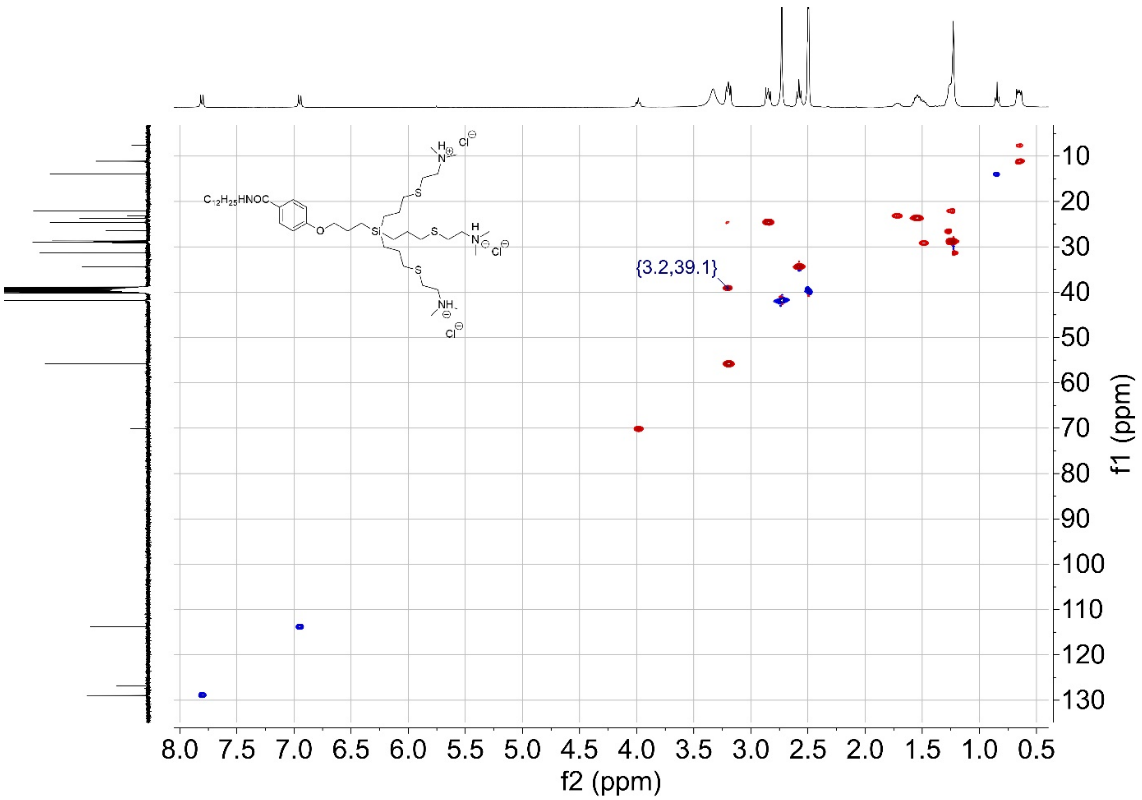

Figure S56. HSQC (400 MHz, DMSO-d_6) of **L2-15a**

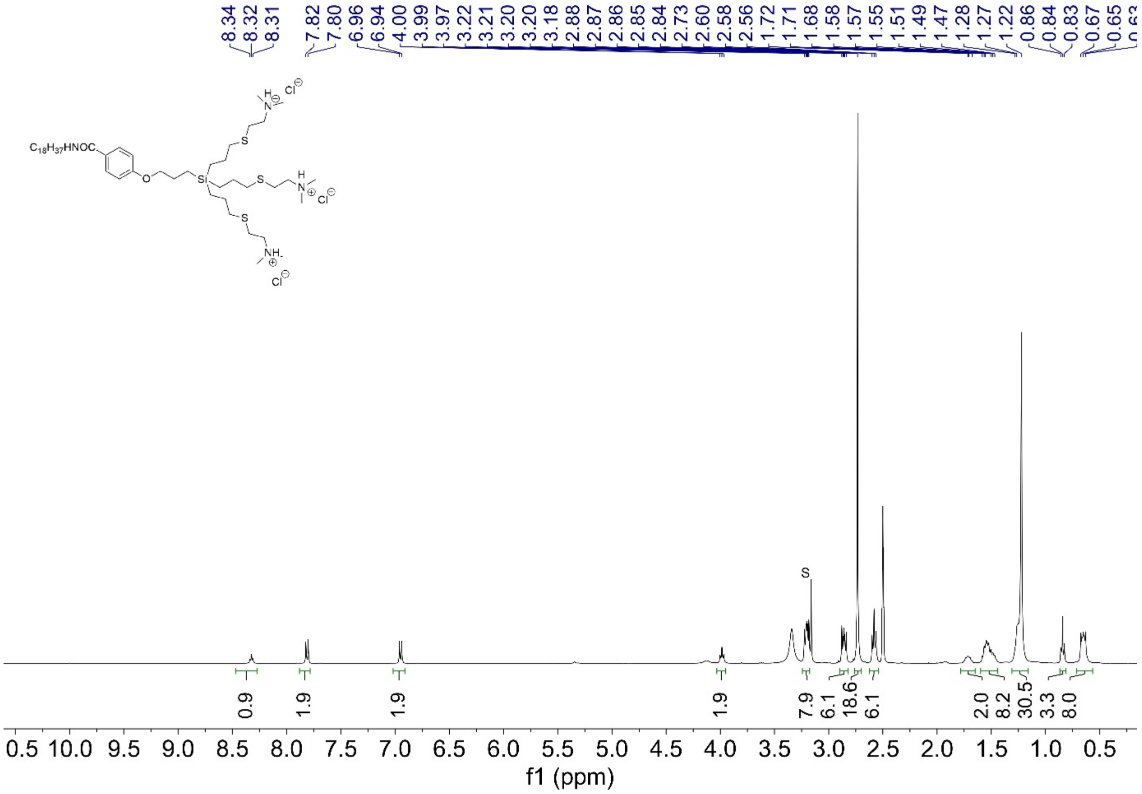

Figure S57. ^{1}H (400 MHz, DMSO-d_6) of **L2-15b**

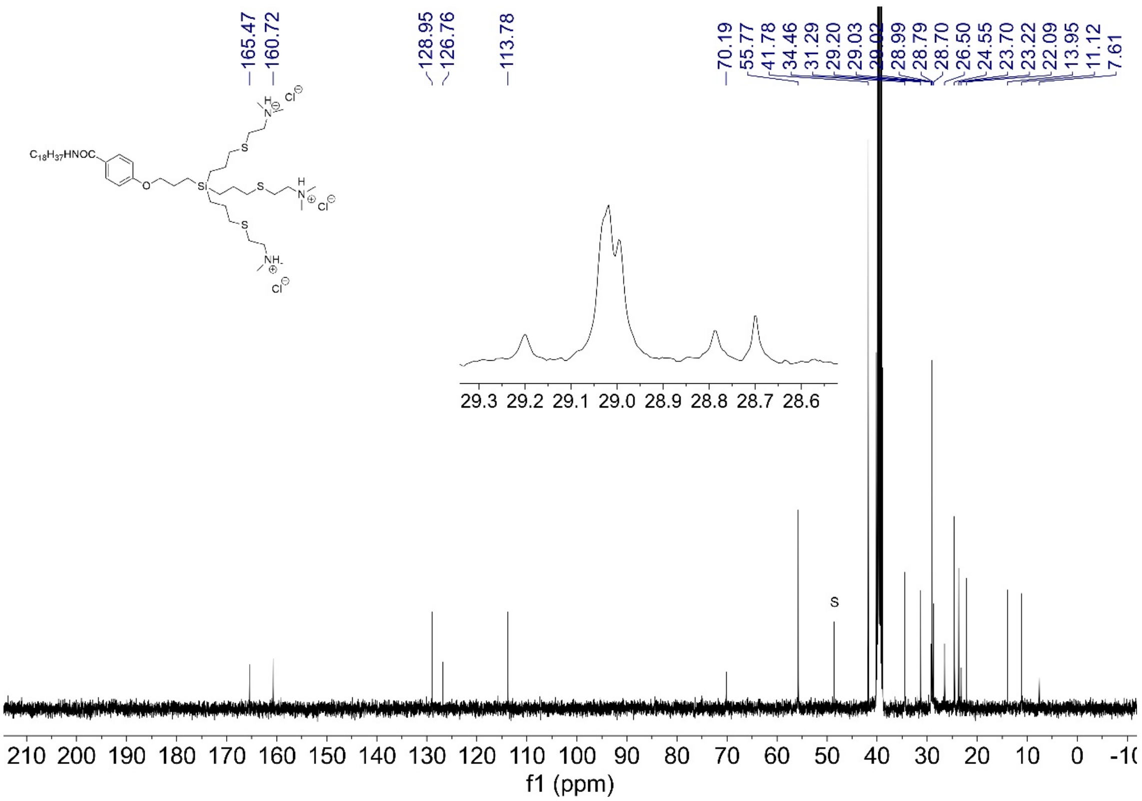

Figure S58. ^{13}C (400 MHz, DMSO-d_6) of **L2-15b**

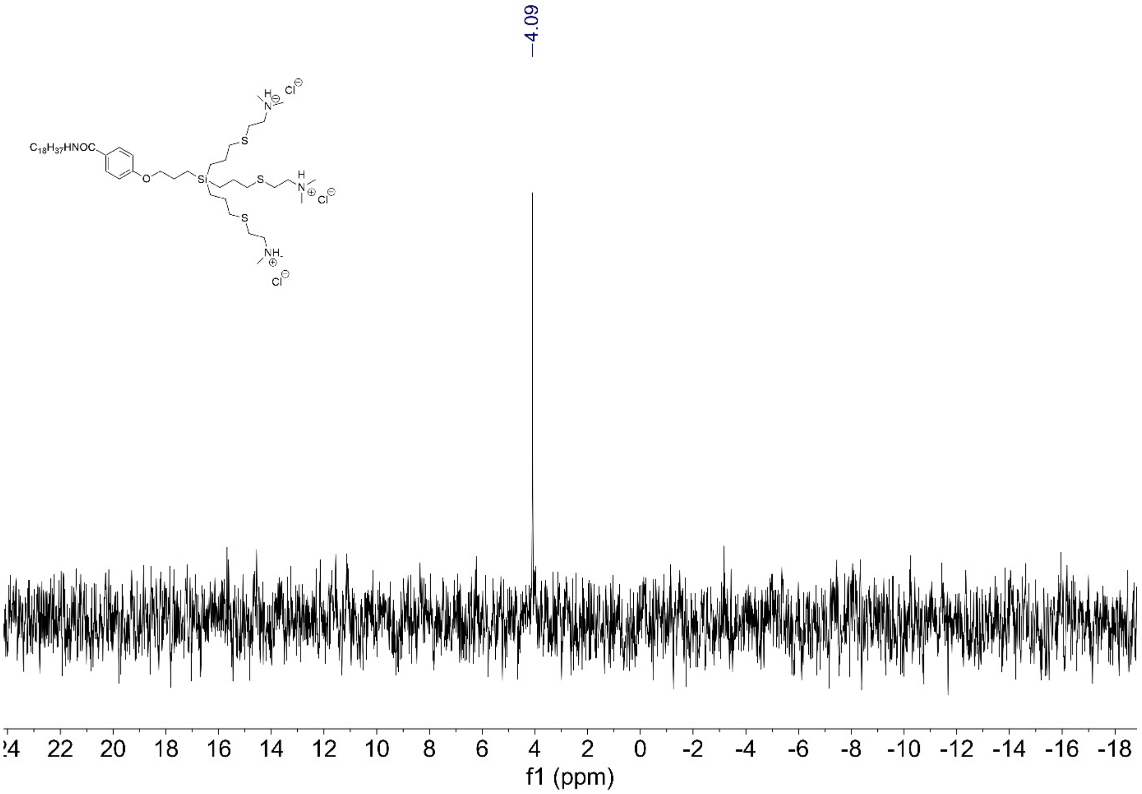

Figure S59. ^{29}Si (400 MHz, DMSO-d_6) of **L2-15b**

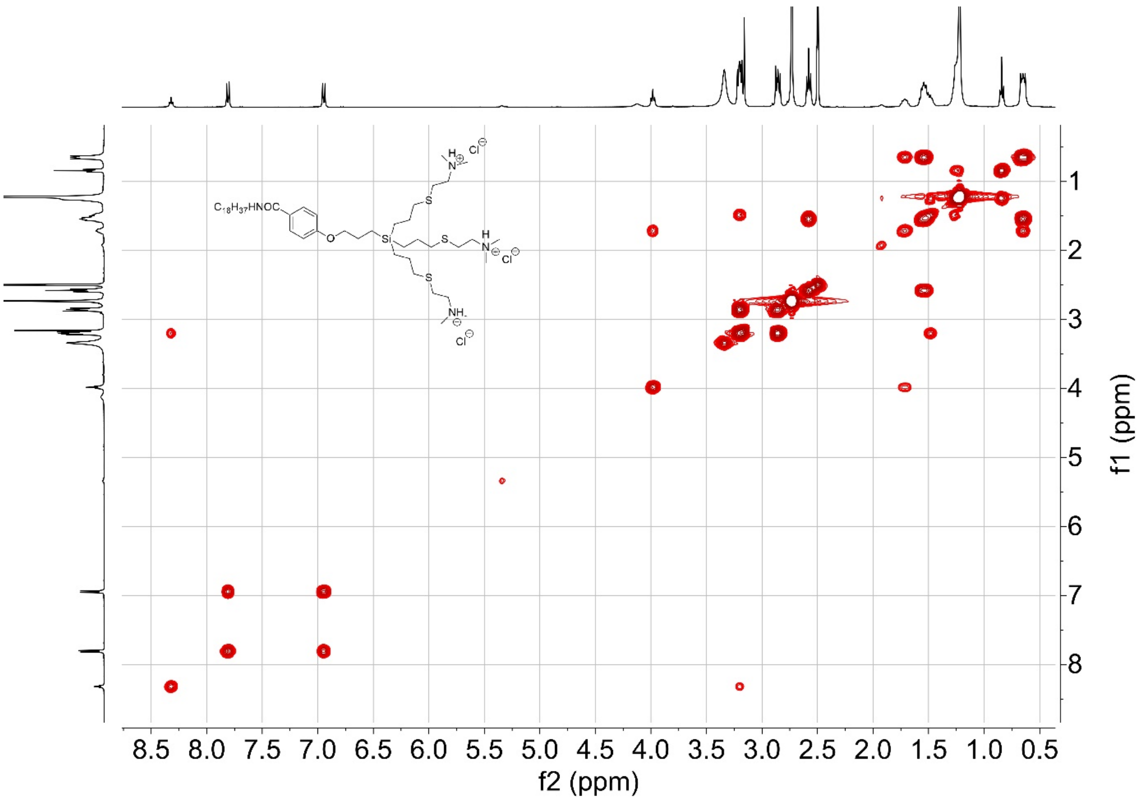

Figure S60. COSY (400 MHz, DMSO-d_6) of **L2-15b**

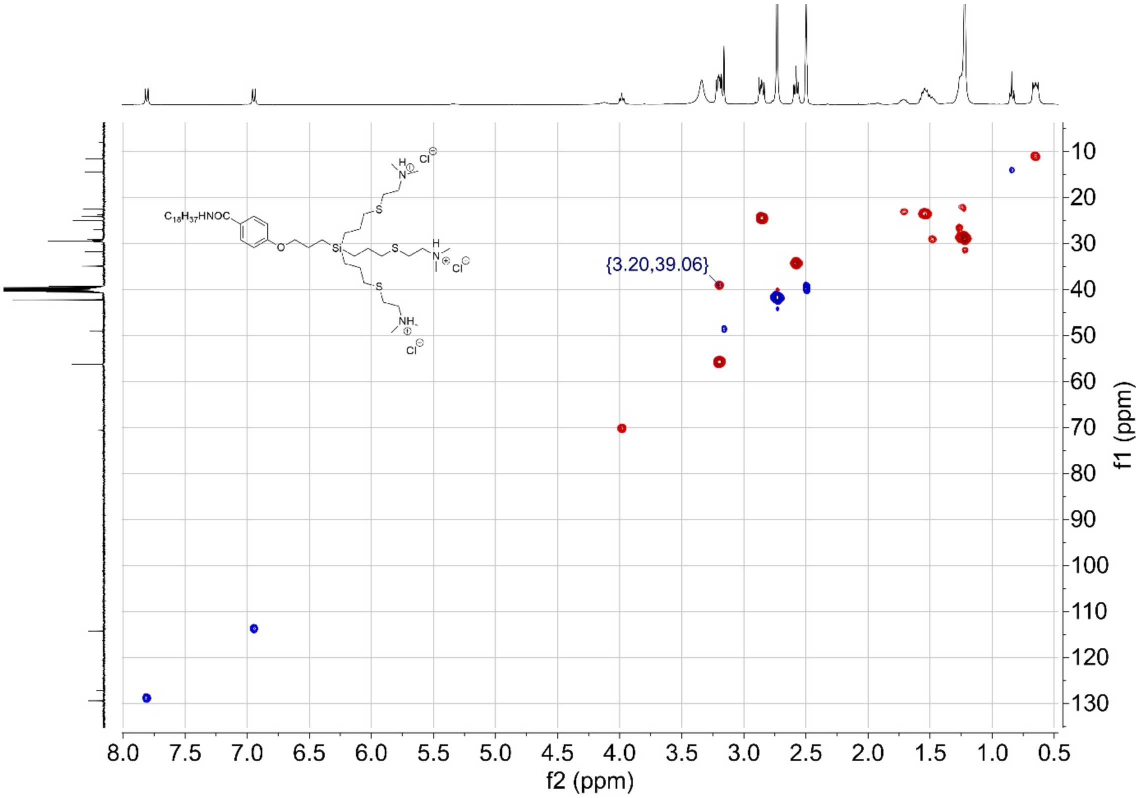

Figure S61. HSQC (400 MHz, DMSO-d_6) of **L2-15b**

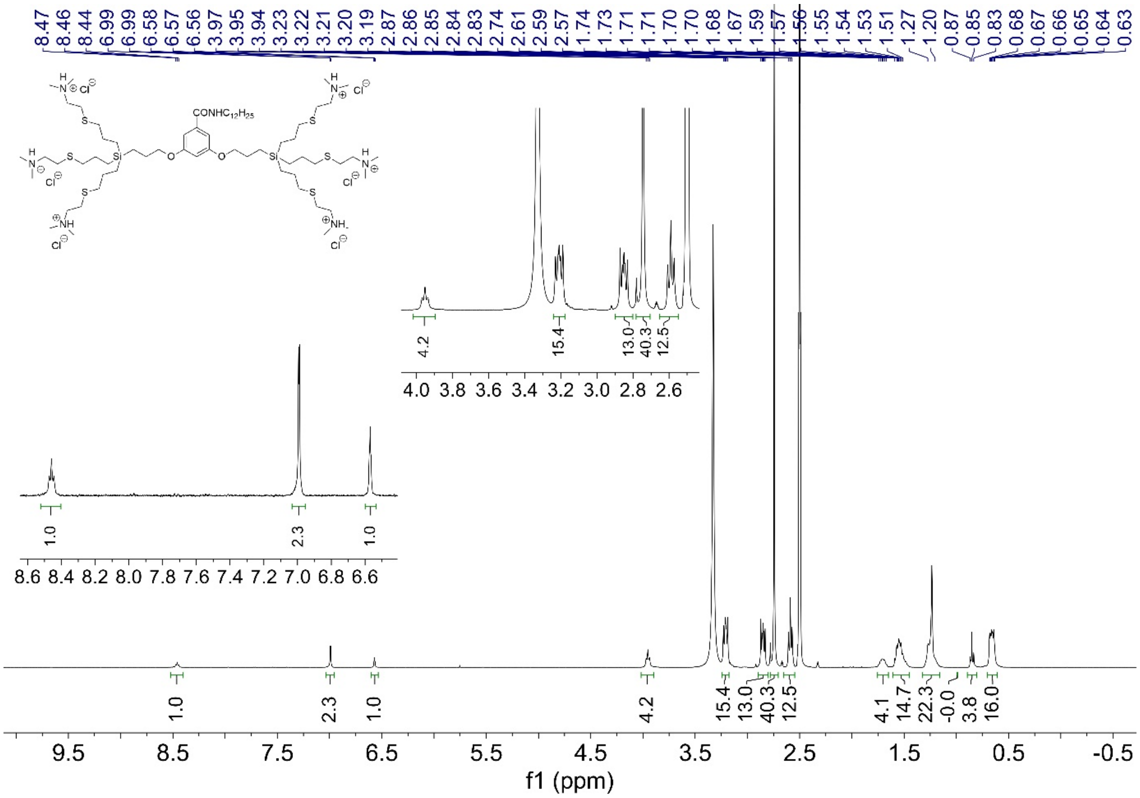

Figure S62. ^{1}H (400 MHz, DMSO-d_6) of **L2-19a**

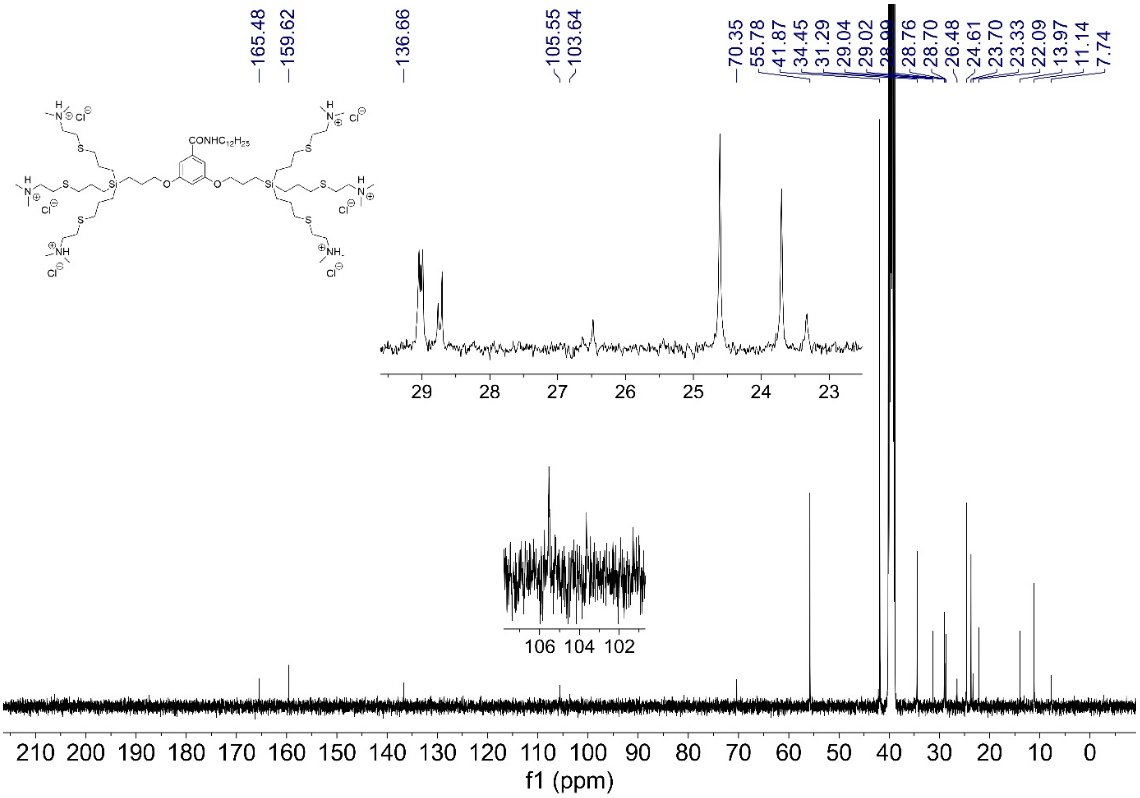

Figure S63. ^{13}C (400 MHz, DMSO-d_6) of **L2-19a**

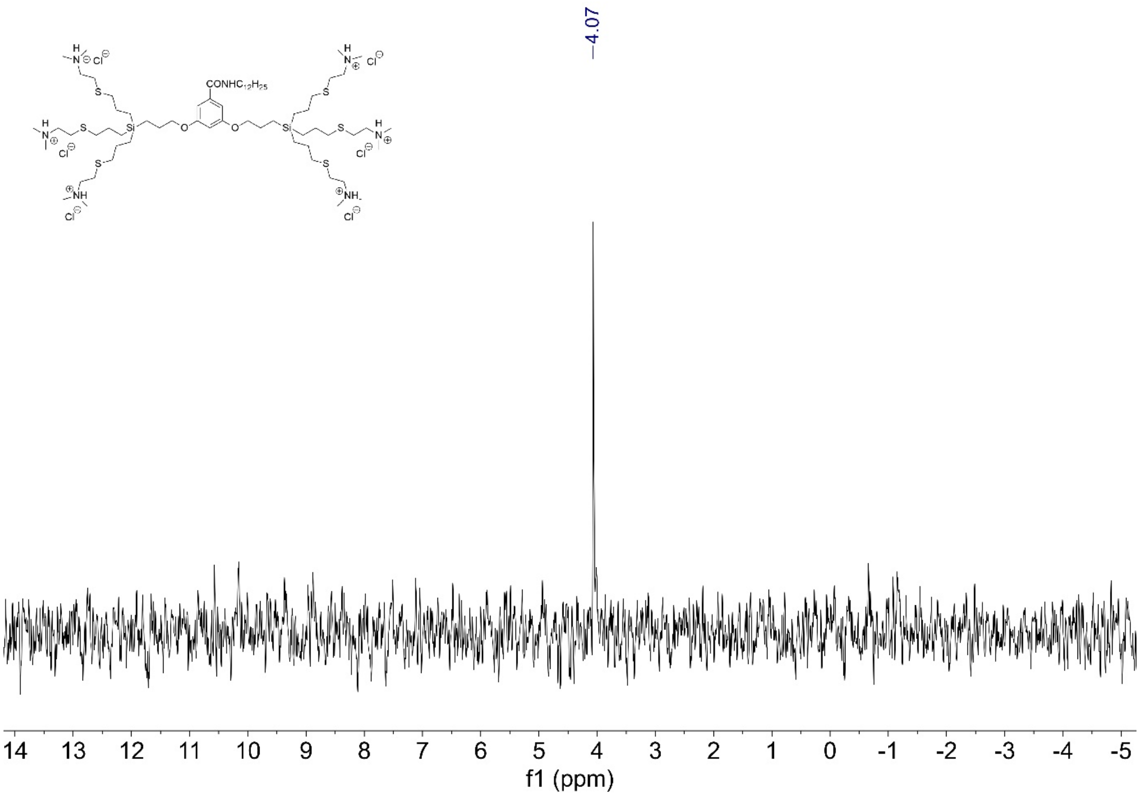

Figure S64. ^{29}Si (400 MHz, DMSO-d_6) of **L2-19a**

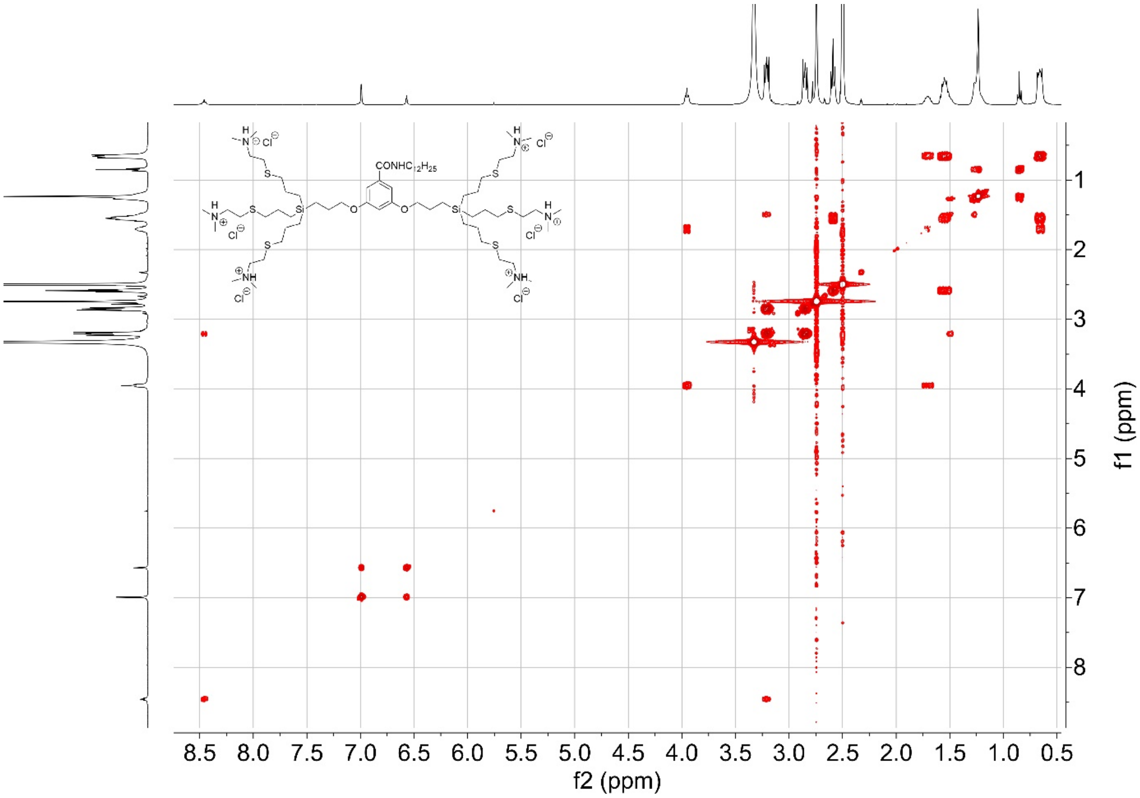

Figure S65. COSY (400 MHz, DMSO-d_6) of **L2-19a**

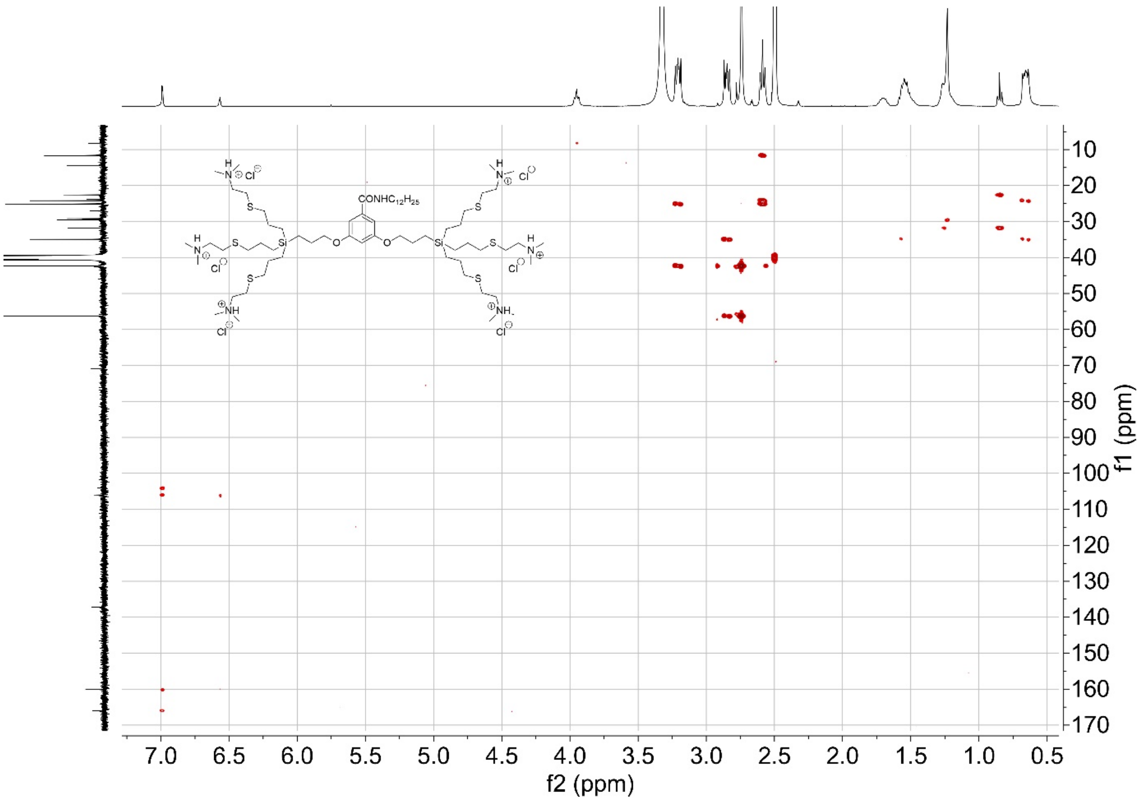

Figure S66. HMBC (400 MHz, DMSO-*d*6) of **L2-19a**

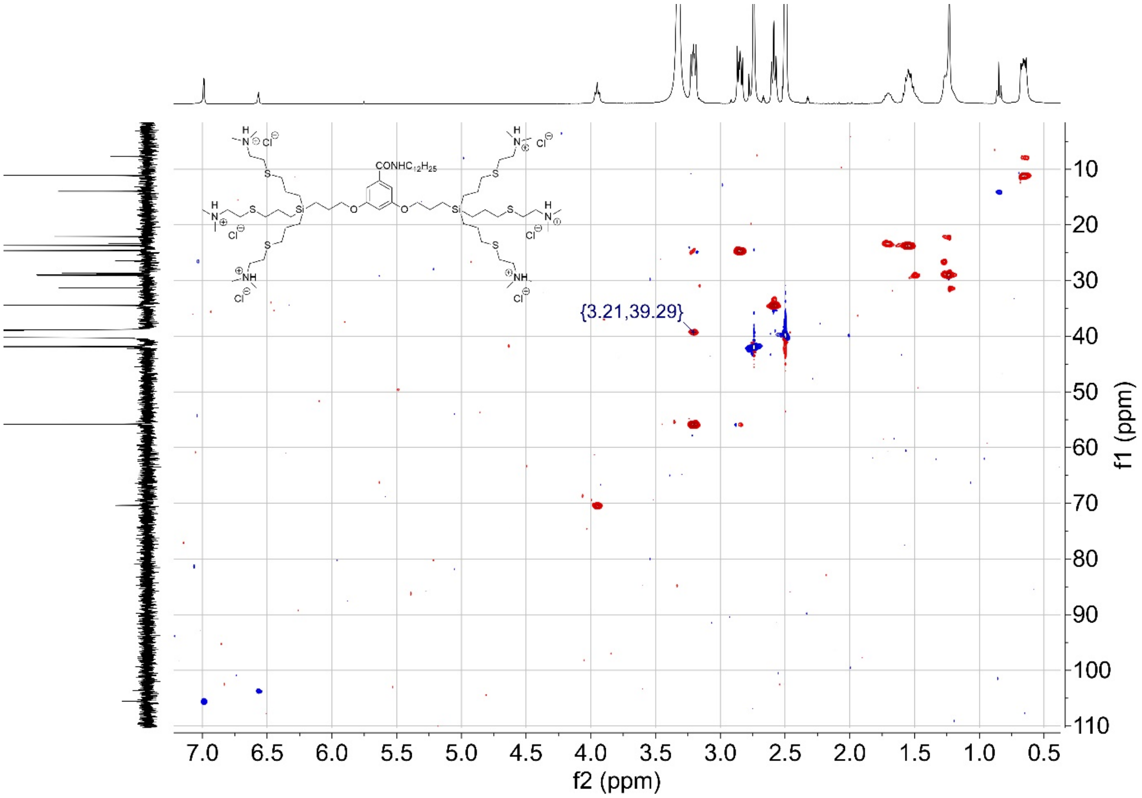

Figure S67. HSQC (400 MHz, DMSO-*d*6) of **L2-19a**

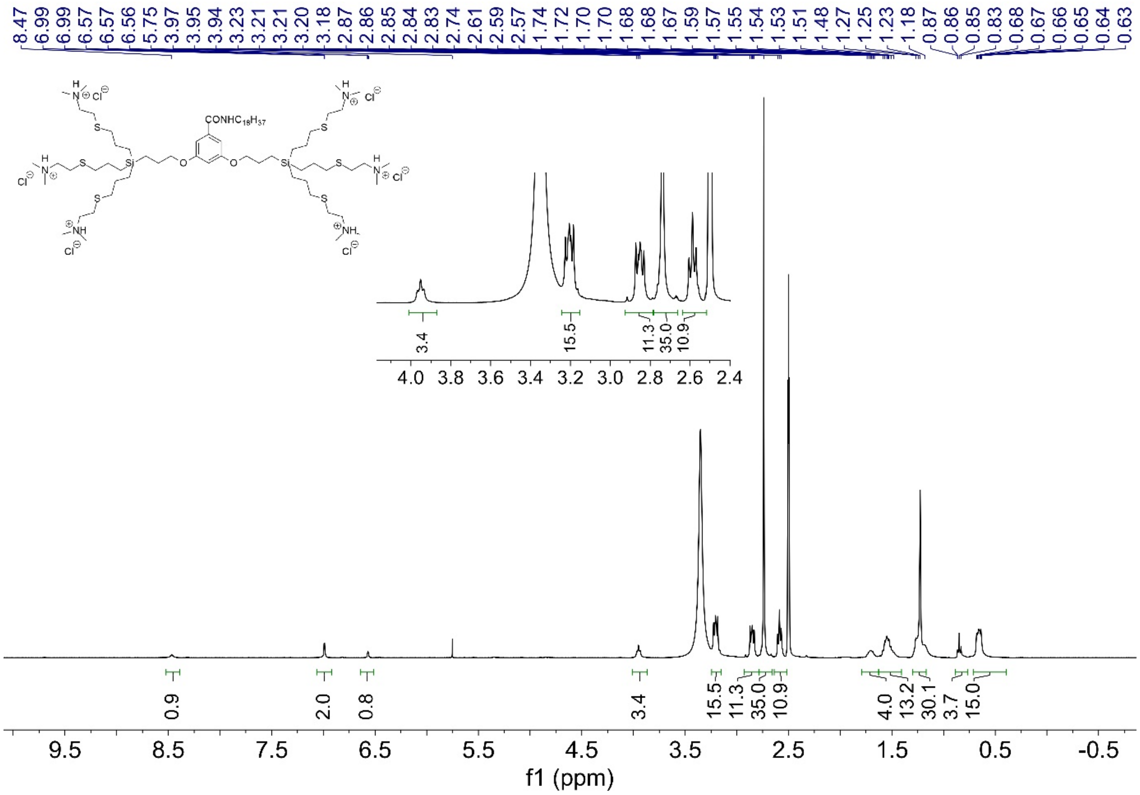

Figure S68. ^{1}H (400 MHz, DMSO-d_6) of **L2-19b**

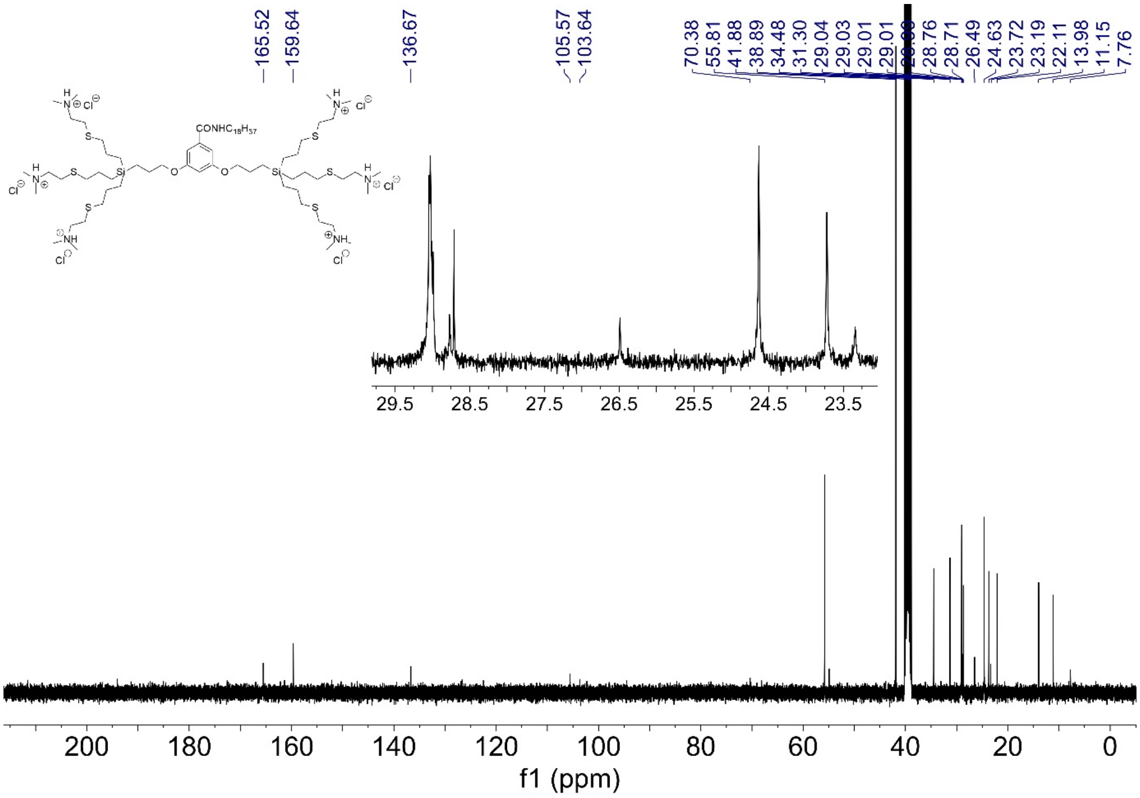

Figure S69. ^{13}C (400 MHz, DMSO-d_6) of **L2-19b**

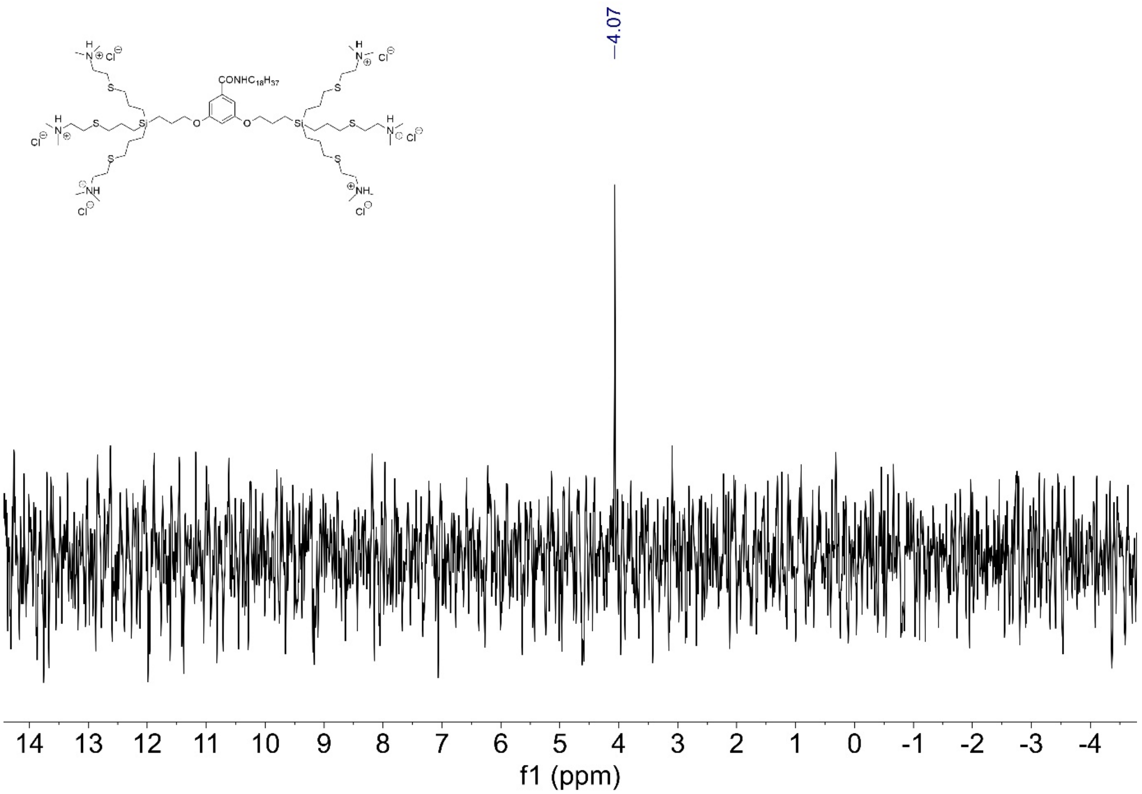

Figure S70. ^{29}Si (400 MHz, DMSO-d_6) of **L2-19b**

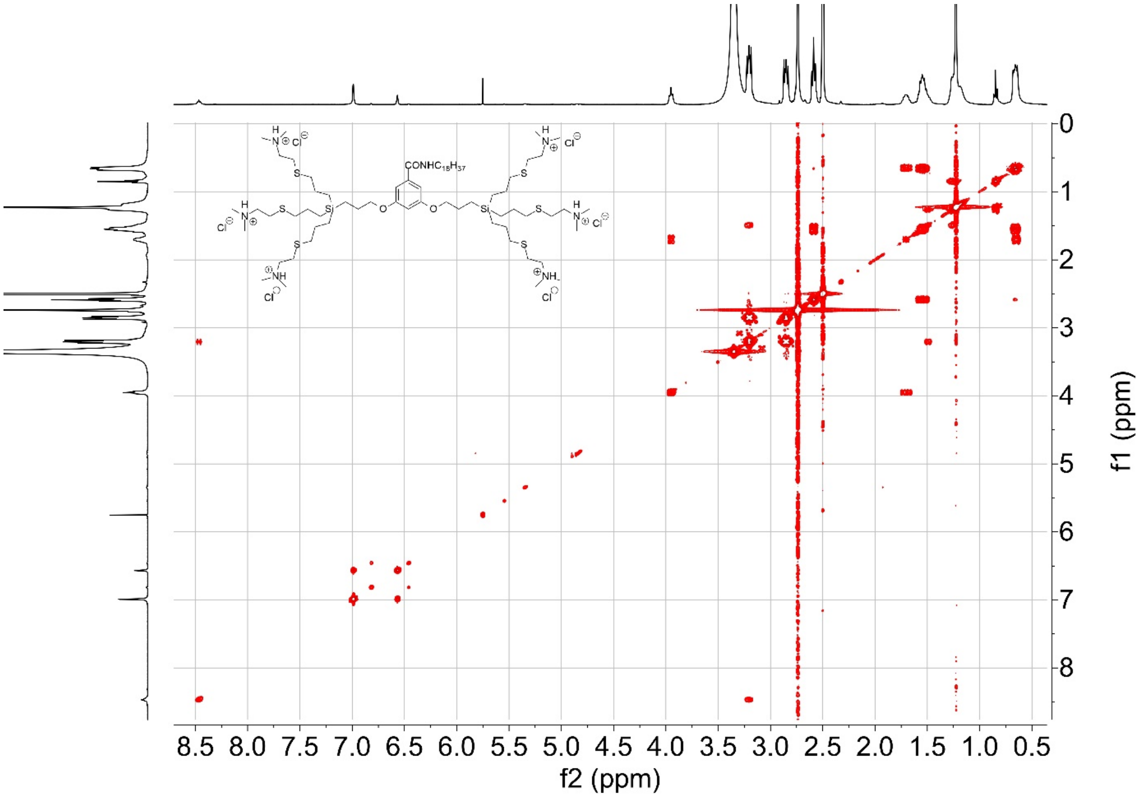

Figure S71. COSY (400 MHz, DMSO-d_6) of **L2-19b**

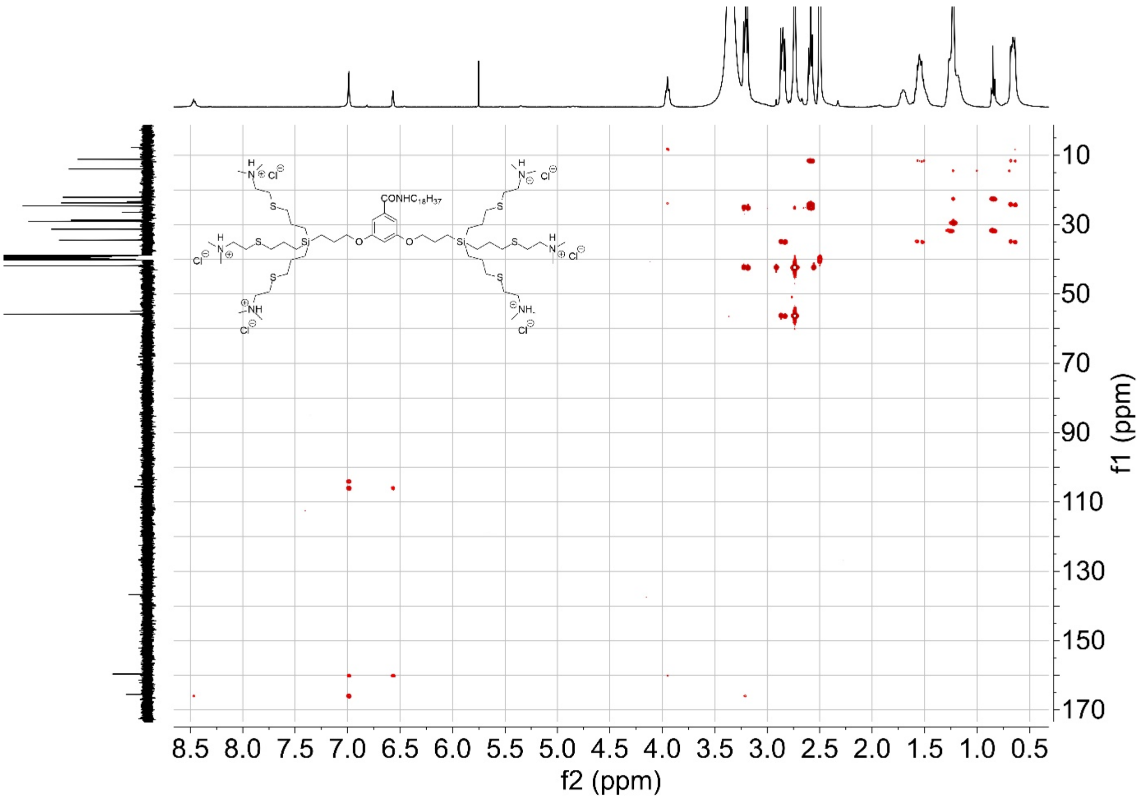

Figure S72. HMBC (400 MHz, DMSO-d_6) of **L2-19b**

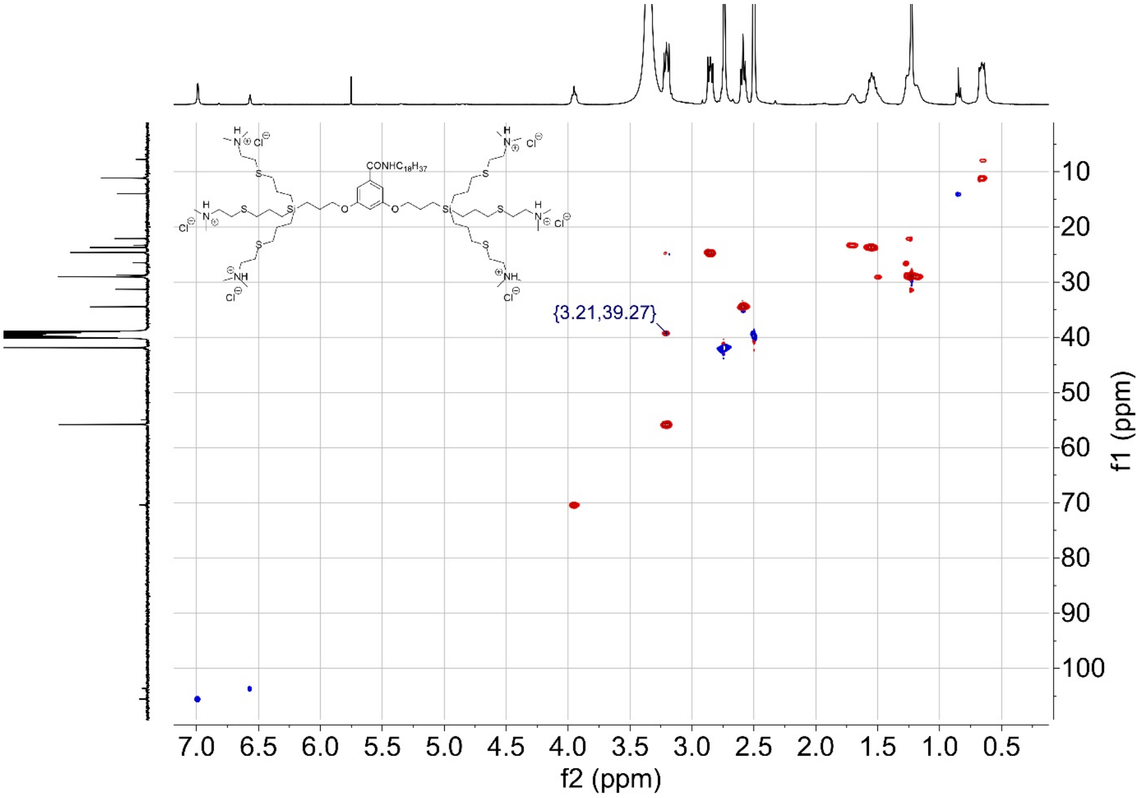

Figure S73. HSQC (400 MHz, DMSO-d_6) of **L2-19b**

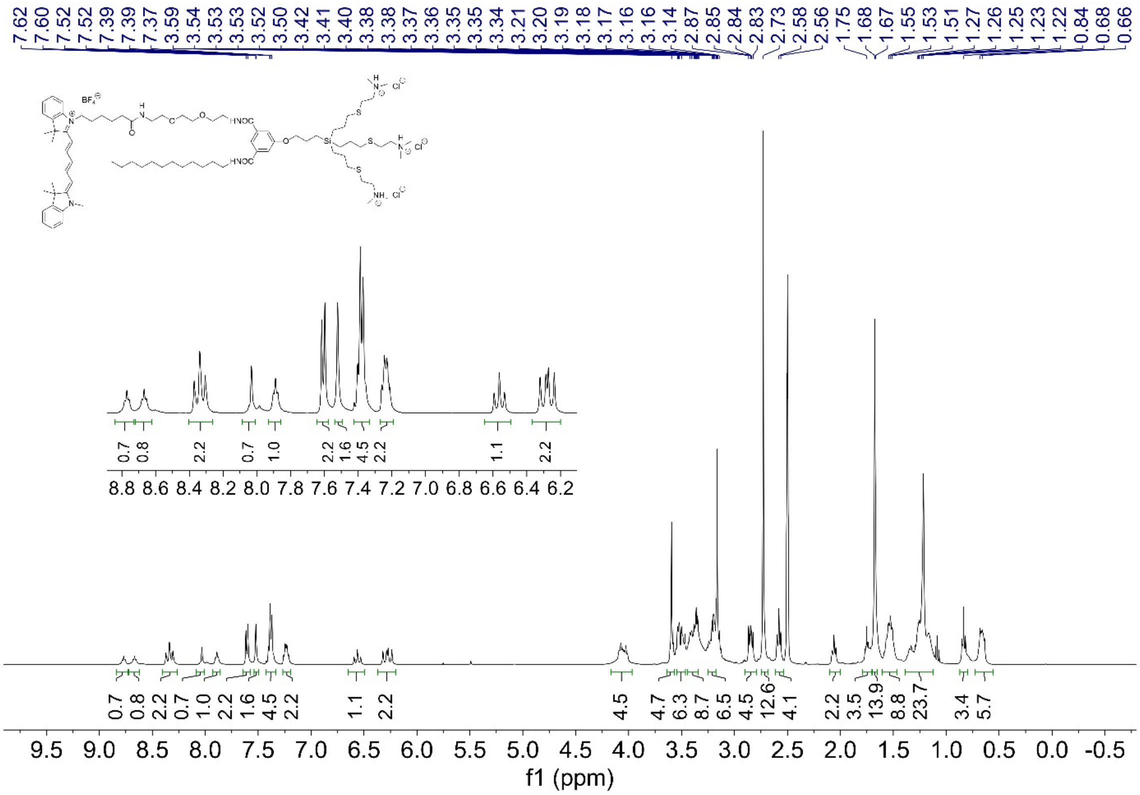

Figure S74. ^{1}H (400 MHz, DMSO-d_6) of **L3-28**

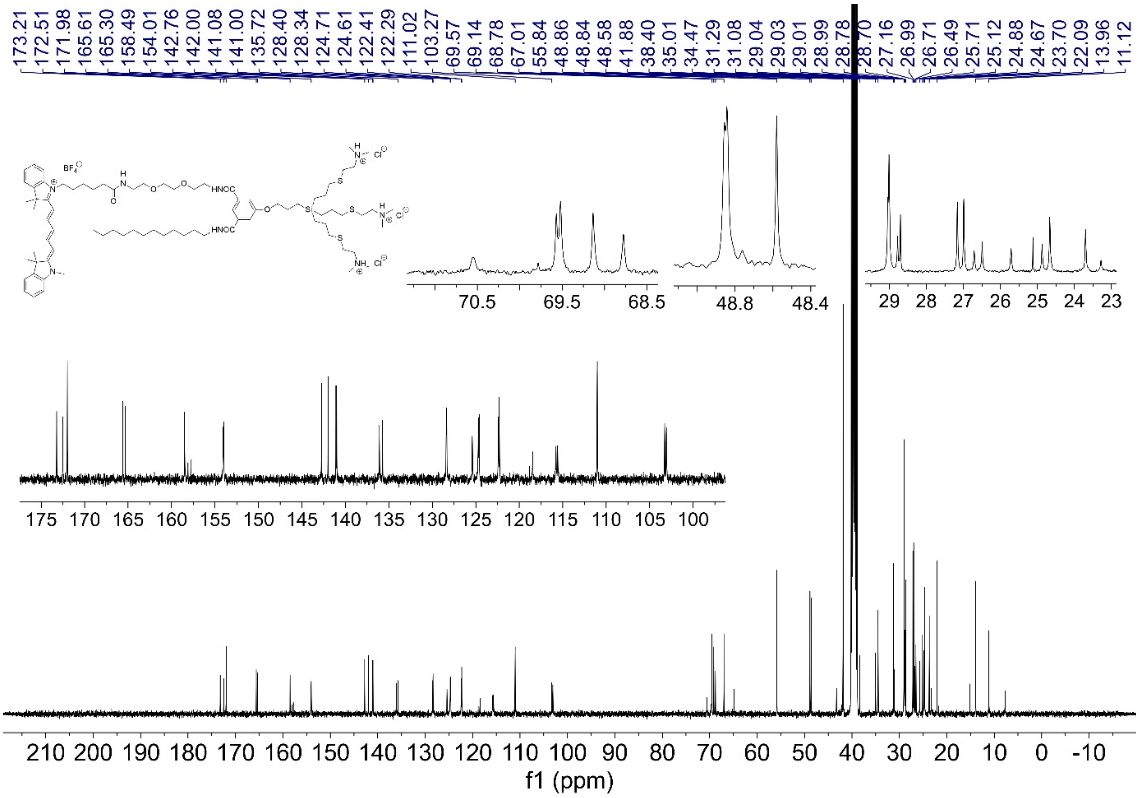

Figure S75. ^{13}C (400 MHz, DMSO-d_6) of **L3-28**

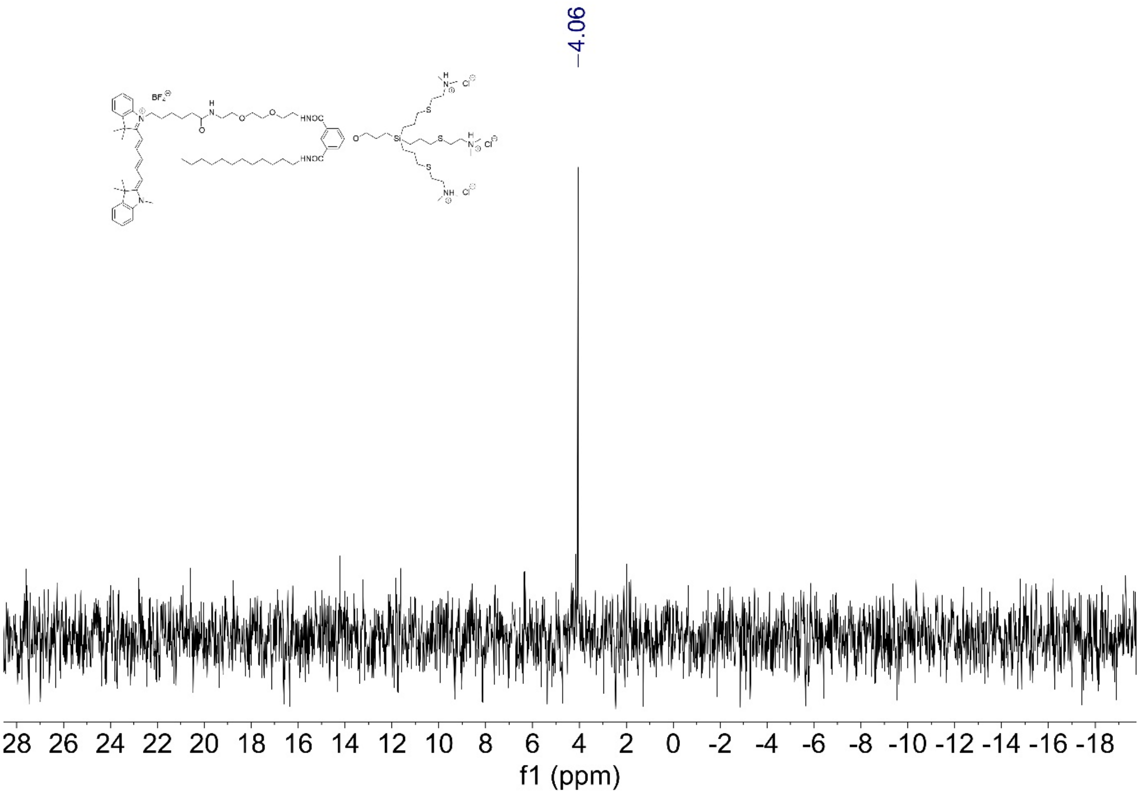

Figure S76. ^{29}Si (400 MHz, DMSO-d_6) of **L3-28**

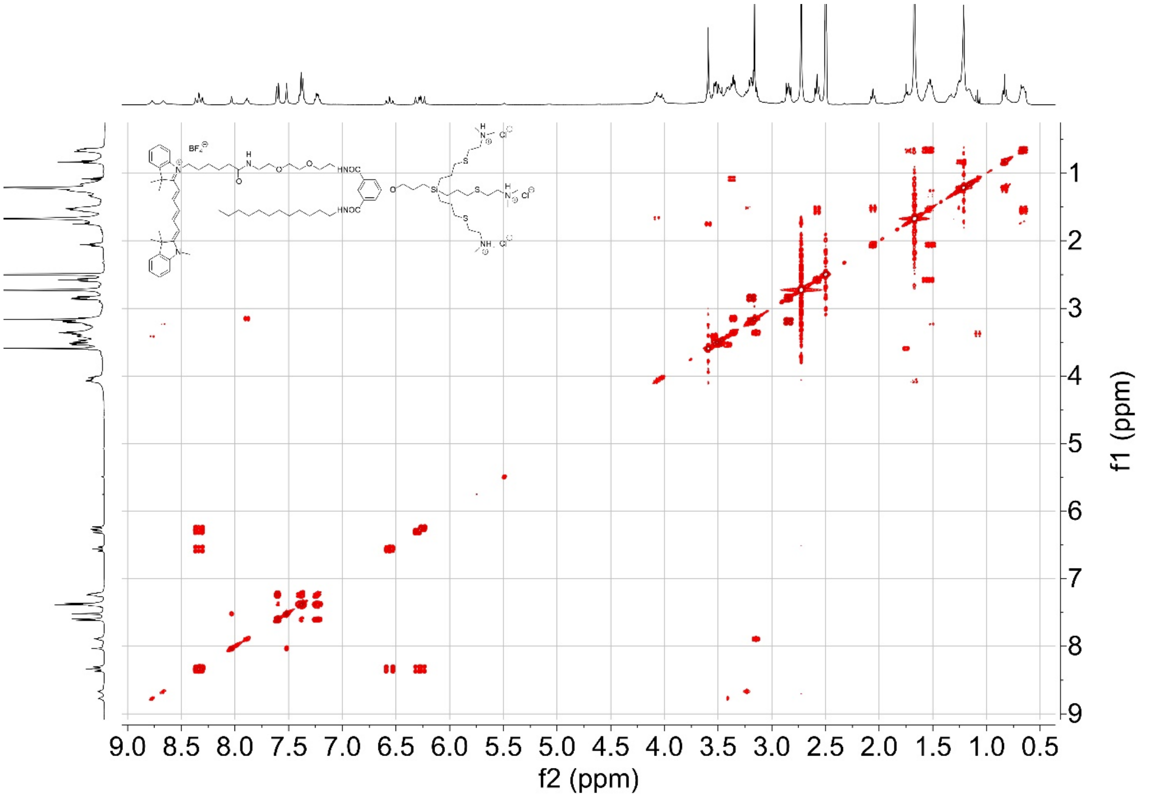

Figure S77. COSY (400 MHz, DMSO-d_6) of **L3-28**

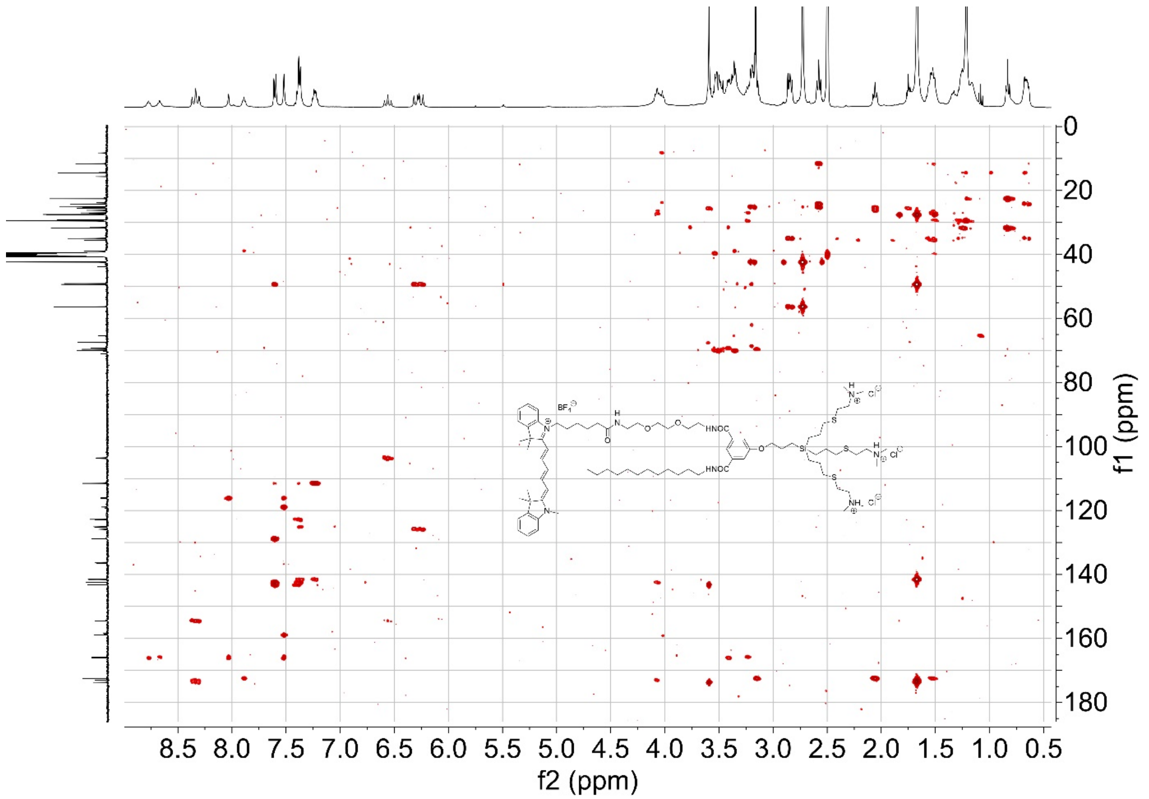

Figure S78. HMBC (400 MHz, DMSO-d_6) of **L3-28**

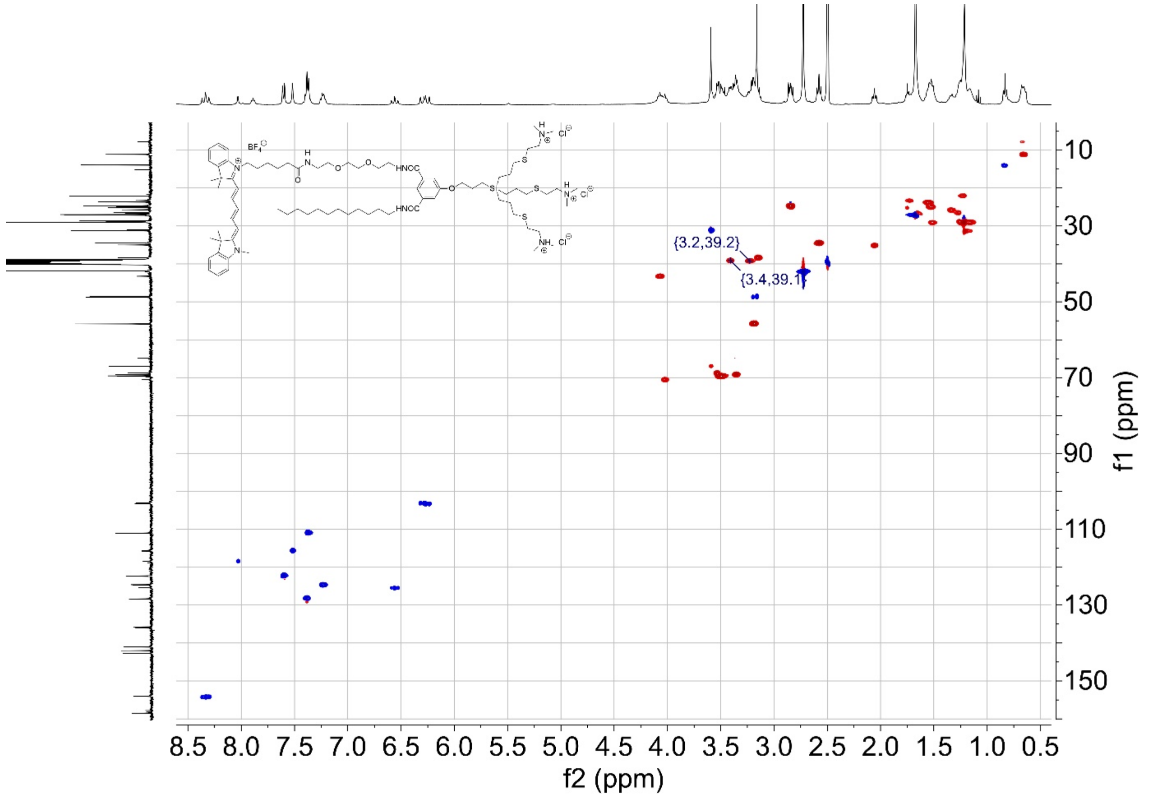

Figure S79. HSQC (400 MHz, DMSO-d_6) of **L3-28**

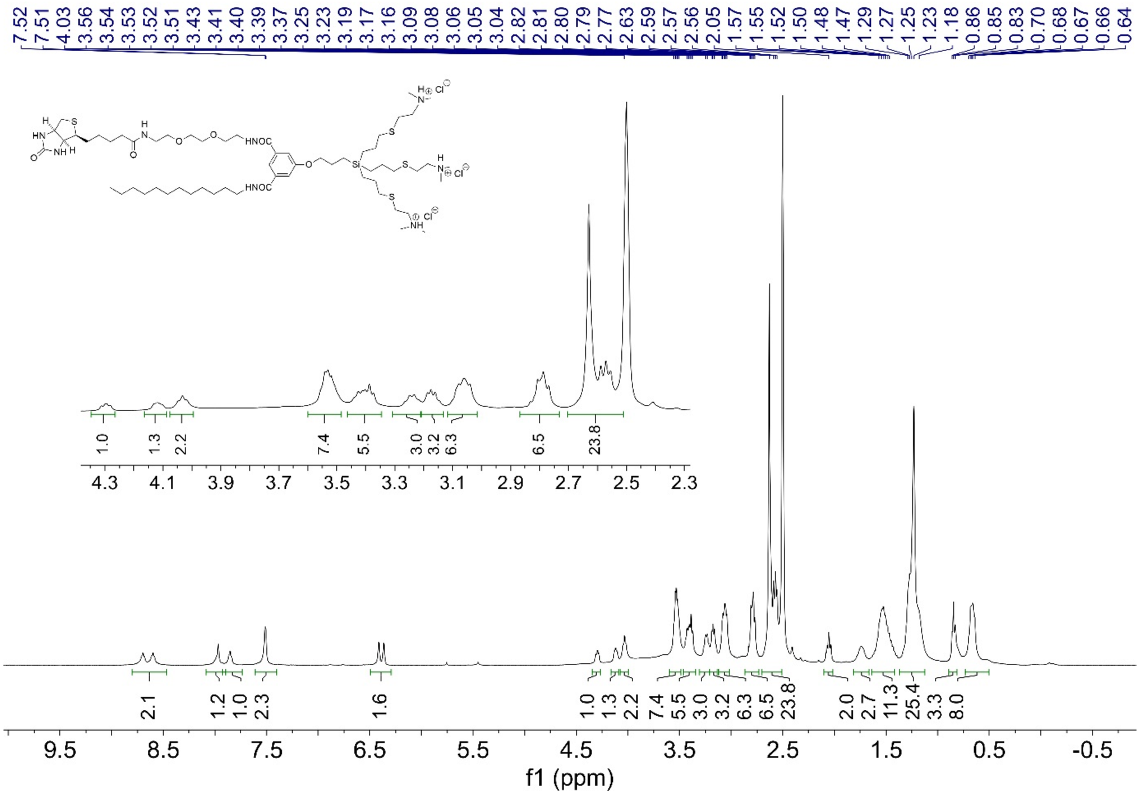

Figure S80. ^{1}H (400 MHz, DMSO-d_6) of **L3-29**

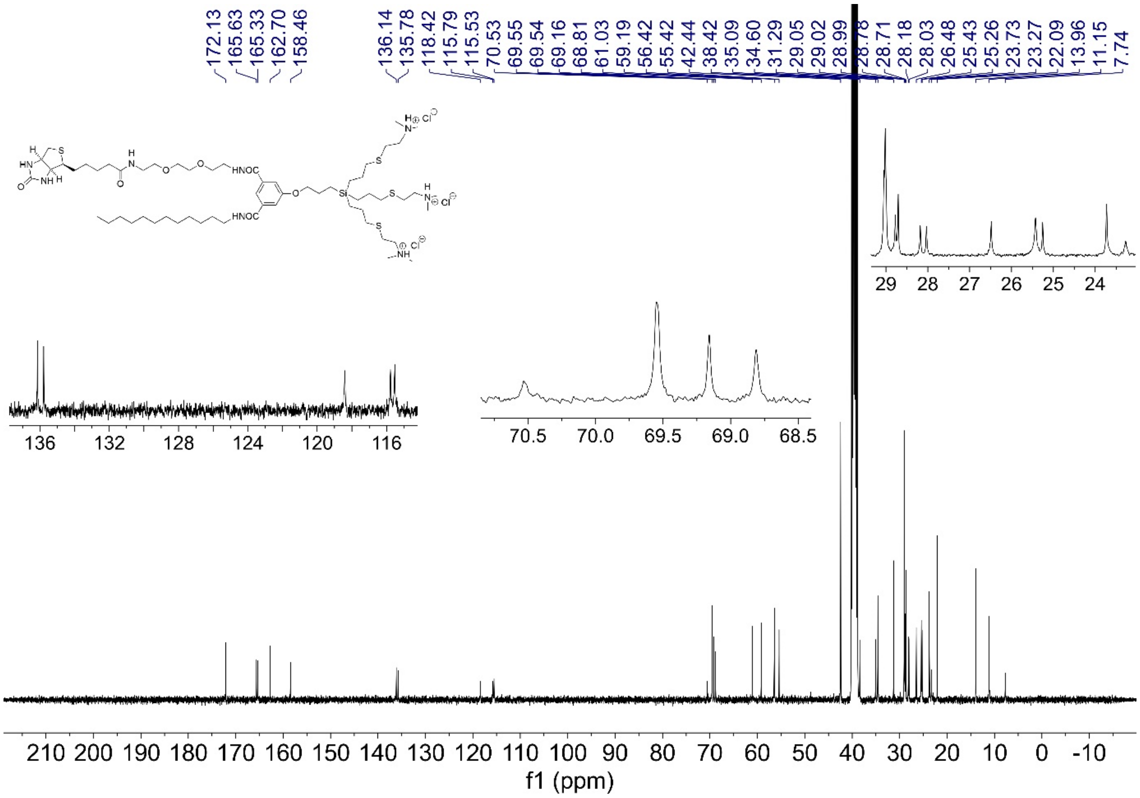

Figure S81. ^{13}C (400 MHz, DMSO-d_6) of **L3-29**

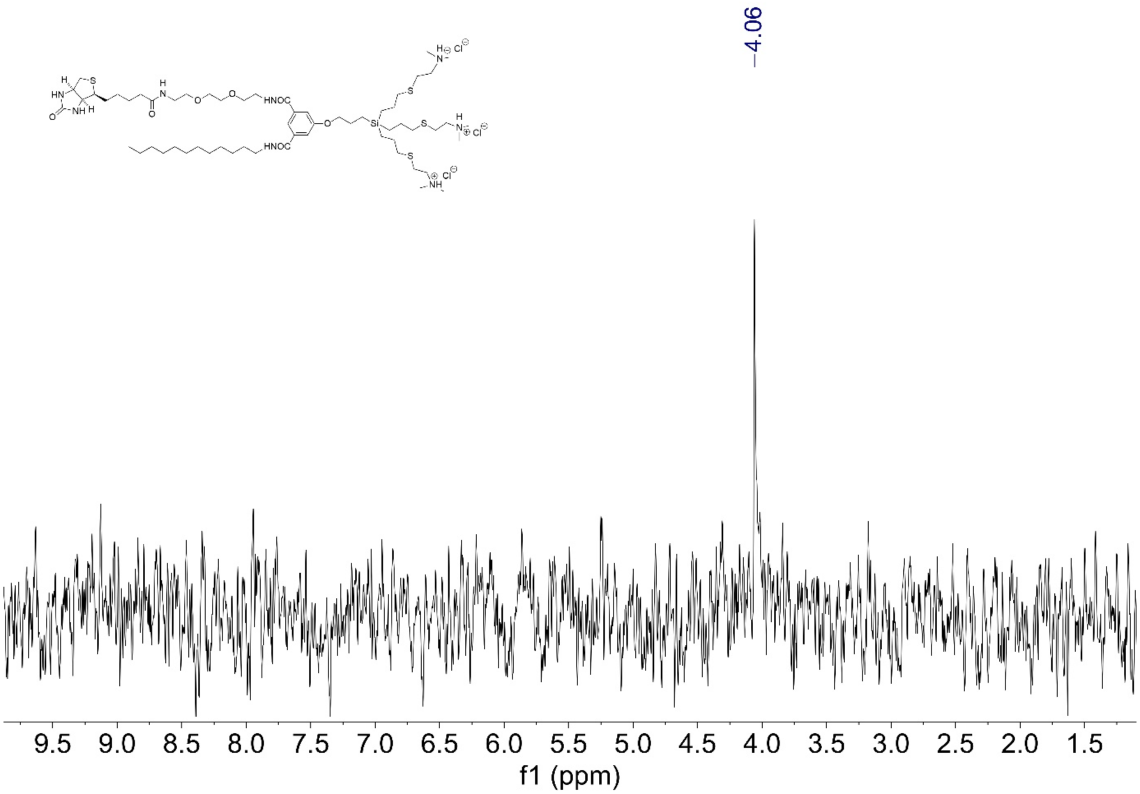

Figure S82. ^{29}Si (400 MHz, DMSO-d_6) of **L3-29**

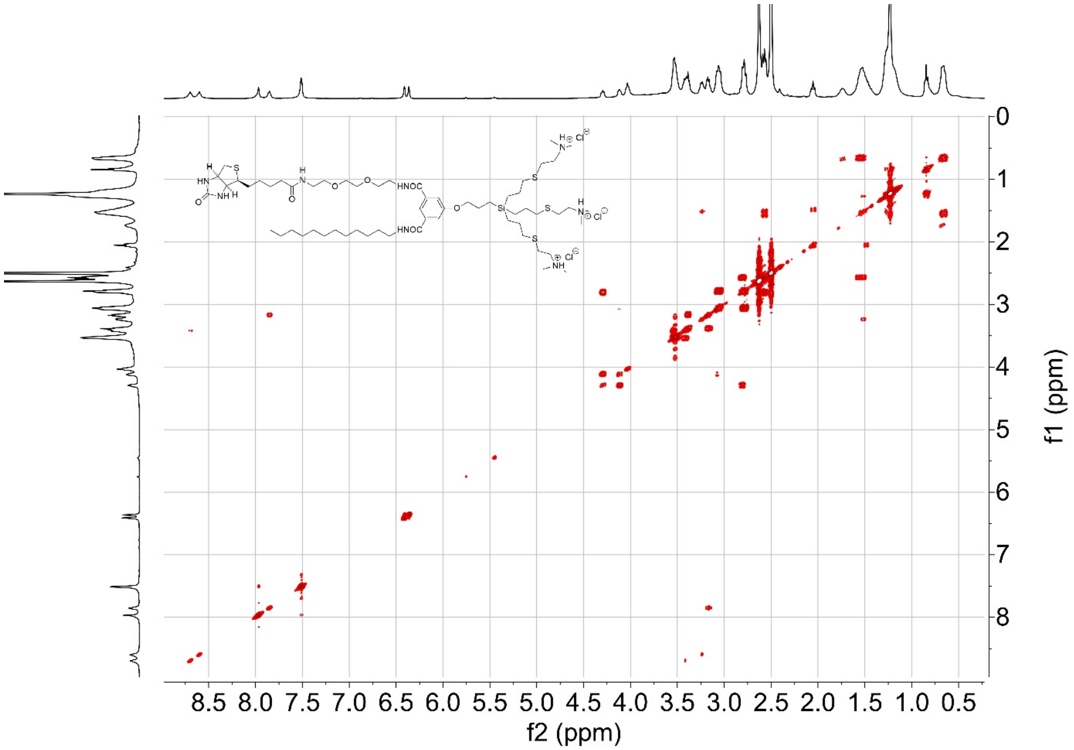

Figure S83. COSY (400 MHz, DMSO-d_6) of **L3-29**

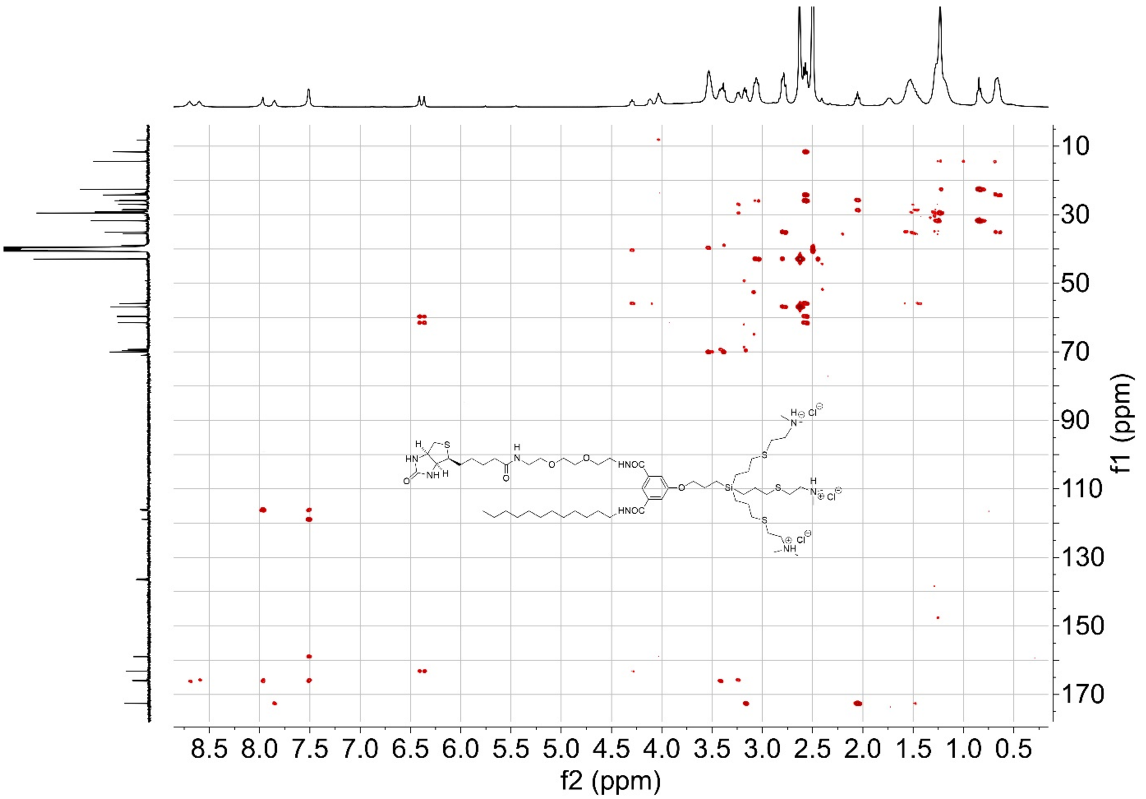

Figure S84. HMBC (400 MHz, DMSO-d_6) of **L3-29**

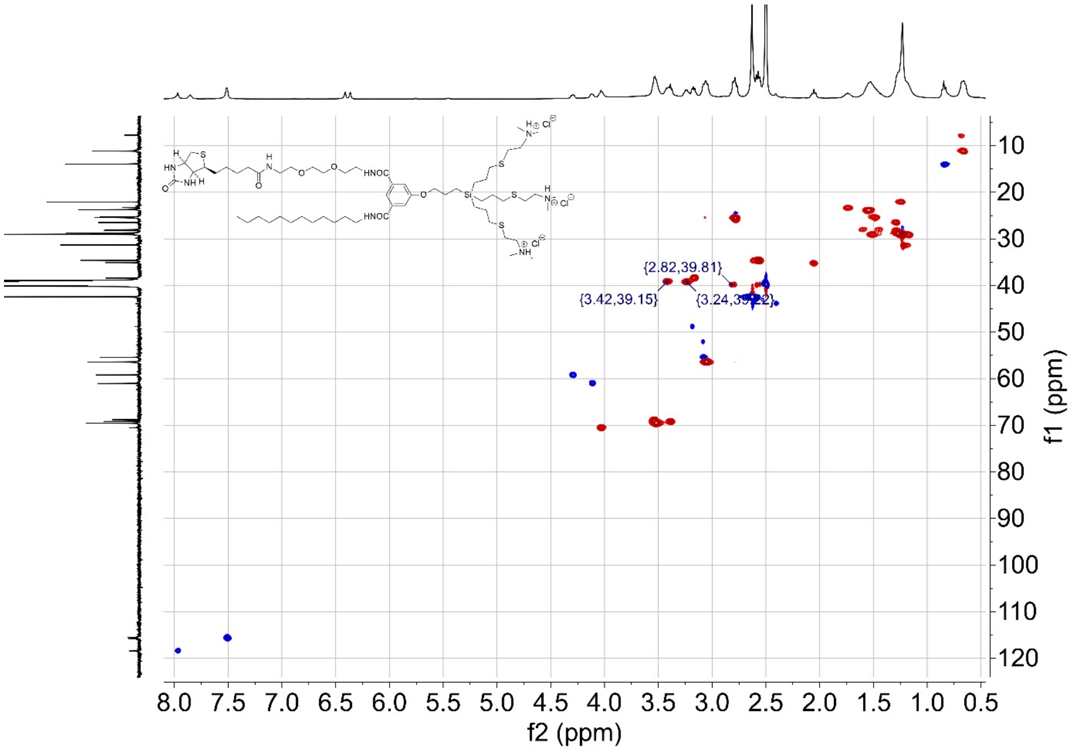

Figure S85. HSQC (400 MHz, DMSO-d_6) of **L3-29**

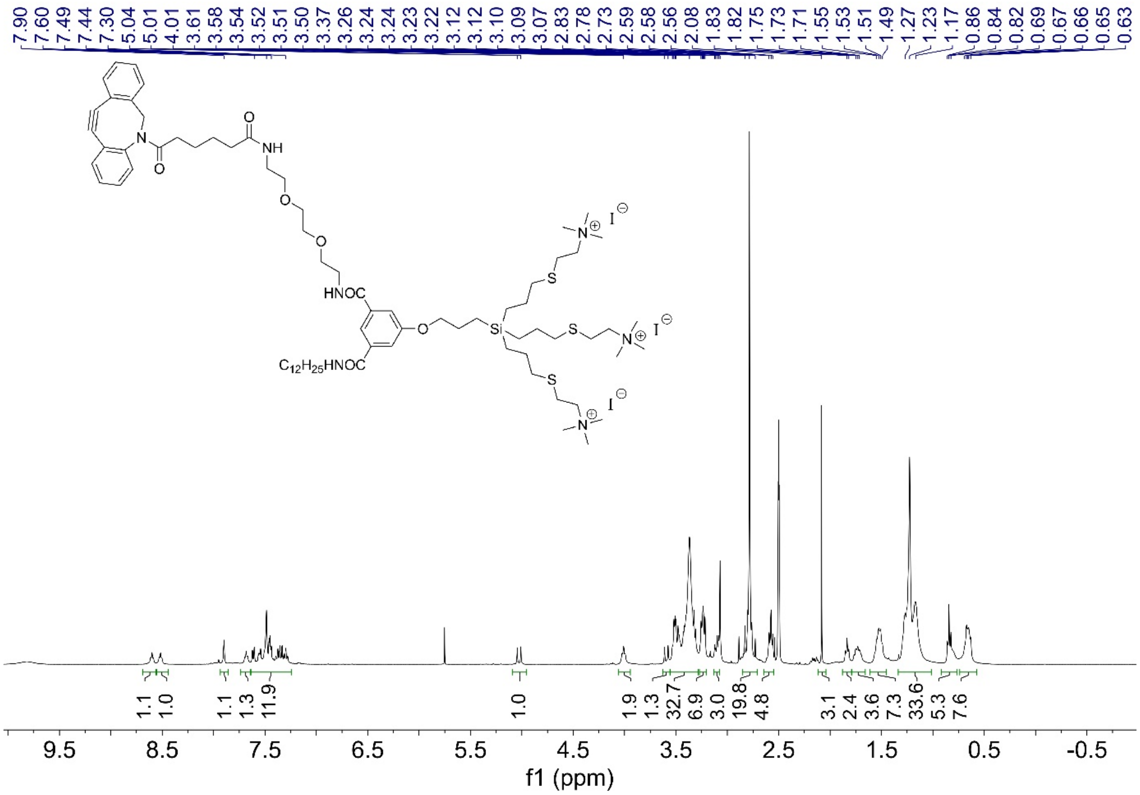

Figure S86. ^{1}H (400 MHz, DMSO-d_6) of **L3-30**

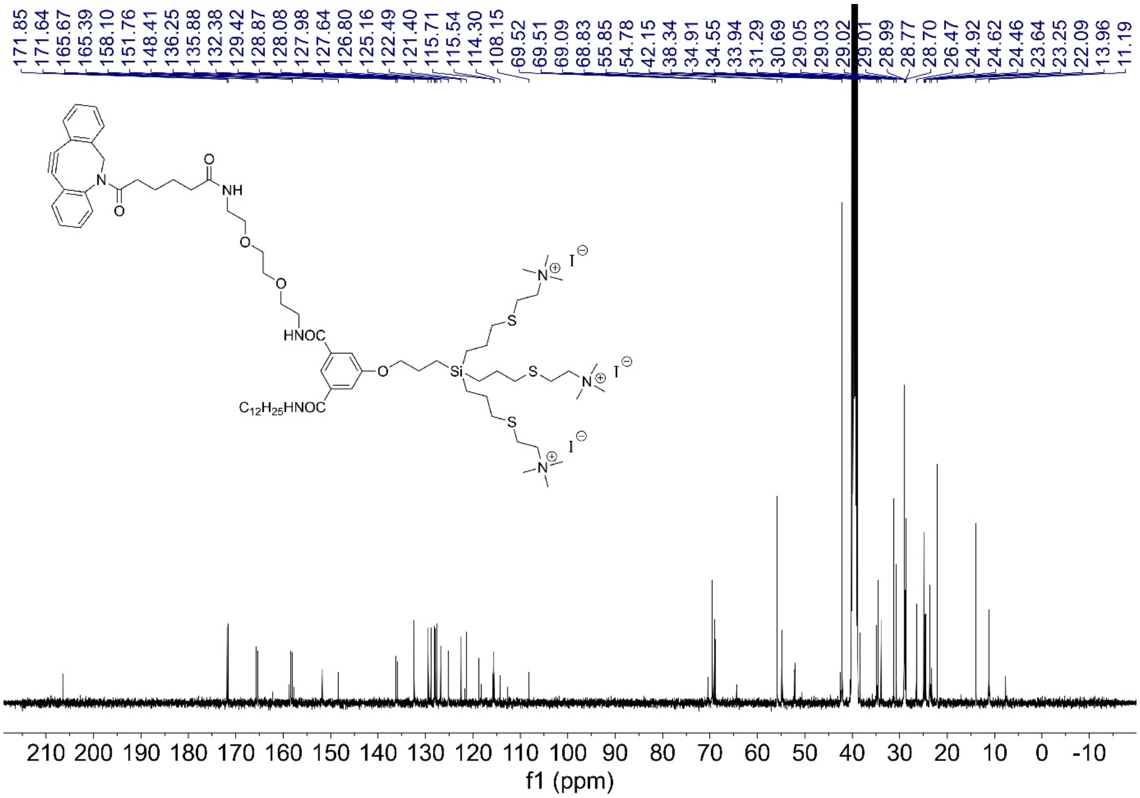

Figure S87. ^{13}C (400 MHz, DMSO-d_6) of **L3-30**

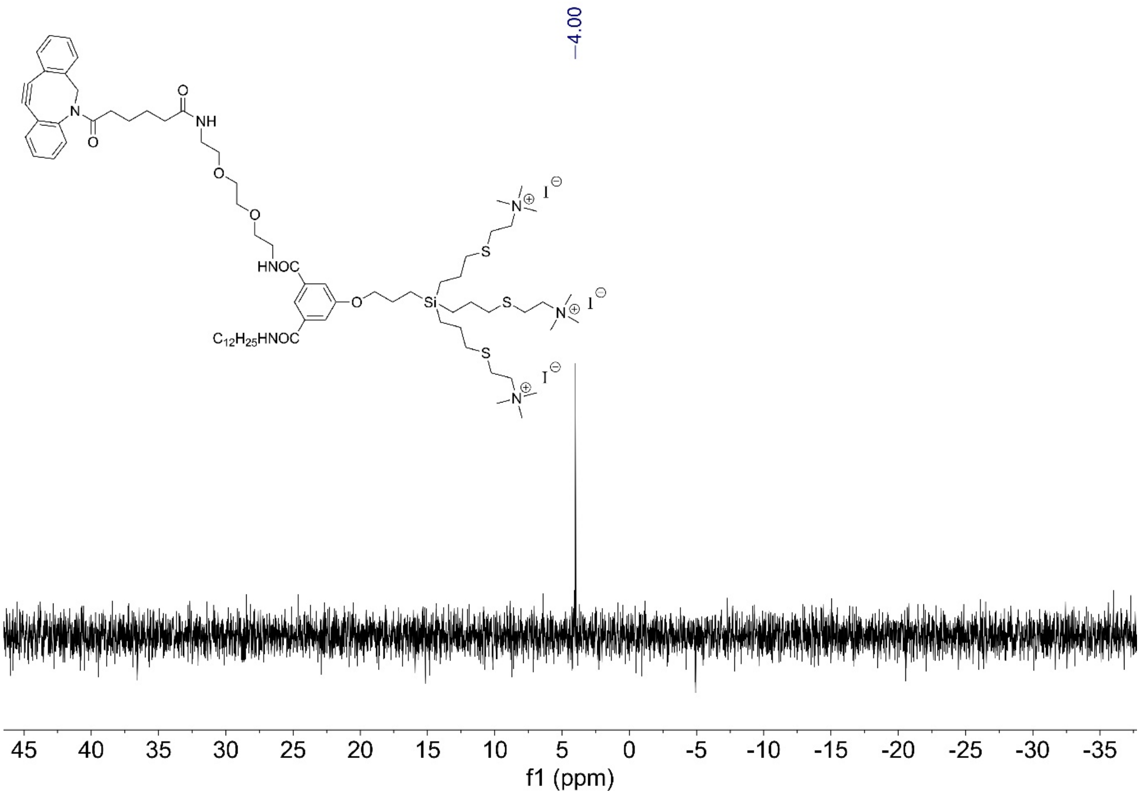

Figure S88. ^{29}Si (400 MHz, DMSO-d_6) of **L3-30**

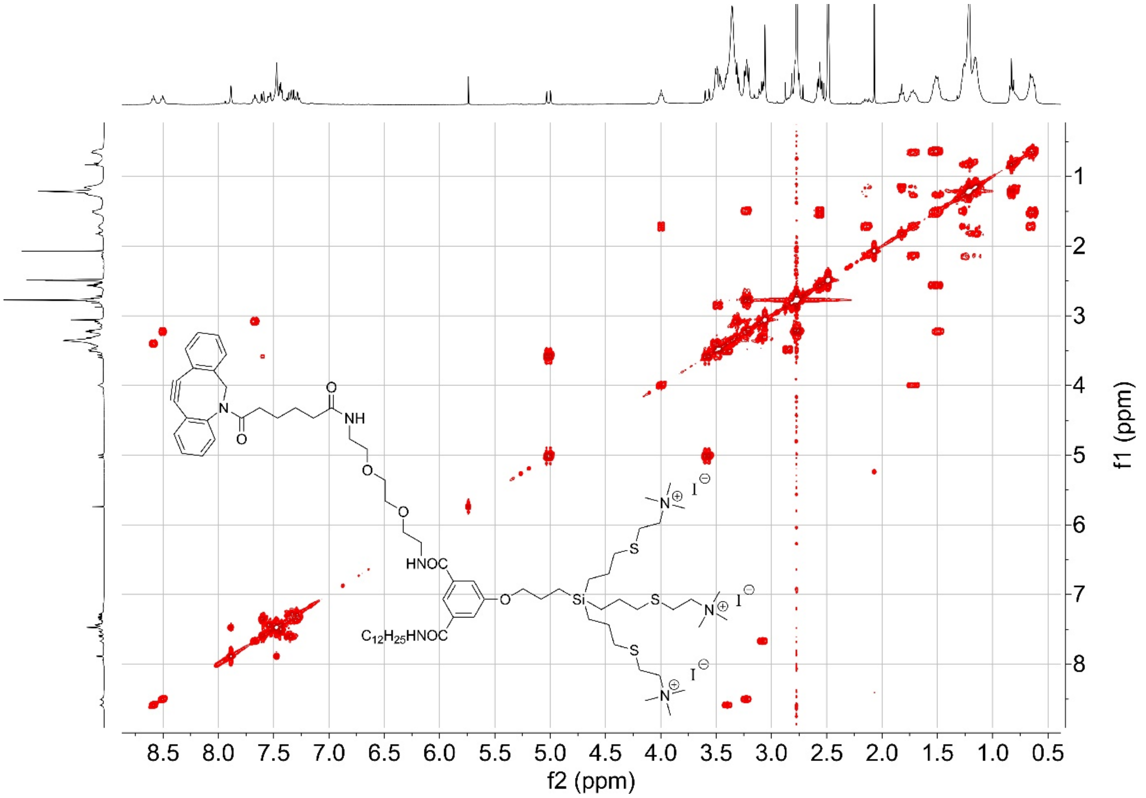

Figure S89. COSY (400 MHz, DMSO-d_6) of **L3-30**

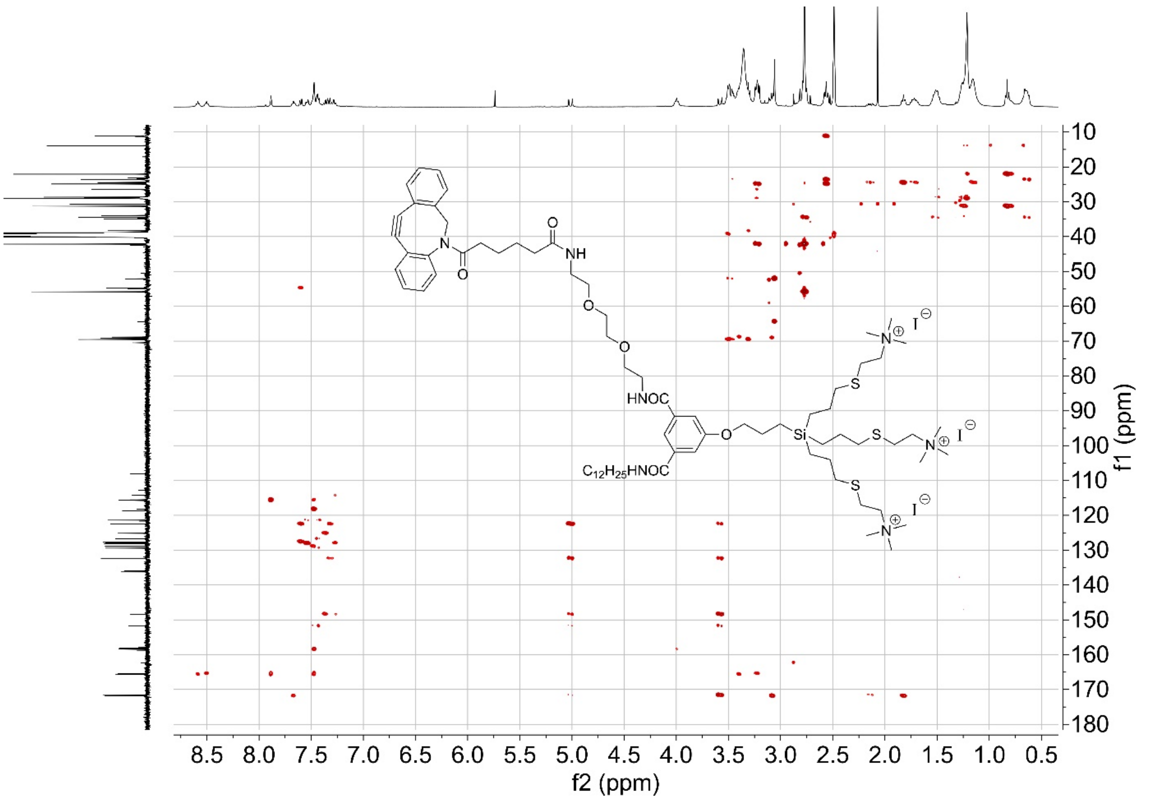

Figure S90. HMBC (400 MHz, DMSO-d_6) of **L3-30**

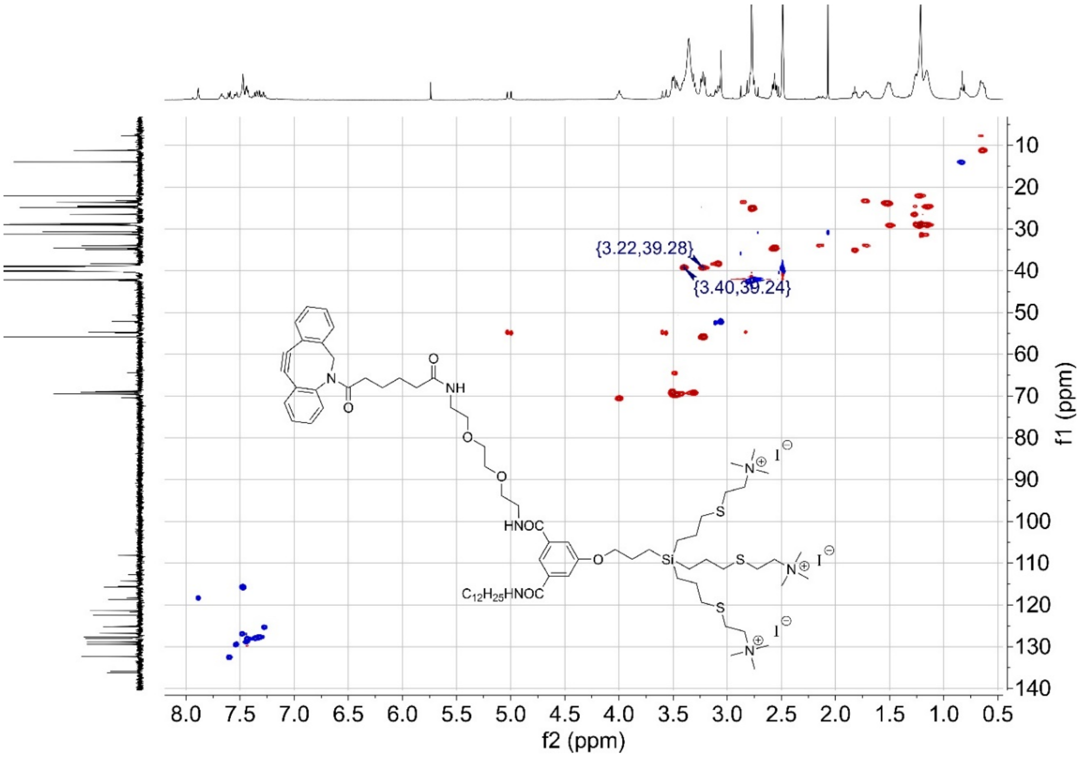

Figure S91. HSQC (400 MHz, DMSO-d_6) of **L3-30**

4. Mass spectrum of compound L3-31

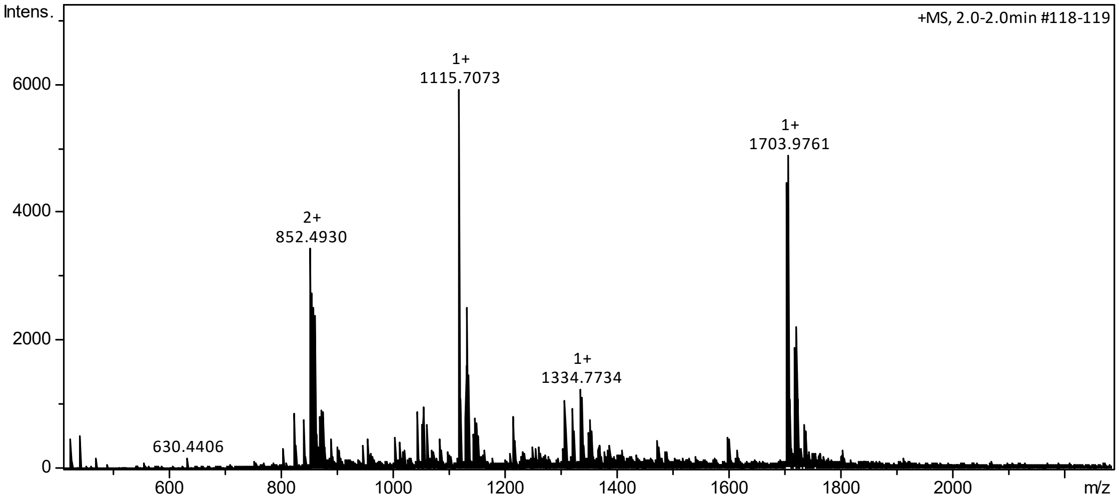

Figure S92. HRMS ESI+ of crude product **L3-31**
([M+H]$^+$ calcd. 1703.9783; [M+2H]$^{2+}$ calcd. 852.4928)
